# JCS/JHRS 2020 Guideline on Pharmacotherapy of Cardiac Arrhythmias

**DOI:** 10.1002/joa3.12714

**Published:** 2022-10-25

**Authors:** Katsushige Ono, Yu‐ki Iwasaki, Masaharu Akao, Takanori Ikeda, Kuniaki Ishii, Yasuya Inden, Kengo Kusano, Yoshinori Kobayashi, Yukihiro Koretsune, Tetsuo Sasano, Naokata Sumitomo, Naohiko Takahashi, Shinichi Niwano, Nobuhisa Hagiwara, Ichiro Hisatome, Tetsushi Furukawa, Haruo Honjo, Toru Maruyama, Yuji Murakawa, Masahiro Yasaka, Eiichi Watanabe, Takeshi Aiba, Mari Amino, Hideki Itoh, Hisashi Ogawa, Yasuo Okumura, Chizuko Aoki‐Kamiya, Jun Kishihara, Eitaro Kodani, Takashi Komatsu, Yusuke Sakamoto, Kazuhiro Satomi, Tsuyoshi Shiga, Tetsuji Shinohara, Atsushi Suzuki, Shinya Suzuki, Yukio Sekiguchi, Satoshi Nagase, Noriyuki Hayami, Masahide Harada, Tadashi Fujino, Takeru Makiyama, Mitsunori Maruyama, Junichiro Miake, Shota Muraji, Hiroshige Murata, Norishige Morita, Hisashi Yokoshiki, Koichiro Yoshioka, Kenji Yodogawa, Hiroshi Inoue, Ken Okumura, Takeshi Kimura, Hiroyuki Tsutsui, Wataru Shimizu, the Japanese Circulation Society and, Japanese Heart Rhythm Society Joint Working Group

**Abbreviations**
ACS: acute coronary syndrome
ADME: absorption, distribution, metabolism, excretion
AHRE: atrial high rate episode
AIVR: accelerated idioventricular rhythm
APD: action potential duration
APTT: activated partial thromboplastin time
ARB: angiotensin II receptor blocker
ATP: adenosine triphosphate
AVRT: atrioventricular reciprocating tachycardia
BMI: body mass index
BNP: B‐type natriuretic peptide
CABG: coronary artery bypass grafting
CCr: creatinine clearance
CKD: chronic kidney disease
COPD: chronic obstructive pulmonary disease
CPR: cardiopulmonary resuscitation
CPVT: catecholaminergic polymorphic ventricular tachycardia
DAD: delayed afterdepolarization
DAPT: dual antiplatelet therapy
dTT: dilute thrombin time
DOAC: direct oral anticoagulant
EAD: early afterdepolarization
ES: electrical storm
ECA: ecarin chromogenic assay
ESUS: embolic stroke of undetermined source
HBR: high bleeding risk
HFpEF: heart failure with preserved ejection fraction
HFrEF: heart failure with reduced ejection fraction
ICD: implantable cardioverter‐defibrillator
INR: international normalized ratio
LQTS: long QT syndrome
LVEF: left ventricular ejection fraction
OAC: oral anticoagulant
OSA: obstructive sleep apnea
PCI: percutaneous coronary intervention
PEA: pulseless electrical activity
PJRT: permanent junctional reciprocating tachycardia
PT: prothrombin time
pVT: pulseless ventricular tachycardia
RAS: renin–angiotensin system
ROSC: return of spontaneous circulation
rt‐PA: recombinant tissue‐type plasminogen activator
SQTS: short QT syndrome
SR: sarcoplasmic reticulum
TdP: torsade de pointes
TIA: transient ischemic attack
TTR: time in therapeutic range
Vd: volume of distribution
VT: ventricular tachycardia
WPW: Wolff‐Parkinson‐White

## 1 TABLE OF CONTENTS

**Table:** 

Preamble	2
I. Mechanisms of Arrhythmia Development and Clinical Pharmacology of Antiarrhythmic Drugs	4
1. Mechanisms of Arrhythmia Development (Abnormal Automaticity, Triggered Activity, and Reentry)	4
2. Classification and Mechanism of Action of Antiarrhythmic Drugs	5
3. Pharmacokinetics (Absorption, Distribution, Metabolism, and Elimination) and Pharmacodynamics	7
4. Side Effects of Antiarrhythmic Drugs and Countermeasures (Excluding Drug‐Induced Long QT Syndrome)	11
II. Bradyarrhythmias	13
1. Clinical Presentation of Bradyarrhythmias	14
2. General Principles of Management of Bradyarrhythmias	14
3. Pharmacological Therapy for Bradyarrhythmias	14
III. Premature Contractions	15
1. Supraventricular Premature Contractions	15
2. Premature Ventricular Contractions	15
IV. Paroxysmal Supraventricular Tachycardia (Atrioventricular Nodal Reentrant Tachycardia, Atrioventricular Reciprocating Tachycardia, Atypical Supraventricular Tachycardias)	17
1. Drugs for Differential Diagnosis of Narrow QRS Tachycardia	17
2. Acute Treatment of Supraventricular Tachycardia	18
3. Prophylactic Therapy	19
4. Atypical Supraventricular Tachycardias	20
V. Atrial Fibrillation	20
1. Epidemiology, Pathophysiology and Electrophysiological Mechanism of Atrial Fibrillation	20
2. Basic Strategy for Diagnosis and Management	23
3. Anticoagulation Therapy	30
4. Rate Control Therapy	48
5. Rhythm Control Therapy	51
6. Upstream Therapy	58
7. Indication and Timing of Non‐Pharmacological Therapy	59
VI. Atrial Flutter/Atrial Tachycardia	61
1. Atrial Tachycardia	61
2. Atrial Flutter	63
VII. Ventricular Tachycardia	67
1. Epidemiology/Pathophysiology/Electrophysiology	67
2. Idiopathic Ventricular Tachycardia	67
3. Ventricular Tachycardia Associated With Organic Heart Disease	69
4. Polymorphic Ventricular Tachycardia in Cases Without QT Prolongation	71
VIII. Polymorphic Ventricular Tachycardia/Torsade de Pointes	72
1. Congenital Long QT Syndrome	72
2. Acquired Long QT Syndrome	73
IX. Ventricular Fibrillation and Ventricular Tachycardia Associated With Special Diseases	74
1. Brugada Syndrome and Early Repolarization Syndrome	74
2. Catecholaminergic Polymorphic Ventricular Tachycardia	76
3. Other Inherited Arrhythmias (Short QT Syndrome)	77
X. Ventricular Fibrillation/Pulseless Ventricular Tachycardia/Cardiac Arrest	79
1. Treatment	79
2. Antiarrhythmic Therapy	79
3. Treatment by Antiarrhythmic Drugs After Return of Spontaneous Circulation	81
XI. Arrhythmias in Pediatrics	81
1. Narrow QRS Tachycardia	82
2. Wide QRS Tachycardia	86
3. Postoperative Arrhythmias in Congenital Heart Disease	88
XII. Arrhythmias During Pregnancy	89
1. Superior Ventricular Extrasystole/Ventricular Extrasystole	89
2. Supraventricular Tachycardia	89
3. Atrial Fibrillation/Atrial Flutter	89
4. Ventricular Tachycardia	89
5. Inherited Arrhythmias	90
6. Bradycardia	90
References	90
Appendix 1	121
Appendix 2	130
Appendix 3	132

## PREAMBLE

### 1 Background to the Update of the Guideline

The Japanese Circulation Society (JCS) published the “Guidelines for Pharmacological Treatment of Arrhythmia” in 2004, and a revised edition was published in 2009.^1^ Both guidelines have recommended that selection of the appropriate treatment should be based on the concept of the Sicilian Gambit approach. However, pharmacological therapy for arrhythmia based on the pharmacological action of antiarrhythmic agents does not always lead to appropriate therapy in real‐world clinical practice. On the other hand, the majority of randomized clinical trials (RCTs) are performed in Western countries, so some of the antiarhythmic drugs are not available in Japan and dose of the drugs will also be different from that used in Japan. In addition, because of differences in lifestyle and ethnicity, including genetic factors, the results of RCTs in Western countries might not directly apply to Japanese. To solve these issues, many large‐scale multicenter studies and nationwide registry studies have been performed since the J‐RHYTHM study, and recently, Japanese evidence associated with pharmacological treatment of arrhythmias has become available. It is important to consider the mechanisms of drug action and pharmacokinetics in order to choose the appropriate therapy with high effectiveness and safety for the patient. Therefore, in the present guideline, we introduce the Vaughan Williams classification as well as the Sicilian Gambit approach.

There are 2 major points of difference from the previous editions. One is the role of antiarrhythmic agents for the treatment of arrhythmia. The goal of pharmacological therapy is to improve the prognosis and quality of life (QOL) rather than merely termination and prevention of arrhythmia. The superiority of non‐pharmacological therapy with implantable cardioverter‐defibrillator for prevention of sudden cardiac death as compared with pharmacological therapy has been reported. However, the effect of non‐pharmacological therapies is limited in terms of the risk of recurrence, complications, and cost effectiveness for some patients. With the emphasis on health life expectancy, especially in aging societies, such as Japan, QOL‐targeted pharmacological therapy is essential for the patient with arrhythmia. The 2nd difference is the direct oral anticoagulation drugs that are now widespread in Japan. The prevalence of atrial fibrillation (AF) has increased and is now considered as a “common disease” in Japan. Therefore, more effective and safe treatment is needed for the patients with AF. Definition of the disease, risk stratification and indication for anticoagulation therapy are important for the patients with AF. In a previous guideline “Guidelines for Pharmacotherapy of Atrial Fibrillation (JCS 2013)”,^2^ mitral valve plasty without artificial valve was defined as “non‐valvular” and valve replacement with artificial valve including mechanical valve and bioprosthetic was defined as “valvular” Since then, evidence associated with valvular disease and AF has increased such that, in the present guideline, artificial valve using a bioprosthetic valve is defined as “non‐valvular”. Accordingly, a prosthetic valve in transcatheter aortic valve implantation in patients with severe aortic valve stenosis is considered as “non‐valvular”.

In recent guidelines by the European Society of Cardiology, American Heart Association, American College of Cardiology, Heart Rhythm Society, and Asia Pacific Heart Rhythm Society, CHA_2_DS_2_‐VASc is used for risk stratification of ischemic stroke and systemic thromboembolism. In the present guideline, the CHADS_2_ score was chosen for risk stratification and indication of the anticoagulation therapy.

This revised version of “2020 JCS/JHRS Guideline on Pharmacotherapy of Cardiac Arrhythmias” was prepared as a joint guideline by the JCS and the Japanese Heart Rhythm Society (JHRS).

### 2 General Principles

1. The main audience of the present guideline is cardiologists, but it will be useful for the general physician and emergency physician. There are many figures and flowcharts that would be useful in clinical practice.
2. The present guideline does not recommend the use of antiarrhythmic drugs but indicates general refer
3. ences of the pharmacological treatment for the patient with arrhythmia. The final decision should be made by physician based on the individual patient’s condition.
4. Antiarrhythmic drugs are chosen according to their importance and high prevalence in general practice. Not all drugs are indicated in the tables and flowcharts.
5. Recommendations of antiarrhythmic drugs are prioritized by evidence levels. It is important to note that some of the drugs are not approved for use in Japan.

### 3 Class of Recommendation and Level of Evidence

The present guideline investigated the recommendations and levels of evidence as described in the ACC/AHA/HRS guidelines (Tables 1 and 2). Guidelines published by the JCS have extensively used a common style that is highly consistent with Western guidelines. However, the Japan Council for Quality Health Care uses a different style in its Medical Information Network Distribution Service (MINDS) to show grades of recommendations and levels of evidence, as described in the “Minds Handbook for Clinical Practice Guideline Development 2007” (Tables 3 and 4).^3^ Therefore, in the present guideline both styles are used in the tables (class of recommendation, level of evidence, grade of recommendation [MINDS] and level of evidence [MINDS]). However, because the concepts of classification differe between the AHA/ACC/HRS guidelines and MINDS, some discrepancy in the evidence level is possible.

**Table 1:** Class of Recommendation

Class I	Evidence and/or general agreement that a given procedure or treatment is useful and effective
Class II	Conflicting evidence and/or a divergence of opinion about the usefulness/efficacy of the given procedure or treatment
Class IIa	Weight of evidence/opinion is in favor of usefulness/efficacy
Class IIb	Usefulness/efficacy is less well established by evidence/opinion
Class III	Evidence or general agreement that the given procedure or treatment is not useful/effective, and in some cases may be harmful

**Table 2:** Level of Evidence

Level A	Data derived from multiple randomized clinical trials or meta‐analyses
Level B	Data derived from a single randomized clinical trial or large‐scale nonrandomized studies
Level C	Consensus of opinion of the experts and/or small‐sized clinical studies, retrospective studies, and registries

**Table 3:** MINDS Grade of Recommendations

Grade A	Strongly recommended and supported by strong evidence
Grade B	Recommended with moderately strong supporting evidence
Grade C1	Recommended despite no strong supporting evidence
Grade C2	Not recommended because of the absence of strong supporting evidence
Grade D	Not recommended as evidence indicates that the treatment is ineffective or even harmful

(Adapted from MINDS Treatment Guidelines Selection Committee.^2a^)

**Table 4:** MINDS Levels of Evidence (Levels of Evidence in Literature on Treatment)

I	Systematic review/meta‐analysis of randomized controlled trials
II	One or more randomized controlled trials
III	Nonrandomized controlled trials
IVa	Analytical epidemiological studies (cohort studies)
IVb	Analytical epidemiological studies (case–control studies and cross‐sectional studies)
V	Descriptive studies (case reports and case series)
VI	Not based on patient data, or based on opinions from a specialist committee or individual specialists

(Adapted from MINDS Treatment Guidelines Selection Committee.^2a^)

This guideline conformed with the consistency among the JCS guideline series, especially “2018 JCS/JHRS Guideline on Non‐Pharmacotherapy of Cardiac Arrhythmias^4^ and Guidelines for Diagnosis and Management of Inherited Arrhythmias (JCS 2017)”.^5^

## 2 MECHANISMS OF A RRHY THMIA DEVELOPMENT AND CLINICAL PHARMACOLOGY OF ANTIARRHYTHMIC DRUGS

### 2.1 Mechanisms of Arrhythmia Development (Abnormal Automaticity, Triggered Activity, and Reentry)

Electrophysiological mechanisms of cardiac arrhythmia are divided into 2 categories: (1) abnormal impulse generation and (2) abnormal impulse conduction. The former includes abnormalities in automaticity and triggered activity, and the latter includes reentry. This section is an overview of these arrhythmogenic mechanisms.

#### 2.1.1 Normal and Abnormal Automaticity

**Figure 1:**
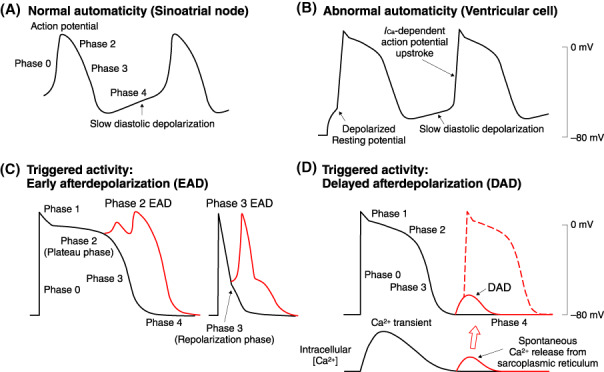
Normal (**A**) and abnormal (**B**) automaticity, and triggered activities mediated by either early afterdepolarization (**C**) or delayed afterdepolarization (**D**).

Automaticity is the ability of cardiac cells to undergo spontaneous slow diastolic depolarization and initiate an electrical impulse in the absence of external electrical stimulation.^6^ Normal automaticity occurs in a variety of cardiac tissues, including the sinoatrial node, some parts of the atria, pulmonary veins, the atrioventricular node (AVN) and the His–Purkinje system. Spontaneous depolarization is the result of the development of a net inward ionic current during the diastolic phase of the action potential (Figure 1A). Two mechanisms for spontaneous depolarization, which is referred to as a “clock”, are involved in spontaneous pacemaking: the membrane potential clock results from interactions of several sarcolemmal ion channels and transporters, and the Ca^2+^ clock is intracellular Ca^2+^ cycling mediated by sarcoplasmic reticulum Ca^2+^ release and uptake. *β*_1_‐adrenergic receptor stimulation by sympathetic nervous tone and catecholamines accelerates the intrinsic rate of automaticity by increasing the slope of the slow diastolic depolarization, whereas M_2_‐muscarinic receptor stimulation by parasympathetic nervous tone slows the intrinsic rate by decreasing the slope of the diastolic depolarization and hyperpolarizing the diastolic membrane potential.

Working myocardial cells in the atria and ventricles show abnormal automaticity when their resting membrane potential is partially depolarized (Figure 1B). The Ca^2+^ current, *I*_Ca_, is responsible for the spontaneous action potential upstroke through the the mechanism of abnormal automaticity.^6^ In the acute phase of myocardial ischemia, extracellular K^+^ accumulation‐induced partial membrane depolarization may cause abnormal automaticity in ventricular cells and Purkinje fibers in the ischemic border zone.

#### 2.1.2 Triggered Activity

Triggered activity is a term used to describe the initiation of electrical excitation from oscillatory afterdepolarizations that follow the action potential upstroke.^6^ Afterdepolarizations are classified into 2 groups: early afterdepolarizations (EADs) and delayed afterdepolarizations (DADs).

EADs are oscillations in membrane potential during the plateau phase (phase 2) or repolarization phase (phase 3) of the action potential (Figure 1C), as a consequence of either an increase in depolarizing inward currents, including *I*_Ca_, the late Na^+^ channel current, late *I*_Na_ and the Na^+^/Ca^2+^ exchange current *I*_NCX_, or a decrease in the outward currents, including the transient outward current, *I*_to_, the delayed rectifier K^+^ currents, *I*_Kr_ and *I*_Ks_, and the inward‐rectifier K^+^ current, *I*_K1_.^6^ The action potential plateau phase is especially vulnerable because the repolarizing and depolarizing currents are nearly balanced, and a small increase in the net inward current can cause prolongation of the action potential, inducing EADs. This type of EAD is believed to be the major trigger for torsades de pointes (TdPs)‐type polymorphic ventricular tachycardia associated with long QT syndromes. EADs may also contribute to arrhythmogenesis in the failing heart with electrical remodeling featuring action potential prolongation. Bradycardia and hypokalemia may facilitate this type of EAD by enhancing the prolongation of the action potential. EADs may occur during the repolarization phase of the action potential (phase 3 EADs) in cardiac myocytes with short action potentials (e.g., atrial and pulmonary vein myocardial cells). Phase 3 EADs are initiated by the Ca^2+^ transient during the action potential, which produces a large inward *I*_NCX_ at a negative membrane potential level during action potential repolarization.

DADs are oscillations in membrane potential after complete action potential repolarization under pathological conditions of Ca^2+^ overload^6^, ^7^ (Figure 1D). Increased intracellular Ca^2+^ content can initiate spontaneous diastolic Ca^2+^ release from the sarcoplasmic reticulum, which activates a transient inward current, *I*_ti_. The inward *I*_NCX_ is the major component of *I*_ti_. Factors inducing intracellular Ca^2+^ overload include catecholamines, digitalis, tachycardia, hypokalemia, and ischemia/reperfusion. Dysfunction of the ryanodine receptor Ca^2+^ release channels can also initiate spontaneous diastolic Ca^2+^ release and induce DADs, which contributes to arrhythmogenic mechanisms in catecholaminergic polymorphic ventricular tachycardia, heart failure and atrial fibrillation. Purkinje fibers are more susceptible to spontaneous diastolic Ca^2+^ release and DAD‐mediated triggered activity than ventricular cells.

#### 2.1.3 Reentry

A cardiac excitation wave may return to and re‐excite myocardial tissues that have been excited by the same excitation wave, and this phenomenon is referred to as reentry. Reentry can occur around a fixed anatomic obstacle, such as myocardial infarction scar or valve annulus, or through an anatomic circuit consisting of the normal AV conduction system and accessory pathways. Anatomic reentry is initiated by unidirectional conduction block and is maintained by the presence of an excitable gap between the reentrant wave front and its tail of refractoriness.

**Figure 2:**
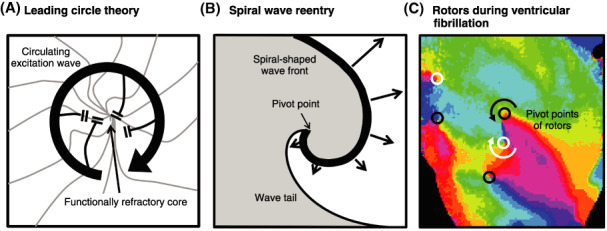
(**A**) Leading circle theory (Source: Prepared base on Allessie MA et al. 1977^9^). (**B**) Spiral wave reentry (Source: Prepared base on Pertsov AM et al. 1993^10^). (**C**) Multiple rotors during ventricular fibrillation. Pivot points can be identified by convergence of all phases of the action potential (Source: Prepared base on Harada M et al. 2008^11^).

Reentry can be established around an area of functional conduction block without anatomic obstacles.^8^ Two theories have been proposed to explain the mechanism of functional reentry: (1) leading circle theory^9^ and (2) spiral wave reentry concept.^8^ The leading circle model is characterized by a functionally refractory core and an excitation wave circulating around the inactive core without a fully excitable gap^9^ (Figure 2A). Spiral wave reentry is driven by an electrical rotor around a functional pivot point where a spiral‐shaped excitation wave front meets its own wave tail^9^ (Figure 2B). Stationary rotors in the ventricles provide an electrogram pattern of monomorphic tachycardia, whereas meandering rotors give rise to polymorphic tachycardia. Spiral wave breakup into multiple rotors may correspond to degeneration from tachycardia to fibrillation^9^, ^10^, ^11^ (Figure 2C). Myocardial tissues with a preserved long action potential plateau can re‐excite tissues with highly abbreviated action potentials when these sites have been recovered from refractoriness. This type of re‐excitation is referred to as phase 2 reentry and is thought to be a mechanism of ventricular tachycardia and fibrillation in Brugada syndrome and during acute myocardial ischemia.

### 2.2 Classification and Mechanism of Action of Antiarrhythmic Drugs

#### 2.2.1 Vaughan Williams Classification

The Vaughan Williams classification,^13^ which was publicized in 1969, classifies antiarrhythmic drugs into 4 classes. Originally, class I represented Na^+^ channel blockers, class II sympathetic *β*‐blockers, class III agents prolonging action potential duration (APD), and class IV Ca^2+^ channel blockers. Subsequently, class I was found to consist of drugs with various actions on ECG, and thus Harrison^14^ classified class I drugs into 3 subclasses. Class IA drugs prolong the PR interval, QRS duration, and QT interval, class IB drugs shorten the QT interval without effects on the PR interval nor QRS duration, and class IC drugs prolong the PR interval and QRS duration without effects on the QT interval (Table 5). These differential effects on ECG parameters are caused by the rate of their binding to and dissociation from the Na^+^ channels. Class IB drugs have the fastest rate of binding to and dissociation from the channels, and thus the drugs that bind to the channels during the systolic period are absent in the channels in the diastolic period, rendering no effects on the PR interval or QRS duration during sinus rhythm. Class IC drugs have the slowest rate of binding to and dissociation from the channels, and thus these drugs are still bound to the channels when the diastolic period is over, causing prolongation of the PR interval and QRS duration during sinus rhythm. Class IA drugs are intermediate between these 2 classes of drugs. It has been shown that APD prolongation by class III drugs is caused by K^+^ channel blockade.^14^ QT interval prolongation by class IA drugs is caused by APD prolongation, which is also attributed to K^+^ channel blockade.

**Table 5:** Vaughan Williams Classification

Class	Action	Representative drugs
Class I	Na^+^ channel blockade	
IA	PR interval/QRS duration: prolong intermediatelyQT interval: prolong	quinidine, procainamide, disopyramide, cibenzoline, pirmenol
IB	PR interval/QRS duration: no changeQT interval: shortening	lidocaine, mexiletine, aprindine
IC	PR interval/QRS duration: prolong stronglyQT interval: no change	propafenone, flecainide, pilsicainide
Class II	Adrenergic *β* receptor blocker	propranolol, metoprolol, bisoprolol, etc.
Class III	APD prolongation (K^+^ channel blockade)	amiodarone, sotalol, nifekalant
Class IV	Ca^2+^ channel blockade	verapamil, diltiazem, bepridil

Abbreviation: APD, action potential duration.

#### 2.2.2 Principle of Sicilian Gambit

Sudden death after myocardial infarction had been a serious social problem in Europe and the USA since the 1970s. Premature ventricular contractions (PVCs) are indicators of sudden death, so class I drugs were prescribed to suppress PVCs without verified evidence they reduced the risk of sudden death. In 1989, the Cardiac Arrhythmia Suppression Trial (CAST) was performed to validate the efficacy of this strategy and the result was shocking: class I drugs flecainide and encainide even increased the incidence of sudden death after myocardial infarction.^15^ The result of CAST provoked the claim that the Vaughan Williams classification did not precisely represent the actions of antiarrhythmic drugs, and the European Society of Cardiology (ESC) characterized the effects of antiarrhythmic drugs on electrophysiology and electrocardiogram as the Principle of the Sicilian Gambit.^16^, ^17^ It characterized the effects of 22 antiarrhythmic drugs on ion channels, receptors, ion pumps, clinical actions and ECG^18^, ^19^ (Table 6). It is worth noting that digitalis, adenosine triphosphate (ATP) and atropine that have been used against arrhythmias were newly classified as antiarrhythmic drugs in the Sicilian Gambit.

**Table 6:** Sicilian Gambit

Drugs	Ion channels						Receptor				Pumps	Clinical effects			ECG		
	Na^+^			Ca^2+^	K^+^	I_f_	*α*	*β*	M_2_	A_1_	Na^+^ ‐ K^+^ ATPase	LV function	Sinus rhythm	Extra‐heart	PR	QRS	JT
	Fast	Med	Slow														
lidocaine	〇											→	→	●			↓
mexiletine	〇											→	→	●			↓
procainamide		●A			●							↓	→	●	↑	↑	↑
disopyramide			●A		●				〇			↓	→	●	↑↓	↑	↑
quinidine		●A			●		〇		〇			→	↑	●	↑↓	↑	↑
propafenone		●A						●				↓	↓	〇	↑	↑	
aprindine		●I		〇	〇	〇						→	→	●	↑	↑	→
cibenzoline			●A	〇	●				〇			↓	→	〇	↑	↑	→
pirmenol			●A		●				〇			↓	↑	〇	↑	↑	↑→
flecainide			●A		〇							↓	→	〇	↑	↑	
pilsicainide			●A									↓→	→	〇	↑	↑	
bepridil	〇			●	●							?	↓	〇			↑
verapamil	〇			●			●					↓	↓	〇	↑		
diltiazem				●								↓	↓	〇	↑		
sotalol					●			●				↓	↓	〇	↑		↑
amiodarone	〇			〇	●		●	●				→	↓	●	↑		↑
nifekalant					●							→	→	〇			↑
nadolol								●				↓	↓	〇	↑		
propranolol	〇							●				↓	↓	〇	↑		
atropine									●			→	↑	●	↓		
ATP										■		?	↓	〇	↑		
digoxin									■		●	↑	↓	●	↑		↓

Relative magnitude of blockade: 〇 low, ● intermediate, ● high. Direction of clinical effects and ECG changes: ↑ increase, ↓ decrease, → no change. A, activated channel blocker; I, inactivated channel blocker. ■ blockade.

Abbreviations: ATP, adenosine triphosphate; ECG, electrocardiogram; LV, left ventricular. (Lifemedicom 2000.^18^)

#### 2.2.3 Mechanism Underlying the Actions of Antiarrhythmic Drugs

**Figure 3:**
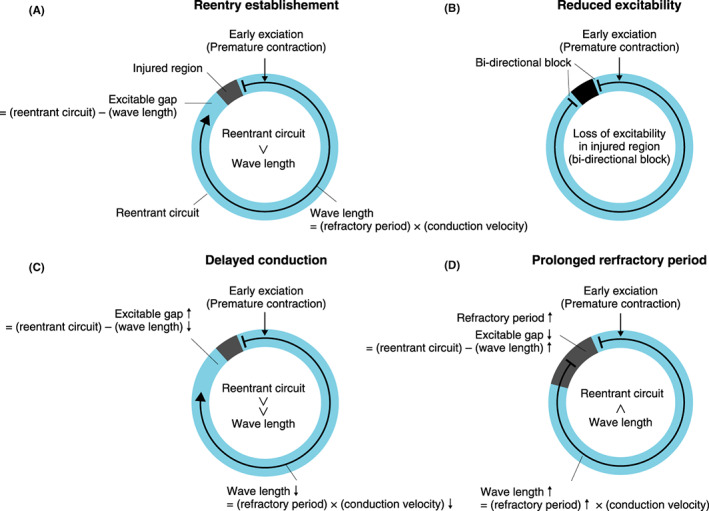
Scheme for reentrant arrhythmia and effects of antiarrhythmic drugs. The situation where early exciation (premature contraction) (vertical arrow) penetrates in the reentrant circuit (blue circle) in vicinity of damaged tissue (gray or black box). (**A**) Without anti‐arrhyhmic drugs. (**B**) Drugs that reduce excitability, such as class I drugs. (**C**) Drugs that delay conduction, such as class IC drugs. (**D**) Drugs that prolong refractory period, such as class III drugs.

The actions of antiarrhythmic drugs should be considered based on their effects on electrophysiological parameters, such as refractory period, conduction, and excitability of cells. These are labeled as “vulnerable factors”. Let’s discuss the actions of antiarrhythmic drugs against reentrant arrhythmia with a certain length of reentrant circuit, a part of which is injured and has decreased excitability (Figure 3A). Premature excitation (ex. premature contraction) arriving adjacent to injured tissue cannot excite the injured tissue, which has decreased excitability, but it can conduct to the other limb of the circuit. The product of refractory period and conduction velocity represents how far the excitation can travel through the circuit during the refractory period, and is referred as the wave length,

Wave length = refractory period x conduction velocity

The difference between the actual length of the reentrant circuit and wave length calculated as above is referred to as the excitable gap.

Excitable gap = (length of reentrant circuit) − (wave length)

If the excitable gap is positive, when excitation traveling in a retrograde direction arrives at the injured tissue, the injured tissue is already out of the refractory period and can excite (unidirectional block), and thus reentry is established. The larger the excitable gap is, the more likely that reentry occurs. Conversely, the smaller the excitable gap is, the less likely reentry occurs. Class I drugs block Na^+^ channels, and thus suppress excitation conduction especially intensely in the injured region. In the case of the injured region losing excitability completely, excitation traveling in a retrograde direction is unable to excite the injured region (bidirectional block) and reentry is not established (Figure 3B). In the case of insufficient blockade of the Na^+^ channels, the excitability of the injured region remains and bidirectional block is not established. In this case, the excitable gap becomes larger due to shortening of the wave length caused by reduced conduction velocity, so that reentry is more likely to occur (Figure 3C). Class III drugs prolong the refractory period and increase the wave length. As a result, the excitable gap becomes smaller and reentry is less likely to occur. As class IA drugs affect both conduction velocity and refractory period, they can show both antiarrhythmic and proarrhythmic effects depending on the magnitude of their effects on conduction velocity and refractory period.

#### 2.2.4 Recent Consensus

Although the Sicilian Gambit was created mainly by the ESC, its recent antiarrhythmia guideline does not mention it. The heart is composed of a wide variety of cells.^20^, ^21^, ^22^ The types and amount of expressed ion channels, receptors and ion pumps differ considerably in each cell; for example, they differ between the atrium and the ventricle, between apex and base of the ventricles, and between the endocardial side and the epicardial side. Thus, no matter how precisely one analyzes the actions of drugs on ion channels, receptors and ion pumps in a certain cell type, one cannot comprehensively dictate their effects on the electrophysiology of the whole heart and on clinical arrhythmias. Current medical practice is grounded in evidence‐based medicine. The Sicilian Gambit did have an important role in establishing evidence for antiarrhythmic drug treatment. Nowadays, a simpler classification of antiarrhythmic drugs is preferable, such as the Vaughan Williams classification, together with guideline treatment with antiarrhythmic drugs based on clinical evidence.

### 2.3 Pharmacokinetics (Absorption, Distribution, Metabolism, and Elimination) and Pharmacodynamics

#### 2.3.1 Pharmacokinetics

**Figure 4:**
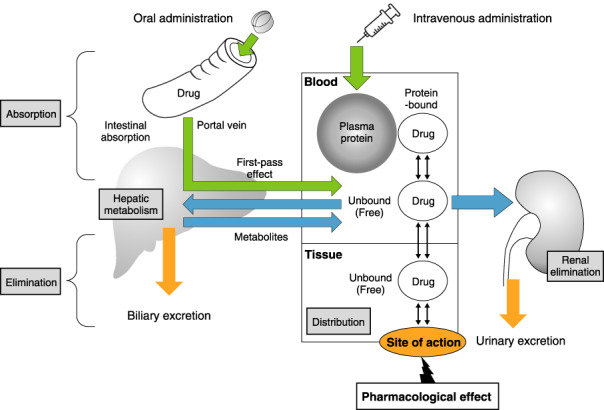
Pharmacokinetics (absorption, distribution, metabolism, and elimination).

Pharmacokinetics refers to how the body handles a drug, which involves absorption, distribution, metabolism and elimination (ADME). After being administered, a drug reaches its site of action, binds to its receptor and exerts its pharmacological effects (Figure 4). By contrast, pharmacodynamics refers to how a drug affects the body; that is, the relationship between a drug binding to its receptor and its pharmacological action. How a drug exerts its effects is determined by its pharmacokinetic and pharmacodynamic profiles.

#### 2.3.2 Absorption

Drugs administered extravascularly must pass through several barriers before entering the blood circulation. Orally administered drugs are absorbed through the gastrointestinal tract, enter the portal vein and then, via the liver, enter the circulating bloodstream. The process during which administered drugs enter the blood circulation is called absorption, and it is affected by the biophysical and chemical properties of the drugs. The fraction of the administered dose that reaches systemic circulation intact is defined as its bioavailability.

Multidrug‐resistance 1 gene product (P‐glycoprotein) is expressed in the small intestine, blood‐brain barrier, hepatocyte, and renal proximal tuble, and pumps drugs out of cells. Direct oral anticoagulants (DOACs) are partly excreted into the gastrointestinal tract via P‐glycoprotein; therefore, their bioavailability and plasma concentration are increased when they are coadministered with P‐glycoprotein inhibitors (verapamil, quinidine, amiodarone, azole antifungal drugs, HIV protease inhibitors, etc.). Special caution should be exerted when dabigatran or edoxaban is coadministered with verapamil, quinidine or amiodarone: their doses should be lowered. On the other hand, when P‐glycoprotein inducers (rifampicin, carbamazepine, etc.) are coadministered, the expected pharmacological effects of DOACs may not be obtained due to decreased bioavailability and plasma concentration (Table 7).

**Table 7:** Pharmacokinetic Profiles of DOACs and Warfarin (From Drug Interview Forms)

	dabigatran	rivaroxaban	apixaban	edoxaban	warfarin
Target	Thrombin	Xa	Xa	Xa	II, VII, IX, X
Bioavailability (%)	6.5	66–112	50	62	<99
Time to maximum concentration (t_max_) (h)	0.5–2	2–4	1–4	1–1.5	0.5
Transporter	P‐gp (GIT)	P‐gp (GIT)	P‐gp (GIT)	P‐gp (GIT)	P‐gp (Liver)
Protein binding rate (%)	35	92–95 (albumin)	87	40–59	97 (albumin)
Metabolism	Glucuronide conjugation	CYP3A4 / CYP2J2	CYP3A4	CYP3A4 (<10%)	S‐form: CYP2C9 R‐form: 1A2, 3A4
Renal excretion rate (%)	80	33	25	50	<1
Elimination half‐life (t_1/2_) (h)	12–14	9–13	8–15	6–11	55–133
Prodrug	〇	×	×	×	×

Abbreviations: CYP, cytochrome P450; DOAC, direct oral anticoagulant; GIT, gastrointestinal tract; P‐gp, P‐glycoprotein.

#### 2.3.3 Distribution

Following absorption, drugs distribute to a variety of organs and tissues. The volume of distribution (Vd) is the value obtained by dividing the total amount of the drug in the body by its plasma concentration. The Vd is calculated as the hypothetical value corresponding to the apparent volume in which the drug at the plasma concentration is evenly distributed. When the Vd is larger, more of the drug will be present in the extravascular tissue (i.e., it will more easily penetrate into the tissue from the blood). Among the antiarrhythmic drugs, digoxin and amiodarone have a large Vd. The Vd of digoxin is 8.4 L/kg, and it is mainly distributed to skeletal muscle, whereas the Vd of amiodarone is 106 L/kg, and it is mainly distributed to fat.

Drugs in the blood bind to plasma proteins, such as albumin and *α*‐acid glycoprotein. Only the drug in the unbound (free) form (unbound to the protein) can reach the site of action. Therefore, when the ratio between the protein‐bound and unbound (free) forms changes, the pharmacological effects of the drug can vary even if the total plasma concentration does not change. Especially in the case of high plasma protein‐bound drugs (>80%), their pharmacological effects may vary during hypoproteinemia and inflammation, which can affect their binding ratio to the plasma protein.

Drugs with high albumin binding, such as warfarin, will exert a stronger effect in hypoalbuminemia, which leads to an increase in the unbound (free) form of the drugs. By contrast, drugs with high *α*‐acid glycoprotein binding, such as lidocaine, disopyramide, propranolol and verapamil, will exert a weaker effect during inflammation, which leads to an increase in *α*‐acid glycoprotein, which in turn reduces the unbound (free) form of the drugs.

#### 2.3.4 Metabolism

Many drugs undergo metabolism in 2 phases (phase I and phase II) to become more hydrophilic and to be excreted in the urine. The phase I reaction involves oxidation, reduction and hydrolysis, in which liver cytochrome P450 (CYP) enzymes play important roles. The phase II reaction involves conjugation, which couples the drug to an endogenous molecule such as glucuronic acid, sulfuric acid or acetic acid. Each drug is metabolized differently. There are several CYP isoforms and different isoforms of CYP metabolize different types of drugs. Among the isoforms, CYP2D6 and CYP3A4 are mainly responsible for the metabolism of antiarrhythmic drugs^23^ (Table 8).^24^

**Table 8:** CYPs Involved in Metabolism of Cardiovascular Drugs: Major Substrates, Inhibitors, and Inducers

Isoforms	Substrates	Inhibitors	Inducers
CYP1A2	propranolol, mexiletine	mexiletine, fluvoxamine	smoking
CYP2C9	S‐warfarin	amiodarone, bucolome, benzbromarone, azole antifungal drugs, cimetidine	rifampicin, phenytoin, phenobarbital, carbamazepine, bosentan
CYP2D6	aprindine, flecainide, mexiletine, lidocaine, propafenone, bepridil, propranolol, metoprolol, carvedilol	amiodarone, quinidine, propafenone, paroxetine, cimetidine, duloxetine	
CYP3A4	dihydropyridine Ca^2+^ channel blockers, amiodarone, quinidine, disopyramide, lidocaine, bepridil, diltiazem, verapamil, rivaroxaban, apixaban, edoxaban	amiodarone, diltiazem, erythromycin, clarithromycin, azole antifungal drugs, cimetidine, grapefruit juice	rifampicin, phenytoin, phenobarbital, carbamazepine, bosentan

Modified from Guidelines for Therapeutic Drug Monitoring of Cardiovascular Drugs: Clinical Use of Blood Drug Concentration Monitoring (JCS 2015).^24^ The following changes were made: bucolome, benzbromarone, azole antifungal drugs and cimetidine were added as CYP2C9 inhibitors; bosentan was added as a CYP2C9/3A4 inducer; rivaroxaban, apixaban and edoxaban were added as CYP3A4 substrates.

Antiarrhythmic drugs are shown in red. CYP, cytochrome P450.

CYP2D6 deficiency is found in 5–10% of Caucasian individuals, but is rare in Japanese individuals (<1% of the population). However, approximately 40% of Japanese individuals have the mutant gene CYP2D6*10, with decreased enzyme activity.^25^ Additionally, CYP2D6 has a low enzymatic capacity, and its metabolic rate becomes constant at a relatively low substrate concentration (saturation). Therefore, drugs metabolized by CYP2D6 exhibit nonlinear pharmacokinetics, where the drug dose–plasma concentration is not proportional. Increasing the drug dose above certain level results in an increase in its plasma concentration larger than expected from linear pharmacokinetics. Examples of such drugs are aprindine, propafenone and bepridil (Table 8).^24^

Ca^2+^ channel blockers, amiodarone and DOACs such as rivaroxaban and apixaban are metabolized by CYP3A4. Although CYP3A4 deficiency has not been reported, its enzymatic activity varies greatly among individuals. Because diltiazem inhibits CYP3A4 activity, the plasma concentration of CYP3A4 substrate drugs may increase when coadministered with diltiazem (Table 8).^24^ By contrast, rifampicin, carbamazepine, phenobarbital, etc. induce CYP3A4. Therefore, when coadministered with the CYP3A4 substrate drug, they may inhibit the pharmacological effects of the drug by reducing its plasma concentration^23^ (Table 8).^24^

S‐warfarin is more potent than its enantiomer R‐warfarin and is metabolized mainly by CYP2C9. Nonsteroidal anti‐inflammatory drugs (NSAIDs), antifungal drugs, uricosuric drugs, amiodarone etc. inhibit CYP2C9, whereas rifampicin, carbamazepine, phenobarbital, bosentan, etc. induce CYP2C9. Therefore, the former drugs enhance and the latter drugs reduce the anticoagulant activity of S‐warfarin (Table 8).^24^

#### 2.3.5 Elimination

Antiarrhythmic drugs are primarily excreted from the kidney and the liver. Glomerular filtration and tubular secretion are involved in renal excretion. Glomerular filtration is a passive process that excretes free‐form drugs of small size. By contrast, tubular secretion is an active process that involves organic anion (negative ion) and cation (positive ion) transport systems. Drugs such as procainamide and pilsicainide are excreted into the urine via the organic cation transport system.^26^ Digoxin is excreted into urine by the transporter P‐glycoprotein. Because P‐glycoprotein is inhibited by quinidine, verapamil, amiodarone, etc., renal excretion of digoxin is decreased when these drugs are coadministered, which leads to increased digoxin plasma concentration.^23^, ^27^ Pilsicainide, sotalol, digoxin, cibenzoline, etc. are antiarrhythmic drugs that are excreted highly unchanged in the urine (renal excretion‐type).

#### 2.3.6 Special Conditions

##### 2.3.6.1 Renal Dysfunction

The dose of renally excreted drugs should be adjusted for patients with renal dysfunction. In particular, drugs that are excreted unchanged by more than 70% in the urine are strongly affected by renal dysfunction. Renal function is estimated by the Cockcroft‐Gault equation (mL/min) or by the glomerular filtration ratio (GFR) equation for Japanese individuals (mL/min/1.73 m^2^) of the Japanese Society of Kidney Disease.

**Figure 5:**
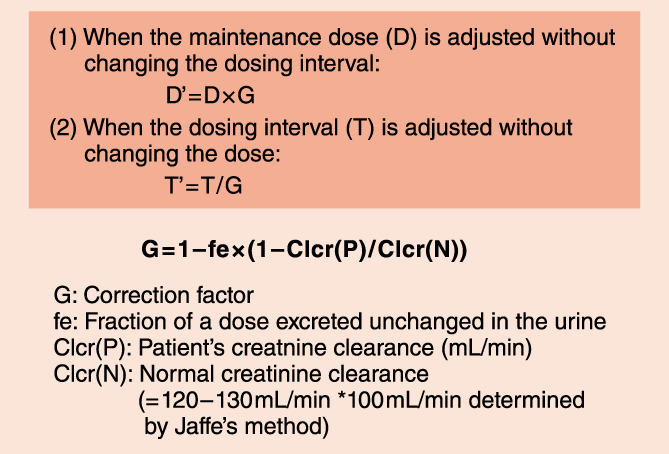
Dose adjustment for patients with renal dysfunction: the Giusti‐Hayton method.

The Giusti‐Hayton method is a simple method of adjusting the drug dose in patients with renal dysfunction^28^ (Figure 5). However, because this method yields an estimation of the initial dose, blood drug concentration monitoring should be performed at steady state during repeated administration.

##### 2.3.6.2 Liver Cirrhosis

Hepatic metabolism of drugs is affected by the severity of liver dysfunction and the metabolizing enzyme(s) involved. Liver metabolism by CYP2D6 is impaired in severe liver cirrhosis (Child‐Pugh class C),^29^ and liver metabolism by CYP3A4 is impaired in moderate and severe liver cirrhosis (Child‐Pugh classes B and C).^30^

##### 2.3.6.3 Children and Women During Pregnancy

Drug‐metabolizing enzymes and renal function are underdeveloped in neonates, and drug elimination capacity per tissue volume or weight is lower in infancy, until approximately 2 years of age, than in later childhood. On the other hand, the weight of drug‐eliminating organs (liver and kidney) per body weight is larger during childhood (until adolescence) than in adulthood; therefore, the dose proportionally calculated by weight is too small in children.

The embryo at weeks 3–9 of gestation is absolutely susceptible to the teratogenic effects of any chemical agents. In addition, pregnant women have an increase in plasma volume, a decrease in plasma protein concentration, an increase in the GFR and increased activity of drug‐metabolizing enzymes such as CYP2D6, all of which should be taken into consideration when drugs are administered.^31^

See **Chapters XI** (Arrhythmias in Pediatrics) and **XII** (Arrhythmias During Pregnancy) for details.

##### 2.3.6.4 Elderly Individuals

Because of the age‐related decline in physiological function, changes in pharmacokinetics and pharmacodynamics (susceptibility to drug effects) should be considered to ensure appropriate pharmacotherapy. Especially in elderly patients, because renal function is impaired, the clearance of renally excreted drugs decreases, and their elimination half‐life is prolonged.

#### 2.3.7 Blood Drug Concentration Monitoring

For antiarrhythmic drugs, therapeutic drug monitoring (TDM) is covered by the National Health Insurance (NHI) program in Japan because the therapeutic ranges are narrow. Clinicians should refer to the “Guidelines for Therapeutic Drug Monitoring of Cardiovascular Drugs: Clinical Use of Blood Drug Concentration Monitoring” (JCS 2015) for appropriate clinical usage and the interpretation of blood concentrations (Table 9).^24^

**Table 9:** Pharmacokinetic Parameters of Antiarrhythmic Drugs

Names	Volume of distribution (L/kg)	Protein binding rate (%)	Major route of excretion	Percentage of metabolites (%)	CYP enzymes mainly responsible for the metabolism	Fraction of dose excreted unchanged in the urine (%)	Half‐life (h)	Reference therapeutic range (*μ*g/mL)
amiodarone*^1,^*^2^	106	96	Liver	100	3A4, 2C8	<1	14–107 days*^4^	0.5–2 (?)
nifekalant	0.14	90	Liver	>90	Conjugation	28–31	1–2	–*^5^
lidocaine*^1,^*^2^	1–2	70	Liver	>95	3A4	<10	1–3	2–5
quinidine	3	80–90	Liver	70–90	3A4	20	6–8	2–5
aprindine*^2^	3	95–98	Liver	100	2D6	<1	1–2 days	0.25–1
propafenone*^1,^*^2^	3.7	75–88	Liver	>90	2D6	3	3–5	0.05–1 (?)
bepridil*^2^	8	99	Liver	>95	2D6	<1	80	0.2–0.8
mexiletine	5–12	70	Liver	>90	2D6, 1A2	6	10	0.5–2.0
disopyramide	0.6	20–75	Liver/kidney	40–50	3A4	48	5–9	2–5
flecainide	7–10	60	Liver/kidney	60	2D6	40	11–15	0.2–1
procainamide*^2^	1.7–2.4	15	Liver/kidney	40–50	NAT*^3^	60	2–3	4–10
pirmenol	1–1.5	80	Liver/kidney	35	3A4 (?)	20–30	7–10	>0.4 (?)
cibenzoline	7	70	Kidney	35	2D6	55–62	5–6	0.2–0.8
pilsicainide	1.5	35	Kidney	10	–	75–86	4–5	0.2–0.9
sotalol	1.2–2.4	10	Kidney	0	–	75	7–11	?*^6^

*^1^Producing active metabolites; *^2^nonlinear excretion; *^3^N‐acetyltransferase; *^4^∼13 days after a single administration; *^5^not determined in commercial laboratories; *^6^not established in Japanese (adults). TDM of all drugs except nifekalant is covered by the National Health Insurance (NHI) in Japan under the category of “specific therapeutic drug monitoring fees”.

(JCS and JSTDM 2017.^22^)

### 2.4 Side Effects of Antiarrhythmic Drugs and Countermeasures (Excluding Drug‐Induced Long QT Syndrome)

When using an antiarrhythmic drug, it is necessary to expect side effects and regular laboratory tests or imaging should be scheduled for the prevention of side effects. The main side effects of antiarrhythmic drugs are described (Table 10).

**Table 10:** Side Effects of Antiarrhythmic Drugs

Cardiac side effects		
Negative inotropic effect		Classes I, II, and IV
Proarrhythmic effect	Sudden death	Class IC increases sudden death in patients with old myocardial infarction
	Atrial flutter	Class IC converts atrial fibrillation to atrial flutter. Class I with anticholinergic effect (disopyramide or cibenzoline) induces atrial flutter with 1:1 conduction
	Brugada syndrome	Class I manifests Brugada syndrome and provokes ventricular fibrillation in Brugada syndrome patients
	Pacemaker failure	Class I
	Increase in the defibrillation threshold	Class I and high dose of amiodarone
	Decrease in the defibrillation threshold	Class III
	QT prolongation (TdP)	Classes IA and III
	Bradyarrhythmia	Classes II, III (amiodarone and sotalol) and IV
	Digitalis intoxication	Bradyarrhythmias and tachyarrhythmias
Extracardiac side effects		
	Benign prostatic hyperplasia	Urinary retention by class I with anticholinergic effects
	Angle‐closure glaucoma	Intraocular pressure elevation
	Bronchial asthma	adenosine triphosphate and nonselective *β*‐blockers
	Lower extremity edema	Class IV
	General malaise, sleep disorders, bronchial asthma, depressive tendency, and intermittent claudication	Class II
	Hypoglycemia	disopyramide, cibenzoline
	Thyroid dysfunction	amiodarone
	Pulmonary complications	amiodarone, bepridil
	Liver dysfunction	amiodarone
	Optic neuritis	amiodarone
	Photosensitivity	amiodarone
	Digestive symptoms	quinidine

Abbreviation: TdP, torsade de pointes.

#### 2.4.1 Heart Failure Due to Negative Inotropic Effect

Decreased Na^+^ influx due to the Na^+^ channel blocking effect of class I drugs causes an increase in Ca^2+^ efflux through the Na–Ca exchanger, resulting in decreased myocardial contractility. Therefore, class I drugs should not be used in heart failure patients.^32^ Class II drugs (*β*‐blockers) may decrease cardiac function and blood pressure, and induce bradycardia. Therefore, *β*‐blockers should be given in small doses and the dose adjusted by observing symptoms, blood pressure, chest X‐ray, and ECG. Class IV drugs (non‐dihydropyridine Ca^2+^ channel antagonists [verapamil and diltiazem]) should also be withheld in patients with cardiac dysfunction because they suppress the intracellular Ca^2+^ influx.

#### 2.4.2 Proarrhythmic Effect

An antiarrhythmic drug may exacerbate existing arrhythmias or provoke new arrhythmias, which is called a proarrhythmic effect. For example, class I drugs used for suppressing ventricular arrhythmias, increase the number of premature ventricular systoles, sustain ventricular tachycardia, and shorten the tachycardia cycle length. In the Cardiac Arrhythmia Suppression Trial (CAST) study, class IC drugs for the prevention of ventricular premature contractions increased the rate of sudden death compared with placebo.^15^, ^33^ The increased rate of sudden death is considered to be the result of the arrhythmogenic effect due to a decrease in conduction velocity with Na^+^ channel inhibition. Although class I drugs are often used for the termination and prevention of recurrence of atrial fibrillation, various arrhythmogenic effects may occur. The use of class IC drugs in patients with atrial fibrillation can cause it to change into atrial flutter because the excitation wavelength is prolonged due to lengthening of the refractory period.

The class I drugs with anticholinergic effects may cause atrial flutter with 1 : 1 conduction because of atrioventricular conduction enhancement. In this case, the heart rate is >300 beats/min and fatal, and thus sinus rhythm reversion or rate control must be promptly achieved. On the other hand, in atrial fibrillation associated with an accessory pathway syndrome, the use of digitalis, Ca^2+^ channel antagonists, and *β*‐blockers, which suppress atrioventricular conduction, facilitates conduction through the accessory pathway, thus increasing the ventricular rate. Not only does the tachycardia persist, but it may shift to ventricular fibrillation. The class I drugs may induce a Brugada‐type ECG or induce ventricular fibrillation in patients with Brugada syndrome. In patients with a cardiac pacemaker, pacing and sensing failure may occur because class I drugs reduce myocardial excitability. In addition, the class I drugs raise the defibrillation threshold, making electrical defibrillation difficult. In contrast, the class III drugs sotalol and nifekalant lower the defibrillation threshold.^34^

The class IA and class III drugs are at risk of causing TdP due to prolongation of the QT interval with K^+^ channel suppression. It is well known that QT interval prolongation is more prominent in female patients, as well as patients with hypokalemia, hypomagnesemia, and heart failure. Bradyarrhythmias such as sinus bradycardia, sinus arrest, sinoatrial block, and atrioventricular block may occur with class I, II, and IV drugs. In digitalis intoxication, both bradyarrhythmias (sinoatrial block and atrioventricular block) and tachyarrhythmias (atrial tachycardia with block, bidirectional ventricular tachycardia, etc.) can occur. Concomitant use of class II and class IV drugs should be avoided because of the excessive bradycardia.

#### 2.4.3 Extracardiac Side Effects

In benign prostatic hyperplasia, class I drugs with anticholinergic effects (quinidine, disopyramide, cibenzoline, pirmenol) may lead to urinary retention. In angle‐closure glaucoma, these drugs may cause a rapid increase in the intraocular pressure, resulting in optic nerve damage. Class I drugs should be avoided in myasthenia gravis because they may worsen symptoms. Adenosine triphosphate (ATP) used for terminating paroxysmal supraventricular tachycardia exacerbates bronchial asthma. Ca^2+^ channel blockers may induce lower extremity edema, and *β*‐blockers may cause general malaise, sleep disorders, bronchial asthma, a depressive tendency, and intermittent claudication. Disopyramide and cibenzoline may cause hypoglycemia in a dose‐dependent manner. Amiodarone has various extracardiac side effects, mainly thyroid dysfunction (hyperfunction and hypofunction), pulmonary complications (interstitial pneumonia, etc.), liver dysfunction, eye complications (optic neuritis), and dermatitis (photosensitivity). Of those, pulmonary complications are observed in approximately 3%,^35^ and the mortality rate is 5–10%.^36^ Pulmonary complications may occur within a few days after the initial administration, but in most cases, the risk increases over 12–60 months. Age, high maintenance doses, high blood levels of the active metabolite desethylamiodarone, and decreased lung diffusion before treatment are risk factors for this complication.^37^ Digoxin causes extracardiac side effects as the blood concentration increases. The main side effects are digestive symptoms (nausea, vomiting, loss of appetite, diarrhea, etc.) central nervous system symptoms (confusion, blurred vision, yellowing, weakness, fatigue, headache, etc.), gynecomastia, and thrombocytopenia.^38^

Blood tests for the early detection and follow‐up of side effects of antiarrhythmic drugs include liver and renal function tests, albumin, electrolytes (especially serum K^+^ and Ca^2+^), and B‐type natriuretic peptide (BNP) levels. The presence of structural disease and cardiac function should be checked by echocardiography. Physicians should be vigilant about monitoring the PR interval, RR interval, QRS width, and QT interval on the 12‐lead ECG. In addition to confirmation of the effects of antiarrhythmic drugs on the Holter ECG, the presence of sinus arrest, atrioventricular block, and new arrhythmias should be checked. When administering amiodarone, thyroid function and the KL‐6 and surfactant protein‐A and ‐D levels should be checked. Lung auscultation, chest X‐rays, and chest CT are helpful for the early detection of pulmonary complications. In patients with heart failure, unexpected adverse events may occur because decreased organ blood flow and glomerular filtration rate affect the pharmacokinetics. To avoid any side effects of class I drugs and amiodarone, therapeutic drug monitoring (TDM) is helpful.^31^ When digoxin is used in renal failure or dialysis patients, it is necessary to monitor the blood concentration frequently to adjust the dose or administration interval.

## 3 BRADYARRHYTHMIAS

The rhythm of the heart is regulated by self‐firing action potentials originating from the sinus node propagating through the specialized conduction system that includes the atrioventricular node (AVN), His‐bundle, right and left bundle branches, and Purkinje fibers. Dysfunction of the sinus node or AVN and the distal conduction system leads to the bradyarrhythmias called sick sinus syndrome (sinus node dysfunction) and atrioventricular block, respectively.

### 3.1 Clinical Presentation of Bradyarrhythmias

Bradyarrhythmias suddenly develop long pauses, which result in transient brain ischemia leading to dizziness, light headedness, and syncope (i.e., Adams‐Stokes attack). The diagnosis may be difficult when the symptoms are transient and infrequent. Patients with type III sick sinus syndrome often have preceding palpitation due to tachyarrhythmias, typically atrial fibrillation. Symptomatic heart failure can result from chronotropic incompetence with sinus node dysfunction or atrioventricular block. Some patients with bradyarrhythmias are asymptomatic.

### 3.2 General Principles of Management of Bradyarrhythmias

No treatment is required for individuals with asymptomatic bradycardia, such as athletes with sinus bradycardia and those with Wenckebach‐type 2nd‐degree atrioventricular block. Pacemaker implantation is indicated for patients with symptomatic bradyarrhythmias.

Bradyarrhythmias with a reversible cause due to drugs, hyperkalemia etc., should be treated to eliminate those influences, in combination with a temporary pacemaker if necessary. Right ventricular pacing using a transvenous lead is a standard technique for temporary pacing. In an emergency, percutaneous pacing using external patch electrodes is also performed. Pharmacological therapy, especially with intravenous drugs, can be a bridging method for temporary pacing as well as a permanent pacemaker implant. The patient’s preference and severity of illness may lead to selection of pharmacological therapy even if pacemaker implantation is preferable for managing the hemodynamic instability with bradyarrhythmias. Oral medications may be used in patients with infrequent or undetected bradycardia whose symptoms are mild.

### 3.3 Pharmacological Therapy for Bradyarrhythmias

The class of recommendation and level of evidence regarding pharmacological therapy for bradyarrhythmias are summarized in Table 11.^39^, ^40^, ^41^, ^42^, ^43^, ^44^, ^45^, ^46^, ^47^, ^48^

**Table 11:** Recommendations and Levels of Evidence for Pharmacological Therapy for Bradycardia Attributable to Sinus Node Dysfunction or Atrioventricular Block

	COR	LOE	GOR (MINDS)	LOE (MINDS)
Intravenous administration of sympathomimetics or atropine as a bridging method for pacemaker therapy	IIa	C	B	V
Oral administration of theophylline* or cilostazol* in patients with bradycardia attributable to sick sinus syndrome or atrioventricular block who refuse or are not eligible for pacemaker implantation^39^, ^40^, ^41^, ^42^, ^43^, ^44^	IIa	C	B	IVb
Intravenous administration of theophylline for atropine‐resistant atrioventricular block in the early phase of acute inferior myocardial infarction^45^, ^46^, ^47^, ^48^	IIb	C	B	V

*Use of theophylline and cilostazol for bradyarrhythmias cannot be reimbursed by healthcare insurance.

Abbreviations: COR, class of recommendation; GOR, grade of recommendation; LOE, level of evidence; MINDS, Medical Information Network Distribution Service.

#### 3.3.1 Atropine

Atropine is used for vagal bradycardia. The initial dose for intravenous administration of atropine is 0.5 mg, and it can be given repeatedly in the case of persistent bradycardia. Atropine activates the atrio‐Hisian or intranodal conduction of AVN, and is effective for AVN block (AH block: the site of the block is the AVN). Self‐firing of the sinus node is also activated by atropine, thereby increasing the rate of atrial contraction. With the increase, the ratio of atrioventricular conduction can deteriorate, such that the heart rate may be decreased in patients with HV (His bundle to ventricular) block (the site of the block is intra‐ or infra‐Hisian).

#### 3.3.2 Sympathomimetics

Intravenous administration of isoproterenol at a dose of 0.01–0.03 *μ*g/kg/min is used in emergency or as an alternative method of temporary pacing. Adrenaline (2–10 *μ*g/min) or dopamine (2–10 *μ*g/kg/min) is also recommended in the case of atropine resistance.^49^

#### 3.3.3 Theophylline

Intravenous or oral administration of theophylline is reported to be useful for symptomatic bradyarrhythmias attributable to sick sinus syndrome or atrioventricular block.^39^, ^40^, ^41^, ^42^ Theophylline inhibits phosphodiesterase activity and is a competitive blocker of adenosine receptors. Therefore, theophylline is expected to antagonize adenosine‐related bradyarrhythmias in which adenosine reduces the excitability of both the sinus node and AVN through activation of the acetylcholine/adenosine‐regulated K^+^ (*I*_K, ACh_) channel. Adenosine is an endogenous metabolite that accumulates in the interstitium during myocardial ischemia. In this regard, slow injection of aminophylline (theophylline with ethylenediamine: 150–300 mg over 15 min) reverses the atropine‐resistant complete atrioventricular block in the early phase of acute inferior myocardial infarction and restores sinus rhythm with 1 : 1 atrioventricular conduction (1st‐degree atrioventricular block).^45^, ^46^, ^47^, ^48^

A group of patients (mean age 55 ± 19 years) with recurrent syncope and idiopathic paroxysmal atrioventricular block has been characterized by low plasma adenosine level and high susceptibility to exogenous and endogenous adenosine.^50^ Oral theophylline is reported to be effective in these patients.^50^, ^51^, ^52^ The dose of theophylline is >600 mg daily in the USA and Europe,^39^, ^52^ but 200–400 mg daily in Japan.^42^ The cost of theophylline for bradyarrhythmias is not applicable for healthcare insurance reimbursement.

#### 3.3.4 Cilostazol

Cilostazol is a phosphodiesterase inhibitor that increases the level of intracellular cyclic AMP (cAMP), leading to vasodilation and inhibition of platelet aggregation. cAMP‐dependent activation of the L‐type Ca^2+^ channel current (*I*_Ca, L_) and pacemaker current (*I*_f_) in the sinus node contributes to an increase in the heart rate, hence cilostazol can have a positive chronotropic effect.

Oral daily administration of 200 mg of cilostazol in patients with sick sinus syndrome increased the mean heart rate from 54 beats/min to 79 beats/min and shortened the mean maximal RR interval from 2.98 s to 1.96 s.^43^ In patients with complete atrioventricular block and symptomatic heart failure, oral cilostazol 200 mg daily increases the rate of ventricular escape rhythm and reduces the level of B‐type natriuretic peptide (BNP).^44^ Cilostazol increases the heart rate of patients with bradycardiac atrial fibrillation.^53^, ^54^ Similar to theophylline, the cost of cilostazol is not applicable for healthcare insurance reimbursement.

### 3.4 PREMATURE CONTRACTIONS

#### 3.4.1 Supraventricular Premature Contractions

Supraventricular premature contractions (SVPCs) are defined as premature contractions that occur in the atrium or at atrioventricular junction. SVPCs occur even in healthy subjects and the incidence increases with aging. SVPCs do not cause hemodynamic disorders and have a good prognosis. Hoever, the relationship between SVPCs and future AF has been drawing attention lately.

#### 3.4.2 Pathology and Clinical Significance

More than 90% of healthy people have SVPCs, and most people have less than 100 beats/day.^55^ Up to about 100 beats/day of SVPCs can be considered normal.

SVPC may increase with caffeine, alcohol, stress, fatigue, chronic obstructive pulmonary disease (COPD), valvular heart disease, cardiomyopathy, etc. SVPCs have little effect on symptoms and hemodynamics, and rarely require treatment. However, SVPCs >100 beats/day is a predictor of new‐onset AF in general population without apparent organic heart disease.^56^

SVPCs detected during a health examination are associated with more cases of AF and cardiovascular death.^57^, ^58^ A meta‐analysis revealed that SVPCs are related with stroke, all‐cause death, cardiovascular disease, and coronary artery disease,^59^ but treatment of SVPCs is not recommended due to the low absolute risk, despite the significant hazard ratio.

Cerebral infarction is classified into cardiogenic cerebral embolism, lacunar infarction, atherothrombotic cerebral infarction, etc. depending on the cause. Strokes of unknown cause account for 20–25% of the whole and are called cryptogenic strokes. Most of them are thought to be embolic and are termed embolic stroke of unknown sources (ESUS). SVPCs are clinically important in cases of ESUS. The more SVPCs the patient with ESUS has, the more episodes of AF are detected during long‐term ECG monitoring.^60^, ^61^ For example, ≈40% of patients with ≥1,000 SVPCs/day develop new AF, which should be taken into consideration in determining the indication for anticoagulant therapy after cerebral infarction.^60^

#### 3.4.3 Pharmacological Treatment

Table 12 show the recommendations for lifestyle and pharmacological treatment, and the levels of evidence for treatment of SVPCs.^15^ It usually requires no treatment, but treatment is considered when SVPCs impair quality of life (QOL); however, the balance between safety and necessity should be considered carefully. It is important to educate patients about the low risk of SVPCs and lifestyle effects such as caffeine and alcohol intake. *β*‐blockers are sometimes recommended, especially for SVPCs that increase during the day.^62^ Class I antiarrhythmic drugs other than mexiletine (e.g., aprindine, cibenzoline, pilsicainide, propafenone and flecainide) can be used in patients without organic heart disease.

**Table 12:** Recommendations of Lifestyle and Pharmacological Treatment, and Levels of Evidence for the Treatment of SVPCs

	COR	LOE	GOR (MINDS)	LOE (MINDS)
Restriction of caffeine or alcohol intake	I	C	C1	VI
Use of *β*‐blockers for symptomatic SVPC patients	IIa	C	C1	V
Use of antiarrhythmic drugs for asymptomatic SVPC patients	IIb	C	C2	VI
Use of class I agents for SVPC patients with myocardial infarction^15^	III	B	D	II

Abbreviation: COR, class of recommendation; GOR, grade of recommendation; LOE, level of evidence; MINDS, Medical Information Network Distribution Service; SVPC, supraventricular premature contraction.

Class I antiarrhythmic drugs are not recommended for patients with prior myocardial infarction or cardiac dysfunction because they may worsen the prognosis.^15^

### 3.5 Premature Ventricular Contractions

Lifestyle changes and/or mild tranquillizers are sufficient to treat patients with mild symptoms of premature ventricular contractions (PVCs). However, PVCs are known to trigger severe arrhythmia,^63^ and cardiac function sometimes declines in patients with frequent PVCs.^64^ Therefore, risk assessment is important in patients with PVCs.

#### 3.5.1 Risk Assessment of Patients With Premature Ventricular Contractions

Evaluation of organic heart disease, cardiac function, type of arrhythmia (frequency of arrhythmia, timing of occurrence, presence/absence of couplets, triplets, and short runs, among other such parameters), and family history of hereditary arrhythmias is necessary to estimate the risk of ventricular tachycardia and sudden cardiac death. The Lown classification,^65^ which is based on the risk of onset of PVCs after myocardial infarction, was proposed before the era of currently available reperfusion treatment, but for practical purposes and convenience, it is applied to patients with PVCs under conditions other than myocardial infarction. The frequency of monomorphic PVCs is >30/h, and polymorphic, triplets, and short‐run, R‐on‐T type, and those with a short coupling interval are at high risk.

Reportedly, increased PVCs during exercise stress testing,^66^ and multiple episodes of PVCs during recovery after exercise are considered risk factors.^67^ Patients with ≥10,000 (≥10%) of PVCs/day, QRS width ≥150 ms,^68^, ^69^ and PVCs recorded throughout the day^70^ are at risk of PVC‐induced cardiomyopathy. However, the above factors of cardiomyopathy have low sensitivity and specificity. Reportedly, patients showing PVCs with short coupling intervals or high pulmonary wedge pressure concomitant with the PVCs are considered high‐risk patients.^71^, ^72^

#### 3.5.2 Idiopathic Premature Ventricular Contractions

PVCs without organic heart disease usually have a good prognosis. Antiarrhythmic drugs should not be administered to patients with minimal symptoms. Treatment with *β*‐blockers and Ca^2+^ channel antagonists is considered for patients with symptoms and frequent and/or multifocal PVCs;^73^, ^74^ however, the effect is limited.^75^, ^76^ Although amiodarone, sotalol, and some class I antiarrhythmic drugs effectively suppress arrhythmias, their proarrhythmic and other adverse effects should also be considered.

##### 3.5.2.1 Outflow Tract Premature Ventricular Contractions

The right ventricular outflow tract is the most common site of origin of PVCs, although they also originate in the left ventricular outflow tract and atrioventricular valve annulus. In most cases, the contributory mechanism includes triggered activity by intracellular Ca overload. *β*‐blockers, Ca^2+^ channel antagonists, and class I antiarrhythmic are recommended in patients with severe symptoms.^76^ Catheter ablation is considered in patients refractory to drug treatment.^4^

##### 3.5.2.2 Papillary Muscle Premature Ventricular Contractions

Notably, 5–10% of idiopathic PVCs originate in the anterior or posterior papillary muscles of the left ventricle, and are of the non‐reentrant type showing a right bundle branch block pattern. *β*‐blockers are used in such cases, depending on symptoms and frequency of the arrhythmia.

##### 3.5.2.3 Fascicular Premature Ventricular Contractions

This entity often shows a right bundle branch block and left axis deviation pattern. Reentry in Ca‐dependent tissue in the area supplied by the posterior branch of the left bundle branch or triggered activity from the Purkinje fibers is the likely contributory mechanism for this abnormality. Ca^2+^ channel antagonists, *β*‐blockers, and Na^+^ channel blockers (aprindine and mexiletine) are first choice.

### 3.6 Premature Ventricular Contractions Associated With Organic Heart Disease

Treatment is warranted for patients with severe subjective symptoms secondary to arrhythmia or in patients with several PVCs (≥10% of the total heart beats). Patients with highly frequent PVCs show improved cardiac function following a decrease in PVCs,^77^, ^78^ and *β*‐blockers, amiodarone, and mexiletine are preferred in such cases. Patients included in the Cardiac Arrhythmia Suppression Trial showed worse prognosis after the administration of class IC antiarrhythmic drugs following myocardial infarction.^33^ Consequently, class IC antiarrhythmic drugs are contraindicated in patients with organic heart disease or in those with diminished cardiac function. In principle, class IA antiarrhythmic drugs are also contraindicated in this patient population. The type and origin of arrhythmia, indicated by the coupling interval and the presence or absence of couplets, triplets, and short‐run PVCs should be considered for patient selection prior to implantable cardioverter‐defibrillator placement or ablation in those with diminished cardiac function and/or organic heart disease. Table 13 shows the recommendations and levels of evidence for drug therapy in patients with PVCs.^33^, ^73^, ^74^, ^79^, ^80^, ^81^

**Table 13:** Recommendations and Levels of Evidence for Drug Therapy in Patients With PVCs

	COR	LOE	GOR (MINDS)	LOE (MINDS)
Use of *β*‐blockers and calcium antagonists to improve QOL in patients with symptomatic PVCs without organic heart disease^73^, ^74^	IIa	B	B	II
Use of *β*‐blockers and amiodarone to improve symptoms and left ventricular function in patients with cardiomyopathy secondary to frequent PVCs^79^, ^80^, ^81^	IIa	B	B	III
Use of classes IA and IC antiarrhythmic drugs to patients with PVCs following myocardial infarction^33^	III	B	D	II

Abbreviations: Ca, calcium; COR, class of recommendation; GOR, grade of recommendation; LOE, level of evidence; MINDS, Medical Information Network Distribution Service; PVC, premature ventricular contraction; QOL, quality of life.

#### 3.6.1 Ischemic Heart Disease

The mechanisms of PVCs associated with ischemic heart disease include reentry, abnormal automaticity, and triggered activity.

##### 3.6.1.1 Acute Myocardial Infarction

*β*‐blockers are recommended for the prevention of ventricular arrhythmias in acute coronary syndromes, particularly in patients with ST‐elevation myocardial infarction.^82^ Prophylactic amiodarone or lidocaine administration is not recommended.^82^, ^83^ PVCs and accelerated idioventricular rhythm (AIVR) secondary to reperfusion injury may occur during treatment of coronary artery disease. Multiple PVCs and short runs may occur before the onset of ventricular fibrillation, and amiodarone, lidocaine, nifekalant, and procainamide are useful in these patients.

##### 3.6.1.2 Subacute and Chronic Myocardial Infarction

Amiodarone,^84^, ^85^ mexiletine, and sotalol are preferable in these patients.^86^, ^87^ Notably, classes IA and IC antiarrhythmic drugs are contraindicated.

#### 3.6.2 Patients With Diminished Cardiac Function

Patients with diminished cardiac function receive standard treatment for heart failure. Multiple PVCs are high risk. *β*‐blockers, amiodarone, and mexiletine are used in some patients. Reportedly, *β*‐blockers and amiodarone improve cardiac function in some patients with heart failure and concomitant frequent PVCs.^79^, ^80^, ^81^

## 4 PAROXYSMAL SUPRAVENTRICULAR TACHYCARDIA (ATRIOVENTRICULAR NODAL REENTRANT TACHYCARDIA, ATRIOVENTRICULAR RECIPROCATING TACHYCARDIA, ATYPICAL SUPRAVENTRICULAR TACHYCARDIAS)

### 4.1 Drugs for Differential Diagnosis of Narrow QRS Tachycardia

The recommendation and evidence level for differential diagnosis of narrow QRS tachycardia using a drug are shown in Table 14.^88^, ^89^

**Table 14:** Recommendation and Level of Evidence for Differential Diagnosis of Narrow QRS Tachycardia Using ATP

	COR	LOE	GOR (MINDS)	LOE (MINDS)
ATP* bolus i.v. to differentiate supraventricular tachycardia^88^, ^89^	I	C	C1	VI

* Not covered by medical insurance, contraindicated for asthmatic patients.

Abbreviations: ATP, adenosine triphosphate; COR, class of recommendation; GOR, grade of recommendation; LOE, level of evidence; MINDS, Medical Information Network Distribution Service.

Adenosine triphosphate (ATP) is one of the drugs that have been reported to inhibit atrioventricular nodular (AVN) conduction. It is effective for stopping narrow QRS tachycardia, as well as for making a differential diagnosis of atrial tachycardia and sinus tachycardia. A definitive differential diagnosis can be made between QRS and atrial or sinus tachycardia depending on whether the P wave remains constant after suppressing AVN conduction. If the tachycardia is controlled by drugs that suppress AVN conduction by the atrial wave (retrograde P wave), then orthodromic AVRT or AVN reentrant tachycardia (AVNRT) is suspected. Atrial tachycardia and other AVN‐dependent supraventricular tachycardias are among those that terminate in the ventricular wave (QRS wave); therefore, it is difficult to differentiate them by ECG waveform alone.

Intravenous ATP, verapamil, and *β*‐blockers all selectively delay conduction of the AVN. Because ATP has an extremely short half‐life, it is often used in making a differential diagnosis and stopping supraventricular arrhythmia, and is administered in a rapid intravenous injection.^88^, ^89^ Because ATP also has the effect of suppressing the sinus node, it should be cautiously administered to patients with sick sinus syndrome.

Group I antiarrhythmic agents suppress AVN conduction, but also act on accessory pathways in atrioventricular reciprocating tachycardia (AVRT). Furthermore, it is difficult to differentiate AVRT from ectopic atrial tachycardia by 12‐lead ECG alone, because it also suppresses ectopic automaticity. The start and maintenance of AVNRT are associated with the balance between the AVN refractory period and the conduction velocity, and drugs that affect such characteristics can affect the start or maintenance of the arrhythmia. To stop tachycardia, antiarrhythmic drugs of the class IA group (e.g., procainamide, quinidine, disopyramide)^90^, ^91^, ^92^, ^93^ or IC group (e.g., flecainide, propafenone)^94^ block retrograde fast pathway conduction. In constrast, atropine and isoproterenol facilitate retrograde fast pathway or orthodromic slow pathway conduction and the induction of AVNRT.^95^, ^96^, ^97^

Differential diagnoses for regular narrow QRS with long RP’ tachycardia and clear retrograde P wave (II, III, aVF, and negative P wave) observed with RP interval > PR interval include ectopic atrial tachycardia, uncommon (fast‐slow) AVNRT, and permanent junctional reciprocating tachycardia (PJRT).^98^ The latter two recur easily even if they are terminated and are often drug‐resistant. PJRT, as with orthodromic AVRT, is caused by a macrorentrant circuit.^99^, ^100^ However, the latent accessory pathway in this circuit is atypical; that is, it has conduction properties like the AVN, and has a long retrograde conduction time. This type of tachycardia responds to autonomic tone, catecholamines, *β*‐blockers, and digoxin.

### 4.2 Acute Treatment of Supraventricular Tachycardia

**Figure 6:**
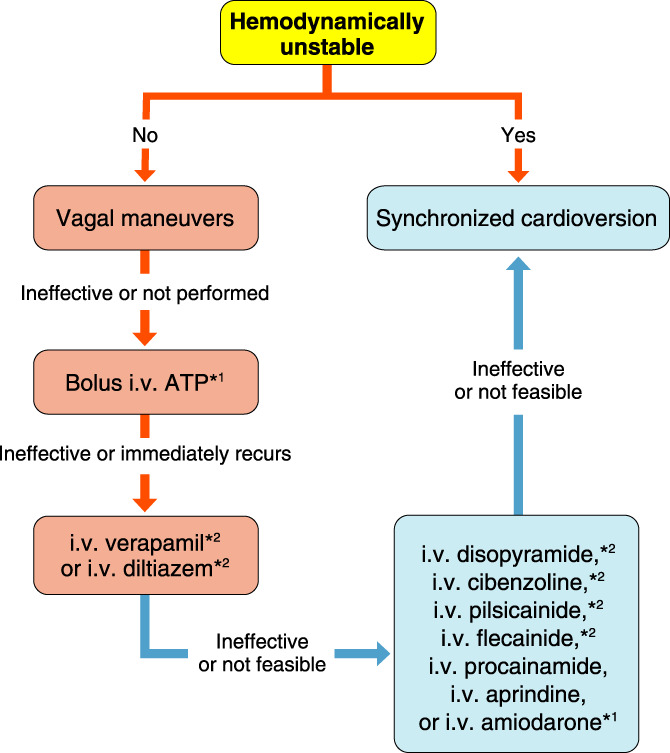
Acute treatment of narrow QRS supraventricular tachycardia. *^1^Off‐label use in Japan. *^2^Contralndicated in pts with reduced systolic function. ATP, adenosine triphosphate

Acute management of narrow QRS supraventricular tachycardia (SVT) begins with hemodynamic assessment. The majority of SVT patients exhibit an initial decrease in blood pressure, followed by a gradual recovery within 30 s.^101^ Although it is rare, sinus rhythm must be promptly restored by direct‐current synchronized cardioversion in patients with hemodynamic compromise (Figure 6, Table 15).

**Table 15:** Recommendations and Levels of Evidence for Acute Treatment of Narrow QRS SVT^*1^

	COR	LOE	GOR (MINDS)	LOE (MINDS)
Vagal maneuvers	I	B	B	II
Bolus i.v. ATP*^2^	I	A	A	I
Synchronized cardioversion for hemodynamically unstable SVT or drug‐refractory SVT	I	C	B	IVa
i.v. verapamil or diltiazem*^3,4^	IIa	A	B	I
i.v. procainamide, disopyramide,*^4^ cibenzoline,*^4^ aprindine, pilsicainide,*^4^ flecainide*^4^ or amiodarone*^2^	IIb	C	C1	V
Self‐administered single‐dose p.o. verapamil, diltiazem, and/or *β*‐blocker for infrequent, well‐tolerated SVT	IIb	C	C1	IVb

*^1^In patients with wide QRS SVT due to antidromic atrioventricular reciprocating tachycardia, i.v. ATP, verapamil, diltiazem or *β*‐blocker should be avoided, but i.v. class I antiarrhythmic drugs are recommended (class of recommendation IIa, level of evidence C, MINDS grade of recommendation C1, MINDS level of evidence V). *^2^Off‐label use in Japan. *^3^Class of recommendation of i.v. verapamil or diltiazem is IIb in patients with SVT who have ventricular pre‐excitation during sinus rhythm. *^4^Contraindication in patients with a reduced left ventricular function.

Abbreviations: ATP, adenosine triphosphate; COR, class of recommendation; GOR, grade of recommendation; LOE, level of evidence; MINDS, Medical Information Network Distribution Service; SVT, supraventricular tachycardia.

In hemodynamically stable patients, vagal maneuvers are the first‐line intervention to terminate SVT.^102^, ^103^, ^104^ There are several techniques used to increase vagal parasympathetic tone, including the Valsalva maneuver and carotid sinus massage. The Valsalva maneuver is performed by forceful attempted exhalation against a closed airway to raise intrathoracic pressure for 10–30 s. An increase in vagal tone occurs after releasing the Valsalva strain. It has recently been reported that leg lifting immediately at the end of the Valsalva strain enhances the vagal effect.^102^ Carotid massage is performed after the absence of bruit is confirmed by auscultation, by applying steady pressure over the right carotid sinus for 5–10 s. When right carotid massage is ineffective, left carotid massage is worth attempting. Because success in terminating SVT with vagal maneuvers depends on the physician’s skills and experience, pharmacological treatments are sometimes preferred as first‐line therapy.

If vagal maneuvers fail or are not attempted, an intravenous bolus injection of adenosine triphosphate (ATP) is the drug of choice (off‐label use in Japan).^105^, ^106^, ^107^ When administered intravenously, ATP is rapidly dephosphorylated into adenosine, which suppresses the atrioventricular node (AVN), resulting in termination of AVN‐dependent tachycardias such as AVN reentrant tachycardia (AVNRT), and atrioventricular reciprocating tachycardia (AVRT). The initial dose of ATP is 5–10 mg. If SVT persists, ATP administration can be repeated up to a maximum of 20 mg (high‐dose ATP should be used with caution because severe bradycardia occurs more frequently). Because the half‐life of ATP is extremely short (<10 s), a 10–20 mL flush of normal saline must follow the ATP injection when administered from a peripheral vein for ATP to reach the heart. Rapid intravenous ATP injection often results in unpleasant but transient side effects (flushing, chest discomfort, and headache, etc.). Thus, patients should be informed about such side effects before injection. ATP should be avoided in patients with bronchial asthma because of the bronchospastic effect of ATP.

When SVT does not respond to ATP or recurs immediately after termination with ATP, intravenous infusion of Ca^2+^ channel blocker (verapamil 5 mg or diltiazem 10 mg) given over 5 min is the next treatment of choice. Ca^2+^ channel blockers suppress the AVN similar to ATP but are longer acting than ATP. Success rates of ATP and Ca^2+^ channel blockers for SVT termination range from 80% to 95%.^105^, ^106^, ^108^ Ca^2+^ channel blockers should be avoided in patients with heart failure, because it is associated with a risk of hemodynamic deterioration.

When SVT does not respond to these treatments, AVN‐independent atrial tachycardia (AT) is the likely mechanism and intravenous class I antiarrhythmic drugs may be effective. Intravenous amiodarone can be considered in patients with a reduced left ventricular function (off‐label use in Japan).

If episodes of SVT are infrequent, a self‐administered single‐dose oral drug treatment might be useful. However, the usefulness of oral verapamil monotherapy has not been proven.^109^ The combination of oral diltiazem and propranolol appears effective in terminating AVN‐dependent tachycardias (e.g., AVNRT and AVRT), but the overall safety of these medications remains unclear because syncope has been reported as an adverse event.^110^

In patients with wide QRS SVT due to antidromic AVRT, AVN‐suppressing drugs should be avoided. Class I antiarrhythmic drugs such as procainamide or flecainide are the first treatment of choice for terminating antidromic AVRT.

### 4.3 Prophylactic Therapy

**Figure 7:**
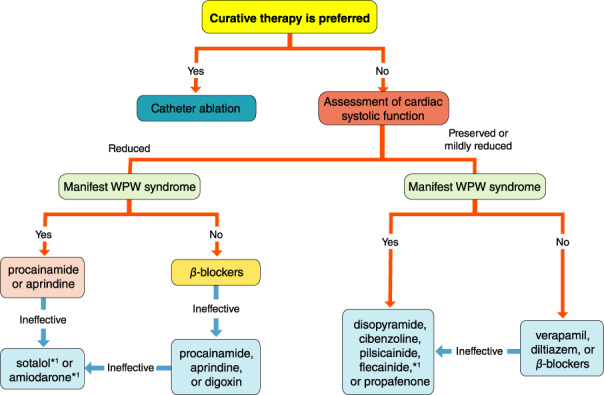
Prophylactic therapy of supraventricular tachycardia. *^1^Off‐label use in Japan. WPW, Wolff‐Parkinson‐White.

If the episodes of SVT are short and self‐limited, prophylactic therapy is not necessarily needed.^111^ When the SVT episodes are long enough to require acute management, catheter ablation is recommended as a first‐line prophylactic therapy that can provide a definitive cure with a high success rate (≥90%) and favorable safety profile.^112^, ^113^ Pharmacological treatments are indicated as prophylactic therapy in patients who are unwilling to undergo catheter ablation or have an unsuccessful catheter ablation (Figure 7, Table 16).

**Table 16:** Recommendations and Levels of Evidence for Prophylactic Therapy of Supraventricular Tachycardia

	COR	LOE	GOR (MINDS)	LOE (MINDS)
Catheter ablation	I	B	A	II
p.o. verapamil,*^1^ diltiazem*^1^ or *β*‐blockers in patients without manifest (including intermittent) WPW syndrome	I	A	A	I
p.o. flecainide*^1,2^ or propafenone*^1^	IIa	B	B	II
p.o. procainamide, disopyramide,*^1^ cibenzoline,*^1^ aprindine, pilsicainide*^1^	IIa	C	C1	V
p.o. sotalol*^3^	IIb	B	C1	II
p.o. amiodarone*^3^	IIb	C	C1	IVa
p.o. digoxin in patients without manifest WPW syndrome	IIb	B	C1	II

*^1^Contraindication in patients with reduced left ventricular function. *^2^Off‐label use for adults in Japan. *^3^Off‐label use in Japan.

COR, class of recommendation; GOR, grade of recommendation; LOE, level of evidence; MINDS, Medical Information Network Distribution Service; WPW, Wolff‐Parkinson‐White.

Oral verapamil, diltiazem, or *β*‐blockers reduce the frequency and duration of SVT episodes, and are the treatment of choice for pharmacological prophylactic therapy in the majority of patients with SVT who do not have ventricular pre‐excitation during sinus rhythm.^114^, ^115^, ^116^, ^117^ Patients with ventricular pre‐excitation may develop atrial fibrillation during SVT and be exposed to increased risk of ventricular fibrillation by accelerating conduction over the accessory pathway while receiving verapamil, diltiazem, or *β*‐blockers, so these drugs must be used with caution even when the ventricular pre‐excitation is intermittent. Also, oral verapamil and diltiazem should be avoided in patients with reduced left ventricular function because they can be harmful through their negative inotropic effect.

If oral verapamil, diltiazem, or *β*‐blockers are ineffective or contraindicated, oral class I antiarrhythmic drugs can be effective. A wide variety of class I antiarrhythmic drugs are available in Japan. The effectiveness and safety of oral flecainide or propafenone, which can be used in Western countries, have been well studied.^114^, ^118^, ^119^, ^120^ Other oral class I antiarrhythmic drugs, such as procainamide,^121^ disopyramide,^122^ cibenzoline,^123^ aprindine,^124^ and pilsicainide, appear to have similar effectiveness, although the evidence is limited. Disopyramide, cibenzoline, pilsicainide, flecainide, and propafenone have a negative inotropic effect and are contraindicated in patients with reduced left ventricular function.

Oral class III antiarrhythmic drugs, sotalol, and amiodarone, can be used even in patients with reduced left ventricular function, and have been shown to prevent episodes of SVT (off‐label use in Japan).^125^, ^126^ However, class III antiarrhythmic drugs have a risk of proarrhythmia or extracardiac side effects, and should be considered second‐line drugs.

Oral digoxin has a prophylactic effect on SVT, but a higher dose is required for that purpose.^117^ Some clinical studies showed higher digoxin levels were associated with worse clinical outcomes.^127^ Thus, caution is advised for the clinical use of digoxin. Digoxin is contraindicated in patients with ventricular pre‐excitation, but can be used in patients with reduced left ventricular function.

### 4.4 Atypical Supraventricular Tachycardias

#### 4.4.1 Reciprocating Tachycardias Using Mahaim Fibers

The most common form of this type of SVT is antidromic reciprocating tachycardias using atriofascicular or atrioventricular Mahaim fibers that have AVN‐like tissues in the proximal portion; therefore, intravenous ATP bolus is effective for acute treatment of the tachycardia.^128^, ^129^ As a prophylactic therapy, catheter ablation can be curative. Pharmacological prevention includes verapamil and *β*‐blockers despite the presence of ventricular pre‐excitation, because atriofascicular/ventricular fibers and the AVN have similar electrophysiologic properties.^130^

Nodo‐fascicular and nodo‐ventricular Mahaim fibers are rare accessory pathways that can be responsible for orthodromic or antidromic reciprocating tachycardias. Because the AVN is involved in SVT using nodo‐fascicular/ventricular fibers, intravenous injection of ATP or verapamil is useful for terminating the tachycardia.^131^ Oral verapamil or *β*‐blockers appears effective as prophylaxis.

#### 4.4.2 Junctional Tachycardia (Non‐Reentrant)

Intravenous *β*‐blockers are modestly effective in terminating and/or reducing the incidence of non‐reentrant junctional tachycardia.^132^ Intravenous verapamil or procainamide is also reasonable for the acute treatment of junctional tachycardia.^133^ Oral *β*‐blockers or verapamil can be used as prophylaxis.^133^, ^134^ Oral flecainide^113^, ^135^ or propafenone^136^ is also effective as a prophylactic treatment, but contraindicated in patients with reduced left ventricular function. Oral amiodarone can be considered in patients who are refractory to these treatments.^137^ Radiofrequency ablation has been performed as a potentially curative therapy; however, the risk of atrioventricular block is relatively high (5–10%). Cryoablation could be an alternative to radiofrequency ablation for safety reasons (so far, inadvertent atrioventricular block has never been reported in patients who underwent cryoablation for junctional tachycardia).^138^, ^139^, ^140^

## 5 ATRIAL FIBRILLATION

### 5.1 Epidemiology, Pathophysiology and Electrophysiological Mechanism of Atrial Fibrillation

#### 5.1.1 Epidemiology of Atrial Fibrillation

##### 5.1.1.1 Prevalence and Associated Risk Factors for Atrial Fibrillation

AF is the most common arrhythmia encountered in clinical practice and its prevalence increases with age. AF is also associated with the risk of developing some adverse cardiovascular outcomes, including stroke, myocardial infarction, heart failure, and death. Therefore, the burden imposed on the medical system by AF is expected to increase with the increase in the population aged ≥65 years. Therefore, epidemiological information such as the prevalence of AF and related factors is important.

The prevalence of AF depends not only on age, but also on conditions such as sex, race, hypertension, heart failure, coronary artery disease, valvular heart disease, obesity, diabetes, chronic kidney disease, and socioeconomic factors.^141^, ^142^, ^143^, ^144^, ^145^ The prevalence of AF is increasing year by year. A community‐based Framingham Heart Study reported that the prevalence of age‐adjusted AF has quadrupled (i.e., in men from 20.4 to 96.2 per 1,000 person‐years and in women from 13.7 to 49.4 per 1,000 person‐years)^146^ However, because the increased prevalence of AF may be due to an increased rate of risk factors, including older age, as well as improved detection methods for asymptomatic AF, interpretation of data requires caution.^146^ In Japan, an epidemiological study was conducted by the Japanese Circulation Society,^147^ and the results of periodical health checkups in 2003 (630,138 people aged ≥40 years who underwent health checkups) showed that the prevalence of AF increased with age in both men and women. The percentage increased to 3.44% for men and 1.12% for women in their 70 s; 4.43% for men and 2.19% for women over 80 years old. When this result is applied to the Japanese population and calculated, it is estimated that 716,000 people in Japan had AF as of 2005.^147^ When calculated using future population projections, it is predicted that by 2050 there will be approximately 1.03 million AF patients, accounting for approximately 1.1% of the total population.

##### 5.1.1.2 Modifiable Clinical Risk Factors Associated With Atrial Fibrillation

Addressing modifiable clinical risk factors may reduce the long‐term risk of developing AF or delay the onset of disease. Therefore, identification, prevention, and treatment of such conditions are important to prevent the development of AF. However, it should be noted that it is unclear how the treatment and removal of a single modifiable risk factor reduces the risk of AF (Table 17).

**Table 17:** Recommendation and Level of Evidence for Assessing Risk of AF

	COR	LOE	GOR (MINDS)	LOE (MINDS)
Risk assessments such as age, sex, hypertension, heart failure, coronary artery disease, valvular heart disease, diabetes, obesity, sleep‐disordered breathing, uric acid, smoking, alcohol consumption, risk score, and genetic predisposition	IIa	B	B	IVa

Abbreviation: AF, atrial fibrillation; COR, class of recommendation; GOR, grade of recommendation; LOE, level of evidence; MINDS, Medical Information Network Distribution Service.

###### 5.1.1.2.1 Hypertension

Hypertension is the most widely accepted risk factor for AF.^141^, ^142^, ^143^, ^144^, ^145^ It is reported that 56.5% of AF cases had ≥1 risk factors, of which hypertension was the most important factor.^142^, ^148^ It has also been reported that differences in long‐term patterns, such as persistently elevated systolic blood pressure and a longer history of antihypertensive treatment, are associated with an increased risk of developing AF.^149^ An urban cohort study of cerebral and cardiovascular disease among Japanese reported that the hazard ratio of systolic blood pressure in AF was further increased by being overweight.^150^

###### 5.1.1.2.2 Diabetes

An increase in fasting glucose of 18 mg/dL increased the risk of developing AF by 33% in a test population with impaired glucose tolerance.^151^ However, no clear association between AF and diabetes has been suggested.^146^, ^151^, ^152^ In addition, intensive glycemic control has not been shown to improve the new incidence of AF.^153^

###### 5.1.1.2.3 Obesity

Reports have been increasing in recent years suggesting a link between obesity and AF. The number of obese patients tend to increase,^146^ and an increase in body mass index (BMI) is associated with AF.^152^ Importantly, among the modifiable risk factors, obesity is the most prominent risk factor affecting the lifetime risk of AF.^146^, ^152^, ^154^ In addition, a study that examined the relationship between changes in BMI over 10 years and AF showed that decreased BMI decreased AF risk and increased BMI increased AF risk in both men and women.^155^

###### 5.1.1.2.4 Sleep‐Disordered Breathing

An increasing number of reports suggest a relationship between sleep‐disordered breathing and AF risk. Meta‐analysis results suggest that sleep‐disordered breathing may increase the risk of AF, and the higher the severity, the higher the risk of AF.^156^, ^157^

###### 5.1.1.2.5 Uric Acid

A limited number of studies have examined the relationship between uric acid and AF. In a cross‐sectional study of 325,771 patients undergoing regular physical examinations at a single health center in Japan, serum uric acid levels were independent of other cardiovascular risk factors for AF in both men and women. It was significantly associated with prevalence.^158^ Besides, the result of a retrospective study of 49,292 Japanese diagnosed with hypertension, diabetes, dyslipidemia, chronic kidney disease, and hyperuricemia or gout by a regular medical examination in a single hospital from January 2004 to June 2010 but without receiving uric acid‐lowering drugs, showed that hyperuricemia was an independent risk factor for AF.^159^

###### 5.1.1.2.6 Smoking

Smoking is a known risk factor for AF.^142^, ^152^, ^160^ It has also been reported that smokers with AF are at increased risk of hospitalization and death.^145^ A nearly 2‐year prospective study investigating the association between AF and smoking and total tobacco consumption in hospitalized patients in a single hospital in Japan found that current smokers and smokers with a Brinkman index ≥800, were independently associated with new onset of AF. However, in current smokers, the Brinkman index does not differ in hazard ratio, suggesting the importance of stopping smoking to prevent AF.^161^

###### 5.1.1.2.7 Alcohol Consumption

Meta‐analysis results indicate that AF risk increases with increasing alcohol consumption.^162^, ^163^ It has been reported that the risk of developing AF increases by 5% and the left atrium expands by 0.16 mm for every 10 g of daily alcohol intake (equivalent to a full glass).^164^

##### 5.1.1.3 Lifetime Risk of Atrial Fibrillation

It has been reported that the lifetime risk of AF is significantly increased even if only 1 clinical risk factor is present.^152^ The CHARGE‐AF score for AF risk assessment has been developed from research focusing on multiple cohorts, including age, race, height, weight, systolic and diastolic blood pressures, current smoking, treatment of hypertension, diabetes, and diabetes features.^144^ This score has subsequently been shown to be useful in predicting AF risk in multiple cohorts.^165^ In Japan, a 10‐year risk score for AF has been created using traditional risk factors that can be easily obtained by ordinary outpatient clinics, but the results of external verification of risk scores using other cohorts are awaited.^148^

The lifetime risk of AF varies significantly with an increased genetic predisposition in addition to clinical risk factors.^160^, ^166^, ^167^ A community‐based Framingham study has reported the long‐term potential for AF considering the CHARGE‐AF score consisting of clinical risk factors, and the genetic predisposition estimated by a genetic risk score generated from approximately 1,000 AF‐related single nucleotide polymorphisms.^160^ It was suggested that the lifetime risk of AF in individuals with a high genetic risk score was sufficiently high even with a low CHARGE‐AF score. Atrial fibrillation was also presumed to occur earlier in individuals with high CHARGE‐AF scores than in individuals with low CHARGE‐AF scores, regardless of genetic risk score.^160^

#### 5.1.2 Pathophysiology of Atrial Fibrillation

**Figure 8:**
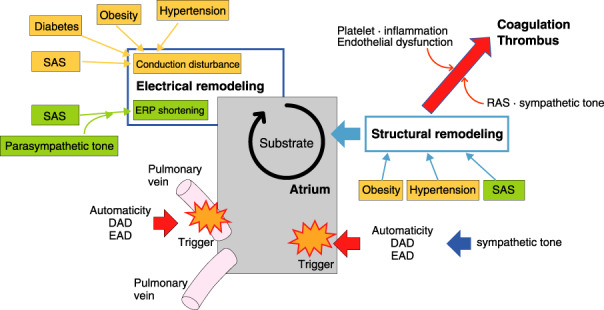
Risk factors contributing to both atrial remodeling and formation of thrombus. DAD, delayed afterdepolarization; EAD, early afterdepolarization; ERP, effective refractory period; RAS, renin–angiotensin system; SAS, sleep apnea syndrome.

The generation of both abnormal excitation (automaticity/auto‐excitation) as a trigger for premature beats and formation of a substrate constituting reentry circuit of AF in the atrium and the pulmonary vein, play a pivotal role for onset and maintenance of AF. Risk factors and AF itself form the substrate. Electrical remodeling alters the ion channels expression, causing conduction disturbance and shortened refractory periods, whereas structural remodeling induced by atrial fibrosis impairs the conduction disturbance. Besides, atrial remodeling (ATR) enhances coagulation to form thrombus. As shown in Figure 8, hypertension, obesity, diabetes and obstructive sleep apnea are identified as risk factors for atrial remodeling.^168^

##### 5.1.2.1 Structural Remodeling of Atrial Muscles

Either external stress, induced by structural heart disease, hypertension, diabetes, and inflammatory disease, or AF itself cause structural remodeling of the atrium. Pathophysiological changes responsible for ATR are characterized by activation of fibroblasts, increased connective tissue, fibrosis etc.^169^, ^170^, ^171^ In addition, atrial fat infiltration, inflammatory cell infiltration, hypertrophy, and necrosis, as well as deposition of amyloid are also observed. The structural remodeling disturbs electrical coupling among cardiac muscle fibers and causes heterogeneity of local electrical conductions, which facilitates formation of a reentry circuit.^172^

##### 5.1.2.2 Atrial Remodeling and Formation of Thrombus

ATR reduces the contractility of atrial muscles in association with enlargement of the atrium, which facilitates blood congestion in the atrium, thus accelerating thrombus formation. Activation of both the renin–angiotensin system^173^, ^174^, ^175^, ^176^ and the autonomic nervous system^177^, ^178^, ^179^ contribute to vascular remodeling, which induces atrial muscle ischemia, leading to ATR. On the other hand, ATR activates the expression of thrombus formation factors on the atrial endothelium together with activation of platelets and inflammatory cells, which contribute to thrombus formation.^180^, ^181^ These intra‐atrial activations and circulating coagulation factors could explain the onset of stroke following a short episode of AF.

#### 5.1.3 Electrophysiological Mechanism of Atrial Fibrillation

Both the electrical remodeling responsible for the arrhythmogenic substrate and the abnormal automaticity responsible for the trigger underly the electrophysiological mechanism of AF.

##### 5.1.3.1 Impaired Automaticity Forming the Trigger

The trigger for reentry arrythmia is defined as early ectopic excitation derived from a source different from the sinus node. Approximately 90% of ectopic excitation originates from the myocardial sleeve localized in the pulmonary vein. Some ectopic excitation derives from ectopic triggers in the area of either the superior vena cava or atrium as well. Underlying mechanisms on trigger are as described.

###### 5.1.3.1.1 Impaired Ca^2+^ Handling and Ectopic Excitation

Impaired regulation of the intracellular Ca^2+^ concentration induces Ca^2+^ leakage from the sarcoplasmic reticulum (SR) during the diastolic phase of cardiac myocytes, which plays a pivotal role in the generation of the trigger responsible for AF. In AF, the Ca^2+^ handling proteins have been hyperphosphorylated, which elevates the intracellular Ca^2+^ concentration and induces spontaneous Ca^2+^ release from the SR. This increases the inward currents on the cell membrane via activation of the Na^+^/Ca^2+^ exchanger causes the delayed afterdepolarization^182^, ^183^, ^184^, ^185^, ^186^ associated with triggered activity that facilitate ectopic excitation and leads to AF.^187^ Furthermore, activation of sympathetic nervous system augments the elevation of the intracellular Ca^2+^ concentration, facilitating abnormal automaticity and triggered activity.^188^

###### 5.1.3.1.2 Local Onset and Sustainability of Atrial Fibrillation

Hassaguerre et al^189^ demonstrated that automaticity excitation induced by triggered activity in the pulmonary vein becomes the trigger for AF, and the boundary between the pulmonary vein and atrial muscles forms the reentry circuit, which results in sustained AF.^190^, ^191^ This hierarchy of trigger and substrate in AF is observed in patients with paroxysmal AF,^192^, ^193^ although it remains unclear whether this hierarchy occurs in patients with sustained AF.^194^

###### 5.1.3.1.3 Multiple Wavelet Hypothesis and Rotors as the Source of Atrial Fibrillation

Moe and Abildskov^179^ demonstrated that AF could be sustained via multiple independent wavelets, which are conducted in a disorderly way through the muscular layer of the atrium. Many experimental and clinical observations support the multiple wavelet hypothesis.^195^ However, localized excitation (i.e., ectopic excitation, rotor and other stable reentry circuits) conducting to atrial fibrillation resembels to the spiral wave derived from multiple wavelet, so it is impossible to distinguish it from sustained AF caused by multiple wavelets.

##### 5.1.3.2 Electrical Remodeling of the Atrium Responsible for the Arrhythmogenic Substrate

The transition from paroxysmal AF to sustained AF is defined by the concept of “AF begets AF”.^196^ This concept encompasses the shortening of both the atrial refractory period and cycle length.^196^, ^197^ Although the number of electrical reentry circuits determines the sustainment of AF, the shorter the wave length of the reentry circuit becomes, the larger the number of reentry circuits, contributing to sustainability of AF (wave length hypothesis).^198^ The underlying mechanism is as follows. First, repeated excitation physiologically shortens the effective refectory period via the inducing of intracellular Ca^2+^ overloading, which is followed by progressive pathological shortening of the atrial refractory period through both downregulation of Ca^2+^ channel current and upregulation of inward‐rectifier K^+^ channel currents.^199^, ^200^ As well, the decreases in the expression of Na^+^ channel current and *I*_to_ currents results in both shortening of the refractory period and a reduction in conduction velocity. On the other hand, activation of parasympathetic nerve tone shortens the atrial action potential duration via activation of the acetylcholine‐sensitive K^+^ channel, which facilitates the formation of a reentry circuit in the atrium.^198^

### 5.2 Basic Strategy for Diagnosis and Management

#### 5.2.1 Classification of Atrial Fibrillation

**Figure 9:**
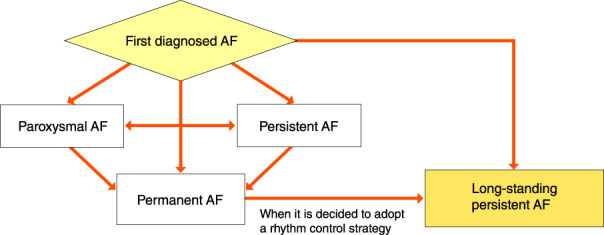
Classification of AF. AF, atrial fibrillation. (Source: Prepared based on Fuster V, et al. 2011,^**202**^ Fuster V, et al. 2006.^203^).

In general, AF starts as the paroxysmal form. The duration and frequency of the episodes increase with time, until AF becomes persistent and permanent. AF can be classified as 1 of 5 forms as described in Figure 9, ^202^, ^203^ and Table 18.^202^, ^203^

**Table 18:** Classification of AF

Disease type	Definition
First diagnosed AF	AF that is first documented on ECG
Paroxysmal AF	Self‐terminating AF May continue for up to 7 days
Persistent AF	AF that lasts longer than 7 days
Long‐standing persistent AF	Continuous AF lasting for ≥1 year when it is decided to adopt a rhythm control strategy
Permanent AF	AF that is accepted by the patient (and physician)

Abbreviation: AF, atrial fibrillation; ECG, electrocardiogram.

(Based on Fuster V, et al. 2011,^202^ Fuster V, et al. 2006.^203^)

##### 5.2.1.1 First‐Diagnosed Atrial Fibrillation

AF that has not been diagnosed before. According to the clinical course, first‐diagnosed AF is classified as paroxysmal, persistent, long‐standing persistent or permanent. If first‐diagnosed AF terminates spontaneously, AF will not recur for several years in ≈50% of patients. In CARAF,^204^ 899 first‐diagnosed AF patients were observed during a mean period of 4.1 years. Within 1 year, AF recurred in ≈50% of the patients. During the follow‐up period, 6–7% of the patients developed cerebral infarction.^204^

##### 5.2.1.2 Paroxysmal Atrial Fibrillation

Paroxysmal AF is AF that is self‐terminating, in most cases, within 48 h. Some AF paroxysms may continue for up to 7 days. In a Japanese study, during a mean follow‐up period of 14.1 years, paroxysmal AF eventually transited to the permanent form under conventional antiarrhythmic therapy (5.5% of patients per year).^205^

##### 5.2.1.3 Persistent Atrial Fibrillation

AF that lasts longer than 7 days. The actuarial cumulative percentages of patients who maintained sinus rhythm after serial cardioversion treatment was reported to be 42% and 27% after 1 and 4 years, respectively.^206^

##### 5.2.1.4 Long‐Standing Persistent Atrial Fibrillation

Continuous AF lasting for ≥1 year, when it is decided to adopt a rhythm control strategy by cardioversion and/or catheter ablation.

##### 5.2.1.5 Permanent Atrial Fibrillation

AF that is accepted by the patient (and physician).

#### 5.2.2 Symptom Burden in Atrial Fibrillation, Cryptogenic Stroke and Embolic Strokes of Undetermined Source

##### 5.2.2.1 Scoring of Symptoms

The recommendation for the evaluation method of AF based on symptoms is given in Table 19. The modified European Heart Rhythm Association (EHRA) divides the original class 2 into mild (2a) or moderate (2b) effect^207^ (Table 20). Class 2b identifies patients with a health utility benefit of rhythm control; this modification may provide a threshold for potential treatment decisions. The Canadian Cardiovascular Society Severity in Atrial Fibrillation (CCS‐SAF) scale is also available.^208^

**Table 19:** Recommendation and Level of Evidence of Evaluation Method of Atrial Fibrillation Based on Symptoms

	COR	LOE	GOR (MINDS)	LOE (MINDS)
Use of modified EHRA scale	IIa	C	B	IVa

Abbreviation: COR, class of recommendation; EHRA, European Heart Rhythm Association; GOR, grade of recommendation; LOE, level of evidence; MINDS, Medical Information Network Distribution Service.

**Table 20:** Modified EHRA Scale

Modified EHRA score	Symptoms	Description
1	None	
2a	Mild	Normal daily activity not affected by symptoms related to AF
2b	Moderate	Normal daily activity not affected by symptoms related to AF, but patient is troubled by symptoms
3	Severe	Normal daily activity affected by symptoms related to AF
4	Disabling	Normal daily activity discontinued

Underlined text indicates the modified portion from the original European Heart Rhythm Association (EHRA) scale.

Abbreviation: AF, atrial fibrillation. (Adapted from Wynn GJ, et al. 2014.^207^)

##### 5.2.2.2 Asymptomatic Atrial Fibrillation

In a Japanese single‐center study,^206^ first‐diagnosed AF patients without structural heart disease (n=289) were reviewed with regard to 2 symptom classifications (CCS‐SAF and EHRA). In both classifications, asymptomatic patients comprised ≈40% of the patients. The Fushimi AF registry^207^ investigated the clinical characteristics and outcomes of asymptomatic versus symptomatic patients with paroxysmal AF (n=1,837) or persistent/permanent (sustained AF; n=1,912) subgroups. Multivariable analysis indicated that age (≥75 years), history of stroke/systemic embolism, male sex, and chronic kidney disease were independent determinants of asymptomatic status in the paroxysmal AF group, whereas age was nonsignificant in the sustained AF group. During the follow‐up period, all‐cause death was significantly higher in the asymptomatic group compared with symptomatic patients in the paroxysmal AF group, whereas it was comparable in the sustained AF group.^210^

##### 5.2.2.3 Cryptogenic Stroke and Embolic Strokes of Undetermined Source

The term “cryptogenic stroke” refers to a stroke for which there is no specific attributable cause after a comprehensive evaluation.^211^ There is persuasive evidence that most cryptogenic strokes are thromboembolic. The thrombus is thought to originate from any of several well‐established potential embolic sources, including minor‐risk or covert cardiac sources, veins via paradoxical embolism, and non‐occlusive atherosclerotic plaques in the aortic arch or the cervical, or cerebral arteries. Accordingly, the term “embolic strokes of undetermined source” (ESUS) was proposed for a therapeutically relevant entity, which is defined as a non‐lacunar brain infarct without proximal arterial stenosis or cardioembolic source.^212^ The diagnostic criteria for ESUS are described in Table 21. Undiagnosed AF is considered to be one of the causes of ESUS. NAVIGATE ESUS tested the hypothesis that anticoagulant treatment with rivaroxaban, an oral factor Xa inhibitor, may result in a lower risk of recurrent stroke than aspirin.^213^ A total of 7,213 participants were enrolled at 459 sites: 3,609 patients were randomly assigned to receive rivaroxaban and 3,604 to receive aspirin. Recurrence of ischemic or hemorrhagic stroke or systemic embolism was not different between the 2 groups. However, major bleeding occurred in 62 patients in the rivaroxaban group (annualized rate, 1.8%) and in 23 in the aspirin group (annualized rate, 0.7%) (P<0.001). It was concluded that rivaroxaban was not superior to aspirin with regard to the prevention of recurrent stroke after an initial ESUS and was associated with a higher risk of bleeding.^213^ The reason why rivaroxaban could not show superiority to aspirin remains unclear. Recent improvement in diagnostic technology for undiagnosed AF might lead to enrollment of fewer patients in whom stroke was caused by AF. In RE‐SPECT ESUS, among patients with a recent history of ESUS, dabigatran was not superior to aspirin in preventing recurrent stroke. The incidence of major bleeding was not greater in the dabigatran group than in the aspirin group, but there were more clinically relevant nonmajor bleeding events in the dabigatran group.^214^

**Table 21:** Diagnostic Criteria for ESUS

Stroke detected by CT or MRI that is not lacuna^†^
Absence of extracranial or intracranial atherosclerosis causing ≥50% luminal stenosis in arteries supplying the area of ischemia
No major‐risk cardioembolic source of embolism^‡^
No other specific cause of stroke identified (e.g., arteritis, dissection, migraine/vasospasm, drug misuse)

Abbreviation: CT, computed tomography; ESUS, Embolic Strokes of Undetermined Source; MRI, magnetic resonance imaging.

(Adapted from Hart RG, et al. 2014.^212^)

#### 5.2.3 Detection of Atrial Fibrillation

##### 5.2.3.1 Pulse Check and Electrocardiogram

The recommendations and levels of evidence for detecting AF are described in Table 22. Pulse check is the simplest method. The Japanese Stroke Association (JSA) and the JHRS have annually determined March 9th as “A day for pulse”, and March 9–15th as “A week of AF”. On their website (http://www.shinbousaidou‐week.org/), JSA and JHRS emphasize the importance of pulse check.

**Table 22:** Recommendations and Levels of Evidence for Screening for AF

	COR	LOE	GOR (MINDS)	LOE (MINDS)
Periodic screening for AF is recommended by pulse check or ECG recording in patients aged >65 years	I	A	A	I
In patients with a history of TIA or ischemic stroke, screening for AF is recommended by short‐term ECG recording followed by continuous ECG monitoring for at least 72 h	I	B	B	IVa
ECG screening for AF may be considered in patients aged >75 years, or those at high stroke risk	IIa	B	B	IVa
In patients with cryptogenic stroke, long‐term noninvasive ECG monitors or implanted loop recorders should be considered to document silent AF	IIa	B	B	II
Interrogation of pacemakers and ICDs is recommended on a regular basis for AHRE. Patients with AHRE should undergo further ECG monitoring to document AF before initiating AF therapy	I	B	B	IVa

Abbreviation: AF, atrial fibrillation; AHRE, atrial high rate episodes; COR, class of recommendation; ECG, electrocardiogram; GOR, grade of recommendation; ICD, implantable cardioverter‐defibrillator; LOE, level of evidence; MINDS, Medical Information Network Distribution Service; TIA, transient ischemic attack.

##### 5.2.3.2 Monitor Electrocardiogram, Holter Electrocardiogram, Event Recorder or Insertable Cardiac Monitor

**Figure 10:**
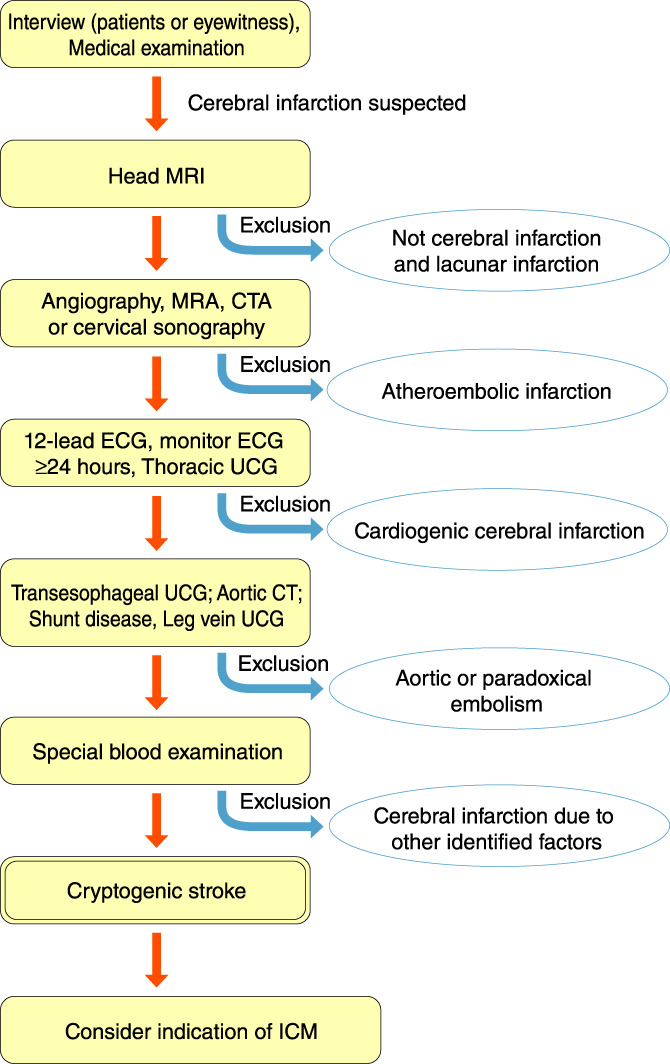
Flow chart for indication of ICM in patients with cryptogenic stroke. CT, computed tomography; CTA, computed tomography angiography; ECG, electrocardiogram; ICM, insertable cardiac monitor; MRA, mineralocorticoid‐receptor antagonist; MRI, magnetic resonance imaging; UCG, ultrasonic echocardiography. (Adapted from the Japan Stroke Society. 2016.^218^)

In patients experiencing a cerebral infarction, the presence of AF is occasionally observed on 12‐lead ECG or monitor ECG after admission. EMBRACE^215^ randomly assigned 572 patients aged 55 years or older, without known AF, who had had a cryptogenic ischemic stroke or TIA within the previous 6 months (cause undetermined after standard tests, including 24‐h ECG), to undergo additional noninvasive ambulatory ECG monitoring with either a 30‐day event‐triggered recorder (intervention group) or a conventional 24‐h monitor (control group). Within 90 days of randomization, AF lasting ≥30 s was detected in 45 of 280 patients (16.1%) in the intervention group, as compared with 9 of 277 (3.2%) in the control group (P<0.001). CRYSTAL AF^216^ was a randomized, controlled study of 441 patients to assess whether long‐term monitoring with an insertable cardiac monitor (ICM) was more effective than conventional follow‐up (control) for detecting AF in patients with cryptogenic stroke. By 6 months, AF had been detected in 8.9% of patients in the ICM group (19 patients) versus 1.4% of patients in the control group (3 patients) (P<0.001). By 12 months, AF had been detected in 12.4% of patients in the ICM group (29 patients) versus 2.0% of patients in the control group (4 patients) (P<0.001). Sposato et al^217^ reported studies that provided the number of patients with ischemic stroke or TIA who were newly diagnosed with AF. They stratified the cardiac monitoring methods into 4 sequential phases of screening: phase 1 (emergency room) consisted of admission ECG; phase 2 (in hospital) comprised serial ECG, continuous inpatient ECG monitoring, continuous inpatient cardiac telemetry, and in‐hospital Holter monitoring; phase 3 (first ambulatory period) consisted of ambulatory Holter ECG; and phase 4 (second ambulatory period) consisted of mobile cardiac outpatient telemetry, external loop recording, and implantable loop recording. The summary proportions of patients diagnosed with post‐stroke AF were 7.7% in phase 1, 5.1% in phase 2, 10.7% in phase 3, and 16.9% in phase 4. The overall AF detection yield after all phases of sequential cardiac monitoring was 23.7%.^217^ The AF detection rate increases in proportion to the ECG monitoring period. However, some researchers question whether AF detected 1–2 years after cerebral infarction was really involved in the development of cerebral infarction. From 2016, ICM to detect AF in patients with cryptogenic stroke has been covered by insurance in Japan.^218^ Figure 10 is a flowchart for the indication of ICM to detect AF in patients with cryptogenic stroke.

##### 5.2.3.3 Device‐Implanted Patients

In patients with an implanted pacemaker or ICD, atrial high rate episodes (AHRE) can be detected by analysis of intracardiac ECG recordings. Most cases of AHRE can be considered to be AF. ASSERT^219^ enrolled 2,580 patients, aged ≥65 years, with hypertension and no history of AF, in whom a pacemaker or defibrillator had recently been implanted. The patients were monitored for 3 months to detect subclinical atrial tachyarrhythmias (episodes of atrial rate >190 beats/min for >6 min) and followed them for a mean of 2.5 years for the primary outcome of ischemic stroke or systemic embolism. By 3 months, subclinical atrial tachyarrhythmias detected by implanted devices had occurred in 261 patients (10.1%), and were associated with an increased risk of clinical AF (P<0.001) and of ischemic stroke or systemic embolism (P=0.007). In ASSERT, subclinical AF episodes varied in duration. In a subanalysis of ASSERT,^220^ among 2,455 patients during a mean follow‐up of 2.5 years, the longest single episode of subclinical AF lasted >6 min to 6 h in 462 patients (18.8%), >6–24 h in 169 (6.9%), and >24 h in 262 (10.7%). Subclinical AF duration >24 h was associated with a significant increased risk of subsequent stroke or systemic embolism (P=0.003). The risk of ischemic stroke or systemic embolism in patients with subclinical AF between 6 min and 24 h was not significantly different from that of patients without subclinical AF.

#### 5.2.4 Integrated Management of Patients With Atrial Fibrillation

##### 5.2.4.1 Diagnosis and Evaluation of Patients With Atrial Fibrillation

The documentation of AF on 12‐lead ECG recording is required for the diagnosis of AF. The recommendations and levels of evidence for diagnosis and evaluation of patients with AF is described in Table 23.

**Table 23:** Recommendations and Levels of Evidence for Diagnosis of AF

	COR	LOE	GOR (MINDS)	LOE (MINDS)
ECG documentation is required to establish the diagnosis of AF	I	C	A	VI
Full cardiovascular evaluation, including an accurate history, careful clinical examination, and assessment of concomitant conditions, is recommended for all AF patients	I	C	A	VI
Transthoracic echocardiography is recommended for all AF patients to guide management	I	C	A	VI
Long‐term ECG monitoring should be considered in selected patients to assess the adequacy of rate control in symptomatic patients and to relate symptoms with AF episodes	IIa	C	B	VI

Abbreviation: AF, atrial fibrillation; ECG, electrocardiogram.

##### 5.2.4.2 Cardiovascular Morbidity and Mortality Associated With Atrial Fibrillation

Cardiovascular morbidity and mortality associated with AF are described in Table 24.^21^ Death due to stroke can largely be mitigated by anticoagulation, but other cardiovascular deaths (e.g., due to heart failure or sudden death) remain common even in AF patients treated according to the current evidence base. AF is also associated with increased morbidity, such as heart failure and stroke. Contemporary studies show that 20–30% of patients with an ischemic stroke have had AF diagnosed before, during, or after the initial event. White matter lesions in the brain, cognitive impairment, decreased quality of life (QOL), and depressed mood are common in AF patients, and between 10% and 40% of AF patients are hospitalized each year. Although most published studies have demonstrated associations between AF and impaired cognition, no AF treatment has yet been associated with a reduced incidence of cognitive decline or dementia.^221^

**Table 24:** Cardiovascular Morbidity and Mortality Associated With AF

Event	Association with AF
Death	Increased mortality, especially cardiovascular mortality due to sudden death, heart failure or stroke
Stroke	20–30% of all strokes are due to AF. A growing number of patients with stroke are diagnosed with ’silent’, paroxysmal AF
Hospitalization	10–40% of AF patients are hospitalized every year
Quality of life	Quality of life is impaired in AF patients independent of other cardiovascular conditions
Left ventricular dysfunction and heart failure	Left ventricular dysfunction is found in 20–30% of all AF patients. AF causes or aggravates LV dysfunction in many AF patients, while others have completely preserved LV function despite long‐standing AF
Cognitive decline and vascular dementia	Cognitive decline and vascular dementia can develop even in anticoagulated AF patients. Brain white matter lesions are more common in AF patients than in patients without AF

Abbreviation: AF, atrial fibrillation; LV, left ventricular. (Modified from Kirchhof P, et al. 2016.^21^)

##### 5.2.4.3 Five‐Step Management of Patients With Atrial Fibrillation

This guideline proposes a 5‐step plan for acute and chronic management of patients with AF (Table 25).^21^ Firstly, if the patient presents with hemodynamic instability or and/or severe symptoms, urgent management is required, including cardioversion. Secondly, precipitating factors, including unfavorable lifestyle and underlying cardiovascular diseases should be managed. Thirdly, following assessment of thromboembolic risk, oral anticoagulants should be administered to patients at risk of thromboembolism. Fourthly, in patients with persistent and permanent AF, rate control <110 beats/min should be considered. Fifthly, following assessment of symptoms, the indication of a rhythm control strategy should be considered. In this regard, there are 4 options: antiarrhythmic drugs, cardioversion, catheter ablation and surgery. Through steps 1–4, improved life expectancy can be obtained. Through steps 1–5, improved QOL, autonomy and social functioning can be expected. Regarding step 5, recent studies have reported that improved life expectancy can be obtained by catheter ablation. In CASTLE‐AF,^232^ catheter ablation for AF in patients with heart failure was associated with a significantly lower rate of a composite endpoint of all‐cause death or hospitalization for worsening heart failure than was medical therapy. In CABANA,^233^ among patients with AF, the strategy of catheter ablation, compared with medical therapy, did not significantly reduce the primary composite endpoint of death, disabling stroke, serious bleeding, or cardiac arrest. However, the estimated treatment effect of catheter ablation was affected by lower‐than‐expected event rates and treatment crossovers, which should be considered in interpreting the results of the trial.^223^ In CABANA,^224^ catheter ablation, compared with medical therapy, led to clinically important and significant improvements in QOL at 12 months.

**Table 25:** The 5‐Step Plan for Acute and Chronic Management of Patients With First‐Diagnosed AF

Step	Contents	Objectives	Benefit for patients
1. Acute rate and rhythm control	Emergency cardioversion, acute rate control	Hemodynamic stability	Improved QOL, autonomy, social functioning Improved life expectancy
2. Manage precipitating factors	Improvement of lifestyle, treatment of underlying cardiovascular diseases	Cardiovascular risk reduction	Improved QOL, autonomy, social functioning Improved life expectancy
3. Assess stroke risk	Oral anticoagulation in patients at risk for stroke	Stroke prevention	Improved QOL, autonomy, social functioning Improved life expectancy
4. Assess heart rate	Rate control therapy	Symptom improvement, preservation of LV function	Improved QOL, autonomy, social functioning Improved life expectancy
5. Assess symptoms	Antiarrhythmic drugs, cardioversion, catheter ablation, AF surgery	Symptom improvement	Improved QOL, autonomy, social functioning Improved life expectancy (Catheter ablation in AF patients associated with heart failure)

Abbreviation: AF, atrial fibrillation; LV, left ventricular; QOL, quality of life.

##### 5.2.4.4 Causes of Death in Patients With Atrial Fibrillation

GARFIELD‐AF^225^ was an observational study of adults with first‐diagnosed non‐valvular AF. The 2‐year outcomes of 17,162 patients prospectively enrolled in GARFIELD‐AF were analyzed. The mean age was 69.8 years, 43.8% were women, and the mean CHA_2_DS_2_‐VASc score was 3.3; 60.8% of patients were prescribed anticoagulant therapy with/without antiplatelet therapy, 27.4% antiplatelet monotherapy, and 11.8% no antithrombotic therapy. At 2‐year follow‐up, all‐cause death, stroke/systemic embolism, and major bleeding had occurred at a rate (95% confidence interval) of 3.83 (3.62; 4.05), 1.25 (1.13; 1.38), and 0.70 (0.62; 0.81) per 100 person‐years, respectively. Rates for all 3 major events were highest during the first 4 months. Congestive heart failure, acute coronary syndromes, sudden/unwitnessed death, malignancy, respiratory failure, and infection/sepsis accounted for 65% of all known causes of death, and strokes for <10%. Anticoagulant treatment was associated with a 35% lower risk of death. In Japan, in a subanalysis of the Fushimi AF Registry,^226^ during a median follow‐up of 1,105 days, there were 705 all‐cause deaths (5.5%/year); 180 cardiovascular (CV) (26% of total deaths), 381 non‐CV (54%), and 144 undetermined causes (20%). The most common causes of CV and non‐CV death were heart failure (14.5%), malignancy (23.1%), and infection/sepsis (17.3%), while mortality due to stroke was only 6.5%. In the multivariate analysis, the strongest indicator of CV death was pre‐existing heart failure and that of non‐CV death was anemia. Taken together, it is suggested that a more comprehensive approach to the management of non‐valvular AF may be needed to improve outcomes.

##### 5.2.4.5 Atrial Fibrillation and Cognitive Function

The expert consensus statement of the EHRA, HRS, Asia Pacific HRS, and the Latin American HRS summarizes the consensus of the international writing group and is based on a thorough review of the medical literature regarding cognitive function in arrhythmias.^227^ Evidence suggests that AF is associated with a higher risk for cognitive impairment and dementia, with or without a history of clinical stroke. Some of the reported brain morphometric changes include: hippocampal atrophy, white matter hyperintensities, and frontal medial lobe atrophy.^227^ Because the precise mechanism(s) of cognitive disorders in patients with AF is not fully known, the optimal way of preventing cognitive dysfunction for a given patient remains to be established. The expert consensus statement^227^ described the recommendations “may do this” for measures to prevent cognitive dysfunction in AF patients, as follows. (1) Appropriate anticoagulation in patients with AF and stroke risk factors should be applied for the prevention of cognitive dysfunction. (2) Consider DOACs instead of vitamin K antagonists (VKA) when using oral anticoagulation for the prevention of stroke in AF, which may have a beneficial effect on subsequent cognitive disorders. (3) In patients with AF managed with long‐term VKA, a high anticoagulation time in therapeutic range may be beneficial for optimal prevention of new‐onset dementia. (4) General health measures (prevention of smoking, hypertension, obesity and diabetes, sleep apnea, and appropriate control of all risk factors) may reduce the concomitant risks of AF (new onset or recurrence) and stroke, with a putative benefit on cognitive function. (5) Prevention of cognitive dysfunction in AF may include general measures proposed in vascular dementia or Alzheimer’s disease. (6) Cognitive assessment should be performed in AF patients whenever there is suspicion of cognitive impairment.

#### 5.2.5 Management of Risk Factors and Comorbidity

##### 5.2.5.1 Heart Failure

Heart failure (HF) and AF coincide in many patients. In the Japanese Guidelines for Diagnosis and Treatment of Acute and Chronic Heart Failure,^228^ the management of AF is described as the initial part of comorbidity. In AF‐CHF,^229^ a total of 1,376 congestive HF patients were enrolled (682 in the rhythm‐control group and 694 in the rate‐control group) and were followed for a mean of 37 months. There was no significant difference between the 2 groups with respect to death from cardiovascular cause, death from any cause, stroke, or worsening HF. It was concluded that for patients with AF and congestive HF, a routine strategy of rhythm control did not reduce the rate of death from cardiovascular causes, as compared with a rate‐control strategy.Recently, however, catheter ablation for AF, as a rhythm‐control strategy, has shown remarkable improvements in efficacy and safety. In CASTLE‐AF,^222^ catheter ablation for AF in patients with HF was associated with a significantly lower rate of a composite endpoint of all‐cause death or hospitalization for worsening HF than was medical therapy. In response to the results of CASTLE‐AF, in the 2018 JCS/JHRS Guideline on Non‐Pharmacotherapy of Cardiac Arrhythmias^4^ catheter ablation in AF patients with HF (LV dysfunction) was recommended as class IIa. In patients with HF with preserved or mid‐range ejection fraction (HFpEF, HFmrEF), AF was associated with similarly increased risk of death, HF hospitalization, and stroke or TIA.^230^, ^231^ Recently, catheter ablation of AF had reportedly similar effectiveness in patients with HF, regardless of the presence of systolic dysfunction.^232^, ^233^ In the study by Black‐Maier et al,^233^ there were no significant differences in procedural characteristics, arrhythmia‐free recurrence, or functional improvements between patients with HFpEF and those with HFrEF. A large‐scale study is necessary to prove the effectiveness of catheter ablation in AF patients with HFpEF.

##### 5.2.5.2 Valvular Heart Disease

Approximately 30% of patients with AF have some form of valvular heart disease. Valvular heart disease is associated with an increased thromboembolic risk, Recently, the number of patients with mitral stenosis has drastically decreased, but with mitral regurgitation is increasing. It is very important to distinguish functional mitral regurgitation from the primary form. When valve dysfunction is severe, AF can be regarded as a marker for progressive disease, thus favoring valve repair or replacement. The Maze procedure to restore sinus rhythm should be considered.

##### 5.2.5.3 Hypertension

Hypertension is a risk of new‐onset of AF, and promotes its progression. It also increases the risk of stroke. Sufficient antihypertensive treatment is required. There has been an argument that lowering blood pressure by angiotensin II‐receptor blockers may have more beneficial effects than using conventional Ca^2+^ channel blockers. In this regard, the J‐RHYTHM II study demonstrated that in patients with paroxysmal AF and hypertension, treatment of hypertension by candesartan did not have an advantage over amlodipine in reducing the frequency of paroxysmal AF.^234^ Similarly, in GISSI‐AF, treatment with valsartan was not associated with a reduction in the incidence of recurrent AF.^235^

##### 5.2.5.4 Diabetes Mellitus

Diabetes mellitus (DM) is a component of the CHADS_2_ score. Long disease duration leads to an increase in the risk of thromboembolism. In the Action to Control Cardiovascular Risk in Diabetes Study,^153^ a total of 10,082 patients with DM were studied in a randomized, double‐blind fashion. Intensive glycemic control did not affect the rate of new‐onset AF. Nonetheless, patients with DM and incident AF had an increased risk for morbidity and mortality compared with those without AF.^153^

##### 5.2.5.5 Obesity

The recommendation and level of evidence for management of obesity in AF patients is described in Table 26. Obesity promotes LV diastolic dysfunction, sympathetic activation and systemic inflammation.^236^ Conversely, stable weight loss decreases the AF burden and risk of AF recurrence.^236^

**Table 26:** Recommendation and Level of Evidence for Managing Obese Patients With AF

	COR	LOE	GOR (MINDS)	LOE (MINDS)
In obese or overweight patients with AF, weight loss together with management of other risk factors should be considered to reduce AF burden and symptoms	IIa	B	A	II

##### 5.2.5.6 Obstructive Sleep Disorder

Recommendations and levels of evidence for management of AF patients with obstructive sleep disorder are described in Table 27. Obstructive sleep disorder promotes AF, probably via hypoxemia, hypercapnia, inflammation, and exaggerated alteration of thoracic pressure, and autonomic dysfunction. When AF patients are suspected to have obstructive sleep disorder, further evaluation using multiple sleep latency tests is required. Following definitive diagnosis, appropriate management using continuous positive airway pressure is required.

**Table 27:** Recommendations and Levels of Evidence for Patients With AF and Obstructive Sleep Disorder

	COR	LOE	GOR (MINDS)	LOE (MINDS)
Interview to disclose obstructive sleep disorder	I	A	A	I
Treatment of obstructive sleep disorder to reduce AF recurrence and improve AF treatment	IIa	B	B	II

Abbreviation: AF, atrial fibrillation; COR, class of recommendation; GOR, grade of recommendation; LOE, level of evidence; MINDS, Medical Information Network Distribution Service.

##### 5.2.5.7 Chronic Kidney Disease

The recommendation and level of evidence for management of AF patients with chronic kidney disease (CKD) are described in Table 28. AF is frequently associated with CKD. The proper dosage of each DOACs is set by renal function (i.e., creatinine clearance). Caution is required, because the level of creatinine clearance in patients with AF decreases over time.^237^

**Table 28:** Recommendations and Levels of Evidence for Patients With AF and Chronic Kidney Disease

	COR	LOE	GOR (MINDS)	LOE (MINDS)
Assessment of kidney function by serum creatinine or creatinine clearance is recommended for all AF patients to detect kidney disease and to support correct dosing of AF therapy	I	A	A	II

Abbreviation: AF, atrial fibrillation; COR, class of recommendation; GOR, grade of recommendation; LOE, level of evidence; MINDS, Medical Information Network Distribution Service.

### 5.3 Anticoagulation Therapy

#### 5.3.1 Risk Assessment (Thromboembolic and Bleeding Risks)

##### 5.3.1.1 Risk Assessment for Cardiogenic Thromboembolism

Risk assessment for thromboembolism is important for the management of patients with AF in the clinical setting, especially when considering anticoagulation therapy (Table 29).

**Table 29:** Recommendations and Levels of Evidence for Risk Assessment of AF

	COR	LOE	GOR (MINDS)	LOE (MINDS)
Risk assessment for thromboembolism in patients with AF				
Use of CHADS_2_ score	I	B	B	IVa
Use of CHA_2_DS_2_‐VASc score	IIa	B	B	IVa
Use of CHA_2_DS_2_‐VA score (CHA_2_DS_2_‐VASc score except female sex)	IIa	B	B	IVa
Use of CHA_2_DS_2_‐VASc score to detect low‐risk cases	IIa	B	B	IVa
Consideration of other risk factors*^1^	IIb	B	C1	IVa
Risk assessment for bleeding complications in patients with AF				
Use of HAS‐BLED score	I	B	B	IVa
Consideration of major risk factors for bleeding*^2^	I	B	B	II

*^1^Cardiomyopathy, age (65–74 years), vascular disease (prior myocardial infarction, aortic plaque, and peripheral arterial disease), persistent and permanent AF, renal dysfunction, low body weight (≤50 kg), left atrial diameter (>45 mm). *^2^Older age (≥75 years), low body weight (≤50 kg), renal dysfunction (CCr ≤50 mL/min), antiplatelet use, and uncontrolled hypertension.

Abbreviation: AF, atrial fibrillation; CCr, creatinine clearance; COR, class of recommendation; GOR, grade of recommendation; LOE, level of evidence; MINDS, Medical Information Network Distribution Service.

Valvular AF should be distinguished from non‐valvular AF because warfarin is the only oral anticoagulant (OAC) approved for valvular AF. The effectiveness or safety of DOACs for valvular AF has never been proven.^238^, ^239^ “Valvular” means rheumatic mitral valve diseases (predominantly mitral stenosis) and the postoperative state with mechanical prosthetic valves. So far, among artificial valves, bioprosthetic valves have been categorized as “valvular” in the Guidelines for Pharmacotherapy of Atrial Fibrillation (JCS 2013).^2^ However, recent reports on the use of DOACs for patients with AF after bioprosthetic valve replacement have suggested that the efficacy of DOACs to prevent thromboembolism was comparable with that of warfarin;^240^, ^241^, ^242^ although the safety of DOACs for bleeding might have been superior to that of warfarin,^240^ the numbers of subjects were relatively small in those studies. According to this evidence, bioprosthetic valves were considered as “non‐valvular” in a joint consensus document from the heart rhythm associations of Europe, Asia, Africa, and Latin America in 2017.^243^ The 2019 AHA/ACC/HRS focuse update of the 2014 AHA/ACC/HRS guideline followed this standpoint.^244^

In this guideline, the definition of “non‐valvular” is updated to include bioprosthetic valves. The postoperative state of mitral valve repair (mitral annulorrhaphy or annuloplasty) and non‐rheumatic mitral regurgitation are included as “non‐valvular”, similar to the latest guidelines.^2^ (Because the effect‐efficacy of DOACs was described as “the prophylaxis of ischemic stroke and systemic embolism in patients with non‐valvular AF” in a statement of the virtues of medicine, the echocardiographic findings of valve stenosis and regurgitation have often misled physicians in an insured medical treatment.) Because DOACs have been approved for the prophylaxis of ischemic stroke and systemic embolism in patients with non‐valvular AF, the definition of “non‐valvular” is important at the selection of DOAC or warfarin. Generally, there is no inconvenience to including all valvular diseases as “non‐valvular”, except mitral stenosis and mechanical prosthetic valves.

In patients with non‐valvular AF (NVAF), because the accumulation of risk factors for thromboembolism increases the incidence of cardiogenic ischemic stroke,^245^, ^246^ it is recommended to determine appropriate anticoagulation therapy based on the risk assessment for thromboembolism. Assessments using risk scores have the advantage of standardization of risks evaluated by each physician.

In the Guidelines for Pharmacotherapy of Atrial Fibrillation (JCS 2013),^2^ the CHADS_2_ score was adopted as a risk assessment score for thromboembolism (Table 30)^245^ This score assigns 1 point each for the presence of congestive heart failure, hypertension, age ≥75 years, and diabetes mellitus, and 2 points for a history of stroke or transient ischemic attack (TIA) (maximum 6 points). The risks for thromboembolism based on the CHADS_2_ score are stratified as low, intermediate, and high for scores of 0, 1, and ≥2, respectively.

**Table 30:** CHADS_2_ Score

	Risk factors	Score
C	**C**ongestive heart failure	1
H	**H**ypertension	1
A	**A**ge ≥75 years	1
D	**D**iabetes mellitus	1
S_2_	**S**troke/TIA	2

Maximum score: 6.

Abbreviation: TIA, transient ischemic attack.

(Adapted from Gage BF, et al. 2001.^245^)

In the 2012 focused update of the ESC guidelines for the Management of Atrial Fibrillation,^247^ the CHA_2_DS_2_‐VASc score was adopted as a risk assessment score for thromboembolism (Table 31).^248^ This score comprises age ≥75 years and a history of stroke, TIA, or thromboembolism for 2 points and the other components of the CHADS_2_ score, vascular disease (prior myocardial infarction, peripheral artery disease, or aortic plaque), age 65–74 years, and sex category (female sex) for 1 point (maximum 9 points). In the Swedish Cohort Atrial Fibrillation study^249^ cited in the ESC guidelines,^244^ factors in addition to the CHADS_2_ score showed a significant risk for thromboembolism. Consequently, the CHA_2_DS_2_‐VASc score was adopted again in the 2016 ESC guidelines developed in collaboration with EACTS.^21^ This score was also adopted as a risk assessment score for thromboembolism in the 2014 AHA/ACC/HRS Guideline^250^ in the USA and by the Asian Pacific Heart Rhythm Society.

**Table 31:** CHA_2_DS_2_‐VASc Score

	Risk factors	Score
C	**C**ongestive heart failure/Left ventricular dysfunction	1
H	**H**ypertension	1
A_2_	**A**ge ≥75 years	2
D	**D**iabetes mellitus	1
S_2_	**S**troke/TIA/TE	2
V	**V**ascular disease (prior myocardial infarction, peripheral artery disease or aortic plaque)	1
A	**A**ge 65–74 years	1
Sc	**S**ex category (ie., female sex)	1

Maximum score: 9.

Abbreviation: TE, thromboembolism; TIA, transient ischemic attack.

(Adapted from Lip GY, et al. 2010.^248^)

In contrast, the CHADS_2_ score^245^ was again adopted in this Japanese guideline as well as in the previous version of the guidelines (JCS 2013),^2^ although opinions on which risk score is suitable for Japanese patients with NVAF differed among specialists. One principal reason is that the simple CHADS_2_ score should be propagated first, because the use of a risk score was not sufficiently widespread in the clinical setting when the latest guidelines were published (JCS 2013).^2^ Although the CHADS_2_ score has several problems and is imperfect, the risk score needs to be simple to encourage non‐specialists to continue active risk assessment.^251^ In addition, the factors added to the CHA_2_DS_2_‐VASc score (vascular disease, age 65–74 years, and female sex) were not a significant risk for ischemic stroke in Japanese patients with NVAF not receiving anticoagulation therapy, according to a pooled analysis of the 3 major domestic AF registries (J‐RHYTHM Registry, Fushimi AF Registry, and Shinken Database).^252^ Because a superior novel risk score of simplicity and predicability has not yet been established since the publication of the latest guidelines (JCS 2013),^2^ risk assessment for thromboembolism based on the CHADS_2_ score is the most suitable for Japanese patients for the moment. Other influential factors not included in the CHADS_2_ score can be thought of as “other risks” when considering anticoagulation therapy as in the latest guidelines (JCS 2013).^2^

###### 5.3.1.1.1 Incidence Rate of Thromboembolism in Each *CHADS_2_* Score

**Figure 11:**
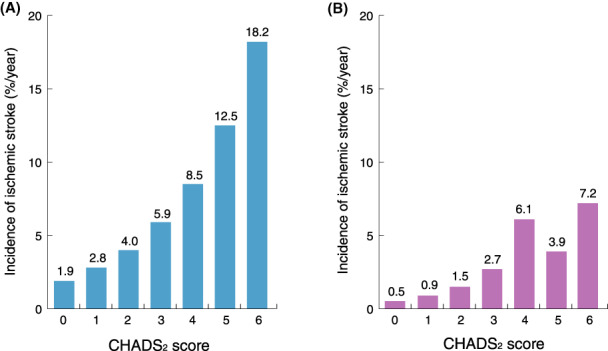
Incidence rates of ischemic stroke for each CHADS_2_ score. (**A**) Incidence rates of ischemic stroke with the original CHADS_2_ score (Adapted from Gage BF et al, 2001^245^). (**B**) Incidence rates of ischemic stroke in Japanese patients not receiving anticoagulation therapy (pooled analysis of the J‐RHYTHM Registry, Fushimi AF Registry, and Shinken Database). (Adapted from Suzuki S et al, 2015^252^)

Components of the CHADS_2_ score^245^ were derived from patients not receiving anticoagulation therapy among the pooled data of 5 randomized clinical trials (RCTs) in the Atrial Fibrillation Investigators (AFI) (n=3,432)^253^, ^254^, ^255^ and the first 2 trials in the Stroke Prevention in Atrial Fibrillation (SPAF) (n=2,012).^256^, ^257^, ^258^ Age, hypertension, prior cerebral ischemia (either stroke or TIA), and diabetes mellitus were detected from the AFI, whereas hypertension (systolic blood pressure [BP] >160 mmHg), prior cerebral ischemia, recent heart failure, and women aged ≥75 years were detected as risk factors for ischemic stroke from SPAF III. These factors were amalgamated and used to determine the 5 factors in the current CHADS_2_ score.^245^ This clinical classification scheme was validated using data from the National Registry of Atrial Fibrillation (NRAF) consisting of 1,733 Medicare beneficiaries aged 65–95 years with NVAF who were not prescribed warfarin at hospital discharge.^245^ The incidence rate of ischemic stroke per 100 patient‐years without antithrombotic therapy increased by a factor of 1.5 for each 1‐point increase in the CHADS_2_ score: 1.9 for a score of 0, 2.8 for 1, 4.0 for 2, 5.9 for 3, 8.5 for 4, 12.5 for 5, and 18.2 for 6, respectively (Figure 11A).^245^ Although these rates are often used as the gold standard for informed consent, the incidence rates of ischemic stroke in Japanese patients with NVAF not receiving anticoagulation therapy in a pooled analysis of the 3 major AF registries^252^ were obviously lower than those in the original report by Gage et al^245^ and in other reports^259^ from foreign countries: 0.5 for a score of 0, 0.9 for 1, 1.5 for 2, 2.7 for 3, 6.1 for 4, 3.9 for 5, and 7.2 for 6, respectively (Figure 11B).^252^ Therefore, the original incidence rates^245^ cannot necessarily be extrapolated to current Japanese patients. The original rates should be interpreted cautiously because all subjects in the NRAF used for validation of the CHADS_2_ score were older (range, 65–95; mean, 81 years) and standard therapeutic drugs for hypertension and heart failure were different from those used today.

###### 5.3.1.1.2 Validity of Components of the CHADS_2_ Score

Although the CHADS_2_ score consists of 5 factors, only 3 (hypertension, age ≥75 years, and a history of stroke or TIA) were identified as significant risks for ischemic stroke in Japanese patients with NVAF not receiving anticoagulation therapy, according to a pooled analysis of the 3 major domestic AF registries (J‐RHYTHM Registry, Fushimi AF Registry, and Shinken Database),^252^ and congestive heart failure or diabetes mellitus were not (Table 32).^252^

**Table 32:** Hazard Ratios for Ischemic Stroke in Japanese Patients With AF

Factors	HR (95% CI)
Age	
<65 years65–74 years≥75 years	Reference1.12 (0.53–2.37)2.31 (1.18–4.52)
Women	1.07 (0.65–1.76)
Hypertension	1.69 (1.01–2.86)
Diabetes mellitus	1.18 (0.64–2.15)
Cerebral infarction or TIA	3.25 (1.86–5.67)
Heart failure	0.86 (0.45–1.65)
Coronary artery disease	0.52 (0.22–1.26)
Antiplatelet use	1.42 (0.86–2.32)

Multivariate Cox regression model. Pooled analysis of the Shinken Database, J‐RHYTHM Registry, and Fushimi AF Registry in patients with AF not receiving anticoagulation therapy (n=3,588).

Abbreviation: AF, atrial fibrillation; CI, confidence interval; HR, hazard ratio; TIA, transient ischemic attack.

(Adapted from Suzuki S, et al. 2015.^249^)

####### 5.3.1.1.2.1 Congestive Heart Failure

“Congestive heart failure” is defined as recent exacerbation of heart failure (HF) within the preceding 100 days.^245^, ^256^ However, because the definition of exacerbation is vague in the clinical setting, it can be determined by symptoms, examination findings, or administration of medications for HF, regardless of the classification of HF. The risk for thromboembolism is comparable between patients with HF with reduced ejection fraction (HFrEF) and those with preserved EF (HFpEF).^231^ In phase III trials using DOACs,^260^, ^261^, ^262^, ^263^ HF was defined as either left ventricular ejection fraction (LVEF) <40%, New York Heart Association (NYHA) class II or more, or HF symptoms within previous 3–6 months.

Possible reasons why HF was not identified as a significant risk factor for thromboembolism include the difference in current standard therapeutic drugs for HF compared with the 1990s, HF being a stronger risk factor for all‐cause and cardiovascular deaths rather than for thromboembolism,^264^ and the severity or duration of HF not taken into consideration.^265^, ^266^ In a subanalysis of the ENGAGE AF‐TIMI 48 trial,^265^ severe HF with NYHA class III or IV was a significant risk factor for thromboembolism (hazard ratio [HR] 1.45, 95% confidence interval [CI] 1.12–1.88). In addition, the incidence of stroke or systemic embolism markedly increased in the 30 days after admission for HF (HR 12.0, 95% CI 4.59–31.98) in a subanalysis of the Fushimi AF Registry.^266^

####### 5.3.1.1.2.2 Hypertension

In the Framingham study, hypertension was a risk factor for stroke even in patients having a history and adequate BP control under antihypertensive medications.^267^ Therefore, the definition of hypertension in the CHADS_2_ score includes a history of hypertension in addition to the criteria of hypertension in those days of systolic BP ≥160 mmHg. Because hypertension is currently defined as systolic BP ≥140 mmHg and/or diastolic BP ≥90 mmHg in the Japanese Society of Hypertension Guidelines for the Management of Hypertension (JSH 2019),^268^ it is defined as BP ≥140/90 mmHg and/or a history (including under treatment) in this guideline, as stated previously. In subanalyses of hypertension in the phase III trials using DOACs, hypertension was a significant risk factor for stroke or systemic embolism in the ROCKET‐AF trial,^269^ and the ARISTOTLE trial.^270^ In contrast, in a subanalysis of the J‐RHYTHM Registry,^271^ neither hypertension (including its history and/or under treatment) nor BP value at the time of enrollment was an independent risk factor for thromboembolism, but the incidence of thromboembolism in the highest quartile of systolic BP (≥136 mmHg) at the time closest to the event was significantly higher than that in the lowest quartile (<114 mmHg) (odds ratio 2.88, 95% CI 1.75–4.74). A subanalysis of the Fushimi AF Registry^272^ also showed that event rates in patients with hypertension were comparable to those without hypertension, but the incidence of stroke/systemic embolism and hemorrhagic stroke in patients with a baseline systolic BP ≥150 mmHg was significantly higher than in those with adequate BP control.

Given that patients with inadequate BP control have consistently indicated high event rates in all studies,^269^, ^270^, ^271^, ^272^, ^273^ including trials in which hypertension was not identified as a significant risk factor for thromboembolism,^271^, ^273^ appropriate BP control may result in a reduced risk of thromboembolism in patients with NVAF.

####### 5.3.1.1.2.3 Age (≥75 Years)

“Age ≥75 years” is indicated as an especially high risk for thromboembolism among factors even with the same CHADS_2_ score of 1.^249^, ^274^ In some Japanese registry studies, it was also shown that age ≥75 years was a strong risk factor for thromboembolism (HR 2.3–2.8).^252^, ^271^, ^275^

####### 5.3.1.1.2.4 Diabetes Mellitus

Although “diabetes mellitus” has been identified as a risk factor for thromboembolism in other studies,^249^, ^253^ it has not been in Japan.^252^, ^271^, ^275^ The reason can be speculated as the control status of blood glucose levels or the recent progression of oral hypoglycemic agents not being reflected in these results, but the precise causes remain unknown. However, HRs were >1.0 in most studies.^249^, ^252^, ^253^, ^271^ Given that diabetes mellitus is not working to reduce events, it was kept as a component of the CHADS_2_ score in this guideline.

####### 5.3.1.1.2.5 History of Stroke or Transient Ischemic Attack

“History of stroke or TIA” is defined as prior cerebral ischemia (either stroke or TIA) and patients with it are categorized in the secondary prevention of stroke. As this factor is indicated to facilitate a higher risk for thromboembolism than the other risk factors,^258^, ^276^, ^277^, ^278^, ^279^ both the CHADS_2_ and CHA_2_DS_2_‐VASc scores assign it 2 points.

Accordingly, of the components of the CHADS_2_ score, age ≥75 years and a history stroke or TIA should be considered most intensively as risk factors; additionally, appropriate control of BP and HF is also important to prevent thromboembolism in patients with NVAF.

###### 5.3.1.1.3 Factors Not Included in the CHADS_2_ Score

In the latest vision of the Japanese guidelines (JCS 2013),^2^ cardiomyopathy, advanced age (65–74 years), and vascular disease (prior myocardial infarction, aortic plaque, and peripheral arterial disease) were listed as “other risks” when considering anticoagulation therapy.

####### 5.3.1.1.3.1 Age (65–74 Years)

Advanced age (65–74 years) was a significant risk factor for stroke in the AFI^253^ and the Swedish Cohort Atrial Fibrillation study.^249^ In the 2016 Focused Update of the Canadian Cardiovascular Society Guidelines for the Management of Atrial Fibrillation,^280^ patients aged ≥65 years are recommended to receive anticoagulation therapy, regardless of other risk factors. It named the score as “CHADS65” due to attaching importance to age ≥65 years. However, it has not been identified as a significant risk factor for thromboembolism in Japan, even though the HR was slightly high at 1.0–1.3.^252^, ^271^, ^275^

####### 5.3.1.1.3.2 Vascular Disease

Although “vascular disease” including prior myocardial infarction,^253^ aortic plaque,^281^ and peripheral arterial disease^282^ was identified as a risk factor for thromboembolism and listed in the latest guidelines (JCS 2013),^2^ these factors were not a significant risk for thromboembolism in Japanese patients.^252^, ^271^, ^275^

####### 5.3.1.1.3.3 Cardiomyopathy

In patients with “cardiomyopathy,” the coagulation system is often activated.^283^, ^284^ In some cohort studies of Japanese patients with NVAF, cardiomyopathy, especially hypertrophic cardiomyopathy, was reportedly an independent risk factor for stroke.^284^, ^285^

####### 5.3.1.1.3.4 Sex Category (Female sex)

Because being “female”^249^, ^253^ had proved not to be a solo risk factor in AF patients aged <65 years without other organic diseases,^247^, ^274^, ^286^ it was deleted in the latest guidelines (JCS 2013).^2^ Subsequently, given that female sex was not detected as a significant risk factor for thromboembolism in subanalyses of both the J‐RHYTHM Registry^287^ and the Fushimi AF Registry,^288^ it was determined not to describe this as a solo risk factor in this guideline. It seems reasonable that female sex is dealt with as a modifier of other risk factors independent of sex.^289^, ^290^

####### 5.3.1.1.3.5 Thyrotoxicosis

“Thyrotoxicosis” is not considered as a risk factor for thromboembolism, as described in the previous version of the Japanese guidelines (JCS 2013),^2^ because thyrotoxicosis or hyperthyroidism has not been sufficiently validated as a solo risk factor for thromboembolism.

####### 5.3.1.1.3.6 Type of Atrial Fibrillation

Although the incidence rate of ischemic stroke appears to be comparable between patients with paroxysmal and persistent/permanent AF,^291^, ^292^ the risk for thromboembolism in patients with persistent or permanent AF is reportedly higher than that in those with paroxysmal AF in subanalyses of phase III trials using DOACs^293^, ^294^, ^295^, ^296^ and the Fushimi AF Registry.^297^ In addition, the risk of adverse events was transiently elevated during the progression period from paroxysmal to sustained AF.^298^

####### 5.3.1.1.3.7 Echocardiography Findings

Among the findings of echocardiography, LV systolic function (LV fractional shortening <25%),^255^, ^257^ left atrial (LA) dysfunction,^299^ and LA diameter (>45 mm) on transthoracic echocardiography (TTE),^275^ and dense spontaneous echocardiographic contrast (so‐called smoke‐like or moyamoya echo) in the LA, LA appendage thrombus, and peak LA appendage flow velocity (<20 cm/s)^281^ on transesophageal echocardiography (TEE) have been reported as risk factors for thromboembolism.

####### 5.3.1.1.3.8 Low Body Weight and Renal Dysfunction

“Low body weight (BW) or low body mass index (BMI)” and “renal dysfunction” often lead to issues in patients with AF, especially in elderly patients. In the Fushimi AF Registry, BW ≤50 kg^300^ and creatinine clearance (CCr) <30 mL/min^301^ were significant risk factors for thromboembolism. In contrast, in the J‐RHYTHM Registry, BMI <18.5 kg/m^2 302^ and CCr <30 mL/min^303^ were stronger risk factors for all‐cause death than for thromboembolism. Factors not included in the CHADS_2_ score in subanalyses of both registries are shown in Table 33.^275^, ^287^, ^288^, ^292^, ^297^, ^300^, ^301^, ^302^, ^303^

**Table 33:** Risk Factors for Thromboembolism Not Included in the CHADS_2_ Score

Factors not included in the CHADS_2_ score	J‐RHYTHM Registry	Fushimi AF Registry
Sex	Male (vs. female)^287^OR 1.24 (95% CI 0.83–1.86)	Female (vs. male)^288^HR 0.74 (95% CI 0.54–1.00)
LAD	Not evaluated	LAD >45 mm (vs. ≤45 mm)^275^HR 1.74 (95% CI 1.25–2.42)
BW or BMI	BMI <18.5 kg/m^2^ (vs. 18.5–24.9 kg/m^2^)^302^HR 1.22 (95% CI 0.63–2.38)	BW ≤50 kg (vs. >50 kg)^300^HR 2.13 (95% CI 1.39–3.27)
Renal dysfunction	CCr <30 mL/min (vs. ≥80 mL/min)^303^HR 1.69 (95% CI 0.62–4.62)	CCr <30 mL/min (vs. ≥50 mL/min)^301^HR 1.68 (95% CI 1.04–2.65)
Type of AF	Permanent (vs. paroxysmal)^302^HR 1.007 (95% CI 0.955–1.061)	Paroxysmal (vs. persistent)^297^HR 0.51 (95% CI 0.30–0.88)

Abbreviation: AF, atrial fibrillation; BMI, body mass index; BW, body weight; CCr, creatinine clearance; CI, confidence interval; HR, hazard ratio; LAD, left atrial diameter; OR, odds ratio.

**Figure 12:**
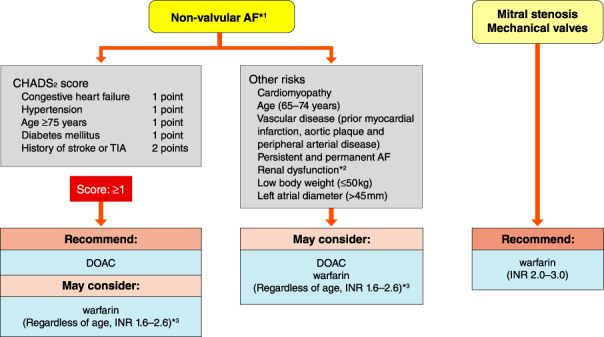
Anticoagulation therapy in AF. *^1^Bioprosthetic values are included in non‐valvular AF. *^2^Regarding anticoagulation according to renal function, see **“3.2.3 Selection of Direct Oral Anticoagulants”** and Table 36. *^3^Regarding target INR 1.6–2.6 in non‐valvular AF, INR close to 2.0 is recommended as possible. INR 2.0–3.0 may be considered in high‐risk patients aged <70 years with a history of stroke or CHADS_2_ score ≥3. AF, atrial fibrillation; DOAC, direct oral anticoagulant; INR, international normalized ratio; TIA, transient ischemic attack.

Accordingly to these AF registry studies in Japan, in the present guideline persistent or permanent AF, low BW (≤50 kg), renal dysfunction, and LA diameter (>45 mm) are newly listed as “other risks” when considering anticoagulation therapy (Figure 12), in addition to cardiomyopathy, age (65–74 years), and vascular disease.

###### 5.3.1.1.4 Predictive Ability of *CHADS_2_* Score for Events

The predictive ability of a risk score for events is generally evaluated using the c‐statistic (or c‐index).^304^ C‐statistics of the schemas in the AFI and SPAF, which were used for derivations of the CHADS_2_ score, were 0.68 and 0.74, respectively. In contrast, that in the NRAF used for validation was 0.82, which was higher than each of the values of the AFI or SPAF.^245^ Novel risk scores with higher predictive ability have been developed by adding other factors such as the CHA_2_DS_2_‐VASc score,^248^ the R_2_CHADS_2_ score^305^ by adding renal dysfunction, the ABC (age, biomarkers, and clinical history) stroke risk score^306^ by adding biomarkers (N‐terminal pro‐B‐type natriuretic peptide [NT‐proBNP], high‐sensitivity troponin T), and so on. However, because the c‐statistics of these scores were 0.6–0.7, it is difficult to determine whether predictive ability has improved compared with that of the CHADS_2_ score.^245^ A validation study of the J‐RHYTHM Registry^289^ also demonstrated that the c‐statistic of either the CHA_2_DS_2_‐VASc score (0.595) or the CHA_2_DS_2_‐VA score (0.624), when female sex was removed, was not higher than that of the CHADS_2_ score (0.638).

###### 5.3.1.1.5 Detectability of *CHADS_2_* Score for True Low‐Risk Patients

Approximately half of patients with NVAF correspond to a CHADS_2_ score of 0 or 1,^274^, ^307^ for which the effectiveness of warfarin has not been proven. The prevalence of patients with a CHADS_2_ score of 0 was reportedly 15.6% in the J‐RHYTHM Registry,^307^ and 11.2% in the Fushimi AF Registry.^308^ In patients with cerebral infarction, those with a CHADS_2_ score of 0 before the onset of stroke comprised 7.3% and were not necessarily rare in the SAMURAI‐NVAF.^309^ The CHADS_2_ score is limited for distinguishing low‐risk patients, but the CHA_2_DS_2_‐VASc score is thought to be useful to detect an especially true low‐risk one who may not require anticoagulation therapy.^310^, ^311^ It can be proposed that anticoagulation therapy is not needed for patients with a CHA_2_DS_2_‐VASc score of 0, except for females, who showed no risk for thromboembolism in Japan.^287^, ^288^, ^289^

##### 5.3.1.2 Risk Assessment for Bleeding

In patients with NVAF, risk assessment for bleeding is important for the determination of anticoagulation therapy and to prevent bleeding complications during anticoagulation therapy.

###### 5.3.1.2.1 HAS‐BLED Score and Other Risk Scores

The HAS‐BLED score (Table 34)^312^ was adopted in the ESC guidelines in 2010 as a risk score for predicting bleeding.^282^ Comparing to the HEMORR_2_HAGES score published in 2006,^313^ this score is able to evaluate bleeding risks more simply and accurately in the clinical setting.^314^ The incidence rates of major bleeding per 100 patient‐years in each HAS‐BLED score were reportedly 1.13 for a score of 0, 1.02 for 1, 1.88 for 2, 3.74 for 3, 8.70 for 4, and 12.50 for 5,^312^ and a high risk was defined as a score ≥3. Because hypertension, prior stroke, and advanced age were mutual factors in the CHADS_2_ score,^245^ high‐risk patients for bleeding are also at high risk for thromboembolism.^315^ However, note that the definitions of risk factors in the HAS‐BLED score differ from those in the CHADS_2_ score. In the HAS‐BLED score, hypertension is defined as uncontrolled in patients with systolic BP >160 mmHg and advanced age is defined as >65 years. Labile international normalized ratio (INR) assumed that patients receive warfarin. Although novel risk scores with higher predictive ability have been developed, such as the ATRIA hemorrhagic risk score,^316^ simplified by omitting INR, the ORBIT score,^317^ and the ABC bleeding risk score by adding biomarkers,^318^ the c‐statistics of these scores were 0.6–0.7 and thus, the predictive ability has not markedly improved.

**Table 34:** HAS‐BLED Score

	Risk factors	Score
H	**H**ypertension*^1^	1
A	**A**bnormal renal and liver function (1 point each)*^2^	1 or 2
S	**S**troke	1
B	**B**leeding*^3^	1
L	**L**abile INR*^4^	1
E	**E**lderly (>65 years)	1
D	**D**rugs or alcohol (1 point each)*^5^	1 or 2

Maximum score: 9.

*^1^Hypertension is defined as systolic blood pressure >160 mmHg. *^2^Abnormal renal function is defined as chronic dialysis or renal transplantation or serum creatinine ≥200 *μ*mol/L (2.26 mg/dL). Abnormal liver function is defined as chronic hepatic disease (e.g., cirrhosis) or biochemical evidence of significant hepatic derangement (e.g., bilirubin >2×upper limit of normal, in association with AST/ALT/ALP >3×upper limit normal). *^3^Bleeding refers to previous bleeding history and/or predisposition to bleeding (e.g., bleeding diathesis, anemia). *^4^Labile INR refers to unstable/high INR or poor time in therapeutic range (i.e., <60%). *^5^Drugs/alcohol use refers to concomitant use of drugs, such as antiplatelet agents, nonsteroidal anti‐inflammatory drugs, or alcohol abuse.

Abbreviation: ALP, alkaline phosphatase; ALT, alanine aminotransferase; AST, aspartate aminotransferase; INR, international normalized ratio.

(Reprinted from Pisters R, et al. 2010.^312^) Copyright (2010) American College of Chest Physicians, with permission from Elsevier. https://www.sciencedirect.com/journal/chest

In actual clinical practice, especially during anticoagulation therapy, it is important to assess bleeding risks using the “major risk factors for bleeding” as listed below since the latest guidelines (JCS 2013),^2^ and to control those factors suitable for intervention among the “factors related to intracranial hemorrhage” also listed below.

###### 5.3.1.2.2 Major Risk Factors for Bleeding

According to the results from a subanalysis of the RE‐LY trial,^319^ a post marketing surveillance of dabigatran,^320^ and some domestic AF registry studies,^321^, ^322^ older age (≥75 years), low BW (≤50 kg), renal dysfunction (CCr ≤50 mL/min), and antiplatelet use are major risk factors for bleeding during anticoagulation therapy. In patients receiving dabigatran, a history of gastrointestinal bleeding and concomitant use of P‐glycoprotein inhibitors are also risk factors for major bleeding (HR ≥3).^320^ In the present guideline, “uncontrolled hypertension” has been newly added as a major risk factor, based on results from the ROCKET AF trial,^269^ ARISTOTLE trial,^270^ and subanalyses of the Japanese AF registry studies.^271^, ^272^ Physicians should watch for critical bleeding complications in these high‐risk patients regardless of the anticoagulant used.

###### 5.3.1.2.3 Factors Related to Intracranial Hemorrhage

In a subanalysis of the RE‐LY trial, age, prior stroke or TIA, use of aspirin or warfarin, and not being Caucasian were identified as factors related to intracranial hemorrhage (ICH).^323^ To date, hypertension, smoking, excessive alcohol consumption, East Asian ethnicity, hypocholesterolemia, hepatitis or liver cirrhosis, advanced age, prior cerebral infarction, and cerebral microbleeds on magnetic resonance imaging (MRI) have been identified as risk factors related to the development of ICH. Hypertension, prior cerebral infarction, hepatitis or liver cirrhosis, hyperglycemia, and antithrombotic therapy were reportedly predisposing factors for enlargement of the ICH.^324^, ^325^, ^326^ To prevent the incidence of ICH, the use of DOACs with a low risk for ICH, adequate control of BP and blood glucose levels, abstention from smoking and excessive alcohol intake, and avoiding antiplatelet use could be recommended.^238^, ^327^

In actual clinical practice, it is crucial to assess the risks for both thromboembolism and bleeding in individual patients and to select treatments that lead to a net clinical benefit^329^ based on a consideration of the risks and benefits.

#### 5.3.2 Direct Oral Anticoagulants and Warfarin

##### 5.3.2.1 Characteristics of Direct Oral Anticoagulants

A direct thrombin inhibitor, dabigatran,^260^ and factor Xa inhibitors, rivaroxaban,^261^ apixaban,^262^ and edoxaban^263^ have been approved in Japan as oral anticoagulants with indications for prevention of ischemic stroke in AF, and are now available in daily clinical practice. These were first named as “new oral anticoagulants”, but have been called with several different names in contrast with VKA.^22^ Here, we call them DOACs.^330^

DOACs have several advantages compared with warfarin, including fixed dosing, no need for regular blood sampling for dose adjustment, low incidence of intracranial hemorrhage (ICH), less frequency of interactions with food and other drugs, rapid onset of action, and relatively short half‐life, all of which accordingly lead to no need or shortening of heparin replacement in the perioperative period. On the other hand, DOACs have several disadvantages, including a contraindication in severe renal dysfunction, rapid offset of action if a dose is missed, due to their short half‐life, no adequate measures for severe bleeding under anticoagulation therapy, and increased cost burden by patients. Among these, measures for severe bleeding have been partially established due to the recent development of antidotes for DOACs^331^, ^332^, ^333^, ^334^, ^335^ (see Section 3.6).

##### 5.3.2.2 Selecting Between Direct Oral Anticoagulants and Warfarin (Table 35, ^21^, ^238^, ^239^, ^260^, ^261^, ^262^, ^263^, ^330^, ^336^, ^337^, ^338^, ^339^, ^340^, ^341^, ^342^, ^343^, ^346^, ^348^, ^349^, ^350^, ^351^, ^352^, ^353^, ^354^, ^355^, ^356^, ^357^, ^358^, ^359^ and Table 36)

**Table 35:** Recommendations and Levels of Evidence for Anticoagulation for AF

	COR	LOE	GOR (MINDS)	LOE (MINDS)
Selecting between DOACs and warfarin				
Warfarin is recommended for stroke prevention in AF patients with moderate‐to‐severe mitral stenosis^260^, ^261^, ^262^, ^263^, ^336^	I	B	A	IVa
Warfarin is recommended for stroke prevention in AF patients with mechanical heart valves^238^, ^260^, ^261^, ^262^, ^263^, ^336^	I	B	A	II
When oral anticoagulation is started in a patient with AF who is eligible for DOACs (dabigatran, rivaroxaban, apixaban, or edoxaban), a DOAC is recommended in preference to warfarin^260^, ^261^, ^262^, ^263^, ^336^, ^337^	I	A	A	I
When patients are treated with warfarin, TTR should be kept as high as possible* ^338^, ^339^, ^340^, ^341^, ^342^	I	A	A	II
AF patients already on treatment with warfarin may be considered for DOAC treatment if TTR is not well controlled despite good adherence (except for cases of contraindications to DOACs)^260^, ^262^, ^336^, ^337^, ^343^	IIa	A	A	II
Selection of DOACs				
For patients with high risk of bleeding, consider agent/dose of DOAC that was significantly lower than warfarin in the large‐scale clinical trials (apixaban, dabigatran 110 mg bid, edoxaban)^337^, ^346^, ^348^, ^349^	IIa	A	B	II
Coagulation assay during warfarin treatment				
Optimal range of PT‐INR under warfarin therapy in NVAF patients without a history of ischemic stroke and having low thromboembolic risks (i.e., CHADS_2_ score ≤2 points) is 1.6–2.6 irrespective of age^350^, ^351^, ^352^	IIa	B	B	IVa
Optimal range of PT‐INR under warfarin therapy in NVAF patients with a history of ischemic stroke or having high thromboembolic risks (i.e., CHADS_2_ score ≥3 points, or cancer patients) is 1.6–2.6 in elderly patients (age ≥70 years) and 2.0–3.0 in younger patients (age <70 years). Even in elderly patients, INR should be kept ≥2.0 as much as possible, unless it threatens the safety for bleeding^353^, ^354^, ^355^	IIa	B	B	IVa
Blood sampling during long‐term follow‐up				
CCr (for apixaban, serum creatinine, body weight, and age) should be evaluated before DOACs are started as judgement for contraindications or dose reduction^346^, ^348^, ^349^	I	A	B	II
Considering the pathogenesis or patient characteristics that possibly decrease coagulation activity (hemophilia, blood type O, etc.), coagulation tests before starting DOACs should be evaluated^356^, ^357^	IIa	C	B	IVa
After DOACs are started, blood tests (renal function, liver function, hemoglobin, etc.) should be done at least once per 12 months^21^, ^330^, ^358^	IIa	C	B	V
In elderly patients (≥75 years) or frail patients, blood tests (renal function, liver function, hemoglobin, etc.) should be done at least once per 6 months^330^	IIa	C	C1	VI
In patients with CCr <60 mL/min, blood test (renal function, liver function, hemoglobin, etc.) should be done at least once per X months (X=CCr/10)^330^	IIa	C	C1	VI

*It has been reported that the threshold TTR under warfarin therapy that reduces mortality compared with no anticoagulation is ≥60% and that yielding better cost‐effective medical care compared with DOACs was ≥65–90% (variation according to the referenced DOAC).^359^ However, the TTR should always be targeted at 100% and thresholds above should be regarded as the least acceptable levels.

Abbreviation: AF, atrial fibrillation; CCr, creatinine clearance; COR, class of recommendation; DOAC, direct oral anticoagulant; GOR, grade of recommendation; LOE, level of evidence; MINDS, Medical Information Network Distribution Service; NVAF, non‐valvular atrial fibrillation; PT‐INR, prothrombin time‐international normalized ratio; TTR, time in therapeutic range.

**Table 36:** Selection of DOACs for Non‐Valvular Atrial Fibrillation According to Renal Function

		Normal renal function–moderate renal dysfunction (CCr ≥30 mL/min)	Severe renal dysfunction (CCr <30 mL/min)		Maintenance HD
			(15≤ CCr <30)	(CCr <15)	
DOACs	dabigatran	Indication	Contraindication	Contraindication	Contraindication
	rivaroxaban	Indication	Indication	Contraindication	Contraindication
	apixaban	Indication	Indication	Contraindication	Contraindication
	edoxaban	Indication	Indication	Contraindication	Contraindication
warfarin		Indication	Indication	Indication	Relative contraindication

Abbreviation: CCr, creatinine clearance; DOAC, direct oral anticoagulant; HD, hemodialysis.

###### 5.3.2.2.1 Patients With Normal Renal Function to Mild Renal Dysfunction

When oral anticoagulation is started in a patient with AF who is indicated for a DOAC (dabigatran, rivaroxaban, apixaban, or edoxaban) among patients with normal renal function to mild renal dysfunction (creatinine clearance [CCr] ≥30 mL/min), a DOAC is recommended as the first choice in preference to a VKA,^21^ because of the convenience of drug prescription, stability of drug effects, low frequency of interactions with foods and other drugs, and the low incidence of ICH. The efficaciousness and safety of DOACs have been reported to be equal or superior to warfarin in randomized clinical trials with both Asian (including Japanese) and non‐Asian subjects or in observational studies with Japanese.^346^, ^348^, ^349^, ^360^, ^361^, ^362^

For AF patients with a history of valvular replacement (mechanical valve) or with a diagnosis of mitral stenosis (mostly rheumatic), namely valvular AF, only warfarin not DOACs is indicated.^260^, ^261^, ^262^, ^263^ Meanwhile, small‐sized studies of DOAC usage in AF patients with bioprosthetic valves have accumulated.^240^, ^241^, ^242^ However, during the 3 months after valvular replacement with a bioprosthetic valve, anticoagulation with warfarin is recommended, even in patients with sinus rhythm,^363^ and studies of AF patients with bioprosthetic valves have excluded this time period. Therefore, the current recommendations of anticoagulation for AF patients with bioprosthetic valves are (i) using warfarin for the 3 months after the valvular replacement and (ii) thereafter, can switch to a DOAC.

###### 5.3.2.2.2 Patients With Severe Renal Dysfunction

DOACs are contraindicated for patients with severe renal dysfunction (CCr <30 mL/min for dabigatran, and CCr <15 mL/min for rivaroxaban, apixaban, and edoxaban)^260^, ^261^, ^262^, ^263^ (Table 37). Therefore, in AF patients with CCr <15 mL/min, warfarin is the only anticoagulant that can be selected for prevention of ischemic stroke. However, warfarin has a relative contraindication for patients with severe renal impairment on the package insert.^364^ The bleeding risk with warfarin usage in AF patients with severe renal impairment is extremely high, which may offset the merit of preventing ischemic stroke.^365^

**Table 37:** Dosage and Administration of Direct Oral Anticoagulants for Non‐Valvular Atrial Fibrillation

	dabigatran	rivaroxaban	apixaban	edoxaban
Standard dose	150 mg twice daily	15 mg once daily	5 mg twice daily	60 mg once daily
Reduced dose	110 mg twice daily	10 mg once daily	2.5 mg twice daily	30 mg once daily
Dose reduction criteria	•CCr <50 mL/min•P‐glycoprotein inhibitors •Age ≥70 years•History of gastrointestinal bleeding (for dabigatran, dose reduction is considered, but not required)	CCr <50 mL/min	2 of the following: •Serum Cr ≥1.5 mg/dL •Age ≥80 years•Body weight ≤60 kg	Any 1 of the following:•CCr <50 mL/min•P‐glycoprotein inhibitors•Body weight ≤60 kg
Contraindications for renal dysfunction	CCr <30 mL/min	CCr <15 mL/min	CCr <15 mL/min	CCr <15 mL/min

Abbreviation: CCr, creatinine clearance.

###### 5.3.2.2.3 Maintenance Hemodialysis

When anticoagulation is considered for patients on maintenance hemodialysis (HD), the indication should be carefully considered. The Japanese Society for Dialysis Therapy recommends that administration of warfarin for patients on maintenance HD is a relative contraindication because it increases not only bleeding events but also thromboembolic events.^366^ The present guidelines also recommend that the administration of warfarin for patients on maintenance HD is a relative contraindication. However, in daily clinical practice, warfarin is widely used in the perioperative period of catheter ablation, in patients with mechanical valves, or in patients who need secondary prevention of ischemic stroke. Therefore, the present guidelines recommend that the administration of warfarin be considered for such cases, even for patients on maintenance HD.

###### 5.3.2.2.4 Patients With Valvular Atrial Fibrillation

For AF patients with a history of valvular replacement (mechanical valve) or with a diagnosis of mitral stenosis (mostly rheumatic), namely valvular AF, only warfarin but not DOACs is indicated.^260^, ^261^, ^262^, ^263^ In the present guidelines, AF with a history of valvular replacement with a bioprosthetic valve is included as NVAF, which is indicated for DOAC usage. However, during the 3 months after a valvular replacement with a bioprosthetic valve, anticoagulation with warfarin is recommended even in patients with sinus rhythm^363^ and, meanwhile, studies evaluating the efficaciousness and safety of DOACs in AF patients with bioprosthetic valves^240^, ^241^, ^242^ excluded this period. Therefore, the current recommendations of anticoagulation for AF patients with bioprosthetic valves are (i) using warfarin for the 3 months after the valvular replacement and (ii) thereafter, can switch to a DOAC.

##### 5.3.2.3 Selection of Direct Oral Anticoagulants

The differences in the pharmacological profiles of the DOACs are shown in Table 37.^367^ Several reviews have proposed flow charts for selection of DOACs based on their pharmacokinetic profiles, including metabolic pathways (renal excretion, hepatic excretion, and intestinal excretion) and number of doses, and the results of the subanalyses of phase III studies divided by the various patient backgrounds.^367^, ^368^, ^369^, ^370^ Of note, a review^367^ described Asian subanalyses including Japanese or a clinical trial with Japanese patients.^346^, ^348^, ^349^ However, although such flow charts may be useful for daily clinical practice, they cannot be regarded as absolute methods of choice because there have been no clinical trials that have directly compared among the DOACs.

It is essential to use DOACs with the dose and administration indicated in the package inserts (Tables 7 and 37), which have been determined through multiple clinical trials considering the characteristic pharmacokinetics for each DOAC. Moreover, phase III clinical trials have demonstrated efficacy and safety of anticoagulation for stroke prevention by DOACs with on‐label dosing that are equal or superior to those of warfarin.^337^

In daily clinical practice in Japan, there are more AF patients who are applicable for the dose reduction criteria of DOACs compared with Western countries due to high age and low body weight. Therefore, it is an issue in Japan whether the appropriateness of on‐label dosing may not be sufficiently validated in AF patients who are either older age or have mild to severe renal dysfunction; in such patients, evidence that verifies the appropriateness of on‐label dosing needs to accumulate. In the J‐ELD AF registry,^371^ which registered ∼3,000 Japanese elderly AF patients (≥75 years) under treatment with on‐label dosing of apixaban demonstrated similar efficiency and safety between the standard dose (n=1,284) and the reduced dose (n=1,747).

##### 5.3.2.4 Coagulation Test Under Prescription of Warfarin

During warfarin administration, the intensity of anticoagulation effect is measured by the prothrombin time‐international normalized ratio (PT‐INR) and according to the PT‐INR, the dose of warfarin should be adjusted. In the JCS guidelines updated in 2013,^2^ the target range of PT‐INR was recommended as 1.6–2.6 for elderly patients (age ≥70 years)^353^ and 2.0–3.0 for younger patients (age <70 years). However, several reports from multicenter cohorts in Japan have demonstrated that clinicians targeted the PT‐INR range of 1.6–2.6, even in younger patients, presumably due to a fear of bleeding events.^307^, ^372^

In the nationwide multicenter cohort of the J‐RHYTHM Registry, optimal PT‐INR under warfarin therapy was investigated using ∼8,000 AF patients. In that analysis, PT‐INR 1.6–2.6 was determined as the range in which both the risk of thromboembolism and major bleeding became minimal, regardless of elderly or younger patients.^350^, ^351^, ^352^

Meanwhile, in studies of Japanese AF patients with acute ischemic stroke, the severity and prognosis after ischemic stroke according to PT‐INR at the time of the occurrence of ischemic stroke were evaluated.^354^, ^355^ In those studies, various measurements differed according to the PT‐INR, including the size of the infarction and National Institute of Health Stroke Scale (NIHSS) at the time of occurrence, and the severity of neural dysfunction or the prognosis of systemic function after ischemic stroke. In patients with PT‐INR 1.6–2.0, the measurements were similar to those with PT‐INR <1.6. Meanwhile, in patients with PT‐INR ≥2.0, the infarct size was small and the functional prognosis was relatively good.

Based on this evidence, for prevention of ischemic stroke in NVAF patients without a history of ischemic stroke and having low thromboembolic risks (i.e., CHADS_2_ score ≤2 points), the optimal range of PT‐INR under warfarin therapy would be 1.6–2.6 irrespective of elderly or younger patients. For this target range, the attending physician should aim to attain the middle (PT‐INR 2.0), but not the lower (1.6 or 1.7) value.

Meanwhile, for prevention of ischemic stroke in NVAF patients with a history of ischemic stroke or having high thromboembolic risks (i.e., CHADS_2_ score ≥3 points, or cancer patients), the optimal range of PT‐INR under warfarin therapy would be 1.6–2.6 in elderly patients (age ≥70 years) and 2.0–3.0 in younger patients (age <70 years). Even in elderly patients, INR should be kept ≥2.0 as much as possible unless it threatens the safety for bleeding events.

As such, the present guidelines separate the optimal target range of warfarin therapy into primary prevention and secondary prevention: (i) in the former, the target range is 1.6–2.6 irrespective of elderly or younger patients, and (ii) in the latter, the target range is 1.6–2.6 in elderly patients (age ≥70 years) and 2.0–3.0 in younger patients (age <70 years), which follows the target range in the previous guidelines.

The time in therapeutic range (TTR) is the measurement that calculates the percentage of time in which the PT‐INR under warfarin treatment is controlled within the target range.^374^ The TTR should be kept as high as possible. Although it has been reported that the threshold of TTR under warfarin therapy that reduces mortality compared with no anticoagulation is ≥60% and that yielding better cost‐effective medical care compared with DOACs was ≥65–90% (variation according to the referenced DOAC),^359^ the TTR should always be targeted at 100% and the thresholds above should be regarded as the least acceptable levels.

In the SAMURAI‐AF registry, which registered AF patients with a history of ischemic stroke, patients with optimal PT‐INR at the time of occurrence of ischemic stroke had a double risk of recurrence.^376^ In such cases, the patients may have a potential thrombophilia such as malignancy or antiphospholipid antibody syndrome.

##### 5.3.2.5 Coagulation Test Under Prescription of Direct Oral Anticoagulants

DOACs have emerged as oral anticoagulants that do not need regular monitoring. Actually, in phase III trials, DOACs without regular monitoring showed similar efficacy and safety compared with warfarin with regular monitoring.^260^, ^261^, ^262^, ^263^ However, it is recommended to measure the anticoagulation intensity of DOACs in high‐risk patients such as elderly patients, patients with renal dysfunction, and in patients who have experienced thromboembolism or bleeding events. However, it has not been well established how to interpret or how to respond to the results of the anticoagulation tests measured in daily clinical practice. Therefore, difficult cases should be referred to experts who have extensive experience with the prescription of DOACs (Table 38, ^330^, ^377^, ^378^, ^379^, ^380^).

**Table 38:** Average Plasma Concentration and Response of Coagulation Tests for DOACs

	dabigatran^377^	rivaroxaban^378^	apixaban^379^	edoxaban^380^
90% intervals of plasma concentration of DOACs with AF patients				
Peak plasma concentration for standard dose (ng/mL)	64–443	78.9–585.1	91–321	49.4–345.3
Trough plasma concentration for standard dose (ng/mL)	31–225	2.5–128.7	42–230	4.8–40.7
Effect of DOACs on coagulation test				
PT	+	+++	+	++
APTT	+++	+	+	+

Abbreviation: AF, atrial fibrillation; APTT, activated partial thrombplastin time; DOAC, direct oral anticoagulant; PT, prothrombin time.

(Adapted from Steffel J, et al. 2018,^330^ van Ryn J, et al. 2010^377^ Suzuki S, et al. 2017^378^ Kowalsk K, et al. 2014^379^ Suzuki S, et al. 2019.^380^)

###### 5.3.2.5.1 Blood Tests Before Starting Direct Oral Anticoagulants

Each DOAC has its own criteria of contraindication determined by renal function (Tables 36 and 37). Moreover, to determine the dose of DOACs, measurement of renal function is mandatory. In consideration of the pathogenesis or patient characteristics that may potentially decrease the coagulation activity (e.g., hemophilia,^356^ blood type O,^357^ etc.), a coagulation test before starting DOACs is encouraged. In hemophilia, the judgement for starting DOACs is based on the level of coagulation activity.^356^

###### 5.3.2.5.2 Blood Tests After Starting Direct Oral Anticoagulants

The bleeding complications after starting DOACs are concentrated in the first 3 months, but especially in the first month. Therefore, for early detection of bleeding, it is useful to check the temporal change in hemoglobin in elderly patients or patients with a history of gastrointestinal bleeding. It is also recommended to check changes in renal or liver function.

The 90% intervals of the distribution of the plasma concentration of DOACs at peak and trough in AF patients are shown in Table 38.^330^, ^377^, ^378^, ^379^, ^380^ The 90% intervals are known as the “on therapy range”,^381^, ^382^ which can indicate the optimal range of the plasma concentration of DOACs used in daily clinical practice. When the plasma concentration of the DOAC exceeds the 90% intervals, the bleeding risk may significantly increase.^383^, ^384^ Therefore, a coagulation test to measure the intensity of anticoagulation in the early phase after starting a DOAC is recommended. For this purpose, the hemoclot thrombin inhibitory assay (HTI) and activated partial thromboplastin time (aPTT) are used for direct thrombin inhibitors, whereas the anti‐Xa assay (AXA) and prothrombin time (PT) are used for factor Xa inhibitors. However, measurement of PT for factor Xa inhibitors cannot be fully encouraged due to the wide variation in response according to the reagents used and the targeted factor Xa inhibitor.

###### 5.3.2.5.3 Blood Tests During Long‐Term Prescription of Direct Oral Anticoagulants

When the prescription of DOACs continues for more than a few years, renal function gradually decreases over time.^385^ Therefore, measurement of renal function at least once per year is recommended.^330^, ^358^ Moreover, as most DOACs have hepatic metabolism, liver function should also be measured. For early detection of subclinical gastrointestinal bleeding, measurement of hemoglobin is also recommended. In elderly patients (≥75 years), blood testing at least once per 6 months is recommended. Moreover, in patients with CCr <60 mL/min, blood test once per X months (X=CCr/10) is recommended.^330^

#### 5.3.3 Prevention of Thromboembolism for Cardioversion

Two case–control studies reported that the risk of thromboembolic complication for cardioversion of AF without oral anticoagulation was 1–5%.^386^, ^387^ The risk is reduced by warfarin for 3 weeks before and 4 weeks after cardioversion.^388^, ^389^, ^390^ This anticoagulant regimen is applied for patients with AF ≥48 h duration or when the duration of AF is unknown. The duration of warfarin therapy is within the target range of PT‐INR; that is, we need more than 3 weeks before cardioversion if we start warfarin in naïve patients. The evidence of the safety of cardioversion of AF without anticoagulation in patients with AF duration <48 h is limited. Left atrial thrombus and thromboembolism can be induced by short‐term AF, but the necessity of anticoagulation is not determined.

In addition, post‐hoc analyses of a subset of patients undergoing cardioversion in phase 3 trials of approved DOACs,^391^, ^392^, ^393^ 4 prospective RCTs,^395^, ^396^, ^397^, ^398^ a cohort study^394^ and meta‐analyses^399^, ^400^, ^401^ have evaluated the safety and efficacy of DOACs for cardioversion as an alternative to warfarin. The results were consistent and supported the idea that DOACs are an alternative to warfarin for patients undergoing cardioversion. For naïve patients, DOACs may be better for reducing the time before cardioversion, because the DOACs are effective from the day of starting, whereas warfarin takes at least several days to reach its therapeutic range. For patients with AF that requires immediate cardioversion because of hemodynamic instability in such clinical settings as angina, acute coronary syndrome, shock, or pulmonary edema, we should do it (if the duration of AF >48 h, with heparin i.v.) and should start and continue anticoagulation for at least 4 weeks.

**Figure 13:**
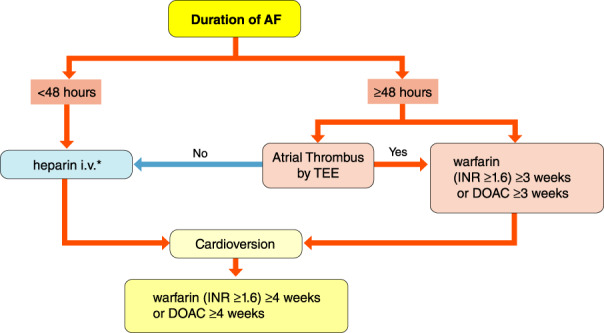
Anticoagulant therapy for cardioversion. *2,000–5,000 U unfractionated heparin i.v. (evidence for the dose is poor). In case of AF lasting <48 h without anticoagulants, DOAC is recommended unless contraindicated as the effect of anticoagulation is rapid. AF, atrial fibrillation; DOAC, direct oral anticoagulant; INR, international normalized ratio; TEE, transesophageal echocardiography.

After cardioversion and the restoration of sinus rhythm, functional recovery of the left atrium and left atrial appendage delay (i.e., atrial stunning) may take more than a few weeks.^402^ Meta‐analysis of 32 studies concerning cardioversion of AF and atrial flutter demonstrated that 98% of thromboembolic events occurred in the 10 days after cardioversion.^403^ The decision about long‐term anticoagulation therapy (beyond 4 weeks) is based on relapse of AF, which includes paroxysmal and asymptomatic forms, thromboembolic risk and bleeding risk (Figure 13).

An alternative to waiting 3 weeks before cardioversion is to perform transesophageal echocardiography (TEE) to exclude thrombus. In patients with AF ≥48 h duration, a RCT comparing cardioversion with and without TEE was performed (ACUTE trial).^404^ The traditional arm (n=603) was without TEE and administered warfarin for 3 weeks before and 4 weeks after cardioversion. In the TEE arm (n=619), immediate cardioversion was performed after heparin i.v. if no thrombus was detected in the left atrium. If a thrombus was detected, warfarin therapy was performed for 3 weeks. Cardioversion was performed if no thrombus was detected after reevaluation of TEE. Warfarin was continued for 4 weeks after cardioversion. The number of days before cardioversion was significantly short in the TEE arm (mean 3.0 days vs 30.6 days), whereas the success rate of cardioversion and the rates of thromboembolism and major bleeding were not significantly different at 8 weeks after cardioversion. These results showed that TEE is an acceptable screening procedure for cardioversion of AF.^404^

Prospective randomized trials with DOACs and warfarin for cardioversion of AF (X‐VerT, ENSURE‐AF, XANTUS, and EMANATE) included a TEE arm and confirmed the efficacy and safety.^395^, ^396^, ^397^, ^398^ If the risk of cerebral infarction is extremely high in patients with AF lasting <48 h, TEE is considered before cardioversion.

Cardioversion of atrial flutter also has the complication of cerebral infarction or systemic embolism, so anticoagulant therapy (warfarin or DOAC) should be applied as for AF before and after cardioversion.^405^, ^406^

#### 5.3.4 Perioperative Anticoagulation Management (e.g., Tooth Extraction, Gastrointestinal Endoscopy, Surgery, etc)

Patients on anticoagulation therapy often undergo invasive procedures (e.g., examinations and treatments). Specific clinical departments that provide such invasive procedures face difficult decisions as to whether their patients need to be temporarily withdrawn from anticoagulants during the perioperative period. Such decisions should be made on a patient‐by‐patient basis, taking several factors into account, such as the risk of thromboembolism, type of anticoagulant used, and the risk of bleeding associated with the particular invasive procedure to be performed. Physicians who are to perform such invasive procedures should consult with the clinic or hospital prescribing the anticoagulant to the patient to help decide whether the patient can be temporarily withdrawn from anticoagulation therapy. Furthermore, they should also consult with the patient to explain the risks associated with interruption, specifically the risk of thromboembolism, and receive consent.

The categories of bleeding risks associated with invasive procedures performed across different specialties are shown in Table 39. In general, to prevent thromboembolism, interruption of anticoagulation therapy is not recommended for patients undergoing invasive procedures in which hemostasis is possible. For invasive procedures that require interruption of anticoagulation therapy, the period of interruption should be kept to a minimum and the treatment should be re‐initiated as soon as hemostasis is confirmed. The categories shown in Table 39 are based on standard invasive procedures; it should be noted that interruption may still be needed in high bleeding risk cases for procedures that are categorized as low risk of bleeding. It should also be noted that the guidelines are specific for elective invasive procedures and do not apply to emergency procedures. Recommendations and levels of evidence for continuation of anticoagulant therapy during invasive procedures are shown in Table 40.

**Table 39:** Classification of Elective Surgical Interventions According to Bleeding Risk

Minor bleeding risk
•Dental surgery [extraction, abscess incision, paradontal surgery, implant positioning, etc.]
•Cataract surgery
•Diagnostic gastroenterological endoscopic procedures without biopsy [upper/lower gastroenterological endoscopy, capsule endoscopy, ERCP, etc.]
•Superficial surgery [abscess incision, dermatologic excisions, etc.]
•Breast biopsy, mammothome biopsy
Low bleeding risk
•Gastroenterological endoscopic procedures with low bleeding risk [balloon‐assisted endoscopy, gastroenterological pancreatic duct/biliary duct stenting, endoscopic papillary balloon dilation, etc.]
•Endoscopic mucosal biopsy
•Prostate biopsy
•Transurethral surgery [bladder biopsy, TUR‐Bt, PVP, TUL, etc.]
•Percutaneous nephrostomy
•Glaucoma or vitreous surgery
•Arthroscopic surgery
•Mastectomy
•Oto‐rhino‐laryngological surgery, head and neck surgery
•Cardiac device implantation
•Angiography, intravacular surgery
•Electrophysiological study or catheter ablation (except AF ablation)
High bleeding risk
•Gastroenterological endoscopic procedures with high bleeding risk [polypectomy, ESD, endoscopic duodenal papillectomy, endoscopic treatment of esophageal and gastric varices, EUS‐FNA, etc.]
•Transbronchial lung biopsy
•Spinal or epidural anesthesia
•Craniotomy, spinal cord surgery
•Carotid endarterectomy
•Thoracic surgery (including thoracoscopic surgery)
•Abdominal/pelvic surgery (including laparoscopic surgery)
•Breast cancer surgery
•Major orthopedic surgery
•Reconstructive surgery for head and neck cancer
•Lower extremity artery bypass surgery
•Liver biopsy
•Kidney biopsy
•Transrectal prostate biopsy
•TUR‐P
•ESWL
•PNL
High bleeding and thromboembolic risk
•AF ablation

Abbreviation: AF, atrial fibrillation; ERCP, endoscopic retrograde cholangiopancreatography; ESD, endoscopic submucosal dissection; ESWL, extracorporeal shockwave lithotripsy; EUS‐FNA, endoscopic ultrasonographyguided fine‐needle aspiration; PNL, percutaneous nephrolithotripsy; PVP, photoselective vaporization of the prostate; TUL, transurethral ureterolithotripsy; TUR‐Bt, transurethral resection of the bladder tumor; TUR‐P, transurethral resection of the prostate.

**Table 40:** Recommendations and Levels of Evidence for Continuation of Anticoagulant Therapy During Invasive Procedures

	COR	LOE	GOR (MINDS)	LOE (MINDS)
Continuation of anticoagulants during minor bleeding risk procedures	I	A	A	I
Continuation of warfarin controlled within therapeutic range during tooth extraction	I	A	A	I
Continuation of DOACs during tooth extraction	IIa	C	C1	VI
Continuation of anticoagulants during low bleeding risk procedures	IIa	C	C1	VI
Continuation of warfarin controlled within therapeutic range during cardiac device implantation	IIa	B	B	II
Continuation of DOACs during cardiac device implantation	IIa	C	C1	IVa
Continuation of warfarin controlled within therapeutic range during gastroenterological endoscopic procedures with low bleeding risk	IIa	B	C1	IVa
Continuation of DOACs during gastroenterological endoscopic procedures with low bleeding risk, carried out at a time avoiding peak DOAC blood concentration	IIa	C	C1	IVa
Interruption of anticoagulants during minor to low bleeding risk procedures in which hemostasis is difficult when bleeding happens	IIa	C	C1	VI
Interruption of anticoagulants during high bleeding risk procedures	IIa	C	C1	VI
Heparin bridging during interruption of warfarin	IIb	B	C2	II
Heparin bridging during interruption of DOACs	IIb	B	C2	IVa
Interruption of warfarin or continuation of warfarin controlled within therapeutic range during gastroenterological endoscopic procedures with high bleeding risk	IIa	C	C1	IVb
Interruption of DOACs on the morning of the procedure and re‐initiation on the morning after gastroenterological endoscopic procedures with high bleeding risk	IIa	C	C1	VI
Continuation of warfarin or DOACs for at least 3 weeks before AF ablation in patients with persistent AF or high risk (CHADS_2_ score ≥2)	IIa	C	C1	VI
Continuation of warfarin or dabigatran during AF ablation	I	A	A	I
Continuation of rivaroxaban or apixaban or edoxaban during AF ablation	IIa	B	B	II
One or two dose skip of DOACs during AF ablation	IIa	B	B	II

Abbreviation: AF, atrial fibrillation; COR, class of recommendation; DOAC, direct oral anticoagulant; GOR, grade of recommendation; LOE, level of evidence; MINDS, Medical Information Network Distribution Service.

##### 5.3.4.1 Dental Extraction

Safety of continuing oral anticoagulant therapy with warfarin for patients undergoing dental extraction or minor dental procedures was shown in several RCTs,^407^, ^408^, ^409^, ^410^ and a meta‐analysis.^411^ In the meta‐analysis, perioperative continuation of warfarin was not associated with an increased risk for clinically significant bleeding or minor bleeding, compared with interrupting warfarin therapy. A review showed that approximately 1% of patients with warfarin therapy discontinued specifically for dental procedures had serious embolic complications (including deaths).^412^ Therefore, continuation of oral anticoagulant therapy with warfarin in patients undergoing dental extraction or minor dental procedures is recommended.

Evidence regarding oral anticoagulant therapy using DOACs in patients undergoing dental procedures is lacking. In a subanalysis of the RE‐LY trial, there was no significant difference in the rates of periprocedural ischemic stroke or systemic embolism between patients receiving dabigatran and those receiving warfarin (0.5% for both), who required surgery, including dental procedures.^413^ A study from Japan regarding the risk of bleeding in patients receiving a DOAC or warfarin without cessation reported that postoperative bleeding occurred in 4 extractions (3.1%) among 128 patients receiving DOACs (all extractions in DOAC patients were performed 6–7 h after taking the DOAC), and in 23 (8.8%) among 262 patients receiving warfarin, and there was no statistically significant difference between the 2 groups.^414^ Thus, continuation of DOACs in patients undergoing dental procedures is recommended, as with the recommendation for warfarin.

##### 5.3.4.2 Gastroenterological Endoscopy

It is reported that the incidence rate of stroke was approximately 1% in AF patients with adjusted anticoagulation (including withholding, reversal, or changing dose) who underwent endoscopy.^415^ In prospective observational studies, there was no significant difference for post‐procedural bleeding following endoscopy between patients with discontinued and continued anticoagulant therapy.^416^, ^417^ Diagnostic endoscopic procedures without biopsy can potentially be performed for patients with continued anticoagulant therapy, and expert opinion suggests that diagnostic endoscopy with biopsy can also potentially be performed for patients with continued anticoagulant therapy with controlled therapeutic INR,^418^, ^419^ but definite evidence is lacking. heparin bridging therapy for endoscopic procedures with high bleeding risk significantly increased the risk of post‐procedural bleeding.^420^, ^421^

The Japan Gastroenterological Endoscopy Society published “Guidelines for gastroenterological endoscopy in patients undergoing antithrombotic treatment” in 2012,^422^, ^423^ and an Appendix including DOACs in 2017.^424^, ^425^ The guideline classified gastroenterological endoscopic examination and treatment procedures according to bleeding risk into 4 categories: (i) diagnostic gastroenterological endoscopic procedures without biopsy, (ii) endoscopic mucosal biopsy (excluding endoscopic ultrasonography‐guided fine‐needle aspiration), (iii) gastroenterological endoscopic procedures with low bleeding risk, and (iv) gastroenterological endoscopic procedures with high bleeding risk. For category (i), withdrawal of warfarin or DOAC is not required. For categories (ii–iv), warfarin withdrawal is not required but it should be confirmed that the PT‐INR is within the therapeutic range. For patients receiving DOACs in category (ii) or (iii), DOAC withdrawal is not required, but performing the procedure outside the peak DOAC blood concentration estimated from the time of administration is recommended. Patients receiving DOACs in category (iv) should discontinue the DOAC on the morning of the procedure. Oral administration of DOAC may be resumed on the morning after the procedure.

##### 5.3.4.3 Surgery

Although there was no established evidence or standard management approach during surgery with high bleeding risk, discontinuation of warfarin therapy and heparin bridging has been performed empirically during the perioperative period.^426^, ^427^ However, the BRIDGE trial showed that the incidence of arterial thromboembolism was comparable between the no‐bridging group (0.4%) and the bridging group (0.3%), and the incidence of major bleeding was significantly lower in the no‐bridging group (1.3%) compared with the bridging group.^428^ Some observational studies reported the similar results to the BRIDGE trial.^429^, ^430^ Generally, routine heparin bridging is not needed for patients with AF who require an interruption in warfarin treatment for an elective invasive procedure with high bleeding risk.

On the other hand, heparin bridging is recommended to be considered for valvular AF patients (mechanical valve or rheumatic mitral stenosis), or non‐valvular AF patients at very high thromboembolic risk, such as history of ischemic stroke within 3 months or extremely high CHADS_2_ score. Analyses developed from the BRIDGE trial showed bridging to only be beneficial for patients with HAS‐BLED scores ≤2 and with CHA_2_DS_2_‐VASc scores of ≥6 (presumed CHADS_2_ score of ≥4).^431^

A subanalysis of the international multicenter phase III trials of each DOAC regarding perioperative events has been published.^413^, ^432^, ^433^, ^434^ Meta‐analysis of these studies showed no significant difference in the risks of thromboembolic events, major bleeding, minor bleeding, and overall mortality at 30 days following surgery or procedure between perioperative interruption of DOAC and interruption of warfarin.^435^

In the case of invasive procedures that carry a high risk for major bleeding, it is recommended to take the last DOAC dose ≥48 h before surgery.^330^ For patients on dabigatran with CCR ≥80 mL/min, the last administration should be taken ≥48 h, for those with CCR 50–79 mL/min it should be taken ≥72 h, and for those with CCR 30–49 mL/min it should be taken ≥96 h before surgery.

Perioperative heparin bridging is not recommended in patients who discontinued DOACs before surgery.^330^ In a substudy of the RE‐LY trial, with dabigatran interruption, bridging was associated with significantly higher incidence of major bleeding than no‐bridging, but for any thromboembolic events there was no difference between bridging and no‐bridging.^436^ In general, heparin bridging in not needed during the interruption of DOAC therapy, but it can be considered in patients at very high thromboembolic risk as described above.

##### 5.3.4.4 Cardiac Device Implantation

Two RCTs are available regarding bridging anticoagulation using warfarin in patients who required cardiac implantable electronic device surgery.^437^, ^438^ They concluded that a strategy of bridging therapy at the time of device implantation markedly increased the incidence of clinically significant device‐pocket hematoma as compared with continued warfarin treatment, and thromboembolic complications were rare and did not differ significantly between bridging therapy and continued warfarin treatment. Additionally, device‐pocket hematoma is associated with a significantly increased risk of infection requiring hospitalization.^439^

There are several observational studies that show continued DOAC treatment during device implantation surgery was not significantly associated with increased incidence of clinically relevant bleeding events.^440^, ^441^, ^442^

##### 5.3.4.5 Atrial Fibrillation Ablation

Patients undergoing AF ablation have a high risk of bleeding and thromboembolism, requiring appropriate anticoagulation therapy during the perioperative period. Large‐scale observational studies of AF ablation demonstrated that cardiac tamponade and perforation are common hemorrhagic complications, with the incidence ranging from 1.2% to 2.5% and from 0.9% to 1,5%, respectively.^443^, ^444^, ^445^, ^446^ The Japanese Catheter Ablation Registry of Atrial Fibrillation (J‐CARAF), which included 8,319 cases, also reported similar incidence rates.^447^ Thus, although technical advancements are expected, it remains essential to consider the risk of hemorrhagic complications. The risk of thromboembolism is further increased in left atrial ablation, with 0.1–0.3% of patients developing cerebral infarction during the perioperative period.^443^, ^444^, ^445^, ^446^ Collectively, appropriate management of anticoagulation therapy during the perioperative period is essential.

A recent international expert consensus recommended that warfarin be administered uninterrupted.^448^ This recommendation is based on evidence from an RCT (COMPARE trial) demonstrating that the use of warfarin reduced the risk of hemorrhagic complications in addition to that of embolism.^449^

Similarly, recent RCTs have generated additional evidence on the use of DOACs. By comparing dabigatran (RE‐CIRCUIT trial),^450^ rivaroxaban (VENTURE‐AF trial),^451^ apixaban (AXAFA‐AFNET 5 trial),^452^ and edoxaban (ELIMINATE‐AF trial)^453^ with uninterrupted warfarin, they found that the efficacy and safety of uninterrupted administration of DOACs during the perioperative period are equivalent or superior to those of warfarin in terms of the risk of embolism and hemorrhagic complications. However, these studies included relatively small numbers of patients. In addition, variations in trial design should be noted; these variations included the half‐life of each DOAC and when the last dose was administered prior to the procedure.

There are limited data on minimally interrupted DOAC therapy, in which administration is interrupted 1–2 times prior to the procedure. An RCT conducted in Japan (ABRIDGE‐J trial) compared minimally interrupted dabigatran and uninterrupted warfarin, and demonstrated that the risk of hemorrhage was higher in the warfarin group, but the risk of embolism was equivalent between groups.^454^ Another Japanese single‐center RCT compared uninterrupted and minimally interrupted DOAC, and reported no significant difference in the incidence of embolism or hemorrhagic complications among the 4 DOACs, suggesting that minimal interruption is acceptable.^455^ A recent multicenter study conducted across hospitals in the Kyushu region (KYU‐RABLE study) also demonstrated that uninterrupted administration of edoxaban during the perioperative period (immediately after the procedure on the day of the surgery) is effective and safe.^456^

Many factors should be considered in order to select the appropriate DOAC, as well as to determine whether interruption is needed (and if so, for how long) during the perioperative period for patients undergoing AF ablation. These factors include the patient‐specific risk of thromboembolism and bleeding, and differences in drug metabolism and route of excretion among DOACs, in addition to the availability of neutralizing agents such as idarucizumab for dabigatran^332^ and Andexanet alfa for factor Xa inhibitors (unapproved, as of March 2020).^457^ The “2018 JCS/JHRS Guideline on Non‐Pharmacotherapy of Cardiac Arrhythmias”^4^ should also be referred to.

#### 5.3.5 Antithrombotic Therapy in Patients With Atrial Fibrillation Concomitant With Ischemic Heart Disease

##### 5.3.5.1 Randomized Clinical Trials in Patients With Atrial Fibrillation Concomitant With Ischemic Heart Disease

The prevalence of the coexistence of ischemic heart disease (IHD) among patients with atrial fibrillation (AF) has been reported to be 8–15% in Japan,^307^, ^308^, ^362^, ^458^, ^459^ and these patients are encountered commonly in clinical practice. The JCS 2018 “Guidelines on Diagnosis and Treatment of Acute Coronary Syndrome”^460^ and on “Revascularization of Stable Coronary Artery Disease”^461^ recommend dual antiplatelet therapy (DAPT) of aspirin plus a P2Y_12_ receptor antagonist (P2Y_12_ inhibitor) after stent implantation in patients undergoing percutaneous coronary intervention (PCI) as class I/evidence level A. Therefore, in patients with AF concomitant with undergoing PCI, triple therapy defined as a DAPT plus an OAC for stroke prevention is needed. In 2019, the Academic Research Consortium proposed a consensus definition of patients at a high bleeding risk (HBR) among those undergoing PCI, which was based on a review of the available evidence. The use of OACs is fulfilled by the major criteria for HBR patients who are defined as having an annual incidence of bleeding events estimated to be >4%.^462^ Because the concomitant use of antiplatelet therapy in these HBR patients would dramatically increase bleeding events, antithrombotic management focusing on the prevention of bleeding events is becoming the main trend worldwide.

The WOEST trial^463^ (n=573) was the first to test the clinical benefit of dual therapy with a VKA plus clopidogrel as a counterpart to the triple therapy of a VKA plus DAPT. Among the patients taking OACs and undergoing a PCI, dual therapy reduced the annual incidence of major bleeding more than the triple therapy, and surprisingly, the annual incidence of the composite cardiovascular events also lowered with the dual therapy. Regarding the benefit of VKA monotherapy as compared with dual therapy in AF patients with stable coronary artery disease (CAD), a nationwide Danish cohort registry provided insightful results that VKA monotherapy had a similar risk of a myocardial infarction/coronary death but decreased risk of serious bleeding events, as compared with dual therapy of a VKA plus aspirin or VKA plus clopidogrel.^464^ An open‐label randomized trial comparing an OAC alone (including four‐thirds of the patients taking warfarin and the remaining patients taking DOACs) and dual therapy with an OAC plus single antiplatelet in patients with AF and stable CAD beyond 1 year after PCI (OAC‐ALONE: Optimizing Antithrombotic Care in patients with AtriaL fibrillatiON and coronary stEnt study) was conducted in Japan.^465^ In that trial, there were no differences in the incidences of the primary endpoint (composite of all‐cause death, myocardial infarction, stroke, or systemic embolism) and major bleeding between the OAC alone therapy and dual therapy, which supported the results obtained from the nationwide Danish cohort study.^464^

As DOACs are being increasingly used as an alternative to warfarin in clinical practice,^362^ the clinical evidence for DOACs in antithrombotic therapy management among AF patients undergoing PCI is becoming established. The PIONEER PCI trial^466^ compared the primary safety outcome and the occurrence of a major adverse cardiovascular event (a composite of death from cardiovascular causes, myocardial infarction, or stroke) between dual therapy with rivaroxaban 15 mg plus P2Y_12_ inhibitor (n=709) for 12 months and triple therapy with VKA plus DAPT adjusted for 1, 6, or 12 months (n=706). Similar to the WOEST trial, the dual therapy had a lower risk of clinically significant bleeding, but the rate of a major adverse cardiovascular event was similar between the dual and triple therapies. The RE‐DUAL PCI trial^467^ (n=2,725), which compared dual therapy with dabigatran (110 mg or 150 mg twice daily) over 12 months and a P2Y_12_ inhibitor (clopidogrel or ticagrelor) with warfarin‐based triple therapy with a P2Y_12_ inhibitor and aspirin (for 1–3 months), showed a similar result that the dual therapy had a lower risk of bleeding and was noninferior to the triple therapy with respect to the risk of thromboembolic events. In the AUGUSTUS trial,^468^ 4,614 AF patients who underwent PCI with a P2Y_12_ inhibitor were randomly assigned to receive apixaban or VKA and aspirin or a matching placebo for 6 months. The P2Y_12_ inhibitor, an antithrombotic regimen that included apixaban, without aspirin, resulted in less bleeding and fewer hospitalizations without significant differences in the incidence of ischemic events than the regimens that included VKA, aspirin, or both. The ENTRUST trial^469^ included 1,506 AF patients who had undergone PCI and were randomly assigned to either edoxaban plus a P2Y_12_ inhibitor for 12 months or VKA plus DAPT (for 1–12 months). The trial also showed consistent results of a bleeding risk reduction and an equivalent ischemic risk of the dual therapy as for the triple therapy. Although these 4 trials established the clinical benefit of dual therapy with a DOAC and P2Y_12_ inhibitor as compared with a warfarin‐based triple therapy in AF patients with CAD, the endpoints in the trials were only evaluated during a limited duration of 1 year after PCI.

Regarding the prognostic effect of DOACs in patients with stable CAD beyond 1 year after PCI, the AFIRE (Atrial Fibrillation and Ischemic events with rivaroxaban in patiEnts with stable coronary artery disease Study) trial was published in 2019.^470^ This multicenter, open‐label trial conducted in Japan randomly assigned 2,236 patients with AF who had undergone PCI or coronary artery bypass grafting (CABG) more than 1 year earlier or who had angiographically confirmed CAD not requiring revascularization to receive monotherapy with rivaroxaban or a combination therapy with rivaroxaban plus a single antiplatelet agent. The trial was stopped early because of increased mortality in the combination‐therapy group. Rivaroxaban monotherapy was superior to combination therapy for the primary safety endpoint of major bleeding, and surprisingly, the risk of the primary efficacy endpoint (composite of a stroke, systemic embolism, myocardial infarction, unstable angina requiring revascularization, or death from any cause) was reduced by 28%, and was statistically noninferior to the combination therapy. Similar data, even for DOACs, supporting the results from the nationwide Danish registry^464^ and OAC‐ALONE trial^465^ were shown by this trial in Japan.

**Figure 14:**
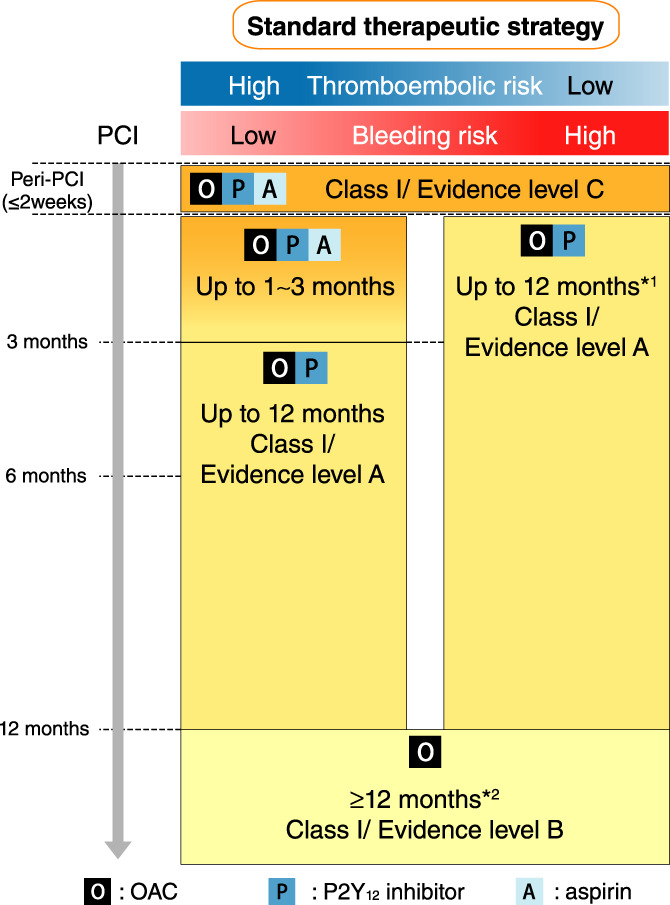
Recommended choice and duration of antithrombic therapy in atrial fibrillation concomitant with ischemic heart disease. *^1^Short duration of 6 months of dual therapy can be considered in patients with a very high bleeding risk. *^2^Continuation of dual therapy with an OAC and aspirin (or P2Y_12_ inhibitor) for longer than 12 months can be considered in patients with a very high thromboembolic risk. PCI, percutaneous coronary intervention, OAC, oral anticoagulant.

On the other hand, to explore the optimal duration of de‐escalation from triple therapy to dual therapy, the ISAR‐TRIPLE trial,^471^ a randomized open‐label study, was conducted. The result was that 6 weeks of triple therapy with aspirin and clopidogrel after PCI in patients receiving an OAC was noninferior to 6 months of a triple therapy with respect to ischemic events and major bleeding events. Furthermore, in the WOEST trial^463^ and the 4 DOAC AF PCI trials^466^, ^467^, ^468^, ^469^ as described above, the patients assigned to the dual therapy group were de‐escalated from a triple therapy to a dual therapy with a DOAC plus P2Y_12_ inhibitor by cessation of aspirin in the periprocedural phase (maximum 2 weeks in the AUGUSTUS trial) after the PCI, which strongly supported the clinical acceptability of an early termination of aspirin after PCI. Meta‐analyses have also suggested that aspirin should be terminated early after PCI.^472^, ^473^ On the basis of these data in this field described above, this guideline recommends that the timing of de‐escalation from triple therapy to dual therapy with an OAC and clopidogrel should be within 2 weeks after PCI (Figure 14). The present European Society of Cardiology (ESC) guidelines^21^, ^330^, ^474^ also consider dual therapy with clopidogrel and an OAC immediately after PCI as an alternative to triple therapy in those for whom the bleeding risk overweighs the ischemic risk, and dual therapy (aspirin is only used in the periprocedural phase after PCI) is recommended as a default strategy in the North American guidelines.^475^

##### 5.3.5.2 Choice and Duration of Antithrombotic Therapy

Based on the increased evidence in this field as described above, this guideline provides a flow chart (Figure 14) and Table 41 for antithrombic management in AF patients with concomitant IHD.^260^, ^261^, ^262^, ^263^, ^461^, ^463^, ^464^, ^465^, ^466^, ^467^, ^468^, ^469^, ^470^, ^471^, ^472^, ^473^, ^476^, ^477^, ^478^, ^479^, ^480^, ^481^ In AF patients receiving an OAC who undergo PCI, triple therapy with aspirin and a P2Y_12_ inhibitor should be initiated and continued in the periprocedural phase (class I/evidence level C).^21^, ^330^, ^450^, ^461^, ^474^, ^475^ At this time, the administration of a proton pump inhibitor is recommended (class I/evidence level B).^21^, ^330^, ^460^, ^476^, ^477^, ^478^ The P2Y_12_ inhibitors, ticagrelor and prasugrel (loading dose, 60 mg; maintenance dose, 10 mg in other countries) have been suggested to have a high bleeding risk.^482^, ^483^, ^484^ However, the approved dose of prasugrel was reduced (loading dose, 20 mg; maintenance dose, 3.75 mg) because East Asian individuals have a higher bleeding risk than Western individuals. Therefore, this guideline allows for the use of prasugrel when considering its use in clinical practice in Japan. While balancing between the thromboembolic‐vs.‐bleeding risk, a default strategy is de‐escalation from triple therapy to dual therapy with an OAC and P2Y_12_ inhibitor by ceasing aspirin within 2 weeks after PCI.^330^, ^461^, ^475^ More than half of the patients enrolled in the 4 DOAC AF PCI trials were compromised by acute coronary syndrome (ACS),^466^, ^467^, ^468^, ^469^ and a subanalysis revealed a consistent benefit of dual therapy with a DOAC and clopidogrel over a warfarin‐based triple therapy, regardless of ACS or stable CAD. Therefore, dual therapy is recommended as a default strategy even in patients with ACS. Nevertheless, the subanalysis of the AUGUSTUS trial demonstrated that a stent thrombosis at 1 month was numerically but not significantly lower in the triple therapy group with concomitant use of aspirin than in the dual therapy group after early cessation of aspirin in the periprocedural phase after PCI,^485^ implying the need for caution in patients with a very high risk of stent‐driven ischemic events. Therefore, triple therapy for longer than 1 month and up to 3 months by physicians’ discretion is acceptable in patients with a very high stent‐driven ischemic risk, as shown in Table 42.^330^, ^475^ In contrast, triple therapy for longer than 1 month cannot be recommended in patients with a high bleeding risk (class III/evidence level B).^330^, ^461^, ^475^

**Table 41:** Recommendations and Levels of Evidence for Antithrombic Therapy in Atrial Fibrillation Concomitant With Ischemic Heart Disease

	COR	LOE	GOR (MINDS)	LOE (MINDS)
Triple therapy with an OAC, aspirin, and P2Y_12_ inhibitor in the periprocedural phase after coronary stent implantation	I	C	B	IVa
Concomitant use of a proton pump inhibitor during the administration of antiplatelet drugs^476^, ^477^, ^478^	I	B	B	II
Dual therapy with an OAC and P2Y_12_ inhibitor beyond the periprocedural phase (2 weeks) after coronary stent implantation^463^, ^466^, ^467^, ^468^, ^469^, ^472^, ^473^	I	A	A	I
OAC*^1^ alone in the chronic phase (beyond 1 year) in patients undergoing stent implantation/CABG and CAD patients who did not undergo PCI^464^, ^465^, ^470^	I	B	B	II
DOAC rather than warfarin in patients undergoing stent implantation^260^, ^261^, ^262^, ^263^, ^468^, ^479^, ^480^, ^481^	I	A	A	I
Lowest established dose of a DOAC effective for stroke prevention when a DOAC is used in combination with an antiplatelet drug^466^, ^467^, ^468^, ^469^	IIa	A	B	II
When warfarin is used in combination with an antiplatelet drug, warfarin should be carefully regulated with the target INR in the lower part of the recommended target INR (2.0–2.5)*^2^ and time in the therapeutic range >65%^466^, ^467^, ^468^, ^469^, ^471^	IIb	C	C1	IVb
Triple therapy should not be continued for longer than 1 month in patients with a very high bleeding risk^461^, ^463^, ^464^, ^465^, ^466^, ^467^, ^468^, ^469^, ^470^	III	B	B	II

*^1^Only rivaroxaban has established evidence. *^2^INR 1.6–2.5 in patients aged ≥70 years.

Abbreviation: CABG, coronary artery bypass grafting; CAD, coronary artery disease; COR, class of recommendation; GOR, grade of recommendation; INR, international normalized ratio; LOE, level of evidence; MINDS, Medical Information Network Distribution Service; OAC, oral anticoagulant; PCI, percutaneous coronary intervention.

**Table 42:** Characteristics of Patients With High Thromboembolic Risk

Stent thrombosis‐driven ischemic event risk factor
•First‐generation drug‐eluting stent
•At least 3 stents implanted
•At least 3 lesions treated
•Bifurcation with 2 stents implanted
•Total stent length >60 mm
•Stenting of a saphenous vein graft
•Prior stent thrombosis on adequate antiplatelet therapy
•Stenting of small vessels
Thromboembolic risk factors
•Current smoker
•History of PCI/CABG
•PAD
•Heart failure
•Old age
•Anemia
Common risk factors in stent thrombosis‐driven ischemic and thromboembolic events
•ACS
•Chronic total occlusion
•Concomitant diabetes mellitus
•CKD (creatine clearance <60 mL/min)

ACS, acute coronary syndrome; CABG, coronary artery bypass grafting; CKD, chronic kidney disease; PAD, peripheral artery disease; PCI, percutaneous coronary intervention.

(Modified from JCS, 2020.^486^)

In the chronic phase beyond 1 year after PCI, an OAC alone can be recommended as a default strategy (class I/evidence level B), which is derived from the results of the AFIR trial, OAC‐ALONE trial, and the nationwide Danish cohort study.^330^, ^461^, ^464^, ^465^, ^470^, ^474^, ^475^ In limited numbers of patients with a very high thromboembolic risk, dual therapy with an OAC and P2Y_12_ inhibitor (or aspirin) for longer than 1 year can be considered,^21^, ^330^, ^461^, ^474^, ^475^ but dual therapy should be considered to be shortened at 6 months in patients with a high bleeding risk.^461^, ^474^, ^475^

The 4 phase III DOAC trials in AF patients included stable CAD in one‐third of the total patients and ACS in 15–20%,^260^, ^261^, ^262^, ^263^ and reported the efficacy and safety of DOACs over warfarin even in those patients. The persistent benefit of DOACs over warfarin was observed in a database analysis, meta‐analysis, and registry in Japan.^479^, ^480^, ^481^ Furthermore, the AUGUSTUS trial^468^ indicated that apixaban had a similar efficacy and in particular, a bleeding risk, superior to warfarin. For these reasons, a DOAC should be used unless the patient has a contraindication (class I/evidence level A).^461^, ^474^, ^475^ Regarding the dose of each DOAC in the combined use as antiplatelet therapy, the lowest dose established for stroke prevention in the phase III DOAC AF trials should be used (class IIa/evidence level A).^466^, ^467^, ^468^, ^469^ When warfarin is used in combination with antiplatelet therapy, the international normalized ratio (INR) should be maintained in the lower part of the recommended target range and the time in the therapeutic range should be > 65% (class IIb/evidence level C). This is because significant increases in bleeding events were evident with VKA‐based triple therapy with a target INR of 2.0–3.0, as reported in the 4 DOAC AF PCI trials^466^, ^467^, ^468^, ^469^ and the ISAR‐TRIPLE trial.^471^

#### 5.3.6 Management of Hemorrhagic Complications: Hemostatic Procedure, Antidotes, etc.

**Figure 15:**
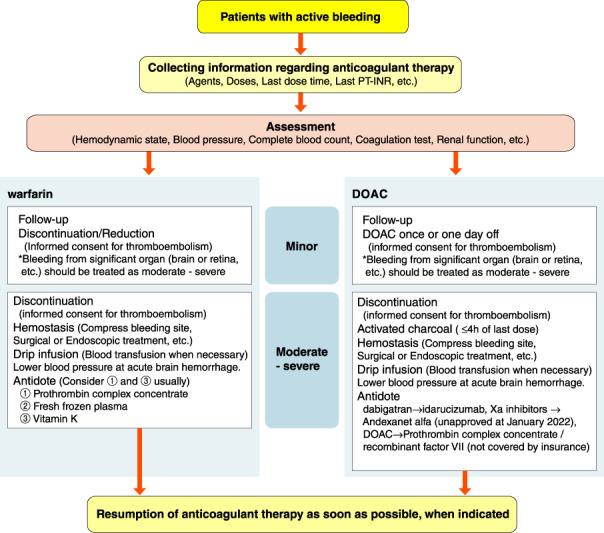
Treatment for active bleeding during anticoagulant therapy in patients with atrial fibrillation. DOAC, direct oral anticoagulant; PT‐INR, prothrombin time‐international normalized ratio.

An observational study with 4,009 patients undergoing antithrombotic therapy in Japan showed that the incidence of major bleeding with single antiplatelet, multiple antiplatelet agents, warfarin, and warfarin plus antiplatelet agents was 1.2%/year, 2.0%/year, 2.1%/year, and 3.6%/year, and the incidence of intracranial hemorrhage was 0.3%/year, 0.6%/year, 0.6%/year, and 1.0%/year, respectively.^327^ We need to recognize that serious bleeding can occur at a certain frequency during antithrombotic therapy, and that the risk increases with the concomitant use of antithrombotic agents. We also need to know how to manage hemorrhagic complications (Figure 15, Table 43).

**Table 43:** Recommendations and Levels of Evidence for the Management of Active Bleeding

	COR	LOE	GOR (MINDS)	LOE (MINDS)
General first aid	I	C	B	VI
Adequate blood pressure reduction in hemorrhagic stroke	I	A	A	I
Discontinuation/reduction of warfarin and administration of vitamin K depending on the severity of hemorrhagic complications during warfarin therapy	I	C	B	III
Administration of PCC when urgent reversal of anticoagulant effect by warfarin is needed	I	A	B	II
Administration of FFP when urgent reversal of anticoagulant effect by warfarin is needed	I	B	B	III
Administration of both PCC and vitamin K to avoid re‐elevation of PT‐INR when reversal of anticoagulant effect by warfarin is needed	I	B	B	III
Discontinuation/reduction of heparin or administration of protamine sulfate depending on the severity of hemorrhagic complications during heparin therapy	I	C	B	III
Resumption of anticoagulant therapy as soon as possible when it is indicated after hemostasis	I	C	B	V
Administration of idarucizumab when urgent reversal of anticoagulant effect by dabigatran is needed	I	B	B	III
Discontinuation of DOAC according to the severity of hemorrhagic complications during DOAC therapy, and promotion of excretion by appropriate infusion of diuretics	IIa	C	B	VI
Administration of Andexanet alfa when urgent reversal of anticoagulant effect by factor Xa inhibitors is needed (unapproved at March 2020)	IIa	C	B	III
Administration of PCC when urgent reversal of anticoagulant effect by warfarin is needed at PT‐INR <2.0	IIa	C	B	V
Administration of recombinant factor VII when urgent reversal of anticoagulant effect by warfarin is needed (not covered by insurance)	IIb	C	C1	V
Hemodialysis during dabigatran therapy	IIb	C	C1	V
Administration of PCC when urgent reversal of anticoagulant effect by DOAC is needed (not covered by insurance)	IIb	C	C1	V
Gastric lavage and administration of activated charcoal for bleeding early after taking DOAC	IIb	C	C1	V

Abbreviation: COR, class of recommendation; DOAC, direct oral anticoagulant; FFP, fresh frozen plasma; GOR, grade of recommendation; INR, international normalized ratio; LOE, level of evidence; MINDS, Medical Information Network Distribution Service; PCC, prothrombin complex concentrate; PT, prothrombin time.

In the case of mild bleeding, we should consider correct continuation of antithrombotic therapy rather than discontinuation without careful consideration. For moderate‐to‐severe bleeding, it is recommended to discontinue the antithrombotic agents, stop the bleeding, stabilize circulatory dynamics with appropriate drip infusion, and lower the blood pressure during intracerebral hemorrhage or subarachnoid hemorrhage (recommended class I).^487^, ^488^, ^489^ Administration of prothrombin complex concentrate (PCC) and vitamin K (recommended class I), fresh frozen plasma (FFP, recommended class I) and recombinant factor VII preparations (not covered by insurance, recommended class IIb) are recommended for the purpose of suppressing bleeding tendency during warfarin therapy and acute severe bleeding, or for urgent surgery/procedures where serious bleeding is anticipated.^490^, ^491^, ^492^, ^493^, ^494^, ^495^, ^496^

The administration of both PCC and vitamin K has the fastest and surest effect.^497^, ^498^, ^499^ Randomized controlled trials of PCC + vitamin K versus FFP + vitamin K performed in patients on warfarin treatment who had severe bleeding or suddenly needed invasive medical treatment showed a non‐inferiority of the hemostatic effect of PCC + vitamin K against FFP + vitamin K.^497^, ^498^ In addition, PCC had a shorter administration time, a shorter time to hemostasis, and a smaller volume than that of FFP. From the standpoint of pasteurization and nanofiltration, PCC is superior to FFP for safety.

The administration dose of the PCC is determined according to the INR value and body weight. PCC of 25 IU/kg (max. dose of 2,500 IU) for INR of ≥2.0 and <4.0, 35 IU/kg (max. dose of 3,500 IU) for INR of ≥4.0 and <6.0, or 50 IU/kg (max. dose of 5,000 IU) for INR ≥6.0 should be administered. Usage and dosage for patients with INR <2.0 have not been officially recognized.

However, a pharmacometric simulation model analysis showing the relationship of INR and coagulation factor activity using the PCC Phase III study data^497^, ^498^, ^499^ showed that PCC doses of 25 IU/kg, 20 IU/kg, and 15 IU/kg were needed at INRs 3.1, 1.9, and 1.6, respectively, to achieve both coagulant factor II and X activities of ≥50% in more than 80% of patients at 30 min after administration of PCC.^500^ Therefore, if urgent correction is required in a patient with INR <2.0, a dose of 20 IU/kg for INR >1.6 and 15 IU/kg for INR ≤ 1.6 might be considered.

In addition, studies in a small number of cases have been reported suggesting the usefulness of administering 15–25 IU/kg of PCC for INR <2.0.^501^, ^502^ For correction during heparin therapy, dilute protamine sulfate is slowly infused.^503^

The incidence of hemorrhagic complications during DOAC therapy is equivalent or less than that of warfarin. For bleeding, in addition to hemostasis treatment, we should consider discontinuation of DOACs according to the severity of hemorrhagic complications during DOAC therapy and promotion of diuresis excretion by appropriate infusion. Administration of PCC (not covered by insurance, recommended class IIb) and that of recombinant factor VII preparations (not covered by insurance, recommended class IIb) can be considered as measures against bleeding during DOAC therapy, but they have not been examined sufficiently. Dabigatran may be removed by hemodialysis because it has a low binding rate to proteins in the blood. For bleeding early after oral administration of each DOAC, gastric lavage or oral administration of activated charcoal may be considered to suppress the increase in blood concentration by suppressing absorption from the digestive tract.^504^

Idarucizumab, a specific neutralizing antibody, is used during dabigatran therapy. To suppress the bleeding tendency during dabigatran therapy with acute severe bleeding or for urgent surgery/procedures in which significant bleeding is anticipated, idarucizumab is administered within 24 h after the last oral dose of dabigatran.^332^ Because a high blood concentration may be sustained in patients with renal dysfunction or oral administration of P‐glycoprotein inhibitors, we may consider administration of idarucizumab for up to 48 h in those patients. Regardless of the dose or time after oral administration, 5 g idarucizumab (two 2.5 g vials) is administered. The anticoagulant effect of dabigatran is rapidly and completely neutralized within 1 min after the administration and the neutralizing effect is sustained for 24 h.

Dabigatran can be administered 24 h after the administration of idarucizumab, and other anticoagulants can be administered within 24 h. Neutralizing the anticoagulant effect of dabigatran may induce a hypercoagulable state prior to the introduction of dabigatran, but idarucizumab itself has no effect on coagulation or fibrinolysis. Therefore, if cerebral infarction develops in a patient on dabigatran treatment, there is an option to administer intravenous recombinant tissue plasminogen activator (rt‐PA) after neutralizing the anticoagulant effect of dabigatran by administration of idarucizumab.^505^

If it is necessary to correct the effects of factor Xa inhibitors, administration of an antidote, Andexanet alfa (unapproved at January 2022) or ciraparantag (unapproved at January 2022) may be considered.^457^, ^506^ Andexanet alfa is a decoy protein of coagulation factor Xa, and the corrective effect can be sustained by intravenous infusion after a fixed amount of intravenous injection.^457^ The dose varies depending on the type of factor Xa inhibitor. In the USA, it was approved as an antidote for rivaroxaban and apixaban in May 2018. In Japan, it is under development as an antidote for all factor Xa inhibitors. Ciraparantag is a low molecular weight compound that has attracted attention because it is suggested to have a neutralizing effect on not only factor Xa inhibitors but also thrombin inhibitors and heparin, but its development has been delayed.^506^

If resumption of anticoagulant therapy is indicated, considering the source of hemorrhage and the state of hemostasis after hemostatic treatment, surgery, or other invasive procedure, it should be resumed as soon as possible to prevent thromboembolism.

### 5.4 Rate Control Therapy

Treatments for atrial fibrillation (AF) include pharmacotherapy and catheter ablation. Pharmacotherapy is a priority, but may be considered positively in patients for whom catheter ablation is indicated. Anticoagulant therapy should be firstly considered as pharmacotherapy for AF. As the next step, rhythm control therapy and rate control therapy were previously recommended as similar, but in recent years, rate control therapy has become a higher priority than rhythm control therapy. Clinical trials such as AFFIRM^507^ and RACE^508^ conducted in Western countries, and J‐RHYTHM^509^ in Japan showed that rhythm control therapy and rate control therapy did not differ in hospitalization for all‐cause death, cardiovascular death, and aggravation of heart failure (HF). Similar results were obtained in AF‐CHF^229^ with AF associated with HF.

Based on this evidence, in the European guideline,^21^ easy rate control therapy is ranked high, but rhythm control therapy is considered in patients with strong subjective symptoms or patients with impaired quality of life (QOL).

**Figure 16:**
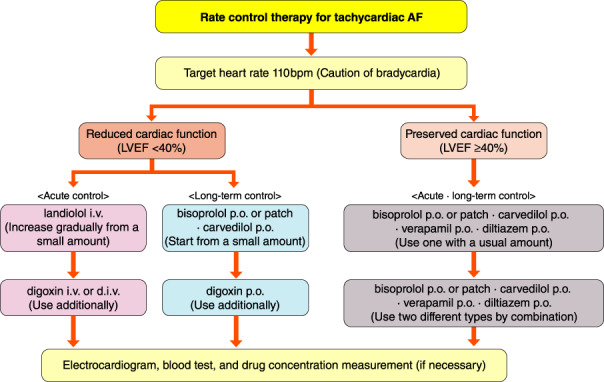
Treatment flowchart of rate control therapy for tachycardiac AF. AF, atrial fibrillation; LVEF, left ventricular ejection fraction.

Drugs used to control heart rate include *β*‐blockers, digitalis, non‐dihydropyridine Ca^2+^ channel antagonists, and the antiarrhythmic drug amiodarone. Among them, *β*‐blockers have added value, such as a protective effect on the myocardium and improvement of life prognosis, and can be expected to improve symptoms by relaxing sympathetic tone. Therefore, *β*‐blockers are widely used, compared with other drugs, in Europe, the USA, and Japan^509^, ^510^ (Table 44, Figure 16).

**Table 44:** Recommendations and Levels of Evidence for Rate Control Therapy Using Drugs for AF

	COR	LOE	GOR (MINDS)	LOE (MINDS)
*β*‐blockers				
Heart rate control using long‐term oral drugs (bisoprolol, carvedilol) for tachycardiac AF with reduced cardiac function (LVEF <40%, ≥25%)	I	A	A	I
Heart rate control using long‐term oral drugs (bisoprolol, carvedilol, verapamil, diltiazem) for tachycardiac AF in patient with preserved (LVEF ≥40%) cardiac function	I	B	A	I
Long‐term oral/patch (bisoprolol, carvedilol) administration to improve prognosis for symptomatic tachycardiac AF	IIa	B	A	I
Heart rate control using acute intravenous landiolol infusion for tachycardiac AF with reduced cardiac function (LVEF <40%, ≥25%) (starting with a small dose and gradually increasing while observing hemodynamics)	IIa	B	B	II
Administration to asymptomatic AF patients	IIb	C	D	VI
Administration to patients with AF without tachycardia	III	B	D	VI
digitalis				
Additional administration with *β*‐blocker for the aim of controlling heart rate in acute phase for tachycardiac AF with reduced cardiac function	IIa	B	B	III
Long‐term heart rate control for patients with tachycardiac AF	III	C	D	II
Non‐dihydropyridine calcium antagonists				
Heart rate control for tachycardiac AF with preserved cardiac function	I	B	A	I
Heart rate control with intravenous/oral drugs (verapamil, diltiazem) for tachycardiac AF with reduced cardiac function	III	C	D	V
amiodarone				
Heart rate control using acute intravenous drug for tachycardiac AF with reduced cardiac function	IIb	C	C1	IVb

AF, atrial fibrillation; COR, class of recommendation; GOR, grade of recommendation; LOE, level of evidence; LVEF, left ventricular ejection fraction; MINDS, Medical Information Network Distribution Service.

#### 5.4.1 Acute Control

Intravenous drugs are mainly used for urgently controlling the heart rate during the acute phase. *β*‐blockers, digitalis, and amiodarone are used.

Intravenous *β*‐blockers include landiolol, esmolol, and propranolol, but usage of the ultra‐short‐acting *β*_1_‐blocker landiolol is high in Japan. JL‐KNIGHT,^511^ which was performed in patients with tachycardiac AF/atrial flutter after thoracic surgery, showed that the heart rate inhibitory effect and recovery of sinus rhythm were superior to that of diltiazem. J‐Land,^512^ which was performed in patients with tachycardiac AF and impaired cardiac function (complicated with HF), showed that heart rate‐suppressing effect of landiolol was superior to that of digoxin, and there was no difference in the occurrence of side effects. It was shown that the effectiveness of landiolol as a heart rate regulator can be expected for tachycardiac AF associated with HF, but it has been shown to be less effective in patients with extremely impaired cardiac function.^513^, ^514^ Moreover, the tachycardia suppression effect on atrial flutter or atrial tachycardia cannot be expected as much as for AF.^515^

When landiolol is used in patients with low cardiac function, the dose is gradually increased from a small dosage (1 *μ*g/kg/min; max. dosage 10 *μ*g/kg/min). If the effect is insufficient, add digitalis. Intravenous non‐dihydropyridine Ca^2+^ channel antagonists (diltiazem and verapamil) are contraindicated for tachycardiac AF associated with low cardiac function with left ventricular ejection fraction (LVEF) <40%.

Amiodarone may be used when attempting to control the heart rate of tachycardiac AF while considering defibrillation (not covered by insurance).

#### 5.4.2 Long‐Term Control

##### 5.4.2.1 *β*‐Blockers

The first‐line treatment is a *β*‐blocker. In a clinical study that verified the prognostic effect of *β*‐blockers and digitalis, *β*‐blockers improved the prognosis of patients regardless of the degree of cardiac function, but digitalis did not show such an effect.^516^

Although there are many types of oral *β*‐blockers, those without intrinsic sympathomimetic action (ISA: bisoprolol, carvedilol, metoprolol, etc.) are used for rate control therapy. MAIN‐AF^517^ for bisoprolol and AF carvedilol^518^ for carvedilol demonstrated a heart rate‐reducing effect in patients with persistent or chronic AF. A bisoprolol patch was also recently approved by BISONO‐AF^519^ because of its heart rate‐reducing effect in patients with AF. In a comparison of bisoprolol and carvedilol, bisoprolol, which has a high cardiac (*β*_1_) selectivity, has a stronger heart rate inhibitory effect. Therefore, when used in elderly patients with HF, it is necessary to pay attention to severe bradycardia as a side effect, as shown in CIBIS‐ELD.^520^

In a meta‐analysis of an old clinical trial that verified the efficacy of *β*‐blockers in patients with HF,^521^ it was reported that the effect of *β*‐blockers on prognosis was not observed in patients with AF. However, recent clinical studies reported thereafter that *β*‐blockers have a prognostic effect on AF with HF.^522^, ^523^, ^524^ The current usage of *β*‐blockers seems to have a prognostic effect on AF associated with HF.

##### 5.4.2.2 Non‐Dihydropyridine Ca^2+^ Channel Antagonists

Verapamil and diltiazem, which are non‐dihydropyridine Ca^2+^ channel antagonists, are used in patients with tachycardiac AF because they have not only antihypertensive effects but also relatively strong bradycardiac effects due to atrioventricular conduction inhibition. However, because they also have a negative inotropic effect, their use is limited to cases where cardiac function is maintained. Negative inotropic effects are more likely to occur in patients with impaired cardiac function, and verapamil is stronger than diltiazem.

In the guidelines in Japan and Europe,^21^, ^250^ non‐dihydropyridine Ca^2+^ channel antagonists are contraindicated in patients with HF and impaired cardiac function (LVEF <40%). Therefore, when selecting a non‐dihydropyridine Ca^2+^ channel antagonists, it is necessary to check cardiac function by echocardiography or blood test (BNP level).

##### 5.4.2.3 Digitalis

Digoxin or methyldigoxin is used. Digitalis also has a cardiotonic action, and is often used in patients with tachycardiac AF with impaired cardiac function. However, as shown in the European guidelines,^21^ it is not used as a first‐line drug, but as a second‐line drug. Digitalis has a heart rate‐reducing effect at rest, but a weaker effect on exercise. The J‐RHYTHM Registry subanalysis showed that digitalis alone had no effect on the prognosis,^525^ but it has been shown in the USA and Europe that mortality increases with long‐term use in patients with AF.^516^, ^526^, ^527^ Therefore, long‐term use should be avoided as much as possible.

Because both digoxin and methyldigoxin are drugs excreted by the kidney, administration in patients with impaired renal function may result in digitalis toxicity. It is necessary to periodically measure the blood concentration and adjust the dosage for the optimum concentration.

#### 5.4.3 Target Heart Rate in Rate Control Therapy

The target heart rate is set in patients with persistent or permanent AF. Traditionally, the heart rate of AF was stated as <80 beats/min at rest and <110 (115) beast/min during exercise. In RACE II,^528^ it was verified in patients with permanent AF whether or not strict heart rate control should be performed. As a result, there is no difference in event rate between strict rate control therapy (resting heart rate <80 beats/min) and lenient rate control therapy (resting heart rate <110 beats/min). Therefore, it is important to individualize the control of heart rate to reduce subjective symptoms and signs of HF.

European guidelines^21^ state that the AF heart rate at rest should be <110 beats/min. This is not supported by clear evidence, and it is important to adjust the heart rate appropriately while considering subjective symptoms and QOL in each patient.

### 5.5 Rhythm Control Therapy

AF, cardiac output is decreased and thrombus formation is enhanced due to the loss of effective atrial contraction. Additionally, irregular beats and tachycardia cause various clinical symptoms such as palpitation, chest discomfort, etc. Therefore, recovery of sinus rhythm itself has great benefit. However, clinical procedures aimed at sinus rhythm recovery, including pharmacological and non‐pharmacological approaches, have not only their benefits but also various risks related to these therapies. The adequacy of treatment must be judged in the balance between risk and benefit.

The mega‐trials that compared rhythm control (sinus rhythm maintenance) and rate control (heart rate control) failed to exhibit any difference in mortality.^229^, ^507^ In the J‐RHYTHM trial, the superiority of rhythm control was demonstrated, at least for paroxysmal AF, by using specific endpoints including quality of life (QOL).^509^ In those trials, rhythm therapy was mainly pharmacological. However, a recent trial that compared rhythm control and rate control by using catheter ablation as the main therapy for rhythm control, demonstrated recovery of cardiac function in cases of left ventricular dysfunction.^222^ Therefore, the value and therapeutic procedures of rhythm control must be reevaluated.

Because the same antiarrhythmic agents are commonly selected for recovery of sinus rhythm (defibrillation) and prevention of AF (sinus rhythm maintenance), the 2008 guideline^530^ unified them, but the 2013 guideline^2^ listed them separately. In this guideline, by considering various therapeutic options, including catheter ablation, especially for preventive therapy, these 2 concepts are described separately. In the selection list of antiarrhythmic agents, the usage record in Japan was weighted. In the selection of antiarrhythmic agents, safety must be the most important issue because pharmacological rhythm control should be understood as therapy for QOL improvement instead of decrease in mortality.

#### 5.5.1 Interruption of AF (Defibrillation)

At the time of AF interruption (defibrillation), great attention must be paid to the possibility of systemic embolism by the pumping out intra‐atrial thrombus. It is important to confirm the absence of intra‐atrial thrombus by transesophageal echocardiography or to perform appropriate anticoagulation therapy, especially in patients in whom the possibility of more than 48‐h continuation of AF cannot be denied. Excepting emergencies, greatest attention must be paid to prevention of systemic embolism.

**Figure 17:**
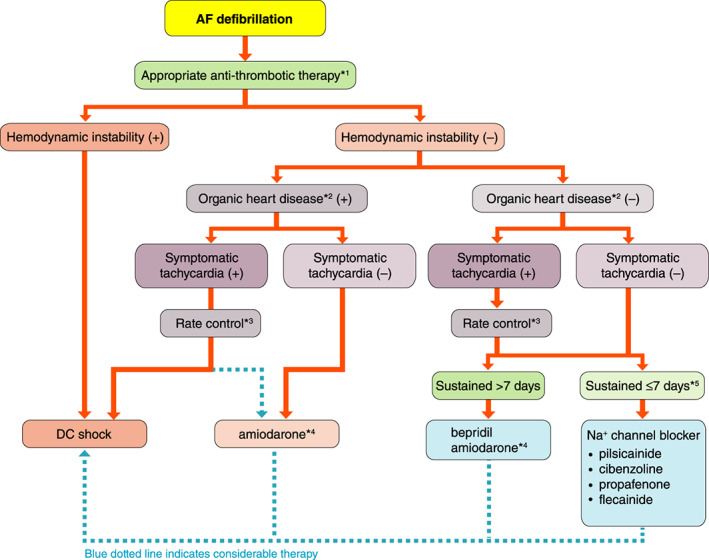
Flow‐chart for defibrillation of AF. *^1^The possibility of 48‐h continuation of AF cannot be denied; essential to confirm the absence of intra‐atrial thrombus by transesophageal echocardiography or to perform appropriate anticoagulation therapy for 3 weeks (see **“Chapter V. 3. Anticoagulation Therapy”** for details). *^2^Hypertrophic heart, heart failure, ischemic heart disease. *^3^Even without hemodynamic instability, rate control therapy might be combined in cases of symptomatic tachycardia (see **“Chapter V. 4. Rate Control Therapy”** for details). *^4^Insurance coverage for Amiodarone is approved only for patients with AF with hypertrophic cardiomyopathy or heart failure in Japan. *^5^Rhythm control therapy should be performed within 48 h of AF onset by considering efficacy of therapy as well as prevention of systemic embolism. AF, atrial fibrillation; DC, direct‐current.

In the case of emergency in which hemodynamic stability cannot be expected, QRS‐triggered direct‐current defibrillation with ≥100 J electrical shock will be rapid and effective under appropriate anesthesia and respiratory control (Figure 17, Table 45).^2^, ^21^, ^530^

**Table 45:** Recommendations and Levels of Evidence for AF Defibrillation

	COR	LOE	GOR (MINDS)	LOE (MINDS)
R‐wave triggered direct‐current defibrillation for drug‐refractory AF causing hemodynamic instability or AF causing sustained cardiac ischemia, symptomatic hypotension, worsening heart failure, or life threatening condition^2^, ^21^, ^530^	I	C	C1	IVb
AF with ventricular pre‐excitation causing hemodynamically unstable tachycardia^2^, ^21^, ^530^	I	C	C1	IVb
Interruption of drug‐refractory AF without anticoagulation therapy within 48 h of AF onset^2^, ^530^	IIa	C	C1	IVb
Case of AF in whom the possibility of 48 h continuation cannot be denied but the absence of intra‐atrial thrombus is confirmed by transesophageal echocardiography, or appropriate anticoagulation therapy has been continued more than 3 weeks at the time of defibrillation^2^, ^21^, ^530^	IIa	C	C1	IVb
Direct‐current defibrillation under the use of antiarrhythmic agent for recurrent AF even after direct‐current defibrillation^2^, ^21^, ^530^	IIa	C	C1	IVb
Case of sustained AF even after normalization of hyperthyroidism or postoperative state of cardiac surgery, in whom antiarrhythmic agent is ineffective or inapplicable^2^, ^530^	IIa	C	C1	IVb
Elective direct‐current defibrillation for asymptomatic AF with continuation <1 year and without obvious left atrial enlargement^2^, ^21^, ^530^	IIb	C	C1	IVb
Elective direct‐current defibrillation for repetitive AF in relatively short period even after preventive antiarrhythmic drug therapy and repeated direct‐current defibrillation^2^, ^21^, ^530^	IIb	C	C1	IVb
Direct‐current defibrillation for AF based on digitalis intoxication or hypokalemia^2^, ^530^	III	C	C2	IVb
Direct‐current defibrillation for AF complicated with obvious high‐degree AV block and/or sick sinus syndrome without backup pacing therapy^2^, ^530^	III	C	C2	IVb
Direct‐current defibrillation for hemodynamically stable persistent AF lasting longer than 48 h without taking standard antithrombotic strategy* ^2^, ^530^	III	C	C2	IVb

*Confirmation of absence of intra‐atrial thrombus with transesophageal echocardiography or continuous appropriate anticoagulation longer than 3 weeks.

AF, atrial fibrillation; AV, atrioventricular; COR, class of recommendation; GOR, grade of recommendation; LOE, level of evidence; MINDS, Medical Information Network Distribution Service.

In addition to emergency treatment, direct‐current defibrillation should be applied when patients prefer it, when AF is refractory to pharmacological defibrillation, and when use of antiarrhythmic agents is considered more dangerous than direct‐current defibrillation. Especially in cases of AF complicated with organic heart disease such as hypertrophic heart, heart failure, and ischemic heart disease, the efficacy of antiarrhythmic agents will be suppressed and the risk of proarrhythmia will be increased. In cases of heart failure, amiodarone might be used for the purpose of defibrillation, but it will take longer to evaluate its efficacy, so its use should be limited to patients in a stable condition. In case of symptomatic tachycardia, appropriate rate control is necessary and direct‐current defibrillation is recommended preferentially, versus pharmacological therapy, because of its immediate effect (Figure 17). However, because AF may recur even after successful defibrillation, preventive use of antiarrhythmic agents might be needed in some cases. When combined defibrillation with antiarrhythmic agent and direct‐current shock fail to achieve sinus rhythm recovery, a re‐trial of defibrillation after appropriate therapy for organic heart disease or catheter ablation should be considered.

Safety is the most important issue in the application of pharmacological defibrillation. Therefore, antiarrhythmic agents with negative inotropic effect or QT prolongation can be applied only in cases without organic heart diseases such as hypertrophic heart, heart failure, or ischemic heart disease. When pharmacological defibrillation is to be attempted in such cases, highly professional and careful decision making is necessary. In this edition of the guideline, use or limitation of Na^+^ channel blockers is highlighted and described in relation to the clinical type of AF.

##### 5.5.1.1 Paroxysmal Atrial Fibrillation

Paroxysmal AF is defined as self‐interrupting AF within 7 days regardless of therapeutic intervention. Artificial defibrillation is recommended within 48 h from the onset of AF for the purpose of removing serious symptoms and/or reduction of risk for systemic embolism. It is known that the efficacy of Na^+^ channel blockers is higher for AF with shorter duration in cases without organic heart disease; therefore, the use of Na^+^ channel blocker is recommended for AF within 7 days of onset. However, appropriate antithrombotic strategy should be attempted for defibrillation because the risk for systemic embolism will increase with time. When an immediate effect is required, intravenous administration of an antiarrhythmic agent is more effective. However, there is also an administration method called “pill‐in‐the‐pocket” in which the patients take the drug orally once, depending on their own decision at the time of the AF attack.^531^ Standard antiarrhythmic agents for this purpose are pilsicainide 100 mg,^532^ flecainide 100 mg,^110^, ^533^, ^534^ propafenone 150 mg,^533^, ^534^ and cibenzoline 100 mg.^531^, ^532^ It is understood that digitalis^537^, ^538^, ^539^ and/or sotalol^540^, ^541^ are ineffective for this purpose.

Na^+^ channel blocker leads to AF interruption by decreasing the activation frequency during AF through conduction delay and/or conduction block in the atrial muscle.^542^ Additional mechanisms, such as prolongation of post‐repolarization refractoriness,^543^, ^544^ increase the radius of the reentrant wave front,^545^, ^546^ and conduction block between the pulmonary veins and left atrium,^547^ are also considered to relate to AF interruption. These effects are stronger with slow kinetic drugs than the others, resulting in higher efficacy for AF interruption. These drugs also have a negative inotropic effect, but they can be used as first‐line medicine in AF without clinically important organic heart diseases. This point coincides with the American–European guidelines^18^ (Figure 17, Table 46, ^1^, ^21^, ^110^, ^235^, ^530^, ^531^, ^532^, ^533^, ^534^, ^535^, ^536^, ^542^, ^543^, ^544^, ^545^, ^548^, ^549^, ^550^, ^551^, ^552^, ^553^, ^554^, ^555^, ^556^).

**Table 46:** Recommendation and Levels of Evidence for Pharmacological AF Defibrillation

	COR	LOE	GOR (MINDS)	LOE (MINDS)
Use of Na^+^ channel blocker*^1^ for AF lasting <48 h without any significant organic heart disease^2^, ^110^, ^530^, ^531^, ^532^, ^533^, ^534^, ^535^, ^536^, ^542^, ^543^, ^544^, ^545^	I	A	A	I
Use of Na^+^ channel blocker*^1^ for AF lasting for 48 h to 7 days with appropriate antithrombotic strategy*^2^ ^2^, ^530^, ^531^, ^532^, ^533^, ^535^, ^536^	IIa	C	C1	IVb
Use of bepridil for AF lasting >7 days with normal cardiac function and QT interval^2^, ^530^, ^548^, ^549^, ^550^, ^551^, ^552^, ^553^	IIa	B	B	II
Once oral use of pilsicainide, flecainide, propafenone or cibenzoline*^5^ in patients with symptomatic parosyxmal AF in whom the risks of sinus rhythm recovery*^3^ and use of Na^+^ channel blocker*^4^ have been denied^2^, ^110^, ^530^, ^531^, ^532^, ^533^, ^534^, ^535^, ^536^, ^542^, ^543^, ^544^	IIa	B	B	II
Use of amiodarone in persistent AF patients with heart failure or hypertrophic cardiomyopathy^2^, ^234^, ^530^, ^554^, ^555^	IIa	B	B	I
Combined use of aprindine with bepridil for persistent AF lasting >7 days^2^, ^530^, ^548^, ^549^, ^550^, ^551^, ^552^, ^553^	IIb	C	B	III
Use of bepridil in persistent AF patients with ventricular dysfunction with normal QT interval^2^, ^552^, ^553^	IIb	C	C1	IVb
Use of strong Na^+^ channel blockers*^1^ in AF patients with ventricular dysfunction^2^, ^530^, ^546^	III	C	C2	IVb
Use of pharmacological defibrillation without pacing backup in patients in complicated with the risk of sinus rhythm recovery*^3^ ^2^, ^530^	III	C	C2	IVb
Use of Na^+^ channel blockers*^1^ in patients with Brugada syndrome^2^, ^530^	III	C	C2	IVb
Use of bepridil in persistent AF patients with QT prolongation^2^, ^530^, ^552^, ^553^	III	C	C2	IVb
Pharmacological defibrillation without appropriate antithrombotic strategy in AF patients whom the possibility of 48‐h continuation cannot be denied^2^, ^21^, ^530^	III	C	C2	IVb
Use of digoxin, sotalol as single use for the purpose of pharmacological defibrillation^2^, ^530^, ^537^, ^538^, ^556^	III	B	C2	II

*^1^pilsicainide, cibenzoline, propafenone, flecainide. *^2^Confirmation of absence of intra‐atrial thrombus with transesophageal echocardiography or continuous appropriate anticoagulation >3 weeks. *^3^Possible appearance of sick sinus syndrome, AV block or bundle branch block after interruption of AF. *^4^Brugada syndrome or organic heart disease that might be affected by negative‐inotropic effect; history of atrial flutter. *^5^Efficacy and safety of the drug must be confirmed by doctor more than once.

AF, atrial fibrillation; AV, atrioventricular; COR, class of recommendation; GOR, grade of recommendation; LOE, level of evidence; MINDS, Medical Information Network Distribution Service.

Various strong Na^+^ channel blockers are approved for use in Japan. Pilsicainide is a pure Na^+^ channel blocker developed in Japan. Single oral use of pilsicainide 150 mg interrupted 45% of cases of AF lasting <7 days in the PSTAF trial.^532^ Cibenzoline blocks several K^+^ channels as well as the Na^+^ channel,^557^, ^558^ and a single oral dose of cibenzoline 200 mg interrupted 75–85% of AF lasting <48 h.^535^, ^536^ The efficacy of propafenone and flecainide have been widely reported, even in Western countries, with defibrillation rates (intravenous 2 mg/kg/20 min) of 72% and 90%, respectively.^559^

There are various class IA antiarrhythmic agents with a K^+^ channel blocking effect and prolonging effect of the refractory period, such as disopyramide, procainamide, quinidine, aprindine and pirmenol, all available in Japan. However, they were not listed as first‐line durgs because clinical use is currently limited. The efficacy of disopyramide, procainamide, and quinidine for AF interruption has been verified, but they are not used widely in clinical practice because they have a higher chance of introducing torsade de pointes.

##### 5.5.1.2 Persistent Atrial Fibrillation

Persistent AF is defined as AF lasting >7 days, regardless of therapeutic intervention, which does not interrupt spontaneously but can be defibrillated by therapeutic intervention. When limited to pharmacological treatment, defibrillation has clinical meaning if targeting QOL improvement or preventing systemic embolism.^509^ However, in cases of persistent AF, it is common to have a long clinical course and organic heart disease as the clinical background, so the risks for systemic embolism and proarrhythmic effect may be high. When considering pharmacological therapy, specific attentions should be paid to the standard antithrombotic strategy and monitoring of side effects.

Continuation of AF promotes progression of atrial remodeling and AF tends to continue longer in the remodeled atria. AF can cause physiological change within tens of minutes and changes in ion channel density within several hours, so that the therapeutic effect may differ in the acute and subacute phases of AF. The level of remodeling can be generally evaluated by left atrial diameter, the duration of AF, fibrillation cycle length, etc.^560^ In a clinical trial of oral pilsicainide 150 mg/day, the defibrillation rate was lower in cases of AF lasting ≥2 weeks and in cases of left atrial diameter ≥45 mm.^561^ Flecainide and sotalol both failed to defibrillate AF in cases of AF lasting ≥7 days.^562^

In this guideline, drug selection is described by setting the boundary as 7 days, at which time the effect of Na^+^ channel blockers becomes weakened. Experimentally, it has been reported that multichannel blockers such as amiodarone and bepridil may show gradual improvement of electrical remodeling (i.e., reverse remodeling).^563^, ^564^, ^565^ Although these drugs are clinically consistent, with interruption of long‐lasting AF after a few weeks,^543^, ^563^ the specific mechanisms have not been clarified.

Defibrillation by oral amiodarone is regarded as the standard treatment in Europe, especially in patients with organic heart disease.^21^ Amiodarone interrupted 23% of AF lasting ≥1 week in the PIAF study,^549^ and 27.1% of AF lasting ≥72 h (vs. placebo 0.8%) in the SAFE‐T study.^550^ Amiodarone is a limited antiarrhythmic agent that can be applied especially in cases of AF and organic heart disease. In this revision of the guideline, it is positioned as an active option for patients without symptomatic tachycardia. However, we should note that insurance coverage for use of amiodarone is limited to cases of hypertrophic cardiomyopathy and heart failure in Japan.

There are some Japanese reports on bepridil. Fujiki et al. have reported that 69% of AF lasting ≥3 months could be interrupted.^566^ The J‐BAF study^551^ conducted in Japan demonstrated a 37.5% defibrillation rate by 100 mg/day and 69.0% defibrillation rate by 200 mg/day in cases of AF lasting ≥7 days, but a case of sudden death has been also reported with 200 mg/day of bepridil. Bepridil should be started from 100 mg/day orally, then possibly increased to 200 mg/day. These adjustments should be decided by a specialist under careful monitoring of QT interval and T‐wave morphology.^567^

#### 5.5.2 Prevention of Atrial Fibrillation Recurrence

Recommendation class and level of evidence for pharmacological prevention of AF recurrence are summarized in Table 47.^2^, ^21^, ^110^, ^235^, ^530^, ^531^, ^532^, ^533^, ^534^, ^535^, ^536^, ^542^, ^543^, ^544^, ^556^, ^566^, ^567^, ^568^, ^569^, ^570^ AF prevention is not necessary in cases of first‐diagnosed AF without organic heart disease, because the recurrence rate is limited in such cases. AF prevention is indicated in cases of symptomatic recurrent AF that repeats at regular intervals, but AF prevention may also be indicated in patients at a risk of systemic embolism even if they are less symptomatic. In recent years, the efficacy of catheter ablation has dramatically improved,^556^ so it should be included as a considered therapeutic option, especially in cases of symptomatic AF requiring long‐term pharmacological therapy (see “Guidelines for Non‐Pharmacological Therapy for Arrhythmia” (edited 2018) for details of indications for catheter ablation^4^).

**Table 47:** Recommendation and Levels of Evidence for Pharmacological Prevention of AF Recurrence

	COR	LOE	GOR (MINDS)	LOE (MINDS)
Use of Na^+^ channel blockers* for symptomatic recurrent AF without organic heart disease^2^, ^109^, ^530^, ^531^, ^532^, ^533^, ^534^, ^535^, ^536^, ^542^, ^543^, ^544^	I	A	A	I
Use of amiodarone for recurrent AF with heart failure or hypertrophic cardiomyopathy^2^, ^234^, ^530^, ^556^, ^566^, ^568^, ^569^	I	B	B	II
Use of antiarrhythmic agent for the purpose of AF prevention when that drug has been effective for AF interruption^2^, ^110^, ^530^, ^531^, ^532^, ^533^, ^534^, ^535^, ^536^, ^542^, ^543^, ^544^	IIa	C	C1	III
Use of amiodarone or sotalol for recurrent AF complicated with organic heart disease other than heart failure and hypertrophic cardiomyopathy (not covered by insurance)^2^, ^235^, ^530^, ^556^, ^569^	IIa	B	B	I
Use of bepridil in symptomatic recurrent AF patients without organic heart disease in whom Na^+^ channel blocker* has been ineffective^2^, ^530^, ^548^, ^549^, ^550^, ^551^, ^552^	IIa	C	C1	III
Use of Na^+^ channel blockers* for asymptomatic or less‐symptomatic recurrent AF without organic heart disease^2^, ^110^, ^530^, ^531^, ^532^, ^533^, ^534^, ^535^, ^536^, ^542^, ^543^, ^544^	IIb	C	C1	IVb
Use of Na^+^ channel blockers* for recurrent AF complicated with atrial flutter without organic heart disease^2^, ^110^, ^530^, ^531^, ^532^, ^533^, ^534^, ^535^, ^536^, ^542^, ^543^, ^544^	IIb	C	C1	IVb
Use of antiarrhythmic agent for the purpose of AF prevention in cases of first‐diagnosed AF, alcohol‐related AF, or postoperative AF^2^, ^530^, ^563^, ^564^, ^567^, ^568^	IIb	C	C1	IVb
Use of amiodarone in symptomatic recurrent AF patients without organic heart disease in whom Na^+^ channel blocker* has been ineffective (not covered by insurance)^2^, ^235^, ^530^, ^556^, ^568^, ^569^	IIb	B	A	I
Use of antiarrhythmic agent for brady/tachycardia syndrome without pacemaker implantation^2^, ^530^	III	C	C2	IVb
Use of Na^+^ channel blockers* for AF complicated with organic heart disease^2^, ^530^, ^570^	III	C	C2	IVb
Continuation of antiarrhythmic agent that is considered clinically ineffective^2^, ^530^	III	C	C2	V
Use of Na^+^ channel blockers* for AF complicated with Brugada syndrome^2^, ^530^	III	C	C2	IVb
Use of K^+^ channel blockers for AF complicated with long QT syndrome^2^, ^21^, ^530^	III	C	C2	IVb

* pilsicainide, cibenzoline, propafenone, flecainide. Use of these drugs should be avoided in cases of Brugada syndrome or organic heart disease that might be affected by negative‐inotropic effect, or with a history of atrial flutter.

AF, atrial fibrillation; COR, class of recommendation; GOR, grade of recommendation; LOE, level of evidence; MINDS, Medical Information Network Distribution Service.

Even when an antiarrhythmic drug with a highly recommended order is selected, it is necessary to pay close attention to the appearance of negative inotropic effects and proarrhythmic effects during long‐term administration, and to the emergence of extracardiac side effects specific to amiodarone.

**Figure 18:**
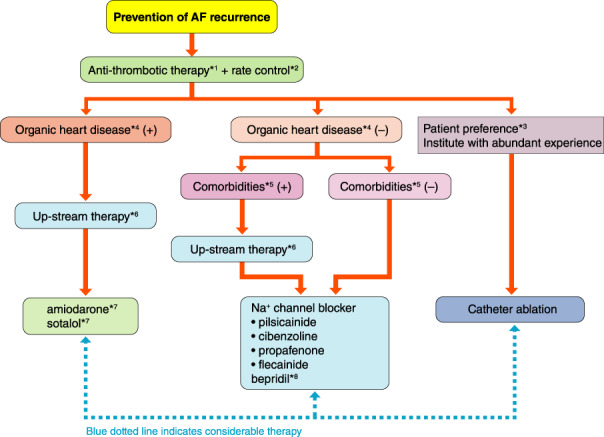
Flowchart for prevention of AF. *^1^Anticoagulation therapy might be continued depending on individual risks for embolism and efficacy of AF preventive therapy (see **“Chapter V. 3 Anticoagulation Therapy”** for details). *^2^Rate control therapy might be continued in cases of possible AF recurrence and considerable symptoms during AF (see **“Chapter V. 4 Rate Control Therapy”** for details). *^3^See the **“Guidelines for Non‐Pharmacological Therapy”**, 2018 edition 3 for details. *^4^Hypertrophic heart, heart failure, ischemic heart disease. *^5^Hypertension, dyslipidemia, diabetes, obesity, chronic kidney disease, sleep apnea syndrome, etc. (see **“Chapter V. 2.5 Management of Risk Factors and Comorbidity”** for details). *^6^Appropriate therapeutic intervention for basic and/or complicated diseases (see **“Chapter V. 6 Upstream Therapy”** for details). *^7^Insurance coverage for amiodarone is approved in Japan only for patients with AF and hypertrophic cardiomyopathy or heart failure. Insurance coverage for sotalol is not approved for patients with AF, although the efficacy of sotalol on AF complicated with ischemic heart disease. *^8^bepridil is reported to be effective for AF with ventricular dysfunction; however there are reports warning of exaggeration of proarrhythmia. AF, atrial fibrillation.

Because antiarrhythmic agents affect the ionic currents in the myocardium, they can cause various secondary pathologies such as negative inotropic effects and the development of proarrhythmic effects through QT prolongation due to delayed repolarization. This revision of the guideline follows the concept in the 2013 revised edition^2^ and describes the drugs indicated for cases with and without organic heart disease (hypertrophic heart, heart failure, ischemic heart disease). In addition, the conditions that are related to AF such as hypertensproion, dyslipidemia are described as complications (Figure 18).

##### 5.5.2.1 Atrial Fibrillation Without Organic Heart Disease

As the background of AF without organic heart disease, systemic pathological conditions such as aging, hypertension, dyslipidemia, and hyperuricemia, as well as activity of the autonomic nervous system are involved. AF can be divided into nighttime type (parasympathetic nerve activation type), daytime type (sympathetic nerve activation type), and all‐day type in relation to autonomic nervous system activity. There are studies that evaluated the effects of drugs with M2 receptor blocking action or *β*‐blocking action in each type of AF, but a universal effect can be expected with strong Na^+^ channel blockers (Figure 18). There is no evidence for any specific drug (e.g., angiotensin‐receptor blockers), which were once anticipated as upstream therapy, exhibiting an anti‐remodeling effect for AF, but strict control of hypertension or dyslipidemia has been effective in suppressing AF as a control measure for these complicating diseases.^234^, ^561^ Therefore, control of complicating diseases is important for controlling AF (Figure 18).

Regarding the long‐term preventive effect of pilsicainide for AF recurrence, it was reported as effective in 53.8% during 12‐month observation for daytime‐type AF.^553^ However, the effect of a Na^+^ channel blocker is expected to diminish over time, because the target of the blocker (i.e., expression of the Na^+^ channel itself) will be decreased during continuation of AF. Cibenzoline is more effective than pilsicainide for prevention of AF lasting ≥48 h because it has effects on several K^+^ channels.^571^ Propafenone, a Na^+^ channel blocker with a *β*‐blocking effect, is reported to be more effective than the others for daytime‐type AF.^572^ These findings will help with choosing specific drugs for individual patients.

The effects of flecainide and propafenone have been demonstrated in a Western report.^534^ A randomized controlled trial (RCT) conducted in Japan also demonstrated that the 1‐month preventive efficacy of flecainide for paroxysmal AF was 39.4% (vs. placebo 3.1%).^573^

Regarding the effect of bepridil for AF prevention, Nakazato et al. have reported 18‐month sinus rhythm maintenance in 81% of 86 cases in whom defibrillation succeeded.^548^ Even in comparison with amiodarone, bepridil exhibited a higher AF prevention rate (75%) in comparison with amiodarone (50%) during 14.7 months’ observation.^574^ In contrast, Shiga et al. observed that 23.5% of cases transitioned to permanent AF from paroxysmal AF even under continuation of bepridil therapy, and concluded that its long‐term effect might be limited.^575^ There are some recent reports of the efficacy of pilsicainide or bepridil for cases of AF recurrence after successful catheter ablation. Bepridil has been reported as more effective for cases of remodeled enlarged left atrium. In cases of AF without organic heart disease, bepridil is reported to be effective for Na^+^ channel blocker refractory AF.^568^

It is controversial when pharmacological therapy should be stopped if it is effective for AF prevention. In a study comparing short‐tem (4 weeks) and long‐term (6 months) treatment,^576^ the recurrence rate was higher with short‐term treatment but the difference was only 20%. Considering the cost and risk of long‐term treatment, short‐term treatment may have its own benefit. An observational study that evaluated long‐term treatment (mean 3.4 years) documented cases of death related to pharmacological treatment,^577^ so careless long‐term administration should be avoided. The actual decision for continuation or cessation, as well as the dosing of antiarrhythmic agents, should be performed by considering the individual conditions of AF as well as liver–kidney function (Table 48).

**Table 48:** Standard Doses of Representative Antiarrhythmic Drugs

Drugs	Usual daily dosage (mg)	Standard daily oral administration times	Standard intravenous administration dosage
pilsicainide	150	3 times daily	1 mg/kg/10 min
cibenzoline	300	3 times daily	1.4 mg/kg/2–5 min
propafenone	450	3 times daily	–
flecainide	200	twice daily	1–2 mg/kg/10 min
bepridil	100–200	once or twice daily	–

Administration should start with the smaller dose, then can be increased to a higher dose under careful monitoring of side and/or proarrhythmic effects.

##### 5.5.2.2 Atrial Fibrillation With Organic Heart Disease

Organic heart disease, such as hypertrophic heart, heart failure, ischemic heart disease, may influence hemodynamics during AF, resulting in more serious symptoms. Therefore, AF prevention is more important in these cases, but the effects of antiarrhythmic agents and catheter ablation will be limited because of the progression in atrial remodeling. Negative inotropic action and/or QT prolongation may appear more strongly in these cases. Therefore, upstream therapy (i.e., not only monotherapy but also appropriate therapeutic strategy) for the basic disease becomes important (Figure 18).

The use of renin–angiotensin system blockers (angiotensin‐converting enzyme inhibitor/angiotensin‐receptor blocker) was related to a decrease of AF in a the subanalyses of several RCTs,^569^, ^570^, ^578^, ^579^ but the effect of a single intervention, so‐called “upstream therapy”, has been denied.^235^, ^552^, ^580^ These drugs should be used as basic appropriate therapy for heart failure with *β*‐blockers^581^ as “upstream therapy” in the broad sense.

Because of the presence of organic heart disease, the target of antiarrhythmic agents will change due to changes in the expression of ion channels. Generally, organic heart disease causes atrial fibrosis, which promotes the formation of random reentry within the atria.^555^ Under such conditions, it will be difficult to suppress the many reentries by simple conduction block caused by a Na^+^ channel blocker. On the other hand, prolongation of the action potential duration caused by a K^+^ channel blocker can make it difficult to form functional reentry in any part of the atria. Therefore, a K^+^ channel blocker will be more effective than a Na^+^ channel blocker in heart failure.^582^ In Japan, amiodarone, sotalol, bepridil and class IA antiarrhythmic agents can be used as K^+^ channel blockers, but a Na^+^ channel blocker cannot be used in heart failure, because of the negative inotropic effect.

Japanese insurance coverage for persistent AF is limited for amiodarone in heart failure or hypertrophic cardiomyopathy, or for bepridil. Because there are some reports of QT prolongation and torsade de pointes during bepridil therapy for AF with organic heart disease,^583^ bepridil is not described as first‐line therapy, but amiodarone and sotalol are listed with comments about limited insurance coverage (Figure 18). Regarding the use of amiodarone for AF with heart failure, the CHF‐STAT study demonstrated the effect for defibrillation and sinus rhythm maintenance.^584^ In that study, sinus rhythm could be maintained in 31% of 51 cases (vs. placebo 8%) and the patients with sinus rhythm exhibited better prognosis than those with AF. However, there may have been a selection bias because the patients in whom defibrillation was successful were assigned to the sinus rhythm group.

In the AF‐CHF study,^508^ an intention‐to‐treat comparison was performed between sinus rhythm and rate control groups. In this study, the sinus rhythm group, which was mainly treated with amiodarone, demonstrated 46–83% of sinus rhythm (vs. 30–41% rate control group) but no difference was observed in prognosis. The study denied the option of maintaining sinus rhythm recovery in drug‐refractory cases, but rhythm control with amiodarone might be an option initially, because the prognosis itself is better with sinus rhythm than with AF.

In the CASTLE‐AF study,^229^ rhythm control and rate control were compared using catheter ablation as the tool for rhythm control in cases of ventricular dysfunction. As a result, the rhythm control group exhibited lower mortality and higher recovery of ventricular dysfunction. Because ablation is more difficult in cases of AF with organic heart disease, the result should be reconfirmed in standardized interventional trials. However, the roles of pharmacological and non‐pharmacological treatments for AF prevention must be further investigated together.

### 5.6 Upstream Therapy

Atrial remodeling (ATR) creates the arrhythmogenic substrate for AF and plays a critical role in the pathophysiology. ATR is characterized by changes in the electrical and structural properties of atrial tissues and cells. These changes are caused by AF‐promoting pathological mediators, including neurohumoral factors (catecholamines, angiotensin II, etc.), growth factors (transforming growth factor‐*β*, etc.), stretch, inflammation, and oxidative stress. “Upstream therapy” is the pharmacological intervention targeting these upstream pathological mediators that promote AF. Although upstream therapy has been demonstrated to prevent ATR and AF in experimental studies, its efficacy has not been validated in clinical studies. There is thus a gap between the results of experimental and clinical research. Evidence of upstream therapy against AF in comorbid diseases associated with AF is shown in Table 49.^173^, ^234^, ^235^, ^521^, ^569^, ^570^, ^578^, ^580^, ^585^, ^586^, ^587^, ^588^, ^589^, ^590^, ^591^, ^592^

**Table 49:** Recommendations and Levels of Evidence for Upstream Therapy for AF Prevention in Underlying Comorbid Diseases

	COR	LOE	GOR (MINDS)	LOE (MINDS)
Use of ACEI/ARB and *β*‐blocker for primary prevention of AF in patients with heart failure and reduced ejection fraction^173^, ^521^, ^569^, ^570^, ^585^, ^586^, ^587^, ^588^	IIa	A	B	I
Use of ACEI/ARB for primary prevention of AF in patients with hypertension and left ventricular hypertrophy^578^, ^589^, ^590^, ^591^	IIa	B	C1	II
Use of ACRI/ARB and statin for the primary and secondary prevention of AF in patients without underlying comorbid diseases^234^, ^235^, ^580^, ^592^	III	B	C2	II

Abbreviations: ACEI, angiotensin‐converting enzyme inhibitor; AF, atrial fibrillation; ARB, angiotensin‐receptor blocker; COR, class of recommendation; GOR, grade of recommendation; LOE, level of evidence; MINDS, Medical Information Network Distribution Service.

#### 5.6.1 Angiotensin‐Converting Enzyme Inhibitors and Angiotensin‐II Receptor Blockers

The renin–angiotensin–aldosterone system (RAAS) is activated by AF and AF‐associated comorbid diseases, which increases the production of angiotensin II (AT‐II), a pathological mediator for AF. Binding of AT‐II to the receptor in atrial cardiomyocytes and fibroblasts activates profibrotic signaling and promotes fibrotic remodeling. Treatment with angiotensin‐converting enzyme inhibitors (ACEIs) and angiotensin‐receptor blockers (ARBs) suppresses ATR and AF in animal models of AF.^593^, ^594^, ^595^

In subanalyses and meta‐analyses of clinical studies, treatment with ACEIs or ARBs has demonstrated efficacy for primary prevention of AF in patients with heart failure, hypertension, and left ventricular hypertrophy.^173^, ^569^, ^570^, ^578^, ^585^, ^586^, ^587^, ^588^, ^589^, ^590^, ^591^ However, that efficacy has not been validated by a large‐scale randomized controlled trial (RCT).

Regarding efficacy for secondary prevention of AF, treatment with ACEIs or ARBs neither prevented AF recurrence^235^, ^580^ nor improved mortality^596^ in paroxysmal and persistent AF patients who had sinus rhythm restored after pharmacological or electrical cardioversion. In the J‐RHYTHM II trial (the Japanese rhythm management trial II for AF), an open‐label randomized study, treatment with an ARB (candesartan) lowered blood pressure in hypertensive patients with paroxysmal AF, and also decreased the frequency and symptoms of AF. However, these effects were comparable to the effects of treatment with a Ca^2+^ channel blocker (CCB, amlodipine); the ARB did not show additional antiarrhythmic effect in AF patients.^234^ In a similar RCT in Asia, treatment with an ARB (telmisartan) did not improve efficacy for secondary prevention of AF in hypertensive patients with paroxysmal AF when compared with a CCB (nifedipine); however, the percentage of patients who developed AF with a transition from the paroxysmal to persistent form significantly decreased with telmisartan compared with nifedipine, suggesting that ARBs have potential to prevent AF maintenance associated with ATR.^597^

#### 5.6.2 Mineralocorticoid‐Receptor Antagonists

In an experimental study, treatment with a mineralocorticoid‐receptor antagonist (MRA) suppressed atrial fibrosis in an animal model of heart failure.^598^ In a subanalysis of the EMPHASIS‐HF trial, patients treated with a MRA showed lower incidence of new‐onset AF than those treated with placebo.^599^ MRAs show potential to prevent AF,^600^, ^601^ but clinical efficacy has not been validated by a large‐scale RCT.

#### 5.6.3 Statins

Statins exhibit actions beyond lipid lowering in the prevention of various cardiovascular diseases, including AF, known as “pleiotropic effects”. Statins are thought to prevent AF and stroke through modulation of the inflammatory response, improvement of endothelial function, and prevention of blood clot formation. Statin treatment has suppressed AF and ATR progression in experimental studies.^602^, ^603^ However, the preventive effect of statins has not been validated in patients with AF‐associated diseases such as heart failure and hypertension.^592^, ^604^, ^605^ In open‐heart surgery, periprocedural statin treatment has been demonstrated to prevent postoperative AF.^606^, ^607^, ^608^ However, in a recent RCT, statin treatment failed to show a protective effect against postoperative AF and increased the risk of acute renal injury compared with placebo.^609^

#### 5.6.4 *β*‐Blockers

Cardiac autonomic nerves are nested in the epicardial adipose tissues of the heart, forming an intrinsic network (i.e., ganglionated plexus, GP). Sympathetic and parasympathetic activities alter the intracellular Ca^2+^ dynamics and thus the cellular electrophysiological properties, facilitating AF.^610^ In a histological analysis, autonomic nerve hyperplasia within the GP was observed in canine models of heart failure with AF substrate.^611^ Treatment with *β*‐blockers has prevented AF recurrence in patients who had sinus rhythm restored after pharmacological or electrical cardioversion.^581^, ^612^ In a meta‐analysis, *β*‐blocker treatment was effective for primary prevention of AF in heart failure patients with reduced cardiac function.^521^, ^588^

#### 5.6.5 Conclusion

Apart from AF, ACEI/ARB, MRA, and *β*‐blocker have become a practical standard therapy for heart failure with reduced ejection fraction. ACEI/ARB are also commonly used to treat hypertension. These drugs target upstream mediators of AF but their effect on AF prevention has not been validated in clinical practice. Although evidence for the clinical benefit of upstream therapy against AF is still limited, it can be anticipated when these drugs are used as a standard treatment for the comorbid diseases associated with AF.

### 5.7 Indication and Timing of Non‐Pharmacological Therapy

Atrial fibrillation (AF) progresses from paroxysmal to persistent, leading to dilatation of the left atrium (LA). Catheter ablation (CA) is less effective in the advanced stage, so should be considered before progression of AF.

#### 5.7.1 Catheter Ablation for Symptomatic Atrial Fibrillation

**Figure 19:**
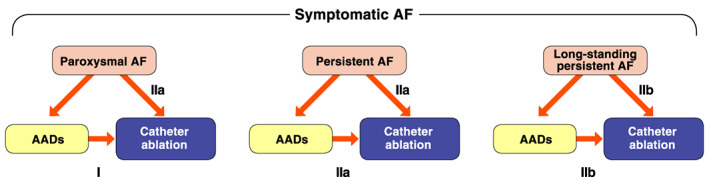
Flowchart diagram of treatment for symptomatic AF patients based on its persistence. AAD, anti‐arrhythmic drugs; AF, atrial fibrillation; CA, catheter ablation. (Adapted from the Japanese Circulation Society 2019^3^)

CA for symptomatic drug‐refractory paroxysmal AF (≥1 class I or III drugs) is recommended in the 2018 JCS/JHRS “Guideline on Non‐Pharmacotherapy of Cardiac Arrhythmias” (Class I; Level of Evidence A, Figure 19, ^4^, Table 50, ^4^). Early CA for drug‐refractory AF is recommended because it is high likely to progress to persistent AF, resulting in LA dilatation. There are 3 randomized controlled trials (RCTs) about CA as a first‐line therapy for symptomatic paroxysmal or persistent AF.^613^, ^614^, ^615^ Furthermore, a meta‐analysis including these RCTs has been reporrted.^616^ All of these studies showed a significant reduction of AF burden in the CA group, and a low incidence of procedure‐related complications. These results suggest that it is reasonable to perform CA as a first‐line therapy for symptomatic paroxysmal AF.^4^

**Table 50:** Recommendations and Levels of Evidence for Catheter Ablation of Symptomatic Drug‐Refractory Paroxysmal AF

	COR	LOE	GOR (MINDS)	LOE (MINDS)
Drug‐refractory* symptomatic paroxysmal AF	I	A	A	I
Symptomatic paroxysmal AF (as a first‐line therapy)	IIa	B	B	I
AF complicated with HF (reduced LV function) (applying the same recommendations used for normal LV function)	IIa	B	B	I
Paroxysmal AF complicated with bradycardia–tachycardia syndrome	IIa	B	B	III
Symptomatic persistent AF (drug‐refractory or as a first‐line therapy)	IIa	B	B	II
Symptomatic longstanding persistent AF (drug‐refractory or as a first‐line therapy)	IIb	B	B	II
Recurrent asymptomatic paroxysmal AF	IIb	C	C1	III
Asymptomatic persistent AF	IIb	C	C1	III
AF with definite or suspected left atrial thrombus	III	A	D	V
AF with contraindication of anticoagulation	III	A	D	V

* Refractory to at least one of class I or III antiarrhythmic drugs.

Abbreviations: AF, atrial fibrillation; COR, class of recommendation; GOR, grade of recommendation; HF, heart failure; LOE, level of evidence; LV, left ventricular; MINDS, Medical Information Network Distribution Service.

(Adapted from JCS and JHRS 2021.^3^)

Regarding CA as a first‐line therapy for symptomatic persistent AF, prospective studies showed higher rates of maintenance of sinus rhythm in the CA group compared with the amiodarone group.^617^, ^618^ It is therefore also reasonable to perform CA as a first‐line therapy for symptomatic persistent AF, given that antiarrhythmic drugs are less effective.^4^ There is insufficient evidence to support CA as a first‐line therapy for symptomatic longstanding AF. Because longstanding persistent AF is accompanied by significant electrical and structural remodeling of the atria, it is difficult to restore and maintain sinus rhythm despite repeated ablation procedures. Factors such as the degree of LA dilatation, patient’s age, symptoms, and patient preference should be taken into account for the indication of CA.^4^

#### 5.7.2 Catheter Ablation for Atrial Fibrillation With Heart Failure (Reduced Left Ventricular Function)

Five RCTs comparing the efficacy of CA to medical therapy (rate control) in patients with low cardiac function have been reported to date,^619^, ^620^, ^621^, ^622^, ^623^ and a meta‐analysis including 4 of those studies has been conducted.^624^ According to the analysis, 224 patients, of whom 82.5% had persistent AF, were randomized to CA group or rate control group. AF ablation was associated with an increase in left ventricular ejection fraction (LVEF, mean difference 8.5%) compared with rate control. CA was superior in improving quality of life (QOL) and increasing peak oxygen consumption compared with rate control. Major adverse event rates were not significantly different between the 2 groups.

The AATAC trial was a RCT comparing efficacy of CA for sinus rhythm maintenance to amiodarone over a 2‐year follow‐up. Sinus rhythm maintenance rate was significantly higher in CA group compared with the amiodarone group (70% vs. 34%). CA was superior in improving QOL and reducing unplanned hospitalizations and mortality compared with amiodarone.^625^

The CASTLE‐AF trial was a RCT designed to investigate whether CA is associated with better prognosis than medical therapy (rate or rhythm control) in patients with heart failure (HF) and AF. After a 3‐year follow‐up, CA for AF in patients with HF was superior to medical therapy in sinus rhythm maintenance, improving cardiac function and prognosis.^222^ Based on these results, the JCS/JHRS “Guideline on Non‐Pharmacotherapy of Cardiac Arrhythmias” (revised in 2018) suggested that CA may improve the prognosis of AF patients with HF, and recommended the same indication (Class IIa, Level of Evidence B) in patients with and without HF (reduced left ventricular function).^4^

#### 5.7.3 Catheter Ablation for Bradycardia–Tachycardia Syndrome

Pacemaker (PM) implantation has been recommended as a first‐line therapy for symptomatic patients with bradycardia–tachycardia syndrome (BTS). However, some patients who have sinus pause only after termination of AF could be treated by CA without PM.^626^, ^627^ The indication should be discussed in detail because of insufficient evidence and lack of RCTs (Class IIa, Level of Evidence B).^4^

#### 5.7.4 Catheter Ablation for Elderly Patients

Many AF patients are elderly, and the efficacy and safety of CA for older AF patients have been reported.^628^, ^629^ Therefore it is reasonable that elderly patients (generally ≥75 years old) with preserved ADL (activities of daily living) have the same therapeutic indication as younger patients. However, CA for elderly patients with persistent or longstanding persistent AF has a lower recommendation than that for young patients because of insufficient evidence. So far, CA for persistent or longstanding persistent AF has limitations, and patients often require repeat procedure. Furthermore, it is often better to choose medical therapy (rate control) in asymptomatic patients with persistent AF. Therefore it is important to make the decision after informing patients about the risks and benefits of CA.

#### 5.7.5 Catheter Ablation for Asymptomatic Atrial Fibrillation

CA for AF has been limited to symptomatic cases. However, recently it was reported that CA can improve prognosis regardless of the presence or absence of symptoms,^630^ and the prognosis of asymptomatic AF patients was worse than that of symptomatic AF patients.^631^ CA is recommended because it can improve prognosis, even if patients are asymptomatic at the time of AF diagnosis, without deterioration of QOL. Based on this consideration, CA for asymptomatic AF patients was newly introduced in the JCS/JHRS “Guideline on Non‐Pharmacotherapy of Cardiac Arrhythmias” (revised in 2018).^74^

To date, 4 studies regarding the efficacy and safety of CA for asymptomatic AF have been reported.^632^, ^633^, ^634^, ^635^ Forleo et al reported that the efficacy of CA in asymptomatic AF patients was the same as that in symptomatic patients.^632^ However, Wu et al demonstrated that symptoms got worse in many of the asymptomatic AF patients after CA because of the development of atrial tachycardia.^633^ Meanwhile, improvement in exercise tolerance, B‐type natriuretic peptide level, and QOL after CA have been reported, even when patients were asymptomatic.^634^, ^635^ Currently, the indication of CA for asymptomatic AF patients should be discussed in detail because of the lack of RCT evidence. CA for asymptomatic longstanding persistent AF is not recommended because it is too late in the clinical course (repeated recurrence of asymptomatic paroxysmal AF, asymptomatic persistent AF: Class IIb, Level of Evidence C).^4^

#### 5.7.6 Atrioventricular Node Ablation for Atrial Fibrillation With Uncontrollable Rapid Heart Rate

AV node ablation is recommended for AF patients with medically uncontrollable heart rate, who have severe symptoms of tachycardia or severely reduced heart function or decline of QOL, in cases of unsuccessful or contraindication of CA.^4^ The indication should be discussed for each case, because a permanent pacemaker is needed.

#### 5.7.7 Pacemaker Implantation for Bradycardic Atrial Fibrillation

PM implantation is recommended in AF patients with bradycardia‐related symptoms such as faintness and shortness of breath (Class I).^636^

## 6 ATRIAL FLUTTER / ATRIAL TACHYCARDIA

### 6.1 Atrial Tachycardia

#### 6.1.1 Pathophysiology

Focal atrial tachycardia (AT) is defined as a fast rhythm from a discrete origin, discharging at a rate that is generally regular, and conducting in a centrifugal manner throughout the atrial tissue. The atrial rate during focal AT is generally between 100 and 250 beats/min. Focal AT represents approximately 3–17% of patients referred for supraventricular tachycardia ablation, and most patients with focal AT are observed to be in the young population.^637^, ^638^ Focal AT in the adult population is usually associated with a benign prognosis; non‐sustained focal AT is common and often does not require treatment, although AT‐induced cardiomyopathy has been reported in up to 10% of patients referred for ablation of incessant supraventricular tachycardia.^639^ Automaticity, triggered activity or micro‐reentry can be considered as the underlying mechanism of focal AT, although methods to distinguish these mechanisms through pharmacological examination or electrophysiological study have modest value because of limited sensitivity and specificity.^640^

Focal AT has been localized to the crista terminalis, right or left atrial free wall or appendage, tricuspid or mitral annulus, para‐septal or para‐nodal areas, pulmonary veins, coronary sinus, and coronary cusps on the basis of mapping during electrophysiological studies and successful catheter ablation. Generally, focal AT originates more frequently from the right atrium than from the left atrium.^112^ Algorithms have been developed to evaluate the origin of the focal AT from the P‐wave morphology assessed on a standard 12‐lead ECG. A positive P wave in lead V1 and negative P waves in leads I and aVL are commonly correlated to AT originating from the left atrium, and positive P waves in leads II, III, and aVF indicate that the origin of AT is from the cranial portion of either atria. Shorter P‐wave duration is correlated to AT originating from the para‐septal portion versus the right or left atrial free wall.^639^, ^641^

Sinus node reentrant tachycardia involves a micro‐reentrant circuit in the region of the sinoatrial node, and atrial rate during tachycardia is generally 100–150 beats/min. The P‐wave morphology is identical to that of sinus tachycardia. The differential diagnosis of sinus node reentrant tachycardia from sinus tachycardia is the abrupt onset and termination and often a longer RP interval than that observed during normal sinus rhythm, possible induction according to programmed stimulation, and possible demonstration of entrainment.^642^

Adenosine‐sensitive atrial reentrant tachycardia also involves a micro‐reentrant circuit in the region of the atrioventricular nodal transitional area, depending on the Ca^2+^ channel current.^643^ This tachycardia has been localized to the anteroseptal or posteroseptal area, cavotricuspid isthmus and lateral tricuspid annulus on the basis of mapping during electrophysiological studies and successful catheter ablation, and can be terminated by rapid intravenous administration of adenosine triphosphate (2–5 mg). The incidence of left atrial tachycardia following pulmonary vein isolation for paroxysmal and persistent atrial fibrillation that involves a micro‐reentrant circuit arising from the mitral annulus or transitional area between the pulmonary veins and left atrium is increasing rapidly; a recent report presents approximately 1–6% of the patients referred for atrial tachycardia ablation.^644^

#### 6.1.2 Treatment

##### 6.1.2.1 Acute Treatment

The strategy of antiarrhythmic drug therapy in the acute setting should be performed on the basis of hemodynamics and organic heart disease in patients with AT. The class of recommendation and levels of evidence are presented in Table 51.^1^, ^21^, ^104^, ^640^, ^645^, ^646^, ^647^, ^648^, ^649^, ^650^, ^651^, ^652^ Synchronized electrical cardioversion (with 50–100 J) is considered as a first‐line therapy in AT patients with cardiogenic shock, heart failure and acute myocardial ischemia.^645^, ^646^ RCTs of antiarrhythmic drug therapy for comparative efficacy and safety in patients with AT in the acute setting are not available.^647^, ^648^ In many reports, the response to intravenous antiarrhythmic drug therapy was estimated by electrophysiological study rather than in the clinical environment.^639^, ^640^, ^642^, ^643^, ^647^, ^648^, ^649^, ^650^, ^651^, ^652^, ^653^, ^654^, ^655^, ^656^, ^657^ Intravenous adenosine triphosphate and atrial overdrive pacing are usually effective in inhibiting AT with an automatic or a triggered mechanism, and intravenous *β*‐blockers, non‐dihydropyridine Ca^2+^ channel blockers (diltiazem, verapamil) and Class I antiarrhythmic drugs are also effective in terminating AT with the abovementioned mechanisms.^640^, ^649^, ^651^ Vagal maneuvers, intravenous *β*‐blockers, diltiazem and verapamil are effective in terminating AT with a micro‐reentrant mechanism when the micro‐reentrant circuit involves the sinoatrial or atrioventricular nodal area.^104^ However, in case of macro‐reentrant circuit involving working myocardium of the atrium, these drugs are not effective to terminate AT. Intravenous digoxin is not selected for terminating AT because of its slow pharmacokinetics. Although these drugs are relatively safe in hemodynamically stable patients with AT, close monitoring is recommended during intravenous antiarrhythmic drug therapy to evaluate for hypotension, bradycardia or heart failure, especially of patients with childhood and manifest Wolff‐Parkinson‐White syndrome.

**Table 51:** Recommendations and Levels of Evidence for Acute Treatment of AT

	COR	LOE	GOR (MINDS)	LOE (MINDS)
Emergency synchronized electrical cardioversion for the termination of hemodynamically unstable or drug‐resistant AT^645^, ^646^	I	C	C1	VI
Intravenous *β*‐blockers, diltiazem or verapamil for the termination of hemodynamically stable AT or rate control therapy^640^, ^647^, ^648^, ^651^	IIa	C	B	IVb
Intravenous adenosine triphosphate for the termination of AT or differential diagnosis of SVT^640^, ^650^, ^651^	IIa	B	B	III
Intravenous Class I antiarrhythmic drugs for the termination of hemodynamically stable AT without organic heart disease^640^, ^649^, ^652^	IIa	C	B	IVb
Self‐administration of Class I antiarrhythmic drugs (pill‐in‐the‐pocket) for the necessary confirmation for efficacy and safety of drugs on ECG before this approach* ^104^	IIa	B	B	II
Intravenous Class I antiarrhythmic drugs for the termination of hemodynamically unstable AT or AT with moderate/severe cardiac dysfunction^1^, ^21^	III	C	C2	IVb
Intravenous Class I antiarrhythmic drugs for the termination of AT in patients with Brugada syndrome or tachycardia–bradycardia syndrome^1^, ^21^	III	C	C2	IVb

* For example, severe bradycardia following termination of AT, occurrence of bundle block, cardiac dysfunction according to negative inotropic action, past history of atrial flutter.

Abbreviations: AT, atrial tachycardia; COR, class of recommendation; ECG, electrocardiogram; GOR, grade of recommendation; LOE, level of evidence; MINDS, Medical Information Network Distribution Service; SVT, supraventricular tachycardia.

On the other hand, out‐of‐hospital self‐administration of antiarrhythmic drugs after the onset of cardiac palpitations, so‐called “pill‐in‐the pocket” therapy, is a favorable approach for AT termination in hemodynamically stable patients with severe subjective symptoms and low frequency.^531^ This approach can not only improve the efficacy of pharmacological AT termination according to subsequent treatment after the onset of cardiac palpitations, but also avoid admission to the emergency room with cardiac palpitations. Oral *β*‐blockers, verapamil and Class I antiarrhythmic drugs such as pilsicainide or cibenzoline are well selected to restore normal sinus rhythm. However, it is necessary to confirm the efficacy and safety of antiarrhythmic drugs on the basis of close monitoring on ECG before using this approach. Synchronized electrical cardioversion under intravenous anesthesia and atrial overdrive pacing are recommended in patients with AT refractory to this approach. Table 51 indicatepatiensts the recommendations and level of evidence for the acute treatment of AT.^1^, ^21^, ^104^, ^640^, ^645^, ^646^, ^647^, ^648^, ^649^, ^650^, ^651^, ^652^

##### 6.1.2.2 Prophylactic Therapy of Atrial Tachycardia

Catheter ablation is recommended for patients with symptomatic AT, deteriorated quality of life, incessant AT and failed or adverse response to antiarrhythmic drugs.^639^ In experienced centers, where AT can be induced in the laboratory, acute success rates >90–95% have consistently been reported, with a complication rate of <1–2%.^638^, ^640^, ^652^ In patients in whom ablation is not being considered because of unsuccessful procedure or because of patient preference, a variety of antiarrhythmic drugs are available. Oral *β*‐blockers,^640^ verapamil and Class I antiarrhythmic drugs^651^, ^654^, ^655^, ^656^, ^657^, ^658^, ^659^ are relatively useful in patients with AT, although there is limited evidence regarding their efficacy and safety.

Several studies report moderate efficacy of oral amiodarone in maintaining sinus rhythm as long‐term treatment in children.^660^, ^661^ Although most reports are in children, limited data suggest similar efficacy in adults.^662^ It is preferable to select oral amiodarone for adult patients with heart failure or failed response to the abovementioned antiarrhythmic drugs.^663^ On the other hand, ongoing management with antithrombotic therapy is recommended in adult congenital heart disease patients and AT to align with recommended antithrombotic therapy for patients with atrial flutter or atrial fibrillation.^664^ Recommendations for ongoing management of AT and clinical evidence from observational, prospective studies and meta‐analysis are shown in Table 52.^1^, ^21^, ^640^, ^647^, ^648^, ^652^, ^656^, ^657^, ^658^, ^659^, ^660^

**Table 52:** Recommendations and Levels of Evidence for Ongoing Management of AT

	COR	LOE	GOR (MINDS)	LOE (MINDS)
Use of oral *β*‐blockers, diltiazem or verapamil for the prevention of symptomatic AT^640^, ^647^, ^648^, ^652^	IIa	C	B	IVb
Use of oral Class I antiarrhythmic drugs for the prevention of hemodynamically stable AT without organic heart disease^656^, ^657^, ^659^, ^660^	IIa	C	B	IVb
Use of oral Class III antiarrhythmic drugs (amiodarone) for the prevention of Class I antiarrhythmic drug‐resistant AT or AT with moderate/severe cardiac dysfunction^652^	IIa	C	B	IVb
Use of oral Class I antiarrhythmic drugs for the prevention of hemodynamically unstable AT or AT with moderate/severe cardiac dysfunction^1^, ^21^	III	C	C2	IVb
Use of oral Class I antiarrhythmic drugs* for the prevention of AT in patients with Brugada syndrome or tachycardia–bradycardia syndrome^1^, ^21^	III	C	C2	IVb

* For example: pilsicainide, cibenzoline, propafenone, flecainide.

Abbreviations: AT, atrial tachycardia; COR, class of recommendation; GOR, grade of recommendation; LOE, level of evidence; MINDS, Medical Information Network Distribution Service.

### 6.2 Atrial Flutter

#### 6.2.1 Pathophysiology

Atrial flutter (AFL) is a macro‐reentrant atrial arrhythmia characterized by a regular atrial rate of 240–440 beats/min and constant P‐wave morphology. AFL is electrocardiographically classified into 2 types: slow cycle length at atrial rates of 240–340 beats/min (type I), and fast cycle length at atrial rates of 340–440 beats/min (type II).^665^ Most of the type I AFL circuits rotating around the tricuspid valve involve the cavotricuspid isthmus (CTI), and are labeled CTI‐dependent AFL.^666^ When CTI‐dependent AFL involves a circuit that rotates around the tricuspid valve in a counterclockwise direction, it is the so‐called “common type”; less commonly, the CTI‐dependent AFL circuit rotates in a clockwise direction, also known as the “uncommon type”.^667^ Common‐type AFL is characterized electrocardiographically by dominant negative flutter waves in the inferior leads (so‐called “sawtooth waves”) and a positive P wave in lead V_1_. In contrast, uncommon‐type AFL has the opposite pattern (i.e., positive flutter waves in the inferior leads and wide, negative flutter waves in lead V1). It is common for AF and AFL to coexist in the same patient. Previous studies have reported that 22–82% of patients developing AF underwent CTI ablation within a mean follow‐up of 14–60 months,^668^, ^669^, ^670^, ^671^ and AFL is often observed in patients with AF treated with Class I antiarrhythmic drugs (so‐called “IA or IC flutter”).^672^

In contrast, non‐isthmus‐dependent AFL, or uncommon flutter, describes macro‐reentrant ATs that are not dependent on conduction through the CTI.^673^, ^674^ A variety of circuits have been described, including a path around the mitral annulus (perimitral flutter), reentry involving the left atrial roof, and reentry around regions of scarring in the right or left atrium.^675^ Non‐isthmus‐dependent AFL can involve multiple atrial reentry circuits and can often occur in patients with atrial scarring from prior cardiac surgery or ablation, but also may occur in any form of cardiac disease or may be idiopathic. The reentrant circuits are classified as either macro‐reentrant AT (large; often several centimeters or longer in diameter) or micro‐reentrant AT (≤2 cm in diameter), which may be indistinguishable from AT.^676^

#### 6.2.2 Treatment

##### 6.2.2.1 Acute Treatment; Recommendation

**Figure 20:**
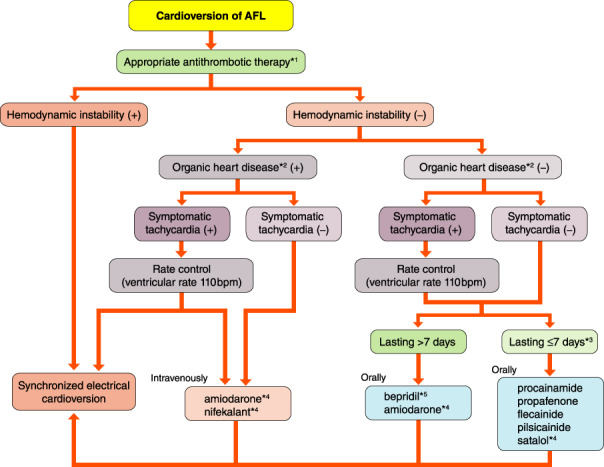
Pharmacotherapy to restore sinus nodal rhythm in patient with hemodynamically stable AFL. *^1^Adequate anticoagulant therapy is required in patients with AFL for 3 weeks before cardioversion and 4 weeks after if it is unclear whether AFL lasts for ≤48 h. *^2^Cardiac hypertrophy, cardiac dysfunction and cardiac ischemia. *^3^In order to ensure efficacy and prevent thromboembolic complications, the duration of an AFL episode should be limited to ≤48 h. *^4^Class III antiarrhythmic drugs are not covered by the National Health Insurance in Japan. *^5^bepridil is not indicated for AFL in Japan. AFL, atrial flutter.

Figure 20, ^1^, ^404^, ^645^, ^646^, ^677^, ^678^, ^679^, ^680^, ^681^, ^682^, ^683^, ^684^, ^685^, Tables 53 and 54^1^, ^21^, ^404^, ^512^, ^686^, ^687^, ^688^, ^689^, ^690^, ^691^, ^692^, ^693^, ^694^, ^695^ indicate acute pharmacological treatment to restore sinus rhythm in patients hemodynamically stable patients with AFL. The strategy of antiarrhythmic drug therapy in the acute setting should be performed on the basis of the hemodynamics and organic heart disease in patients with Synchronized electrical cardioversion (with 50–100 J) under intravenous anesthesia is considered as a first‐line therapy in AFL patients with cardiogenic shock, heart failure and acute myocardial ischemia.^645^, ^646^, ^678^ Antiarrhythmic drugs therapy or synchronized electrical cardioversion is recommended to restore sinus nodal rhythm in hemodynamically stable patients with AFL (with appropriate considerations regarding anticoagulation).^677^, ^679^

**Table 53:** Recommendations and Levels of Evidence for Acute Treatment (Electrical Cardioversion) of AFL

	COR	LOE	GOR (MINDS)	LOE (MINDS)
Emergency synchronized electrical cardioversion for the termination of AFL with prolonged myocardial ischemia, symptomatic hypotension, exacerbation of heart failure and hemodynamically unstable or drug‐resistant AFL^645^, ^646^, ^677^	I	C	C1	IVb
Termination of hemodynamically unstable tachycardiac AFL with pre‐excitation syndrome^678^, ^679^	I	C	C1	IVb
Elective synchronized electrical cardioversion for the termination of drug‐resistant AFL lasting ≤48 h^680^, ^681^, ^682^	IIa	C	C1	IVb
Termination of AFL in whom the possibility of 48 h continuation cannot be denied but the absence of intra‐atrial thrombus is confirmed by transesophageal echocardiography, or appropriate anticoagulation therapy has been continued more than 3 weeks at the time of defibrillation^2^, ^21^, ^530^	IIa	C	C1	IVb
Termination of AFL with hyperthyroidism and susutained AFL following euthyroid status undergoing treatment or postoperative lasting AFL in which antiarrhythmic drugs are ineffective or contraindicated^1^	IIa	C	C1	IVb
Termination of AFL without support with pacing therapy in patients with advanced atrioventricular block or sick sinus syndrome^1^	III	C	C2	IVb
Termination of AFL by direct current defibrillation in patients with digitalis intoxication, hypokalemia or severe bradycardia^1^	III	C	C2	IVb
Termination of hemodynamically‐stable by direct current defibrillation for persistent AFL lasting ≥48 h without standard anticoagulation therapy	III	C	C2	IVb

Abbreviations: AFL, atrial flutter; COR, class of recommendation; GOR, grade of recommendation; LOE, level of evidence; MINDS, Medical Information Network Distribution Service.

**Table:** Table 54 Recommendations and Levels of Evidence for Acute Treatment (Pharmacotherapy) of AFL

	COR	LOE	GOR (MINDS)	LOE (MINDS)
Intravenous and oral diltiazem or verapamil for the rate control therapy in patients with hemodynamically stable AFL^687^	I	A	B	II
Anticoagulant therapy to restore sinus nodal rhythm for the pharmacological or electrical cardioversion in patients with AFL^404^, ^692^	I	A	B	II
Intravenous *β*‐blockers (landiolol) for the rate control therapy in patients with hemodynamically unstable AFL^512^	IIa	B	B	II
Intravenous digitalis for the rate control therapy in patients with hemodynamically unstable AFL^686^	IIa	C	B	II
Intravenous Class I antiarrhythmic drugs for the termination of hemodynamically stable AFL in patients without organic heart disease^688^, ^689^, ^690^, ^691^	IIa	C	B	IVb
Intravenous Class III antiarrhythmic drugs (amiodarone or nifekalant)* for the termination of symptomatic AFL^693^, ^694^, ^695^	IIa	C	B	IVb
Intravenous Class I antiarrhythmic drugs for the termination of hemodynamically unstable AFL or AFL with moderate/severe cardiac dysfunction^1^, ^21^	III	C	C2	IVb
Intravenous or self‐administration of Class I antiarrhythmic drugs (pill‐in‐the‐pocket) for the termination of AFL in patients with Brugada syndrome or tachycardia–bradycardia syndrome^1^, ^21^	III	C	C2	IVb

^*^ Class III antiarrhythmic drugs are not covered by the National Health Insurance in Japan.

Abbreviations: AFL, atrial flutter; COR, class of recommendation; GOR, grade of recommendation; LOE, level of evidence; MINDS, Medical Information Network Distribution Service.

K^+^ channel blockers or Na^+^ channel blockers with slow kinetics are considered as a first‐line therapy in terminating AFL because there is an excitable gap that corresponds to 20% of the cycle length^696^ and delayed conduction velocity^697^, ^698^ in the region of the CTI in patients with CTI‐dependent AFL.^693^, ^699^, ^700^, ^701^ In previous studies, the efficacy of Class I antiarrhythmic drugs such as intravenous disopyramide,^688^ oral propafenone,^689^ intravenous procainamide^690^ and intravenous flecainide^691^ in terminating AFL was comparable or inferior to that of Class III antiarrhythmic drugs. When Class I antiarrhythmic drugs with anticholinergic action, which may lead to a rapid 1 : 1 ventricular response, are intravenously administered to restore sinus nodal rhythm in patients with AFL, intravenous *β*‐blockers and non‐dihydropyridine Ca^2+^ channel blockers (diltiazem, verapamil), which can delay conduction velocity in the AV node through a direct pharmacological effect, are preferable to achieve favorable rate control for AFL.^687^

On the other hand, it has been reported in Europe, America and Japan that intravenous amiodarone is effective for terminating AFL.^694^, ^695^ However, intravenous amiodarone should be only administered to patients with AFL and heart failure, or who are refractory to Class I antiarrhythmic drug therapy, because intravenous amiodarone in patients with AFL is not covered by the National Health Insurance (NHI) in Japan. Synchronized electrical cardioversion under intravenous anesthesia is recommended for patients with AFL refractory to antiarrhythmic drug therapy.

Intravenous digoxin is generally selected to achieve favorable rate control for AFL in hemodynamically unstable patients with cardiac dysfunction.^686^ Intravenous *β*‐blockers (landiolol), diltiazem and verapamil are also effective as favorable rate control drugs for AFL.^512^, ^687^, ^689^ However, avoidance of concomitant diltiazem and verapamil use in patients with pre‐excited AFL, moderate or severe heart failure, sick sinus syndrome, AV block and intraventricular conduction disturbance without implantation of permanent pacemaker is recommended.

According to a randomized control study (J‐LAND study) in Japan, the efficacy and safety of intravenous landiolol has been demonstrated in patients with AFL and cardiac dysfunction.^512^ However, it is reported that the efficacy of intravenous landiolol for achieving favorable rate control in patents with AFL is inferior to that in patients with AF.^515^ In addition, careful management of intravenous landiolol for AFL is required in patients with severe cardiac dysfunction (left ventricular ejection fraction <25%) or hemodynamically unstable heart failure (systolic blood pressure <90 mmHg).

##### 6.2.2.2 Prophylactic Therapy of Atrial Flutter

**Figure 21:**
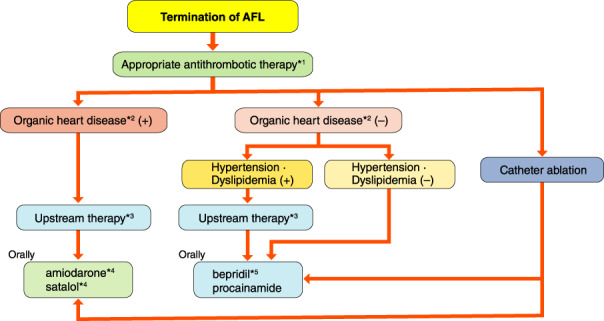
Pharmacotherapy to prevent AFL recurrence. *^1^Continuous rate control therapy is recommended for patients in whom it is not impossible for there to be recurrence of symptomatic AFL regardless of pharmacotherapy. *^2^Cardiac hypertrophy, cardiac dysfunction and cardiac ischemia. *^3^Appropriate therapeutic intervention to patient’s underlying disease. *^4^amiodarone is not indicatd for AFL in Japan. *^5^bepridil is not indicatd for AFL in Japan. AFL, atrial flutter.

Figure 21 and Table 55 indicate the pharmacological treatment for the prophylactic treatment of AFL.^1^, ^21^, ^80^, ^691^, ^695^, ^696^, ^697^, ^698^, ^699^, ^700^, ^701^, ^702^, ^703^, ^704^, ^705^, ^706^, ^707^, ^708^

**Table 55:** Recommendations and Levels of Evidence for Ongoing Management of AFL

	COR	LOE	GOR (MINDS)	LOE (MINDS)
Use of oral *β*‐blockers, diltiazem or verapamil for the rate control therapy in patients with hemodynamically stable AFL^691^	I	B	A	I
Anticoagulant therapy to maintain sinus nodal rhythm in patients with AFL^80^, ^703^, ^704^, ^705^, ^706^, ^707^, ^708^	I	A	A	II
Use of oral Class III antiarrhythmic drugs (bepridil or sotalol)* for the prevention of AFL with normal/mild reduced cardiac function^696^, ^697^	IIa	C	B	IVb
Use of oral Class III antiarrhythmic drugs to maintain sinus nodal rhythm (amiodarone)* for the prevention of Class I antiarrhythmic drug‐resistant AFL or AFL with moderate/severe cardiac dysfunction^701^	IIa	C	B	IVb
Use of oral Class I antiarrhythmic drugs to maintain sinus nodal rhythm for the prevention of AFL with normal/mild reduced cardiac function^698^, ^699^, ^700^	IIa	C	B	IVb
Use of oral Class III antiarrhythmic drugs (amiodarone or sotalol)* for the prevention of AFL with QT prolongation syndrome^695^, ^702^	III	C	C2	IVb
Use of oral Class I antiarrhythmic drugs to maintain sinus nodal rhythm for the prevention of hemodynamically unstable AFL or AFL with moderate/severe cardiac dysfunction^1^, ^21^	III	C	C2	IVb
Use of oral Class I antiarrhythmic drugs* for the prevention of AFL in patients with Brugada syndrome or tachycardia–bradycardia syndrome^1^, ^21^	III	C	C2	IVb

* Class III antiarrhythmic drugs are not covered by the National Health Insurance in Japan.

Abbreviations: AFL, atrial flutter; COR, class of recommendation; GOR, grade of recommendation; LOE, level of evidence; MINDS, Medical Information Network Distribution Service.

Radiofrequency catheter ablation is often preferred to long‐term pharmacotherapy for preventing recurrence of AFL, and is considered as a first‐line therapy in patients with CTI‐dependent and non‐CTI‐dependent AFL.^703^, ^709^ For patients in whom ablation is not being considered because of previous unsuccessful procedure or because of patient preference, antiarrhythmic drug therapy is available. The strategy of antiarrhythmic drug therapy in the ongoing management should be also performed on the basis of hemodynamics and organic heart disease in patients with AFL (Figure 21, Table 55).

In AFL patients with normal or mildly reduced cardiac function, Class III antiarrhythmic drugs (e.g., bepridil and sotalol)^710^, ^711^ or Class I antiarrhythmic drugs (Na^+^ channel blockers),^704^, ^705^, ^712^ which can prolong the atrial refractory period and delay the conduction velocity in the AV node through a direct pharmacological effect, are considered as first‐line therapy. However, Class III antiarrhythmic drugs in patients with AFL are not covered by the NHI in Japan. Although these drugs are relatively safe in hemodynamically stable patients with AFL, close monitoring of the QT interval on ECG is required during oral Class III antiarrhythmic drug therapy to evaluate for torsade de pointes, bradycardia or heart failure.

On the other hand, Class I antiarrhythmic drugs, which can inhibit atrial extrasystoles as triggers and delay conduction velocity in the region of the CTI, are also considered as a first‐line therapy. When Class I antiarrhythmic drugs with anticholinergic action, which may lead to a rapid 1 : 1 ventricular response, are orally administered to maintain sinus nodal rhythm in patients with AFL, concomitant *β*‐blockers, diltiazem and verapamil use, which can delay conduction velocity in the AV node, are preferable to achieve favorable rate control in AFL patients.^686^ amiodarone is considered as a second‐line therapy in patients with AFL refractory to the abovementioned antiarrhythmic drugs and has demonstrated favorable long‐term efficacy for preventing recurrence of AFL in Europe and the USA.^703^ However, amiodarone in patients with AFL is not covered by the NHI in Japan. In AFL patients with moderately or severely reduced cardiac function, *β*‐blockers are recommended as a first‐line therapy.^706^, ^707^ Careful ongoing management of *β*‐blockers is especially required to avoid recurrence of heart failure when *β*‐blockers are initially introduced or are adjusted at an appropriate dosage. Amiodarone without negative inotropic action is considered as a second‐line therapy.^80^ Careful ongoing management of amiodarone is also required to avoid occurrence of extracardiac adverse effects.

##### 6.2.2.3 Recommendations for Anticoagulant Therapy of Atrial Flutter

Several observational studies of AFL patients undergoing electrical cardioversion to restore sinus nodal rhythm reported short‐term thromboembolic risks ranging from 1.5% to 2.2%,^680^, ^681^ and the thromboembolic rate is significantly higher in patients with AFL lasting ≥48 h than in those with AFL lasting <48 h.^682^ Anticoagulants should be also given to maintain anticoagulation in the therapeutic range for 4 weeks after cardioversion because of transient deterioration of atrial myocardial contraction following cardioversion, so‐called “atrial stunning”, as a mechanism of thromboembolic complications.^708^, ^713^ In previous studies, atrial stunning has been observed for several weeks following not only pharmacological cardioversion but also electrical cardioversion in patients with AFL and AF. Ongoing management with anticoagulant therapy (warfarin or direct oral anticoagulants) is recommended in patients with AFL to align with recommended anticoagulant therapy for patients with AF. Appropriate anticoagulation therapy should prescribe for 3 weeks before cardioversion and continue at least 4 weeks after cardioversion.^404^

According to several prospective studies and a meta‐analysis,^683^, ^684^, ^685^ the long‐term thromboembolic risks in patients with AFL are comparable to those in patients with AF. Multicenter clinical studies of patients undergoing electrical cardioversion demonstrate a short‐term thromboembolic risk ranging from 1.7% to 7% in patients with AFL and AF,^714^ and the thromboembolic rate in patients with sustained AFL is 3% annually.^692^ Other studies have reported similar efficacy of anticoagulation in patients with AFL.^702^ Therefore, on the basis of available data, recommendations for anticoagulant therapy in patients with AFL are similar to those in patients with AF.

## 7 VENTRICULAR TACHYCARDIA

### 7.1 Epidemiology / Pathophysiology / Electrophysiology

Ventricular tachycardia (VT) is defined as tachycardia originating below the bifurcation of the His bundle. Sustained VT is defined as continuing for at least 30 s or requiring intervention. VT for <30 s is called non‐sustained VT. VT is divided into that occurring in the heart with structural heart disease, and idiopathic VT with no apparent heart disease. The most common forms of idiopathic VT are fascicular left ventricular VT (LF‐VT) and VT originating in the outflow tract (OT‐VT).

#### 7.1.1 Epidemiology

The organic heart diseases in VT with structural heart disease include myocardial infarction (MI), dilated or hypertrophic cardiomyopathy, arrhythmogenic right ventricular cardiomyopathy, congenital heart disease, cardiac sarcoidosis, and post‐cardiac surgery.^715^ In Europe and North America, the most common underlying disease is old MI. However, in Japan, the rate of cardiomyopathy is relatively high.^1^ Sustained VT is a major cause of syncope and sudden death due to arrhythmia. The risk of sudden death increases in patients with reduced cardiac function because of the underlying disease.

Regarding idiopathic VT, LF‐VT is often reported in Asia, including Japan.^716^, ^717^ Both LF‐VT and OT‐VT are likely to occur with physical exercise or mental excitation. In general, idiopathic VT has a better prognosis than VT with structural heart disease.

#### 7.1.2 Pathophysiology / Electrophysiology

Most cases of VT with structural heart disease are caused by reentry, dependent on the scar tissue associated with the organic heart disease.^718^ Cardiomyocytes are surrounded by fibrous tissue in the scar area, which causes conduction disturbance and formation of a reentry circuit.^719^, ^720^ Infiltration of adipocytes also contributes to the formation of reentry circuits.^721^ Sustained VT with structural heart disease typically presents as a monomorphic wide QRS tachycardia, and is often evoked and terminated by programmed stimulation.

Idiopathic LF‐VT is caused by abnormal Purkinje fibers in part of the reentry circuit.^722^, ^723^, ^724^ Because it is known that abnormal Purkinje fibers have decremental conduction properties and verapamil sensitivity, LF‐VT is also called verapamil‐sensitive VT.^725^ A bundle branch reentry is considered another type of VT originating from the bundle branch and Purkinje fibers.^726^ These VTs have an anatomically fixed reentry circuit and can be diagnosed as reentry by electrophysiological study.

The focus of OT‐VT is distributed through the left and right ventricular OTs, the left ventricular epicardium, the cusp of the aortic valve, the pulmonary artery, and the basal septum, although the most frequent focus is in the right OT.^727^, ^728^, ^729^ Most cases of OT‐VT are catecholamine‐dependent, and present with multiple monomorphic premature ventricular contractions and recurrent non‐sustained VT. It has been reported that OT‐VT is caused by abnormal automaticity or triggered activity.^730^, ^731^

### 7.2 Idiopathic Ventricular Tachycardia

Idiopathic VT is defined as the VT without any apparent structural heart diseases, diagnosed by physical examination, 12‐lead ECG, echocardiography and cardiac magnetic resonance imaging. Patients with idiopathic VT generally have a preferable prognosis, and the indication of pharmacological therapy based on the patient’s preference, symptoms etc. The tachycardia‐induced cardiomyopathy is a reversible myocardial dysfunction due to frequent extrasystoles or tachycardia. Therapeutic options should be considered in this form of cardiomyopathy even without symptoms of tachycardia.^732^

**Figure 22:**
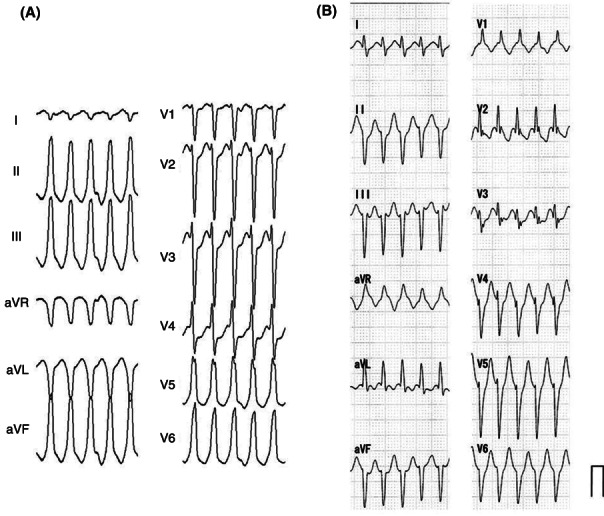
The 12‐lead ECGs during typical idiopathic VT. (**A**) Idiopathic VT with left bundle branch block and right axis deviation, (**B**) Idiopathic VT with right bundle branch block and left axis deviation. ECG, electrocardiogram; VT, ventricular tachycardia.

**Figure 23:**
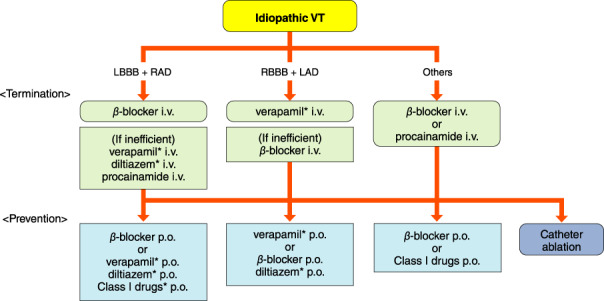
Choice of antiarrhythmic drugs based on the ECG morphology of VT. Intravenous (i.v.) drugs should be injected gradually, and oral (p.o.) drugs should be started with a lower dose. *No insurance reimbursement in Japan. ECG, electrocardiogram. LAD, left axis deviation; LBBB, left bundle branch block; RAD, right axis deviation; RBBB, right bundle branch block; VT, ventricular tachycardia.

The origin and the mechanism of VT can be predicted by the QRS morphology during VT in typical idiopathic VT (Figure 22). The mode of onset (during activity or rest), ambulatory ECG recording and exercise ECG are helpful for the decision of antiarrhythmic drugs (Figure 23, Table 56).

**Table 56:** Recommendations and Levels of Evidence for Treatment of Idiopathic VT

	COR	LOE	GOR (MINDS)	LOE (MINDS)
Use of *β*‐blocker for the symptomatic VT without structural heart disease and/or inherited arrhythmia	IIa	C	C1	IVa
Use of non‐dihydropyridine calcium antagonist instead of *β*‐blocker for VT without organic heart disease	IIa	C	C1	IVa
Use of Class I antiarrhythmic drugs for calcium antagonist‐ and *β*‐blocker‐refractory VT	IIb	C	C1	IVa

COR, class of recommendation; GOR, grade of recommendation; LOE, level of evidence; MINDS, Medical Information Network Distribution Service; VT, ventricular tachycardia.

#### 7.2.1 Acute and Chronic Management of Idiopathic Ventricular Tachycardia

The origin of idiopathic VT is predominantly in the area of the right ventricular OT, left ventricular OT, aortic sinus, tricuspid annulus, mitral annulus, and papillary muscle.^733^ The most frequent form of 12‐lead ECG during VT shows left bundle branch block morphology and right deviation (inferior axis), which originates from the OT (Figure 22A). The mechanism of this form of VT is mainly abnormal automaticity or triggered activity, and OT‐VT usually shows a non‐sustained and repetitive form. The mechanism of triggered activity is delayed afterdepolarization, which is responsible for the Ca^2+^ channel current. Antiarrhythmic drugs that inhibit the Ca^2+^ channel current, such as *β*‐blockers or non‐dihydropyridine Ca^2+^ channel blockers, may be effective for OT‐VT. These drugs are administered by bolus injection to terminate VT, and then orally to prevent recurrence. Class I antiarrhythmic drugs (Na^+^ channel blocker), such as propafenone, are an alternative choice if patients do not have reduced cardiac and renal function.^74^, ^76^, ^734^, ^735^, ^736^

VT with right bundle branch block and left deviation (superior axis) is idiopathic reentrant VT (idiopathic left ventricular tachycardia; ILVT), related to the Purkinje network around the left bundle branch (Figure 22B). This VT is characterized as verapamil‐sensitive. Verapamil is effective for terminating and preventing this VT. Intravenous injection of 5 mg of verapamil usually terminates VT. Higher doses (>5 mg) of verapamil are sometime required to terminate VT if it has been sustained for a long time and the intrinsic adrenergic activity is high. The other form of idiopathic VT, such as papillary muscle VT, should be considered in the case of verapamil non‐sensitive VT.^723^ *β*‐blockade is effective at inhibiting papillary muscle VT. There is no specific pharmacological approach for the other forms of idiopathic VT. *β*‐blocker or Class I antiarrhythmic drug is empirically administered.

#### 7.2.2 Treatment for Radical Cure

Avoidance of aggravating factors, such as excessive consumption of caffeine, smoking and alcohol, may be sufficient for mild symptoms.^737^ Catheter ablation can be a highly effective treatment with high acute success rate and low recurrence rate during long‐term follow‐up.^4^, ^76^, ^737^ If catheter ablation fails or is not applicable, pharmacological therapy should be considered.

### 7.3 Ventricular Tachycardia Associated With Organic Heart Disease

VT can occur in patients with organic heart disease such as MI, dilated cardiomyopathy, hypertrophic cardiomyopathy, arrhythmogenic right ventricular cardiomyopathy, post‐myocarditis, and congenital heart diseases, including Fallot’s tetralogy. In those cases, cardiac function is mostly deteriorated by the cardiac disease, and tachycardic attacks are more likely to occur in patients with more severe deterioration. It is very common for those patients to become hemodynamically unstable at the time of the VT attacks, and the first treatment is to terminate the tachycardia. Table 57 shows treatment of recommendations and levels of evidence for VT associated with organic heart disease.^4^

**Table 57:** Recommendations and Levels of Evidence for Therapy for VT Associated With Organic Heart Disease

	COR	LOE	GOR (MINDS)	LOE (MINDS)
Immediate direct‐current defibrillation in the patients with sustained hemodynamically unstable VT	I	B	A	III
Use of ICD for recurrent VT and prevention of sudden cardiac death	I	A	A	I
Catheter ablation for drug‐resistant VT due to IHD*	I	B	A	II
Intravenous administration of amiodarone or nifekalant for resuscitation in patients with sustained or recurrent hemodynamically unstable VT after direct‐current defibrillation	IIa	A	B	II
Intravenous administration of procainamide for termination of hemodynamically stable monomorphic sustained VT	IIa	A	B	II
Use of oral amiodarone or sotalol for recurrence of VT	IIa	A	A	II
Intravenous administration of amiodarone for termination of hemodynamically stable polymorphic sustained VT	IIb	A	C1	II

^*^ Indication of catheter ablation is the same as in the “Guideline for Non‐pharmacotherapy of Cardiac Arrhythmias”.^3^

Abbreviations: COR, class of recommendation; GOR, grade of recommendation; ICD, implantable cardioverter‐defibrillator; IHD, ischemic heart disease; LOE, level of evidence; MINDS, Medical Information Network Distribution Service; VT, ventricular tachycardia.

#### 7.3.1 Termination of Ventricular Tachycardia

**Figure 24:**
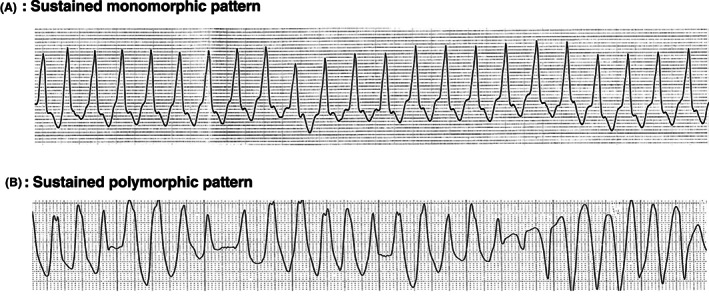
Monitoring ECGs of sustained ventricular tachycardia associated with organic heart disease. ECG, electrocardiogram. (Adapted from Ikeda T 2011.^738^)

**Figure 25:**
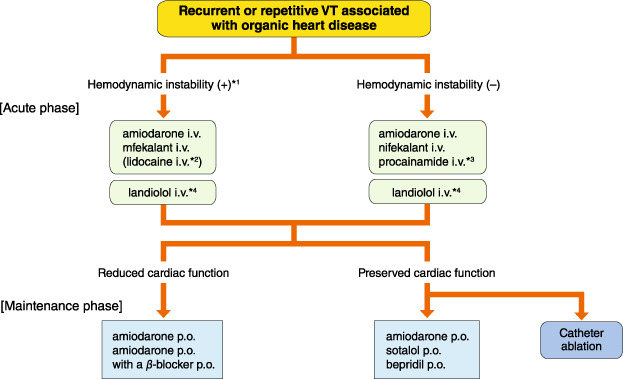
Drug selection for the purpose of termination and prevention of sustained VT associated with organic heart disease. *^1^In the cases of sustaining hemodynamically unstable tachycardia, the drug should be administered where immediate direct current defibrillation can be performed. *^2^Alternative drug when no other antiarrhythmic drug is available. *^3^The drug should be used only for the monomorphic sustained VT. *^4^The drug should be initiate from low dose and gradually increase for sustained VT. VT, ventricular tachycardia.

Sustained VT associated with organic heart disease has different ECG findings depending on whether it is monomorphic or polymorphic (Figure 24).^738^ If the patient is hemodynamically unstable with VT, perform direct‐current defibrillation according to the procedure of cardiopulmonary resuscitation, and consider intravenous administration of amiodarone or nifekalant as an antiarrhythmic drug (Figure 25).^739^, ^740^, ^741^, ^742^ When these drugs are ineffective or unavailable, consider intravenous lidocaine as an alternative.^739^, ^743^ Emergency catheter ablation may be indicated for frequent recurrent monomorphic VT and non‐sustained VT. Because reentry is usually the main mechanism of sustained VT, pacing stimulation from the ventricle can be used to terminate it in many cases.

In patients with frequent VT after implantation of an implantable cardioverter‐defibrillator (ICD), intravenous administration of an antiarrhythmic drug, such as nifekalant in Japan,^744^ may be performed when using sedation and anesthesia is being considered.

If the status is hemodynamically stable, drug treatment can be considered after 12‐lead ECG recording and careful observation of hemodynamics. At the same time, proceed with a detailed evaluation of the organic heart disease from physical and laboratory findings. When an antiarrhythmic drug is intravenously administered with ECG monitoring, attention should be paid to blood pressure, bradycardia, and QRS width prolongation until the tachycardia terminates. Always keep in mind the possibility of sudden changes, and apply direct‐current defibrillation immediately the hemodynamics deteriorate due to drug administration, etc.

Intravenous administration of amiodarone, nifekalant, or procainamide is considered a first‐line drug therapy.^745^, ^746^, ^747^, ^748^, ^749^ If the tachycardia is stable monomorphic sustained VT, intravenous procainamide should be administered preferentially.^745^, ^746^, ^747^, ^748^ Randomized controlled trials (RCTs) in other countries, meta‐analyses, and retrospective studies in Japan have shown that procainamide is significantly superior to lidocaine in terminating monomorphic VT.^745^, ^746^, ^747^ In addition, procainamide is superior to amiodarone, both for terminating tachycardia and less adverse events, according to the RCTs, which targeted approximately 80% of cases of VT related to organic heart disease with cardiac dysfunction.^748^ Another study reported that amiodarone was significantly superior to lidocaine in terminating tachycardia and 1‐day survival.^749^ On the other hand, another retrospective study has reported that amiodarone had limited acute effect on terminating VT in patients with ischemic heart disease (IHD) and cardiac dysfunction,^750^ which suggests the acute effect of terminating VT by amiodarone is not been well established. With nifekalant, VT was terminated in 52.8% of the Japanese study patients.^744^ Although lidocaine is not recommended for monomorphic VT,^745^, ^746^, ^747^, ^749^, ^751^ it may be selected as an alternative drug when amiodarone, nifekalant, and procainamide cannot be used or the patient is in the acute phase of MI.^752^ Intravenous drip infusion of landiolol, a very short‐acting *β*_1_‐blocker, is recommended for recurrent VT associated with organic heart disease in which antiarrhythmic drugs such as amiodarone, and nifekalant are ineffective.^753^, ^754^ It should be intravenously administered with gradually increasing dose while monitoring hemodynamics and ECG. Intravenous administration of sotalol, which is not available in Japan, has been shown to be significantly superior to terminating tachycardia compared with lidocaine.^745^, ^751^

#### 7.3.2 Prevention of Ventricular Tachycardia

Prevention of VT associated with organic heart disease is classified into primary prevention to prevent the onset of symptoms, and secondary prevention to prevent recurrence of attacks. As sustained VT is directly related to prognosis, positive consideration to preventing it should be given. First, fully evaluate whether there is any possibility that VT is associated with reversible factors such as ischemia, electrolyte abnormality, and drugs. When myocardial ischemia causes tachycardia, coronary revascularization is strongly considered. When drug administration is tried to prevent tachycardia, the drug with fewest side effects should be selected because of the need for long‐term administration.

##### 7.3.2.1 Secondary Prevention

Implantation of an ICD is the first choice for preventing recurrent hemodynamically unstable VT.^755^, ^756^, ^757^

Even if VT is hemodynamically stable, unstable VT can occasionally occur later in patients with organic heart disease such as IHD with cardiac dysfunction. An ICD is recommended in such cases for preventing poor prognosis.^758^, ^759^ Drug therapy is used when an ICD cannot be implanted or when it reduces the occurrence of VT in an ICD‐implanted patient. Whether or not an antiarrhythmic drug is used for an ICD‐implanted patient depends on the case and the facility’s policy. Drugs that can terminate VT do not always have the effect of preventing recurrence.

Amiodarone and sotalol are mainly used to prevent recurrence of VT.^84^, ^760^, ^761^ Bepridil or *β*‐blocker may be used in some cases. Generally, amiodarone is used without evaluating drug effect by electrophysiological study.^755^ No one knows if drug selection based on electrophysiological study prevents recurrence of VT.^762^ According to a Japanese trial, amiodarone administration (150 or 200 mg/day) based on electrophysiological study was effective in preventing tachycardia in patients with relatively preserved cardiac function (left ventricular ejection fraction [LVEF] 30–50%).^763^ On the other hand, it did not work in the patients with LVEF <30%.^763^ A multicenter RCT clarified that sotalol significantly reduces both mortality and the need for ICD operation.^764^ In addition, it has been reported that the combination of amiodarone and *β*‐blocker significantly reduced the need for ICD operation compared with administration of *β*‐blocker or sotalol alone.^84^

Electrical storm (ES) patients should be managed in hospital. Amiodarone or nifekalant is generally used to suppress the recurrence of arrhythmias, and a combination of amiodarone with propranolol significantly suppresses the occurrence of events compared with amiodarone plus metoprolol.^765^ In cases where these drugs are ineffective, Na^+^ channel blockers can be used while checking their effectiveness. Cather ablation is recommended when arrhythmia control is difficult even with the abovementioned drugs.^766^, ^767^, ^768^ At this time, do not forget the use of pharmacotherapy to prevent deterioration of the organic heart disease.

##### 7.3.2.2 Primary Prevention

Holter‐ECG monitoring in out‐of‐hospital cardiac arrest and sudden death, and the effectiveness of ICDs in primary prevention of sudden death clarify that VT is closely associated with sudden death. However, the prognostic effect of ICDs in primary prevention varies according to the type of organic heart disease. In patients with cardiac dysfunction after MI, ICD therapy has been shown to have a significant prognostic effect in comparison with conventional medical therapy.^769^, ^770^, ^771^ On the other hand, although sudden death from fatal arrhythmia is suppressed by an ICD,^772^ there is no significant difference in total death between ICD therapy and amiodarone or conventional medical therapy in cases of dilated cardiomyopathy.^771^, ^772^, ^773^, ^774^ However, ICD therapy improved prognosis compared with conventional medical therapy in dilated cardiomyopathy patients who have NYHA III heart failure.^772^

Na^+^ channel blockers did not improve the prognosis, even though frequent ventricular premature contractions and non‐sustained VT associated with MI increase the risk of sudden death. In a meta‐analysis of post‐MI and heart failure cases, amiodarone reduced sudden arrhythmic death by 29% and total death by 13%.^775^ However, a subsequent RCT showed no significant difference in survival between amiodarone and placebo in either IHD or non‐IHD.^771^ Moreover, according to a recent meta‐analysis, amiodarone had no favorable effect on overall mortality, although it suppressed sudden death and cardiovascular death.^761^

Angiotensin‐converting enzyme inhibitors and aldosterone blockers, as well as *β*‐blockers, are known to improve mortality, including sudden death, among patients with congestive heart failure.

### 7.4 Polymorphic Ventricular Tachycardia in Cases Without QT Prolongation

**Figure 26:**
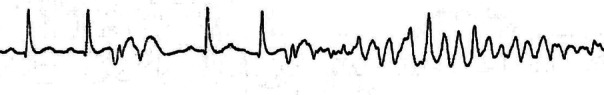
Ambulatory ECG Monitoring at the time of initiation of short‐coupled TdP. ECG, electrocardiogram; TdP, torsade de pointes.

Polymorphic VT (PMVT) often occurs during acute myocardial ischemia, often degenerating into ventricular fibrillation (VF). PMVT is documented more frequently than monomorphic VT under such conditions.^776^, ^777^ Apart from acute ischemia, as the clinical entity that promotes PMVT, PMVT has been triggered by ectopic firing from the right ventricular outflow tract (RVOT) and from left Purkinje fibers.^63^, ^778^, ^779^, ^780^ The coupling interval (CI) of the triggering premature ventricular contraction (PVC) from the Purkinje fibers has been shown to be shorter and the QRS width to be narrower, as compared with the triggering PVC from the RVOT.^63^, ^781^ PMVT that is induced by PVC with a CI <300 ms is termed short‐coupled variant of TdP (Figure 26).^63^, ^781^, ^782^ Recommendations and levels of evidence for therapy are shown in Table 58.^781^, ^782^, ^783^, ^784^, ^785^, ^786^

**Table 58:** Recommendations and Levels of Evidence for Pharmacological Therapy for PMVT and Short‐Coupled TdP

	COR	LOE	GOR (MINDS)	LOE (MINDS)
Intravenous administration of *β*‐blocker for the bail‐out of PMVT storm when it occurs during the subacute phase ≥72 h after the onset of ACS^783^	IIa	B	B	II
Use of prophylactic *β*‐blocker for prevention of short‐coupled TdP if triggered by PVC arising from the RVOT^784^, ^785^	IIa	C	C1	V
Use of verapamil for prophylaxis of short‐coupled TdP in patients without any ischemic or structural disease^782^	IIb	C	C1	V
Use of quinidine for prophylaxis of short‐coupled TdP in patients with any ischemic or structural disease^781^, ^786^	IIb	C	C1	V

ACS, acute coronary syndrome; COR, class of recommendation; GOR, grade of recommendation; LOE, level of evidence; MINDS, Medical Information Network Distribution Service; PMVT, polymorphic ventricular tachycardia; PVC, premature ventricular contraction; RVOT, right ventricular outflow tract; TdP, torsade de pointes.

#### 7.4.1 Termination of Polymorphic Ventricular Tachycardia

Direct‐current (DC) shock using biphasic waveform with >150 J should be done for the termination of PMVT, and subsequent cardiopulmonary resuscitation should be initiated according to advanced cardiac life support.^787^ Amiodarone or nifekalant should be administered for PMVT refractory to DC shock.^740^, ^741^, ^742^, ^787^ *β*‐blockers might be effective for bail‐out of PMVT that repeatedly appears during the subacute phase of acute coronary syndrome (ACS).^783^ PMVT that occurs after relieving ischemia during the subacute phase of ACS or after percutaneous coronary intervention for chronic coronary syndrome is subject to progressing to ES. Quinidine has potential to terminate such PMVT.^788^

#### 7.4.2 Prophylaxis for Recurrent Polymorphic Ventricular Tachycardia

The pharmacological approach to preventing short‐coupled TdP developing under the condition of neither ischemic nor structural heart disease has not been established, as there have been only retrospective cohort studies with small numbers of the patients, in which the preventive effects of *β*‐blockers, Ca^2+^ channel blockers, and quinidine on short‐coupled TdP were reported.^781^, ^782^, ^784^, ^785^, ^786^, ^788^

## 8 POLYMORPHIC VENTRICULAR TACHYCARDIA / TORSADE DE POINTES

### 8.1 Congenital Long QT Syndrome

**Figure 27:**
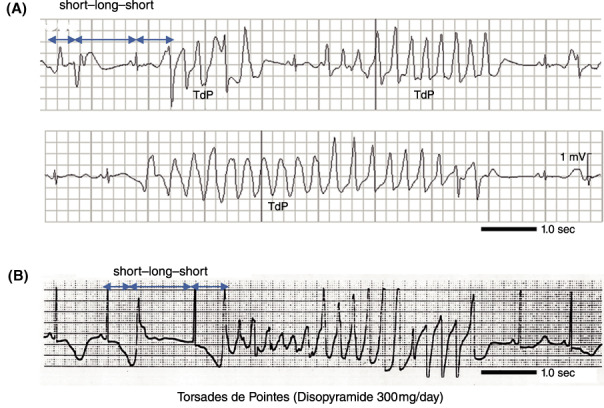
(**A**) Monitoring ECGs of polymorphic ventricular tachycardia (Torsade de Pointes) with syncope in a patient with LQT2. Change in the R‐R interval (short–long–short) followed by polymorphic ventricular tachycardia, TdP, characterized as a twisting QRS axis. (**B**) Secondary (Drug) induced QT prolongation. Drug‐induced TdP due to oral disopyramide (300 mg/day). A similar short–long–short change in R‐R followed by TdP. ECG, electrocardiogram; LQT2, long QT syndrome type 2; TdP, torsade de pointes.

Long QT syndrome (LQTS) is characterized by QT interval prolongation on ECG and polymorphic ventricular tachycardia (VT), named torsade de pointes (TdP), leading to syncope and sudden cardiac death.^789^, ^790^ Thus, when we look at a representative ECG of TdP, we should speculate the patient may have LQTS (Figure 27A).

LQTS is conventionally classified as congenital (genetic) or secondary (acquired). Secondary LQTS is determined by the QT interval not being prolonged under normal conditions but is significantly prolonged by secondary factors such as drugs and bradycardia (Figure 27B). However, recent studies have shown that nearly 30% of patients with secondary LQTS have the same genetic abnormalities as those with congenital LQTS,^791^ so secondary LQTS can be thought as a concealed type of congenital LQTS. Therefore, for either congenital or secondary LQTS, the pharmacological treatment is almost the same. Furthermore, pharmacological treatment of congenital LQTS is divided into 1) acute treatment at TdP occurrence based on QT interval prolongation, and 2) prevention of TdP and cardiac arrest or sudden death at non‐acute phase (preventive medicine).

#### 8.1.1 Acute Treatment at Torsade de Pointes Occurrence

Most cases of TdP spontaneously terminate and patients usually feel dizzy, lightheadedness and faintness (loss of consciousness); however, if it progresses to ventricular fibrillation (VF) without termination, immediate cardiopulmonary resuscitation and electrical defibrillation are required.

To suppress and prevent recurrence of TdP in the acute phase, intravenous injection of magnesium sulfate (bolus of 30–40 mg/kg over 5–10 min, i.e. 2 g of magnesium sulfate [1 ampoule] for adult weighing 60 kg) is recommended, and if effective, continuous infusion of 3–20 mg/min^793^ (in children: 0.05–0.3 mg/kg/min). Intravenous infusion of a *β*‐blocker (propranolol or landiolol) is also effective,^794^ and in some patients, antiarrhythmic drugs (lidocaine or mexiletine) or Ca^2+^ channel antagonists (verapamil) may be effective in suppressing TdP.^795^, ^796^

When bradycardia exacerbates the QT interval prolongation and occurrence of TdP, temporary pacing to increase the heart rate is very effective. Hypokalemia often promotes development of TdP, thus maintain the serum potassium level >4.0 mEq/L as much as possible.

#### 8.1.2 Preventive Treatment for Torsade de Pointes

**Figure 28:**
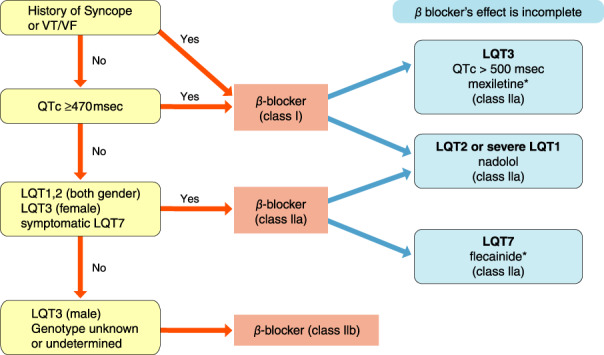
Chronic pharmacological treatment for prevention of TdP in congenital LQTS. *Add to *β*‐blockers. LQTS, long QT syndrome; TdP, torsade de pointes; VF, ventricular fibrillation; VT, ventricular tachycardia.

*β*‐blockers are effective for suppression of cardiac events in 74% of LQT1 and 63% of LQT2,^798^, ^799^ and are the first therapeutic choice for most cases of the congenital LQTS. Oral *β*‐blocker is a Class I indication for patients with a history of syncope or VT/VF, but even if asymptomatic, *β*‐blocker is a Class I indication for patients with QTc interval ≥470 ms (especially in LQT1 or LQT2). In case with asymptomatic and QTc <470 ms, oral *β*‐blocker may be recommended as a Class IIa indication in LQT1, LQT2 (both sexes) and female LQT3.^800^ Oral *β*‐blocker can be used (recommendation Class IIb) in asymptomatic male LQT3 with QTc <470 ms, or genetically unknown or undetermined cases^801^ (Table 59, Figure 28).

**Table 59:** Recommendations and Levels of Evidence for *β*‐Blockers in Congenital LQTS

	COR	LOE	GOR (MINDS)	LOE (MINDS)
Use of *β* blocker for the patients with history of syncope, VT/VF	I	B	A	IVa
Use of *β* blocker for the patients with asymptomatic, QTc ≥470 ms	I	B	B	IVa
Use of *β* blocker for the patients with asymptomatic, QTc <470 ms, LQT1,2 (both sexes) or LQT3 (female)	IIa	B	B	IVa
Use of nadolol for LQT2 or high‐risk LQT1	IIa	C	C1	IVa
Use of *β* blocker for the patients with asymptomatic, QTc <470 ms, male LQT3 or Genotype unknown or undermined	IIb	C	C1	IVa

COR, class of recommendation; GOR, grade of recommendation; LOE, level of evidence; LQTS, long QT syndrome; MINDS, Medical Information Network Distribution Service; VF, ventricular fibrillation; VT, ventricular tachycardia.

Moreover, *β*‐1 receptor non‐selective *β*‐blockers, such as propranolol and nadolol, are more effective than the *β*‐1‐receptor selective *β*‐blockers such as atenolol and metroprolol.^802^ Particularly in LQT2 patients, nadolol is more recommended than other *β*‐blockers.^803^, ^804^ LQT3 is caused by increased late *I*_Na_ due to a gain‐of‐function mutation in the *SCN5A* gene, and the late *I*_Na_ inhibitor mexiletine can abbreviate the QT interval and may be effective in suppressing cardiac events.^805^, ^806^, ^807^, ^808^ Flecainide is effective in LQT7 (Andersen‐Tawil syndrome), as well as in catecholaminergic polymorphic ventricular tachycardia (CPVT).^809^

The Ca^2+^ channel blocker, verapamil, suppresses early afterdepolarization and development of TdP in the acute phase,^795^, ^796^ so might be effective as a supportive therapy in patients who still have recurrence of events despite optimal *β*‐blocker therapy. Verapamil can be effective in a case of LQT8 (Timothy syndrome) caused by a gain‐of‐function mutation in the *CACNA1C* gene;^810^ however, there was no evidence of suppression of events by oral Ca^2+^ channel blockers over a long‐term follow‐up.

Hypokalemia exacerbates the increase in the QT interval, so potassium containing drugs is sometimes prescribed as a supportive treatment in LQTS. Maintaining the serum potassium level >4.0 mEq/L may be effective in suppressing cardiac events^811^, ^812^ (Table 60).

**Table 60:** Recommendations and Levels of Evidence for Other Pharmacological Treatment Except for *β*‐Blockers in Congenital LQTS

	COR	LOE	GOR (MINDS)	LOE (MINDS)
Additional mexiletine in LQT3 and QTc >500 ms	IIa	B	B	IVa
Additional flecainide in LQT7	IIa	B	C1	IVa
Additional potassium containing drugs chloride in hypokalemia (K<4.0 mEq/L)	IIa	C	C1	V
Additional Ca^2+^ channel blocker (verapamil) for high‐risk LQTS or LQT8	IIb	C	C1	V

COR, class of recommendation; GOR, grade of recommendation; LOE, level of evidence; LQTS, long QT syndrome; MINDS, Medical Information Network Distribution Service.

In congenital LQTS, TdP is sometimes induced by drugs. Therefore, in either congenital or acquired LQTS, patients should not take any medicine that has a potential to prolong the QT interval (Table 61).

**Table 61:** Causes of Acquired Long QT Syndrome

(1) Drugs:
**Antiarrhythmic drugs**
Class IA (quinidine, disopyramide, procainamide, cibenzoline, etc.)
Class IC (flecainide)
Class III (sotalol, nifekalant, amiodarone, etc.)
Class IV (bepridil)
**Antibiotics** (macrolides, new quinolone, trimethoprim/sulfamethoxazole, etc.)
**Antifungals** (itraconazole, etc.)
**Antiallergic drugs** (hydroxyzine, hydrochloride, etc.)
**Antihyperlipidemic drug** (probucol, etc.)
**Psychotropic drugs** (haloperidol, chlorpromazine, etc.)
**Tricyclic antidepressants** (imipramine, amitriptyline, etc.)
**Antiulcer drug** (famotidine, sulpiride, etc.)
**Antiemetics** (domperidone, etc.)
**Anticancer drug** (doxorubicin, etc.)
**(2) Bradycardia:**
atrioventricular block, sick sinus syndrome, pauses after the termination of atrial fibrillation, etc.
**(3) Electrolyte abnormalities:**
hypokalemia, hypomagnesemia, hypocalcemia
**(4) Cardiac diseases:**
acute myocardial infarction, left ventricular hypertrophy, stress cardiomyopathy (takotsubo cardiomyopathy)
**(5) Intracranial disorders:**
subarachnoid hemorrhage, brain bleeding, central nervous system diseases
**(6) Endocrine and nutritional disorders:**
hypothyroidism, adrenal dysfunction, anorexia nervosa
**(7) Inflammatory disorders:**
myocarditis, Chagas’ disease, rheumatic heart disease, collagen disease
**(8) Other:**
female gender, elderly, malnutrition, hypothermia, liver failure, HIV infection

#### 8.1.3 Acute Treatment for Torsade de Pointes

**Figure 29:**
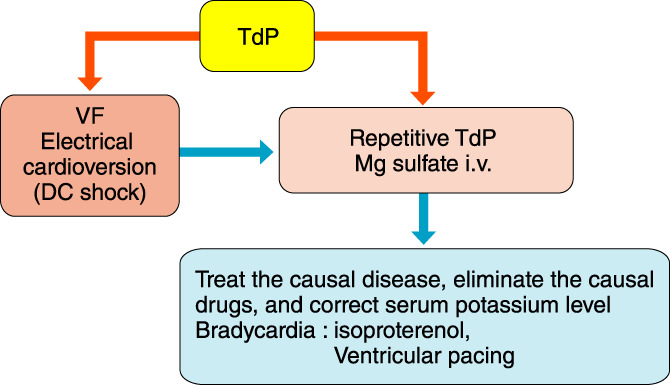
Acute pharmacological treatment for TdP in congenital or acquired LQTS. DC, direct current; LQTS, long QT syndrome; TdP, torsade de pointes; VF, ventricular fibrillation.

Please refer to the acute therapy of secondary (acquired) LQTS (Figure 29).

### 8.2 Acquired Long QT Syndrome

Regarding the treatment of acquired LQTS, the first priority is to remove any factor that is prolonging the QT interval, and the second is to treat any primary disease that may be inducing prolongation of the QT interval. It is important to remove secondary factors, as shown in Table 61; for example, withdraw a culprit drug, conduct the pacing therapy for bradycardia and correct hypokalemia. In cases of TdP, physicians must treat the patient as an emergency as described below and need to follow‐up the patient under continuous ECG monitoring on hospitalization until the causative factor can be removed and TdP is no longer a risk (Figure 29).

(1) Magnesium sulfate i.v.: bolus of 30–40 mg/kg over 5–10 min, and if effective, an infusion of 3–20 mg/min in adults or 1–5 mg/min (0.05–0.3 mg/kg/min) in children. Magnesium sulfate is effective for the prevention of TdP,^793^, ^813^ but physicians should consider reducing or ceasing magnesium sulfate administration if adverse effects occurred. Carefully observe the patient’s physical condition, and control the dosage under monitoring of the plasma concentration of magnesium sulfate because patients with renal dysfunction or elderly patients can easily become hypermagnesemia.

(2) Infusion of isoproterenol: 0.5–5 *μ*g/min (0.1–1 *μ*g/kg/min in children). The infusion rate should be adjusted to keep heart rate >100 beats/min; however, this therapy should be used as a bridge therapy to temporary pacing.^814^ Moreover, this therapy is not recommended for patients with congenital LQTS because of its exacerbating effect to QT interval prolongation.^815^

(3) Serum potassium is adjusted to maintain within 4.5∼5.0 mEq/mL.^792^ Patients need to be kept ≥70 beats/min under overdrive pacing for the prevention of TdP induced by a short–long–short sequence pattern.^814^, ^816^

## 9 VENTRICULAR FIBRILLATION AND VENTRICULAR TACHYCARDIA ASSOCIATED WITH SPECIAL DISEASES

### 9.1 Brugada Syndrome and Early Repolarization Syndrome

Several hypotheses of the mechanism underlying the specific ECG manifestation and ventricular fibrillation (VF) have been proposed for Brugada syndrome (BrS) and early repolarization syndrome (ERS). One of the major theories reported by experimental studies relies on a prominent transient outward current (*I*_to_) in epicardial cells but not in endocardial cells, which creates a transmural voltage gradient and thus causes J–ST‐segment elevation.^817^ Prominent *I*_to_‐mediated dispersion of repolarization also leads to the development of reentrant arrhythmia. Despite still being controversial, this hypothesis suggests that reduced *I*_to_ and increased inward current (*I*_Na_, *I*_Ca_) can decrease J–ST‐segment elevation and suppress reentrant arrhythmia. In fact, the pharmacologic mechanism of therapeutic agents that are effective in BrS and ERS is quite similar to this hypothesis.

Pharmacologic therapy has been reported to suppress the occurrence of ventricular tachyarrhythmia accompanied by attenuation of specific ECG change in BrS and ERS, despite still lacking studies of high evidence level.^818^, ^819^, ^820^, ^821^, ^822^, ^823^, ^824^, ^825^ Because this section conforms to the “Guidelines for Diagnosis and Management of Inherited Arrhythmias” (JCS 2017 revised),^5^ please also refer to the medical care flowchart in those guidelines.

#### 9.1.1 Brugada Syndrome

Implantable cardioverter‐defibrillator (ICD) is the first‐line treatment for preventing sudden cardiac death in BrS, and at present, pharmacologic therapy is essentially an adjunctive treatment (Tables 62 and 63).

**Table 62:** Recommendations and Levels of Evidence of Pharmacologic Therapy for Preventing Recurrence of VF in Brugada Syndrome

	COR	LOE	GOR (MINDS)	LOE (MINDS)
isoproterenol infusion* for suppression of VF storm	IIa	C	B	IVb
Use of quinidine in patients with frequent VF episodes	IIa	C	B	IVb
Use of quinidine in patients with ICD indication but refusal or contraindication	IIb	C	B	III
Use of bepridil or cilostazol* in patients with frequent VF episodes	IIb	C	B	V

^*^ Not covered by insurance in Japan.

COR, class of recommendation; GOR, grade of recommendation; ICD, implantable cardioverter‐defibrillator; LOE, level of evidence; MINDS, Medical Information Network Distribution Service; VF, ventricular fibrillation.

(Adapted from JCS, 2017.^5^)

**Table 63:** Pharmacologic Therapy in Brugada Syndrome

	Classification	Pharmacologic mechanism	Administration method	Dose
isoproterenol*	*β*‐stimulant	*I*_Ca_↑ *I*_to_↓ due to an increase in heart rate	Intravenous	1–2 *μ*g bolus injection followed by 0.15 *μ*g/min or 0.003–0.006 *μ*g/kg/min
quinidine	Class Ia	*I*_to_↓	Oral	300–600 mg/day
cilostazol*	Phosphodiesterase III inhibitor	*I*_Ca_↑ due to cAMP↑	Oral	200 mg/day
bepridil	Class IV	*I*_Na_↑, *I*_to_↓	Oral	100–200 mg/day

* Not covered by insurance in Japan. *I*_Na_, *I*_Ca_, inward currents; *I*_to_, transient outward current.

(Adapted from JCS, 2017.^5^)

##### 9.1.1.1 Acute Treatment

Isoproterenol (ISP), which increases the L‐type *I*_Ca_ and reduces *I*_to_, followed by increased heart rate, has been reported to be useful for suppressing frequent VF episodes, including electrical storm, in BrS (not covered by insurance in Japan). As reported from Japan, intravenous ISP is recommended to be administered as a bolus injection (1–2 *μ*g) followed by continuous infusion (0.15 *μ*g/min) or a continuous infusion (0.003–0.006 *μ*g/kg/min).^818^, ^819^

##### 9.1.1.2 Chronic Treatment

Oral pharmacologic therapy can be considered in patients with frequent ICD discharges due to VF, including electrical storm, as a chronic treatment.

###### 9.1.1.2.1 Quinidine

In Europe and the USA, quinidine of 600–900 mg/day is recommended for the prevention of VF;^826^, ^827^ however, the usual dose in Japan is 300–600 mg/day. Careful attention is necessary to the occurrence of side effects such as gastrointestinal,^828^ and it is not recommended as an alternative to ICD because of the uncertainty of the prevention of sudden death.^820^

###### 9.1.1.2.2 Cilostazol (Not Covered by Insurance in Japan)

Cilostazol, a phosphodiesterase III inhibitor, has been shown to suppress VF, most likely by augmentation of the Ca^2+^ channel current (*I*_Ca_), as well as by reduction of *I*_to_ secondary to an increase in heart rate.^821^, ^829^

###### 9.1.1.2.3 Bepridil

Bepridil, a Ca^2+^ channel antagonists, blocks multiple K^+^ channels, including *I*_to_. Long‐term administration of bepridil has been reported to increase the Na^+^ channel current. These pharmacologic mechanisms should prevent spontaneous VF episodes.^830^ It is usually effective at a dose of 200 mg/day, but patients with *SCN5A* mutation are reported to show an effect even at a dose of 100 mg/day.^822^ Combination with cilostazol is also reported to be effective.^823^

In addition, some reports suggest the effectiveness of sotalol,^831^ disopyramide,^832^ and denopamine^818^; however, the studies had small numbers of cases and sufficient evidence is lacking.

#### 9.1.2 Early Repolarization Syndrome

Similar to BrS, ICD is the first‐line treatment for prevention of sudden cardiac death, and pharmacologic therapy is only an adjunct treatment in ERS. There are fewer studies and reports on pharmacologic therapy for ERS than for BrS, and the evidence level is low (Table 64).

**Table 64:** Recommendations and Levels of Evidence of Pharmacologic Therapy for Preventing Recurrence of VF in Early Repolarization Syndrome

	COR	LOE	GOR (MINDS)	LOE (MINDS)
Use of isoproterenol infusion* in suppressing VF storm	IIa	C	B	IVb
Use of quinidine in patients with frequent VF episodes	IIa	C	B	IVb
Use of quinidine in patients with ICD indication but refusal or contraindication	IIb	C	C1	VI
Use of bepridil or cilostazol* in patients with frequent VF episodes	IIb	C	B	V

* Not covered by insurance in Japan.

COR, class of recommendation; GOR, grade of recommendation; ICD, implantable cardioverter‐defibrillator; LOE, level of evidence; MINDS, Medical Information Network Distribution Service; VF, ventricular fibrillation.

ERS and BrS are reported to share similarities with respect to the response to pharmacologic therapy. Intravenous infusion of ISP is the most useful treatment for suppression of electrical storm as in BrS, and quinidine is highly useful as an oral medication for chronic treatment.^824^ Quinidine can be considered for prevention of VF in patients with ICD indication but without consent of ICD implantation, but its long‐term efficacy is unknown. Gastrointestinal side effects such as nausea and diarrhea caused by quinidine are known to be common in Japanese patients, thus caution is required when it is administered. The phosphodiesterase III inhibitor cilostazol (not covered by insurance) and bepridil are also reported to be effective.^825^, ^833^ The combination of cilostazol and bepridil is also reported to be effective.^823^

### 9.2 Catecholaminergic Polymorphic Ventricular Tachycardia

**Figure 30:**
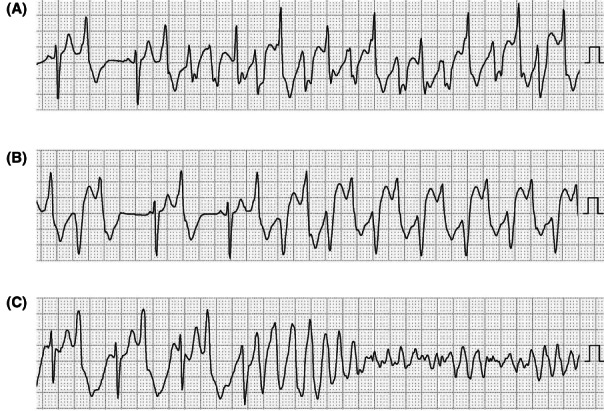
Monitoring ECGs at VT in patients with CPVT: (**A**) polymorphic VT, (**B**) bidirectional VT, (**C**) polymorphic VT leading to ventricular fibrillation. CPVT, catecholaminergic polymorphic ventricular tachycardia; ECG, electrocardiogram; VT, ventricular tachycardia. (Adapted from Sumitomo N et al. 2016.^823^)

Catecholaminergic polymorphic ventricular tachycardia (CPVT) is a relatively rare but potentially lethal ventricular arrhythmia^834^, ^835^ (Figure 30, ^836^). The diagnostic criteria are shown in Table 65.^5^, ^801^

**Table 65:** CPVT Diagnostic Criteria of CPVT

1. CPVT is diagnosed with normal ECG, structurally normal heart, and bidirectional VT and polymorphic PVCs induced by exercise or administration of a catecholamine in a patient under 40 years of age
2. CPVT is diagnosed in patients (including index case or family member) who have a pathogenic gene mutation
3. CPVT is diagnosed in family members of a CPVT index case without organic heart disease who have exercise‐induced PVCs or bidirectional/polymorphic VT
4. CPVT can be diagnosed with normal ECG, structurally normal heart and coronary arteries, and polymorphic PVCs by exercise or administration of catecholamine in a patient over 40 years of age
1, 2, and 3 are confirmed; 4 is suspicion
Abbreviations: CPVT, catecholaminergic polymorphic ventricular tachycardia; ECG, electrocardiogram; PVC, premature ventricular contraction; VT, ventricular tachycardia. (Modified from Priori SG, et al^801^ and Aonuma K, et al.^5^)

Several genetic mutations associated with CPVT are reported. CPVT1 is the most common genetic mutation, encoding the cardiac ryanodine receptor (*RyR2*).^837^, ^838^ CPVT2 is the second most common genetic mutation, encoding calsequestrin2 (*CASQ2*).^839^, ^840^, ^841^, ^842^ These genetic mutations lead to abnormal Ca^2+^ handling and Ca^2+^ overload in the cytoplasm from the sarcoplasmic reticulum. Consequently, various ventricular tachycardias or ventricular fibrillation can develop due to delayed afterdepolarization. It is reported that about 1/7 to 1/8 of all unexplained sudden cardiac deaths might be caused by CPVT.^843^

#### 9.2.1 Treatment

##### 9.2.1.1 Termination of Ventricular Arrhythmias

Adenosine triphosphate,^836^, ^844^ verapamil,^835^ and intravenous *β*‐blockers^792^ are useful to terminate bidirectional/polymorphic VT. Class IA, IB, and III drugs are usually ineffective on terminating CPVT.^792^, ^847^, ^848^ Deep sedation^843^, ^847^, ^848^ are very effective in controlling VT storm, whereas electrical cardioversion is often ineffective to terminate VT, and might exacerbate VT by pain; however, electrical defibrillation might terminate ventricular fibrillation in CPVT patients (Table 66).

**Table 66:** Recommendations and Levels of Evidence for Acute Therapeutic Intervention for CPVT

	COR	LOE	GOR (MINDS)	LOE (MINDS)
Deep sedation	I	C	B	V
Infusion of *β*‐blockers	IIa	C	B	V
Infusion of verapamil or adenosine triphosphate	IIb	C	C1	V

Abbreviations: COR, class of recommendation; CPVT, catecholaminergic polymorphic ventricular tachycardia; GOR, grade of recommendation; LOE, level of evidence; MINDS, Medical Information Network Distribution Service.

##### 9.2.1.2 Prevention of Lethal Arrhythmias

Patients with CPVT are recommended to change their lifestyle (strong restriction of exercise and stress avoidance), and the use of high‐dose *β*‐blockers^801^, ^835^, ^849^, ^850^ and verapamil^835^ for the prevention of fatal arrhythmias were recommended. Recently, flecainide has given priority for patients who are insufficiently controlled by *β*‐blockers.^801^, ^849^, ^850^ Although *β*‐blockers are the first‐line treatment for CPVT, the rate of arrhythmic recurrence and the occurrence of fatal events during 4 years after initiation of *β*‐blockers were reported as 19% and 3%, respectively.^851^ Flecainide should be added to *β*‐blockers in patients with a definite diagnosis or strong clinical suspicion (Table 67).

**Table 67:** Recommendations and Levels of Evidence for Prophylactic Therapeutic Intervention for the Ventricular Tachyarrhythmias of CPVT

	COR	LOE	GOR (MINDS)	LOE (MINDS)
Lifestyle counseling recommended for all patients with diagnosis of CPVT: limitation or avoidance of competitive sports, strenuous exercise, and stressful environments	I	C	B	IVa
*β*‐blockers in all symptomatic patients with diagnosis of CPVT				
Use of flecainide in addition to *β*‐blockers patients with diagnosis of CPVT who experience syncope or polymorphic/bidirectional VT while on *β*‐blockers	IIa	C	B	III
Use of *β*‐blockers in carriers of a pathogenic CPVT mutation without clinical manifestation of CPVT (concealed mutation‐positive patients)	IIa	C	C1	IVa
Use of flecainide monotherapy in patients with diagnosis of CPVT who have difficulty with *β*‐blockers for any reason	IIb	C	C1	V

Abbreviations: COR, class of recommendation; CPVT, catecholaminergic polymorphic ventricular tachycardia; GOR, grade of recommendation; LOE, level of evidence; MINDS, Medical Information Network Distribution Service; VT, ventricular tachycardia.

To confirm medication compliance and to evaluate drug efficacy, repeat studies of Holter or exercise ECG are inevitable.

###### 9.2.1.2.1 *β*‐Blockers

Nadolol has been reported as the most effective *β*‐blockers to prevent ventricular arrhythmias in patients with CPVT,^852^, ^853^ although some reported a negative effect.^854^ Carvedilol has been reported directly suppress *RyR2*,^855^ but there is no report that carvedilol is superior to the other *β*‐blockers in a clinical trial.

###### 9.2.1.2.2 Flecainide

Flecainide is strongly recommended in CPVT patients without control of ventricular arrhythmia by *β*‐blockers.^856^, ^857^, ^858^ Flecainide monotherapy is also reported to be effective,^854^, ^859^ and may be useful in patients who have difficulty with taking *β*‐blockers because of intolerance or side effects. Propafenone is also reported to suppress the ryanodine receptor, and might be useful to prevent ventricular arrhythmia in CPVT patients.^860^

###### 9.2.1.2.3 Verapamil

Although verapamil combined with *β*‐blocker has been reported as effective in a small case series,^861^, ^862^ clinical evidence of prophylactic effect is still limited.^854^

### 9.3 Other Inherited Arrhythmias (Short QT Syndrome)

#### 9.3.1 Short QT Syndrome

Short QT syndrome (SQTS) is a rare inherited arrhythmia characterized by markedly shortened QT intervals, atrial fibrillation (AF), ventricular tachycardia (VT), ventricular fibrillation (VF), episodes of syncope and even sudden cardiac death (SCD). Accoriding to the HRS/EHRA/APHRS expert consensus statement on the diagnosis and management of patients with inherited primary arrhythmia syndromes., SQTS is diagnosed in the presence of (1) QT ≤330 msec, or (2) QT <360 ms and one of the following conditions: presence of SQTS mutation, family history of SQTS, family history of sudden cardiac death which occured less than 40 years old, and VT/VF without organic heart disease.^863^ The diagnosis is based on the QT interval, clinical symptoms, family history, and genetic mutations. On the other hand, in the 2015 European Society of Cardiology (ESC) guidelines, SQTS is (1) diagnosed in the presence of QTc ≤340 ms (Class I), and (2) should be considered in the presence of a QTc ≤360 ms and ≥1 of the following: (a) confirmed pathogenic mutation, (b) family history of SQTS, (c) family history of SCD at age <40 years, (d) surviving a VT/VF episode in the absence of organic heart disease (Class IIa).^850^ Mutations in 7 genes encoding the K^+^ channels (*KCNH2*, *KCNQ1*, and *KCNJ2*), L‐type Ca^2+^ channels (*CACNA1C*, *CACNB2b*, and *CACNA2D1*), and the Cl^−^/HCO^3−^ exchanger (*SLC4A3*) have hitherto been reported in SQTS. An ICD is the most effective treatment for preventing sudden death in high‐risk patients, and is recommended in patients with a diagnosis of SQTS who (a) are survivors of an aborted cardiac arrest, and/or (b) have documented spontaneous sustained VT (Class I).^850^, ^863^

#### 9.3.2 Drug Therapy

The purpose of pharmacologic therapy in patients with SQTS is to prevent concomitant AF and ventricular arrhythmias. In patients with recurrent ventricular arrhythmias during ICD treatment, or in patients who are eligible for ICD treatment but for whom it cannot be done for any reason, pharmacologic treatment should be considered to prevent VF. Many previous reports have shown the effectiveness of quinidine and it is considered as the first‐line pharmacologic treatment of SQTS.^864^, ^865^, ^866^, ^867^

In 2017, Mazzanti et al^867^ reported the inhibitory effect of quinidine on arrhythmic events in 17 SQTS cases. Two patients discontinued the drug due to gastrointestinal symptoms, and the remaining 15 patients received quinidine (584 ± 53 mg/day) for approximately 6 years. Quinidine treatment prolonged the QTc interval by a mean of 60 ms. No fatal arrhythmic events were observed in the quinidine group during the follow‐up period, and the annual rate of cardiac arrest before and after treatment improved from 12% to 0%. Quinidine has a high pharmacological affinity for the activated state of IKr channels, while other IKr inhibitors such as sotalol have a high affinity for the inactivated state of the channels.^868^ Therefore, quinidine may have a greater effect on QT prolongation than sotalol in SQTS type 1 (e.g., *KCNH2*‐N588K mutation), which is enhanced IKr channel function.^865^, ^869^ In small cohorts of patients, disopyramide,^870^, ^871^ nifekalant^872^ or a combination of both^873^ have been reported to prolong the QTc interval; isoproterenol^874^ and amiodarone^875^ have been reported to inhibit VF; and propafenone has been reported to inhibit AF.^876^ Accoring to the HRS/EHRS/APHRS expert consensus statement in 2013, the use of quinidine or sotalol is considered as Class IIb recommendation for the patients with asymptomatic SQTS who had a family history of SCD.^863^ In the 2015 ESC guidelines, quinidine or sotalol may be considered in asymptomatic patients with a diagnosis of SQTS and a family history of SCD, patients who qualify for an ICD but present a contraindication to the ICD or refuse it (Class IIb).^850^ However, because of the lack of evidence for sotalol, only quinidine is recommended in this guideline (Table 68).

**Table 68:** Recommendations and Levels of Evidence for Pharmacologic Treatment of SQTS

	COR	LOE	GOR (MINDS)	LOE (MINDS)
Use of quinidine in patients with SQTS who experience cardiac arrest or sustained VT and have a contraindication of ICD implantation	IIb	C	C1	IVa
Use of quinidine in patients with SQTS who have a family history of sudden cardiac death	IIb	C	C1	IVa

Abbreviations: COR, class of recommendation; GOR, grade of recommendation; ICD, implantable cardioverter‐defibrillator; LOE, level of evidence; MINDS, Medical Information Network Distribution Service; SQTS, short QT syndrome; VT, ventricular tachycardia.

## 10 VENTRICULAR FIBRILLATION / PULSELESS VENTRICULAR TACHYCARDIA / CARDIAC ARREST

### 10.1 Treatment

**Figure 31:**
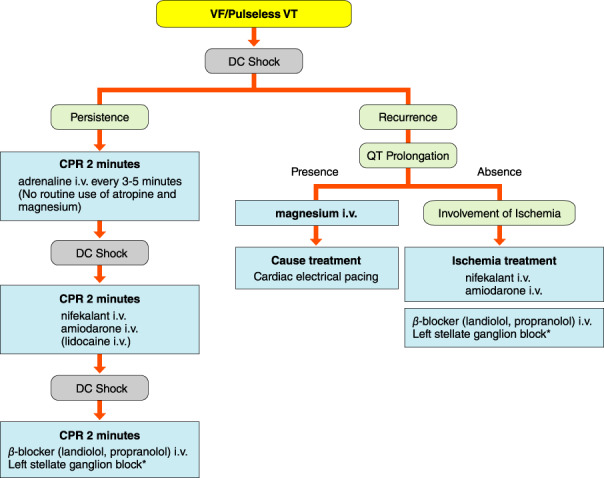
Treatment flowchart for VF/pulseless VT. *Please refer to Figure 32. CPR, cardiopulmonary resuscitation; DC, direct‐current; VF, ventricular fibrillation; VT, ventricular tachycardia.

**Figure 32:**
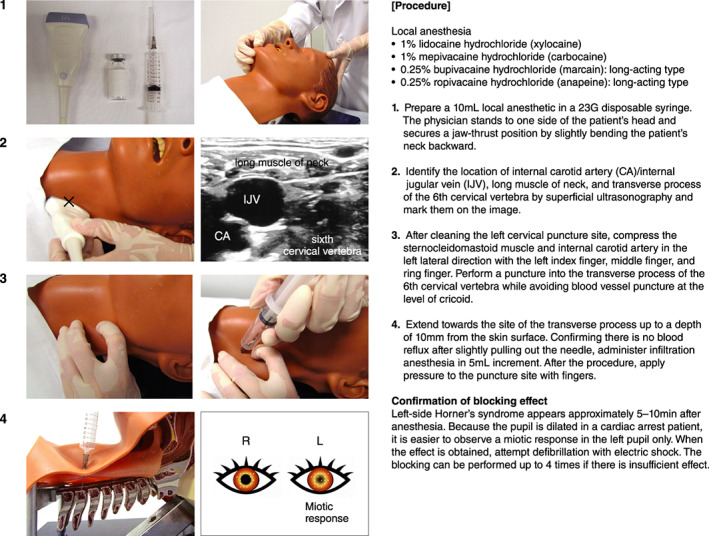
Procedure of left stellate ganglion block for refractory VT/VF. CA, carotid artery; IJV, internal jugular vein; VF, ventricular fibrillation; VT, ventricular tachycardia.

Because ventricular fibrillation (VF)/pulseless ventricular tachycardia (pVT) are extremely severe arrhythmias causing rapid decline in cardiac output and possibly leading to unconsciousness and cardiac arrest, they require immediate cardiopulmonary resuscitation (CRP). Figure 31 shows the treatment flowchart. Concomitant use of vasopressin^877^, ^878^ and routine use of magnesium are not recommended.^879^, ^880^ However, magnesium may be considered in cases of polymorphic VT associated with QT prolongation.^881^ Atropine is not recommended for routine use in either pulseless electrical activity (PEA) or asystole,^881^ but such usage should be considered in the case of ineffective adrenaline administration for asystole.^882^ Figure 32 shows the method for performing left stellate ganglion block against refractory VT/VF.

### 10.2 Antiarrhythmic Therapy

Antiarrhythmic drugs are associated with return of spontaneous circulation (ROSC), but there is no proof of long‐term survival or favorable neurological outcome. According to a systematic review/meta‐analysis of 17 previous reports (10 reports from randomized controlled trials and 7 reports from observational studies), amiodarone injection, lidocaine, and nifekalant can be effective for obtaining ROSC, but had no improvement on survival discharge.^883^ In a recent systematic review/meta‐analysis (30 previous reports, 39,914 patients), there was no conclusive evidence that amiodarone, lidocaine, esmolol, nifekalant, sotalol, magnesium, or vasopressin may possibly improve ROSC, survival admission, survival discharge, and neurological outcome, and only nifekalant achieved significantly more cases of survival admission compared with lidocaine.^740^

According to a multicenter cohort study in Japan, the groups administered nifekalant, amiodarone, or lidocaine indicated higher 1‐month survival rates compared with the non‐administration group, but neurological outcomes remain unknown.^884^ Table 69 shows recommendation for injection and evidence level at the time of CPR.^739^, ^740^, ^741^, ^742^, ^743^, ^753^, ^754^, ^783^, ^879^, ^880^, ^881^, ^882^, ^883^, ^884^, ^885^, ^886^, ^887^, ^888^, ^889^, ^890^, ^891^, ^892^, ^893^, ^894^, ^895^, ^896^, ^899^, ^900^, ^901^

**Table 69:** Recommendations and Levels of Evidence for Injection at the Time of CPR for VF/pVT

	COR	LOE	GOR (MINDS)	LOE (MINDS)
nifekalant administration to obtain ROSC for VF/pVT with no response to CPR^740^, ^741^, ^742^, ^883^, ^884^, ^885^, ^886^, ^887^, ^888^, ^889^, ^890^, ^891^	IIa	B	B	I
amiodarone administration to obtain ROSC for VF/pVT with no response to CPR^739^, ^740^, ^741^, ^743^, ^883^, ^884^, ^885^, ^892^, ^893^, ^894^, ^895^	IIa	A	B	I
magnesium administration for polymorphic VT associated with QT prolongation^901^	IIa	B	B	III
Sympathetic block treatment for ES and antiarrhythmic drug‐resistant VF/pVT^753^, ^754^, ^783^, ^896^, ^899^	IIa	C	B	III
lidocaine administration to obtain ROSC for VF/pVT with no response to CPR^739^, ^740^, ^743^, ^883^	IIb	B	C2	I
Prophylactic routine administration of lidocaine for VF/pVT after ROSC^900^	IIb	B	C1	IVa
Routine administration of *β*‐blocker (p.o./i.v. injection) in early stage after ROSC^901^	IIb	C	C1	IVb
Routine administration of magnesium for adult VF/pVT^879^, ^880^	III	A	D	I
Routine administration of atropine for PEA and asystole^881^, ^882^	III	B	D	IVa

Abbreviations: COR, class of recommendation; CPR, cardiopulmonary resuscitation; ES, electrical storm; GOR, grade of recommendation; ICD, implantable cardioverter‐defibrillator; LOE, level of evidence; MINDS, Medical Information Network Distribution Service; PEA, pulseless electrical activity; pVT, pulseless ventricular tachycardia; ROSC, return of spontaneous circulation; VF, ventricular fibrillation; VT, ventricular tachycardia.

#### 10.2.1 Amiodarone

Although amiodarone was effective for survival admission in the 2015 guideline presented by the American Heart Association,^743^, ^892^ it could not obtain favorable survival discharge and neurological outcome compared with placebo in a large double‐blind randomized controlled trial presented in 2016.^739^ Although there were similar results in meta‐analyses,^893^, ^894^ survival discharge did not indicate more favorable outcomes compared with lidocaine in an analysis of the administrative claim database of Japan.^895^ According to a multicenter cohort study and review in Japan, amiodarone 125–150 mg can be superior to 300 mg in terms of efficacy and reduction in side effects.^741^, ^885^ Thus, the appropriate initial dose of amiodarone might be ≤150 mg.

#### 10.2.2 Nifekalant

Nifekalant has a greater defibrillation effect than lidocaine or other conventional treatments.^742^, ^886^, ^887^, ^888^ According to a multicenter cohort study in Japan, nifekalant or amiodarone improved the 24‐h survival rate to the same degree.^741^ On the basis of an administrative claim database study^889^ and a single‐center prospective study^890^ in Japan, there was no difference in ROSC between nifekalant and amiodarone, but nifekalant could potentially improve survival admission. As one of the reasons, the time to successful defibrillation was shorter than with amiodarone.^890^, ^891^ Thus, there are more reports in Japan indicating the usefulness of nifekalant in such cases.

#### 10.2.3 Lidocaine

In a large‐scale clinical study of out‐of‐hospital cardiac arrest cases, lidocaine was inferior to amiodarone in survival admission but showed no difference in survival discharge.^743^ The large‐scale clinical trials described previously did not indicate either a higher rate of survival discharge or favorable neurological outcomes compared with placebo.^739^

#### 10.2.4 *β*‐Blockers

It is reported that sympathetic block treatment (e.g., esmolol intravenous injection, propranolol intravenous injection, and stellate ganglion block) could be more effective than conventional antiarrhythmic drugs against electrical storm (ES) after acute myocardial infarction.^783^ The clinical studies in Japan, such as J‐Land II, reported some usefulness of landiolol intravenous injection against nifekalant/amiodarone‐resistant ES.^753^, ^754^ Furthermore, it was also indicated that left stellate ganglion block could be effective for nifekalant‐resistant VF/pVT in the case of patients with out‐of‐hospital cardiac arrest.^896^ Stellate ganglion block is a method of administering infiltration anesthesia to sympathetic ganglion after confirming the 6th cervical vertebra at the level of the cricoid cartilage by ultrasound examination of the neck.^897^, ^898^ It is effective as a means of treating VF/pVT that cannot be controlled by conventional antiarrhythmic drugs.^899^

### 10.3 Treatment by Antiarrhythmic Drugs After Return of Spontaneous Circulation

Although there is an observational study describing suppression of recurrence by lidocaine in terms of antiarrhythmic drug administration as a preventive, it did not indicate a significant effect on survival admission or survival discharge in the propensity score analysis.^900^ It is reported that administration of early *β*‐blocker (oral or intravenous injection) after ROSC could increase chances of long‐term survival,^901^ but it also may possibly cause hemodynamic instability, worsening of heart failure, and bradyarrhythmia.

## 11 ARRHYTHMIAS IN PEDIATRICS

The mechanisms of most arrhythmias in pediatric patients are the same as in adult patients. However, prevalence, natural history, and expression of symptoms of arrhythmias differ in children according to their age. As a result, treatment strategies also differ between adults and children. Typical symptoms during tachycardia in infants are vomiting, poor feeding, and respiratory distress, and in preschool children, chest or abdominal pain are the most common symptoms. In school‐age children, palpitations, and chest discomfort are the most common, but some children are first diagnosed with arrhythmias in school heart screening without any symptoms.

Indications of catheter ablation for tachyarrhythmias in children are expanding to infants and perioperative patients with congenital heart disease, consequently the importance of antiarrhythmic medications for tachycardia has relatively declined, but they still remain valuable therapy in some pediatric patients for bridge therapy to catheter ablation or with difficulty to perform catheter ablation in some reason.

This guideline is based on the “Guideline for Antiarrhythmic Drugs” developed by the Japanese Society of Pediatric Cardiology,^902^ and other local and foreign guidelines^4^, ^5^, ^699^, ^801^, ^849^, ^850^ for children.

The evidence for antiarrhythmic drug therapy in children is much less than for adults, and most of the studies have been retrospective, small in size, and observational. As a result, most of the indications and dosages of antiarrhythmic drugs in children are basically determined according to those in adults, and only a few drugs have determined indications and dosages after clinical trials in children. For these reasons, the use of antiarrhythmic drugs in children largely depends on the experience of the physician. In this guideline, pediatric patients are defined as those under 15 years of age.

### 11.1 Narrow QRS Tachycardia

#### 11.1.1 Mechanism and Diagnosis

Narrow QRS tachycardia is clinically equal to paroxysmal supraventricular tachycardia (SVT) and includes the following diagnoses: atrioventricular reciprocating tachycardia (AVRT), AV nodal reentrant tachycardia (AVNRT), atrial tachycardia (AT), and intra‐atrial reentrant tachycardia. AVRT accounts for more than half of the cases in preschool children, whereas AVNRT is more common in school‐aged children.^903^

#### 11.1.2 Acute Treatment of Narrow QRS Tachycardia

##### 11.1.2.1 Termination of Tachycardia^904^, ^905^

**Figure 33:**
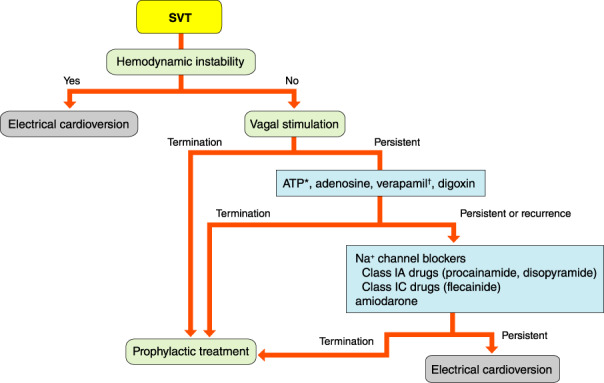
Flow chart to terminate SVT in children. *Off‐label. ^†^Contraindicated for neonates and infants. ATP, adenosine triphosphate; SVT, supraventricular tachycardia.

In neonates and infants, sustained or repetitive tachycardia is likely to result in heart failure, because they cannot complain of palpitations; therefore, the tachycardia should be terminated as soon as possible (Figure 33).

###### 11.1.2.1.1 Non‐Pharmacotherapy

In critically ill children with tachycardia, if it is not determined to be SVT, synchronized electrical cardioversion (0.5–2.0 J/kg) should be performed during cardiopulmonary resuscitation if necessary. In hemodynamically stable children with tachycardia, vagal stimulation (e.g., breath‐holding, ice immersion, carotid sinus massage) should be performed, and if these are not effective, antiarrhythmic drugs should be administered. Transesophageal atrial overdrive pacing can also be useful in neonates and infants, and vagal reflexes by insertion of an esophageal catheter may terminate tachycardia in some patients.

###### 11.1.2.1.2 Pharmacotherapy

The tachycardia circuit of most SVTs includes the AV node. Therefore, bolus intravenous administration of adenosine triphosphate (ATP), which transiently suppresses AV nodal conduction, is useful for the diagnosis and treatment of SVT.^906^, ^907^, ^909^, ^910^ The effect of ATP is augmented by the use of dipyridamole, and attenuated by the use of xanthine derivatives. In addition, we need to pay attention to bronchospasm in patients with asthma. Due to the short half‐life of ATP in blood, ATP should be administered bolus injection as rapidly as possible, followed by an adequate amount of physiological saline or 5% glucose solution infusion.

Ca^2+^ channel blocker (verapamil)^911^, ^913^ or digoxin is administered intravenously in recurrent cases. Verapamil may be given in older children but is contraindicated in small infants because it may lead to cardiovascular collapse.^911^, ^914^ In AVRT, slow intravenous administration of a Class IC (flecainide) or IA (procainamide and disopyramide) antiarrhythmic drug is also recommended because of the prolonged refractory period of the accessory pathways. Amiodarone is also recommended when other drugs are ineffective. It may take some time to terminate tachycardia after injection of amiodarone, and one may need to pay attention to hypotension, especially in infants and neonates.^915^

#### 11.1.3 Prophylactic Treatment of Narrow QRS Tachycardia (Figure 34)

**Figure 34:**
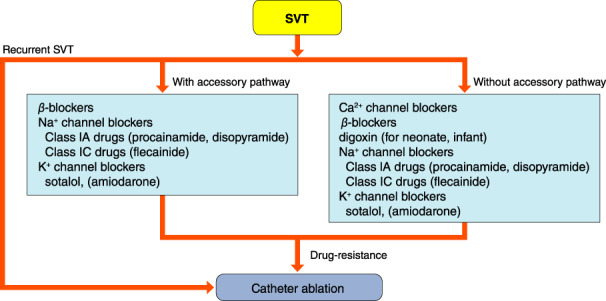
Prophylactic treatment of SVT in children. SVT, supraventricular tachycardia.

The indication for prophylactic treatment of arrhythmia is repetitive or long‐lasting tachycardia. Newborns and infants are likely to develop heart failure, because the tachycardia is usually noticed only after the patient becomes quite ill. Therefore, antiarrhythmic drugs should be administered to prevent tachycardia.

In recent years, the selection of antiarrhythmic drugs for recurrent SVT has shifted towards Class IC or III drugs away from digoxin and *β*‐blockers.^849^ Combinations of these drugs are effective for SVT that is refractory to monotherapy,^916^ but interactions and arrhythmogenesis with multiple antiarrhythmic drugs also need to be considered. If the SVT is recurrent after 1 year of age, it is unlikely to resolve spontaneously,^917^ and long‐term drug management should be considered according to the severity of the tachyarrhythmia. In patients with short duration and spontaneously terminated tachycardia without organic heart disease or pre‐excitation syndrome, antitachycardia medication is usually unnecessary. Children with a history of tachycardia, or their guardians, should be educated in how to terminate the tachycardia with appropriate vagal stimulation maneuvers according to their ages.

The safety and efficacy of catheter ablation in children has improved, and it should be considered for children aged >5 years, or body weight >15 kg, who require continuous antiarrhythmic medication. However, the indication of catheter ablation should be carefully determined by the type and origin of the arrhythmia, and body size. On the other hand, a single oral dose to be taken only during an attack may be recommended in cases of ineffective of vagal nerve stimulation procedures. However, the indication of antiarrhythmic medication should be restricted in children with severe left ventricular dysfunction and/or sinus bradycardia. The efficacy of a single oral dose of verapamil, propranolol^110^ or sotalol^918^ has been reported.

#### 11.1.4 Treatment for Each Type of Supraventricular Tachycardia

##### 11.1.4.1 Atrioventricular Nodal Reentrant Tachycardia

The frequency of AVNRT is <10% in infants and toddlers, but higher in patients over 5 years of age. The indication of catheter ablation is determined by age, body size, severity of symptoms, frequency and duration of tachyarrhythmia, efficacy and side effect of antiarrhythmic drugs, and the presence of concomitant cardiac disease. Catheter ablation is the first‐line treatment for older children because the efficacy of pharmacotherapy is 30–50%, and the risk of AV block associated with catheter ablation is low in older children.^4^, ^849^

The standard prophylactic medication for AVNRT in children has been Ca^2+^ channel blockers and *β*‐blockers. The monotherapy with atenolol was reported to be effective in 59% of adolescents with AVNRT.^919^ The combination of *β*‐blocker and Class IC drug was preferred in patients without sufficient suppression of AV nodal conduction and no structural heart disease.^849^

##### 11.1.4.2 Accessory Pathway‐Mediated Tachycardias

Pre‐excitation syndrome can cause AVRT via accessory pathway, rapid ventricular conduction via antegrade accessory pathway with atrial fibrillation (AF),^920^ and decreased ventricular function associated with ventricular dyssynchronous contractions.^921^ The onset of AVRT in children doublepeaks in infants or teenagers. Over 90% of patients with pre‐excitation syndrome diagnosed in infancy show improvement of the tachycardia by 18 months of age.^922^, ^923^ However, those without improvement tend to have persistent tachycardia episodes (Table 70).^924^

**Table 70:** Recommendations and Levels of Evidence for Prophylactic Therapeutic Intervention for Recurrent Tachyarrhythmias due to WPW Syndrome in Pediatric Patients

	COR	LOE	GOR (MINDS)	LOE (MINDS)
Patients with aborted sudden cardiac death, syncope or decreased cardiac function, and body weight ≥15 kg				
Catheter ablation	I	C	C1	V
Use of Class IC drugs (e.g., flecainide)	IIa	C	C1	V
Use of *β*‐blockers	IIb	C	C2	VI
Patients with recurrent persistent SVT induced on electrophysiology study, or palpitations, and body weight ≥15 kg				
Catheter ablation	I	C	C1	V
Use of Class IC drugs	I	C	C1	V
Use of sotalol	IIa	C	C1	V
Use of amiodarone	IIb	C	C2	VI
Patient with recurrent and/or symptomatic SVT, and body weight <15 kg				
Use of Class IC drugs	I	C	B	IVa
Use of sotalol, Class IA drugs (e.g., disopyramide)	IIa	C	C1	V
Use of *β*‐blocker, amiodarone	IIb	C	C1	V
Catheter ablation	IIb	C	C1	V
Asymptomatic patient				
Arrhythmic drug therapy	III	C	D	V

Abbreviations: COR, class of recommendation; GOR, grade of recommendation; LOE, level of evidence; MINDS, Medical Information Network Distribution Service; SVT, supraventricular tachycardia; WPW, Wolff‐Parkinson‐White.

If the accessory pathway has a short anterograde refractory period, rapid conduction to the ventricles may occur during AF. The incidence of sudden cardiac death in a cohort of pediatric and adult patients with pre‐excitation syndrome was 0.0025, 0.0000, and 0.0015 per patient‐year for symptomatic, asymptomatic, and the overall patients, respectively.^925^, ^926^, ^927^

The risk factors for complications of catheter ablation in pediatric patients used to have been reported as young age (<5 years), and low body weight (≤15 kg).^903^, ^928^, ^929^ Catheter ablation is the first‐line treatment for pre‐excitation syndrome in older children with tachycardia, and pharmacotherapy is recommended as the first‐line therapy in children under 5 years of age with recurrent SVT.^4^, ^849^ Therefore, long‐term management with antiarrhythmic drugs is still recommended in infants and older children who are a high risk for catheter ablation.

The results of pharmacotherapy for SVT in infants and toddlers have been reported in different clinical situations such as age, dosages of antiarrhythmic drugs, and association of organic heart disease.^657^, ^930^, ^931^, ^932^, ^933^, ^934^ In children with pre‐excitation syndrome, Class IC, IA, or III drugs are preferred to prolong the refractory period of the accessory pathway, especially in cases of a short effective refractory period.

The efficacy of flecainide is reported to be 73–100%,^933^, ^934^ with no deaths, and <1% of serious proarrhythmic events.^933^ The efficacy of sotalol is reported to have an efficacy be 64–94%,^935^, ^936^, ^937^, ^938^ and ≈10% of proarrhythmic events such as sinus atrial block, advanced AV block, and torsade de pointes (TdP).^937^ Reports of the use of amiodarone for AVRT are limited, and none has demonstrated that amiodarone is superior to Class IC medications or sotalol from the efficacy and safety standpoints. Amiodarone should be administered to patients with uncontrollable tachycardia by several conventional antiarrhythmic drugs and with a high risk for catheter ablation.^849^

In addition, one should keep in mind that the use of drugs to suppress AV nodal conduction in children with WPW syndrome has a risk of rapid conduction to the ventricles during AF.^939^

##### 11.1.4.3 Junctional Ectopic Tachycardia

Junctional ectopic tachycardia (JET) mainly presents in 2–10% of pediatric patients in the early postoperative period after surgery for the following congenital heart diseases (CHDs): ventricular septal defect, AV septal defect, tetralogy of Fallot, complete transposition of the great arteries, and the Norwood operation.^940^ Although JET usually recovers spontaneously within a few days, it can be fatal in the early postoperative period due to hypotension and hemodynamic collapse. Therefore, aggressive and intensive treatment for JET may be required, including extracorporeal membrane oxygenation (Table 71).

**Table 71:** Recommendations and Levels of Evidence for Acute Therapeutic Intervention for Junctional Ectopic Tachycardia in Pediatric Patients

	COR	LOE	GOR (MINDS)	LOE (MINDS)
Reduce dose or discontinue catecholamine, atrial overdrive pacing, hypothermia, deep sedation	I	C	C1	V
Extracorporeal membrane oxygenation for patients with hemodynamic collapse	I	C	C1	V
Intravenous administration of amiodarone	I	C	C1	V
Intravenous administration of landiolol	IIa	C	C1	V
Intravenous administration of nifekalant, procainamide	IIb	C	C1	V
Intravenous administration of flecainide, digoxin				

Abbreviations: COR, class of recommendation; GOR, grade of recommendation; LOE, level of evidence; MINDS, Medical Information Network Distribution Service.

Postoperative JET is treated with combinations of reducing dose or discontinuation of catecholamine, atrial overdrive pacing, hypothermia, deep sedation, and antiarrhythmic drugs. Although amiodarone has been reported to decrease mortality from 35% to 4%,^941^, ^942^, ^943^, ^944^ monotherapy resulted in termination of JET in only 11%, and usually requires a combination of non‐pharmacological therapy.^941^

In Japan, procainamide and nifekalant were commonly used before the introduction of amiodarone. The efficacy of nifekalant manifests more immediately, it has a shorter half‐life, and there is less chance of hypotension than with amiodarone; however, nifekalant has more chance of QT prolongation and TdP than amiodarone. Landiolol is also reported as an effective medication to suppress JET.^945^ If these antiarrhythmic medications are not sufficient to control JET, additional treatment with digoxin and flecainide might be effective.^849^ Intraoperative and postoperative administration of dexmedetomidine might reduce the occurrence of postoperative JET.^946^, ^947^ On the other hand, congenital or non‐surgical JET might be controlled by single use or combination of amiodarone, *β*‐blocker, and Class IC drug.^940^

##### 11.1.4.4 Permanent Junctional Reciprocating Tachycardia

Permanent junctional reciprocating tachycardia (PJRT) is a long RP’ narrow QRS tachycardia due to a rare form of accessory pathway with decremental conduction properties and usually located in the posteroseptal region of tricuspid annulus. Several retrospective multicenter studies were reported that amiodarone, verapamil, and digoxin were effective for controlling PJRT in 40–85% of cases, and Class IC drugs in 60–66%.^849^ Because PJRT frequently leads to tachycardia‐induced cardiomyopathy, pharmacological therapy should be initiated without delay.^849^, ^948^, ^949^ Most of the patients with PJRT will require catheter ablation to control the tachycardia sometime in the future.^950^

##### 11.1.4.5 Focal Atrial Tachycardia/Multifocal Atrial Tachycardia (Figure 35)

**Figure 35:**
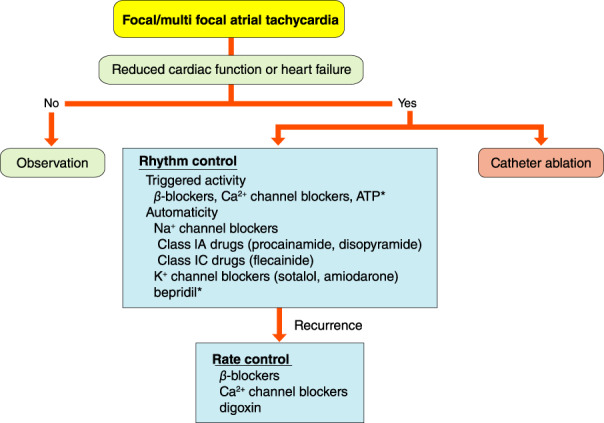
Treatment of atrial tachycardia. *Off‐label. ATP, adenosine triphosphate.

Most of the mechanisms of AT are reported to be increased automaticity or triggered activity, but part of the mechanism of AT is micro‐reentry or macro‐reentry. AT can be repetitive or persistent, and may result in congestive heart failure or tachycardia‐induced cardiomyopathy. In neonates and infants, AT usually improves spontaneously in a few months, and recurrence was not common during a long follow‐up period.^647^, ^663^, ^951^ However, conversion of AT to sinus rhythm by medication is sometimes difficult, and considerable number of cases of AT require rate control.^952^ On the other hand, AT onset in older children is more resistant to antiarrhythmic drugs and less likely to terminate spontaneously.^663^, ^951^

Catheter ablation is the first‐line therapy for drug‐resistance, in older children, and for patients with impaired cardiac function. The purpose of pharmacological therapy is classified into termination of AT (rhythm control) and control of heart rate (rate control).

###### 11.1.4.5.1 Termination of Atrial Tachycardia (Rhythm Control)

In general, AT due to automaticity is terminated by *β*‐blockers,^663^, ^953^ whereas AT due to triggered activity and micro‐reentry is terminated by *β*‐blockers and Ca^2+^ channel blockers, but some ATs are terminated by ATP.^954^, ^955^ In addition, Class IC and IA drugs,^930^, ^956^, ^957^, ^958^ Class III drugs,^959^, ^960^ and Class IV drugs (bepridil) are also effective for controlling refractory AT. Multidrug therapy with 1–3 drugs of digoxin, Class IC or III, combined with *β*‐blockers resulted in conversion to sinus rhythm in 70% of patients.^66^, ^951^, ^952^, ^961^ Although the outcome of multifocal AT is poor, combined use of amiodarone and propafenone was reported as effective to control multifocal AT.^956^ Antiarrhythmic drugs with negative inotropic effect should be used carefully, or avoid, in cases of decreased cardiac function.

###### 11.1.4.5.2 Control of Heart Rate (Rate Control)

If combined therapy fails to convert AT to sinus rhythm, combined therapy with *β*‐blockers, verapamil, and digoxin, which may suppress AV conduction (i.e., rate control), may be a second‐line therapy.^647^, ^951^, ^952^

##### 11.1.4.6 Atrial Flutter

Atrial flutter (AFL) sometimes develops in the fetus and neonate without any organic heart disease, but the incidence is quite rare in school‐aged children. Furthermore, AFL often manifests as a late complication of surgical treatment of CHD. In hemodynamically unstable neonates, R wave‐synchronized electrical cardioversion is the first‐line therapy, and transesophageal overdrive pacing might also be effective to terminate AFL. The sinus rhythm recovery rate of cardioversion and transesophageal overdrive pacing are reported as 87% and 60–70%, respectively.^962^, ^963^ Once AFL is converted to sinus rhythm, the recurrence rate is very low, and long‐term prophylactic antiarrhythmic drug therapy should be unnecessary.^962^ In the hemodynamically stable newborn with AFL, pharmacological therapy might be another option, but it may take some time until AFL terminates and resolves to sinus rhythm.

In school‐aged and older children, the mechanism of AFL without structural heart disease is counterclockwise rotating peritricuspid valve flutter. If the patient has unstable hemodynamics or syncope, synchronized electrical cardioversion should be performed. Catheter ablation is also recommended in these patients. One should note that AFL in older children may be complicated by sick sinus syndrome, and the presence of bradycardia after ablation.

### 11.2 Wide QRS Tachycardia

**Figure 36:**
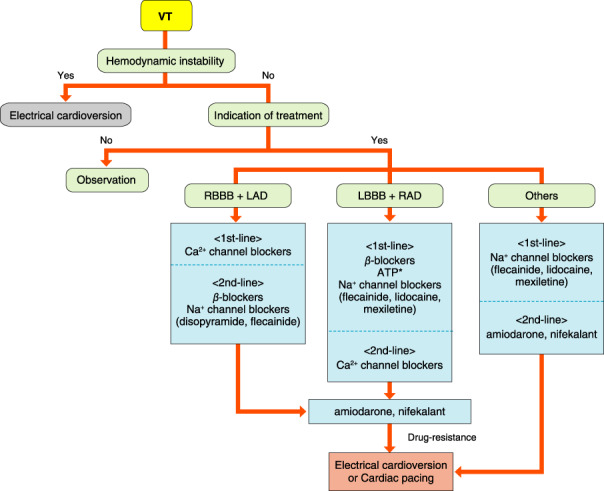
Termination of VT. *Off‐label. ATP, adenosine triphosphate; LAD, left axis deviation; LBBB, left bundle branch block; RAD, right axis deviation; RBBB, right bundle branch block; VT, ventricular tachycardia.

**Figure 37:**
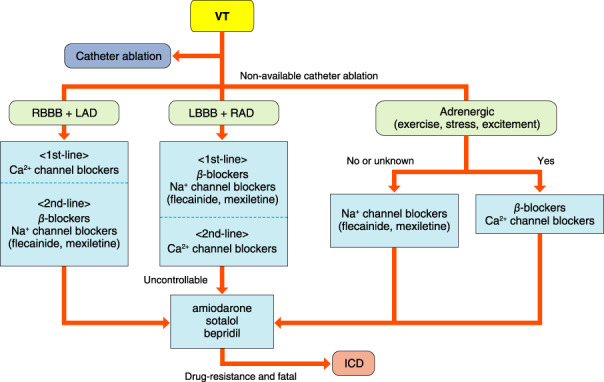
Prophylactic treatment of VT. ICD, implantable cardioverter‐defibrillator; LAD, left axis deviation; LBBB, left bundle branch block; RAD, right axis deviation; RBBB, right bundle branch block; VT, ventricular tachycardia.

The differential diagnosis of wide QRS tachycardia includes monomorphic/polymorphic VT, antidromic AVRT, SVT with bundle branch block, and rapid ventricular conduction via antegrade accessory pathway with AF. In general, fatal VT is uncommon in pediatric patients;^964^, ^965^ however, some patients with ventricular arrhythmia who developed convulsions or syncope have been misdiagnosed as neurological disorders such as epilepsy. Most of the antiarrhythmic drug therapy for wide QRS tachycardia overlaps the treatment for adults (see further discussions in relevant sections) (Figures 36 and 37).

#### 11.2.1 Treatment for Unstable Hemodynamic Patients With Wide QRS Tachycardia

Electrical cardioversion with 1–2 J/kg should be delivered, with an increase in the dose of energy to 4 J/kg if unsuccessful. In cases of recurrence or failure to terminate the tachycardia, combined use with antiarrhythmic drugs should be considered.

#### 11.2.2 Treatment of Stable Hemodynamic Patients With Wide QRS Tachycardia

In general, intravenous administration of lidocaine, nifekalant or amiodarone should be used as first‐line pharmacological therapy. In the case of neonates or patients with low cardiac function after CHD surgery, one must pay close attention to the use of intravenous amiodarone because of the potential for hypotension and circulatory collapse.^902^ Magnesium sulfate and *β*‐blockers may also be administered in the case of TdP.^849^ Even in stable hemodynamic patients, electrical cardioversion should always be kept on standby for emergency use.

#### 11.2.3 Treatment for Each Type of Ventricular Tachycardia

##### 11.2.3.1 Idiopathic Ventricular Tachycardia

**Table 72:** Recommendations and Levels of Evidence for Pharmacotherapy of VT in Pediatric Patients

	COR	LOE	GOR (MINDS)	LOE (MINDS)
Follow‐up without treatment for PVCs or accelerated idioventricular rhythm with normal ventricular function	I	B	B	IVa
Frequent PVC or VT with symptoms or ventricular dysfunction				
Use of *β*‐blockers, Class IC drugs	IIa	C	C1	V
Use of Class IA, III drugs, verapamil*	IIb	C	C1	V
Pharmacotherapy for verapamil‐sensitive VT				
Use of verapamil* >1 year old	I	C	C1	V
Use of *β*‐blockers for infants or children who are unable to take verapamil for any reason	I	C	C1	V
Polymorphic VT or ventricular fibrillation with aborted sudden cardiac death or difficulty with improving the cause of the arrhythmia				
ICD	I	C	C1	V
Use of amiodarone, *β*‐blockers	IIa	C	C1	V

* Contraindicated for neonates and infants.

Abbreviations: ICD, implantable cardioverter‐defibrillator; COR, class of recommendation; GOR, grade of recommendation; LOE, level of evidence; MINDS, Medical Information Network Distribution Service; PVC, premature ventricular contraction; VT, ventricular tachycardia.

Idiopathic VT in children is considered benign when the ventricular arrhythmia disappears during exercise. The incidence of ventricular arrhythmia in children is high in the neonatal period and adolescence. Although non‐sustained VT usually has a relatively good prognosis, it may cause palpitations, syncope, and also heart failure in some patients. For medical therapy, see further discussion in VT section of the adult (**Chapter VII**) (Table 72).^849^, ^902^ If the patient develops polymorphic VT during exercise, one should note the possibility of catecholaminergic polymorphic VT (CPVT; see **Chapter IX.2**).

The first‐line therapy for idiopathic VT is catheter ablation in older children.^4^, ^849^ An implantable cardioverter‐defibrillator (ICD) should be considered for fatal arrhythmias requiring cardioversion, and uncontrollable VT with medication or catheter ablation.

##### 11.2.3.2 Inherited Arrhythmias and Cardiomyopathy

###### 11.2.3.2.1 Long QT Syndrome

Patients with long QT syndrome (LQTS) should be encouraged to make lifestyle and daily living changes. At school, children with LQT1 should avoid competitive exercise, swimming, and marathons. The first‐line pharmacotherapy is *β*‐blockers. Patients who remain symptomatic despite medication should be considered for an ICD. Methylphenidate and atomoxetine, medications for attention deficit hyperactivity disorder, have been reported to increase cardiac events in patients with LQTS.^966^ Neonates with LQT2 complicated by 2 : 1 AV block^967^, ^968^ may require pacemaker implantation with the use of *β*‐blocker or mexiletine.^968^

###### 11.2.3.2.2 Brugada Syndrome

There is a potential risk of sudden cardiac death in older children with Brugada syndrome; therefore, children who develop syncope or cardiac arrest with type I Brugada ST‐T change have a risk of VT or sudden death, and are recommended for an ICD. Implantation of ICD in children has several problems around issues with the leads because of body growth, and inappropriate shock by sinus tachycardia or supraventricular tachycardia. Quinidine or other antiarrhythmic medication might be effective to delay implantation of ICD.^849^

###### 11.2.3.2.3 Catecholaminergic Polymorphic Ventricular Tachycardia

See **Chapter IX.2**.

##### 11.2.3.3 Hypertrophic Cardiomyopathy

Patients with hypertrophic cardiomyopathy with a high risk of sudden cardiac death due to distinct arrhythmia have an indication of ICD with the use of amiodarone. *β*‐blockers and amiodarone could be a bridging therapy in high‐risk children with hypertrophic cardiomyopathy until device implantation.^849^

### 11.3 Postoperative Arrhythmias in Congenital Heart Disease

The mechanism of arrhythmias after CHD surgery is electrical or pathological over time due to residual anatomical defect and surgical scar. After CHD surgery, there can be coincident occurrence of supraventricular and ventricular arrhythmias in the same patient.^969^, ^970^ Spontaneous disappearance of these arrhythmias is unlikely, and usually requires appropriate treatment. One should initiate antiarrhythmic medications after careful evaluation of cardiac function.

#### 11.3.1 Postoperative Atrial Arrhythmias in Congenital Heart Disease

Patients after CHD surgery often develop postoperative scar tissue that may result in intra‐atrial reentrant tachycardia, because almost all surgical procedures require an atriotomy. Catheter ablation is effective treatment for these arrhythmias, and should be an option instead of long‐term pharmacological therapy.^971^ Long‐term drug therapy may not always result in good outcomes.^972^ Although amiodarone might be effective in some of these tachycardias, it has a risk of fast ventricular response, and hemodynamic compromise due to decreased atrial tachycaria rate. *β*‐blockers, Class IC drugs, and Class III drugs might be effective for these reentrant atrial tachycardias, and sotalol was effective for AFL in 78% of postoperative CHD patients.^973^

#### 11.3.2 Postoperative Ventricular Arrhythmias in Congenital Heart Disease

It is well known that VT may develop in long‐term patients with repaired tetralogy of Fallot (TOF). It is reported that 12% of postoperative TOF patients may have VT, and in 8% of the patients it led to sudden cardiac death during 21 years’ follow‐up.^969^, ^974^ *β*‐blockers are recommended in patients with mild symptoms associated with VT, but catheter ablation should be considered for patients with severe symptoms or sustained VT. The outcome of catheter ablation for VT after repaired TOF is reportedly favorable.^975^, ^976^

Medical treatment for VT after CHD surgery might be continued until, or in some cases even after, successful catheter ablation, or ICD implantation. Patients with impaired cardiac function should avoid Class IC drugs, and the use of *β*‐blockers or Class III drugs is recommended.^849^

#### 11.3.3 Arrhythmia in Patients With Single Ventricle and Post‐Fontan Procedure

Most of the patients with single ventricular physiology undergo the Fontan operation as functional hemodynamic repair, and part of the procedure is using the atrial wall as a Fontan circuit. The atrial muscle after this procedure is dilated, scarred, and arrhythmogenic; thus, occurrence of resistant atrial arrhythmia is not uncommon.^977^ These atrial arrhythmias might be improved by surgical replacement with an artificial conduit (total cavopulmonary connection),^978^ catheter ablation, or combined pharmacotherapy such as *β*‐blockers and Class III drugs.^979^, ^980^, ^981^

## 12 Arrhythmias During Pregnancy

During pregnancy, the maternal physiology changes dynamically: volume of circulating blood, heart rate and sympathetic nerve activity increase, and blood electrolytes and many hormone levels associated with pregnancy fluctuate. Myocardial extension with increased preload can cause potential myocardial instability. Therefore, the incidence of arrhythmias, including benign ones that do not require therapeutic intervention, increases. Arrhythmia is the most common cardiovascular event observed in pregnant women, especially during the second trimester (14th to 27th week) to the third trimester (28th week) of pregnancy.^982^ Sinus arrhythmia, including sinus tachycardia, and supraventricular/ventricular extrasystoles are frequently observed, even in normal pregnancy.^983^ In addition, many pregnant and postpartum women complain of palpitations and dizziness, but these are not always caused by arrhythmia.^984^

Pregnant women with a history of arrhythmia before pregnancy are more likely to relapse during the perinatal period. Especially, a pregnancy complicated with AF and/or atrial flutter needs careful attention because the risks of neonatal complications, such as preterm birth and low birth weight, have been reported as high.^985^, ^986^ Catheter ablation or device implantation before pregnancy is preferred in women with this indication.

Most treatments for arrhythmia during pregnancy are those given in the non‐pregnant condition, but it is necessary to select drugs while considering the effect on the fetus (Table 73). As a point to note, because it is difficult to carry out a safety test of drug treatment in pregnant or lactating women, the description in the package insert and the experience in actual clinical use are sometimes dissociated. In this guideline, following the previous guidelines, a comprehensive evaluation of the use of antiarrhythmic agents during pregnancy was performed.^987^, ^988^ Please note that some of the recommendations are different from those in the package insert. A *β*‐blocker can be administered (only when the benefit exceeds the risk), with consideration for side effects on the baby, such as intrauterine growth retardation.^987^

**Table 73:** Safety of Antiarrhythmic Drugs for Pregnant and Lactating Women

Vaughan Williams’ classification	Antiarrhythmic drugs	Administration to pregnant women (Information on package insert)	Comprehensive evaluation of pregnancy risk	Administration to lactating women (Information on package insert)	Comprehensive evaluation of lactation risk
IA	procainamide	Probably compatible	Probably compatible	Avoid lactation	Probably compatible
	cibenzoline	Probably compatible	Probably compatible	Avoid lactation	No data
	disopyramide	Possibly harmful	Probably compatible	Avoid lactation	Probably compatible
	quinidine	Probably compatible	Compatible	Avoid lactation	Probably compatible
IB	lidocaine	Probably compatible	Compatible	Not mentioned	Probably compatible
	mexilletine	Probably compatible	Probably compatible	Avoid lactation	Probably compatible
	aprindine	Contraindicated	Possibly harmful	Avoid lactation	No data
IC	pilsicainide	Probably compatible	Probably compatible	Avoid lactation	No data
	propafenone	Probably compatible	Probably compatible	Avoid lactation	Probably compatible
	flecainide	Contraindicated	Probably compatible	Avoid lactation	Probably compatible
II	propranolol	Probably compatible (in emergency use)	Probably compatible	Avoid lactation	Probably compatible
	bisoprolol	Contraindicated	Probably compatible	Avoid lactation	Insufficient data (refer similar drugs)
	metoprolol	Contraindicated	Probably compatible	Avoid lactation	Probably compatible
	nadolol	Contraindicated	Probably compatible	Avoid lactation	Insufficient data (refer similar drugs)
	carvedilol	Contraindicated	Probably compatible	Avoid lactation	Probably compatible
	landiolol	Probably compatible	Probably compatible	Not mentioned	No data
III	amiodarone	Possibly harmful	Possibly harmful	Avoid lactation	Contraindicated
	sotalol	Possibly harmful	Probably compatible	Avoid lactation	Insufficient data (Considering *β*‐blocking action)
IV	verapamil	Contraindicated	Probably compatible	Avoid lactation	Probably compatible
	diltiazem	Contraindicated	Probably compatible	Avoid lactation	Probably compatible
Others	digoxin	Probably compatible	Compatible	Not mentioned	Probably compatible

Amiodarone should be avoided as much as possible during pregnancy because of its effects on the fetal thyroid gland, but not in cases of low cardiac function and high risk for sudden death. Because amiodarone has a high rate of transfer to breast milk, breastfeeding is not recommended.

### 12.1 Superior Ventricular Extrasystole / Ventricular Extrasystole

Superior ventricular/ventricular extrasystole occurs very often during pregnancy, in women with and without organic heart disease.^983^, ^984^ Many are asymptomatic and do not require treatment; but if symptoms are severe, or if they are associated with paroxysmal AF or hypofunction of the heart, drug treatment should be considered.

### 12.2 Supraventricular Tachycardia

During pregnancy, pharmacotherapy is the main focus, and the drugs used are the same as when non‐pregnant. For reentrant supraventricular tachycardia that persists even after attempting the Valsalva procedure, adenosine has no placental crossing and does not affect the fetus, so can be safely used.^988^, ^989^ In severe cases, group I drugs for Wolf‐Parkinson‐White (WPW) syndrome. *β*‐blockers and verapamil, for cases other than WPW syndrome, are effective for prevention in severe cases.^988^ Because ectopic atrial tachycardia causes tachycardia‐induced cardiomyopathy, if it is difficult to return to sinus rhythm, use a *β*‐blocker, verapamil or digoxin to control the heart rate. If heart rate control is inadequate, consider ablation treatment in a specialized facility.^988^

### 12.3 Atrial Fibrillation / Atrial Flutter

AF in pregnant women with structural heart disease is more likely to occur between the 20th and 30th weeks of gestation, and maternal mortality and rates of fetal complications are high.^986^ Angiotensin‐converting enzyme inhibitors (ACE inhibitors) and angiotensin II receptor blockers (ARBs) as upstream treatment are contraindicated after the second trimester of pregnancy. AF in pregnant women without structural heart disease is rare, and differential diagnosis for hyperthyroidism or electrolyte abnormalities is necessary.

Heart rate control is the main focus for persistent AF. Electrical cardioversion is selected when hemodynamics are unstable or when heart failure is exacerbated by the continuation of tachycardia. Pharmacotherapy for the purpose of preventing recurrence is similar to that administered during non‐pregnancy, but attention should be paid to arrhythmogenic and negative inotropic effects.^987^ Amiodarone should be replaced with other antiarrhythmic drugs as much as possible, and minimum use should be observed.

Anticoagulant therapy is performed according to the risk of thromboembolism during non‐pregnancy.^21^ In a pregnant woman needing anticoagulation for AF, heparin use is recommended because warfarin has a great risk for the fetus. The safety of direct oral anticoagulants during pregnancy has not been well established.^988^

### 12.4 Ventricular Tachycardia

Idiopathic ventricular tachycardia (outflow tract origin or verapamil‐sensitive VT) may be present during pregnancy, but many cases are associated with organic heart disease. Clinically significant VT occurs in 1–2% of pregnancies with organic heart disease, such as cardiomyopathy, with the most frequent occurrence in the third trimester of pregnancy. In cases of VT, maternal death, neonatal death, premature birth and low birth weight are more common.^990^ If hemodynamics are unstable, emergency cardioversion should be performed. If stable, oral treatment with *β*‐blockers, verapamil, sotalol or other antiarrhythmic drugs is recommended.^850^ Severe cases may require an ICD. However, its application should be carefully considered for ventricular arrhythmias associated with peripartum cardiomyopathy, in which cases cardiac function often recovers.^991^, ^992^

### 12.5 Inherited Arrhythmias

In congenital long QT syndrome, especially type 2, postpartum arrhythmic events are more common than during pregnancy, and *β*‐blockers are effective in avoiding such events.^993^, ^994^ Catecholaminergic polymorphic ventricular tachycardia may be exacerbated during pregnancy with increased sympathetic activity, so use of *β*‐blockers and flecainide is recommended. In Brugada syndrome, pregnancy is not reported to increase arrhythmic events.^995^, ^996^

### 12.6 Bradycardia

Because heart rate tends to increase during pregnancy, pregnancy and childbirth in women with bradycardia, but without pacemaker indication before pregnancy, often end without complications. However, because sympathetic nerve activity can decrease postpartum, careful attention is needed for prevention of bradycardia exacerbation.^997^

## Appendix 1

### 1.1

**Table:** Table 74 Indication, Dosage and Administration of Antiarrhythmic Drugs for Adults

Vaughan Williams classification	Antiarrhythmic drug	Indication with respect to National Health Insurance	Composition, formulation and dosage	Usage and administration	FDA/ADEC criteria	Mothers’ milk criteria
IA	quinidine	quinidine is indicated widely for the prevention of extrasystoles, paroxysmal tachycardia and paroxysmal atrial fibrillation (AFIB), i.e., used for the maintenance of sinus rhythm after the termination of new‐onset AFIB, recurrent AFIB and atrial flutter (AFL) sometimes in combination with electrical cardioversion/defibrillation. quinidine is also used for the prevention of ventricular arrhythmias observed with acute myocardial infarction (AMI)	Tablet: 100 mg	Oral administration: begin at 600 mg/day three times daily and increase dosage thereafter. Maintenance dose is 200–600 mg 1–3 times daily. If this regimen is not effective, quinidine should be terminated 6 days after initiation, and so high‐dose quinidine ceased 3 days after initiation	ID / C	L3
IA	disopyramide	disopyramide capsule is indicated for extrasystoles, paroxysmal supraventricular tachyarrhythmias and AFIB Extended‐release disopyramide tablet is indicated for tachyarrhythmias Oral disopyramide is used for tachyarrhythmias refractory to other antiarrhythmic drugs or patient is intolerant of alternative agents Intravenous disopyramide is used for urgent treatment of extrasystoles, paroxysmal tachycardia and AFIB/AFL	Capsule: 50 mg, 100 mg Extended‐release tablet: 150 mg Ampule for dilution: 50 mg (5 mL/ampule)	Oral administration: disopyramide capsules are taken at 300 mg/day three times daily, and extended‐release tablet is taken at 300 mg/day twice daily Intravenous administration: disopyramide is infused at dose of 50–100 mg slowly over 5 min	C / B2	L2
IA	cibenzoline	cibenzoline ampule is indicated for tachyarrhythmias. cibenzoline tablet is used for the treatment of tachyarrhythmias refractory to other antiarrhythmic drugs or patient is intolerant of alternative agents	Tablet: 50 mg, 100 mg Ampule for dilution: 70 mg (5 mL/ampule)	Oral administration: cibenzoline tablet is started at 300 mg/day and can be increased incrementally to 450 mg/day three times daily. Intravenous administration: cibenzoline (0.1 mL/kg, 1.4 mg/kg) is infused slowly (2–5 min) after dilution with saline or glucose solution under monitoring of blood pressure (BP) and ECG	ID / ID	ID
IA	pirmenol	pirmenol capsule is administered for the treatment of (ventricular) tachyarrhythmias refractory to other antiarrhythmic drugs or patient is intolerant of alternative agents	Capsule: 50 mg, 100 mg	Oral administration: pirmenol is taken at 200 mg/day twice daily	ID / ID	ID
IA	procainamide	procainamide tablet is used for the prevention and the treatment of extrasystoles, paroxysmal tachycardias including new‐onset or recurrent AFIB. Also used for the prevention of (ventricular) arrhythmias observed in patients with AMI, the maintenance of sinus rhythm after electrical cardioversion/defibrillation or the prevention of arrhythmias observed in perioperative patients under anesthesia procainamide ampule is used for the treatment of extrasystoles, paroxysmal tachycardias and perioperative arrhythmias including new‐onset AFIB. Intravenous injection is available for AFL	Tablet: 125 mg, 250 mg Ready‐to‐use ampule: 100 mg (10% 1 mL/ampule), 200 mg (10% 2 mL/ampule)	Oral administration: procainamide is taken at 250–500 mg every 3–6 h daily Systemic administration: intravenous procainamide (200–1,000 mg as a single use) is infused at 50–100 mg/min, and intramuscular procainamide is used at a dose of 500 mg every 4–6 h	C / B2	L3
IB	aprindine	aprindine ampule is indicated for tachyarrhythmias. aprindine capsule is used for the treatment of tachyarrhythmias refractory to other antiarrhythmic drugs or patient is intolerant of alternative agents	Capsule: 10 mg, 20 mg Ampule for dilution: 100 mg (10 mL/ampule)	Oral administration: aprindine is initiated at 40 mg/day and can be increased incrementally to 60 mg/day 2–3 times daily Intravenous administration: aprindine (1.5–2.0 mg/kg) is infused at 5–10 mL/min after 10‐fold dilution with 5% glucose solution. Single cumulative dosage should be limited to 100 mg	ID / ID	ID
IB	mexilletine	mexilletine is administered for the treatment of (ventricular) tachyarrhythmias	Capsule: 50 mg, 100 mg Ampule for dilution: 125 mg (5 mL/ampule)	Oral administration: mexilletine is taken at 300–450 mg/day three times daily Intravenous administration: ampule of mexilletine (125 mg) is infused at a dose of 2–3 mg/kg for 5–10 min. Ampule can be administered by drip infusion at 0.4–0.6 mg/kg/h after dilution with saline or glucose solution	C / B1	L2
IB	lidocaine	lidocaine is administered for the treatment of ventricular or supraventricular extrasystoles, paroxysmal tachycardias and for the prevention of perioperative ventricular arrhythmias (VA) and VA associated with AMI	Ready‐to‐use syringe: 100 mg, 2% (5 mL) Ready‐to‐use bag: 2,000 mg, 1% (200 mL)	Intravenous injection: ready‐to‐use syringe of lidocaine is injected at a dosage of 50–100 mg (1–2 mg/kg) for 1–2 min Intravenous drip infusion: ready‐to‐use bag of lidocaine is infused at a dosage of 1–2 mg/min (up to 4 mg/min)	B / A	L2
IC	pilsicainide	pilsicainide capsule is used for the treatment of tachyarrhythmias refractory to other antiarrhythmic drugs or patient is intolerant of alternative agents pilsicainide ampule is indicated for the urgent treatment of tachyarrhythmias	Capsule: 25 mg, 50 mg Ampule for dilution: 50 mg (5 mL/ampule)	Oral administration: pilsicainide is taken at 150 mg/day three times a day (maximum dose is 225 mg/day) Intravenous administration: pilsicainide is infused slowly (10 min) at the maximum dose of 0.75 mg/kg for extrasystoles and 1 mg/kg for tachycardia after dilution with saline or glucose solution under monitoring of BP and ECG	ID / ID	ID
IC	flecainide	flecainide tablet is used for the treatment of tachyarrhythmias such as paroxysmal AFIB/AFL and ventricular tachyarrhythmias flecainide ampule is indicated for the urgent treatment of tachyarrhythmias.	Tablet: 50 mg, 100 mg Ampule for dilution: 50 mg (5 mL/ampule)	Oral administration: flecainide is started at 100 mg/day and can be increased incrementally to 200 mg/day twice daily Intravenous administration: flecainide is infused slowly (10 min) at a dosage of 1–2 mg/kg after dilution with glucose solution under monitoring of BP and ECG. Single cumulative dosage is limited to 150 mg	C / B3	L3
IC	propafenone	propafenone tablet is indicated for the treatment of tachyarrhythmias refractory to other antiarrhythmic drugs or patient is intolerant of alternative agents	Tablet: 100 mg, 150 mg	Oral administration: propafenone is taken at 450 mg/day three times daily The dosage is individualized, and 100 mg tablet is used for initial administration to the elderly	C / ID	ID
II	atenolol	atenolol tablet is indicated for tachyarrhythmias of sinus tachycardia and extrasystoles	Tablet: 25 mg, 50 mg	Oral administration: atenolol is usually taken at 50–100 mg once daily	ID / C	L3
II	arotinolol	arotinolol tablet is indicated for the treatment of tachyarrhythmias	Tablet: 5 mg, 10 mg	Oral administration: arotinolol is usually administered at 20 mg/day twice daily, and the maximum dose is 30 mg/day, when, necessary.	ID / ID	ID
II	esmolol	esmolol is indicated for the urgent treatment of intraoperative supraventricular tachyarrhythmias	Vial: 100 mg (10 mL/vial)	Intravenous administration: esmolol is infused slowly (30 s) to adults at a dosage of 1 mg/kg, which may be followed by intravenous drip infsion at 150 *μ*g/kg/min. Infusion speed may be customized	ID / C	ID
II	carvedilol	carvedilol tablet is indicated for the treatment of rapid AFIB	Tablet: 1.25 mg, 2.5 mg, 10 mg, 20 mg	Oral administration: carvedilol is initiated at 5 mg once daily and may be increased incrementally to 10 mg and then 20 mg once a day (maximum dose is 20 mg/day)	ID / C	L3
II	carteolol	carteolol is indicated for the treatment of arrhythmias	Granule medicine: 1%, 0.2% glanular form is applied when converted to mg/day unit. Tablet: 5 mg Extended‐release capsule: 15 mg	Oral administration: carteolol tablet is started at 10–15 mg/day 2–3 times daily, and the maximum dose is 30 mg/day. carteolol capsule is taken at 15–30 mg once daily	C / ID	ID
II	nadolol	nadolol tablet is indicated for the treatment of tachyarrhythmias	Tablet: 30 mg, 60 mg	Oral administration: nadolol is usually taken at 30–60 mg once daily	ID / ID	ID
II	bisoprolol	bisoprolol tablets (2.5 mg, 5 mg) are indicated for the treatment of ventricular extrasystoles and rapid AFIB. bisoprolol transdermal patch is used for rapid AFIB	Tablet: 2.5 mg, 5 mg Transdermal patch: 2 mg, 4 mg, 8 mg	Oral administration: bisoprolol is taken once a day at 5 mg/day for ventricular extrasystoles and 2.5–5 mg/day for rapid AFIB Transdermal treatment: patch is used at 4–8 mg/day for rate control of rapid AFIB	C / C	ID
II	pindolol	pindolol tablet is used for sinus tachycardia alone	Tablet: 5 mg	Oral administration: pindolol is taken at 1–5 mg as a single dose and three times a day. This means that doses are from 3 mg/day to 15 mg/day	B / C	L3
II	propranolol	propranolol tablet is indicated for the prevention of extrasystoles and paroxysmal tachycardias including AFIB. Also used for rate control of rapid AFIB and the treatment of sinus tachycardia and new‐onset AFIB Use of extended‐release capsule is limited to the treatment of angina pectoris and essential hypertension in the National Health Insurance propranolol ampule is used for the treatment of extrasystoles, paroxysmal tachycardia, rapid AFIB, arrhythmias observed under anesthesia, sinus tachycardia and new‐onset AFIB	Tablet: 10 mg Extended‐release capsule: 60 mg Ampule for dilution: 2 mg (2 mL/ampule)	Oral administration: propranolol is started at 30 mg/day three times daily and can be increased incrementally to 90 mg/day three times daily.Intravenous administration: follow the instructions in the package insert	ID / C	L2
II	metoprolol	metoprolol tablet is used for tachyarrhythmias	Tablet: 20 mg, 40 mg Extended‐release tablet: 120 mg	Oral administration: metoprolol is taken at 60–120 mg/day 2–3 times daily	ID / C	L2
II	landiolol	landiolol is indicated for the urgent treatment of (1) intraoperative or (2) postoperative tachyarrhythmias including sinus tachycardia and rapid AFIB/AFL under hemodynamic monitoring landiolol is also indicated for the urgent treatment of (3) tachyarrhythmias (AFIB/AFL) and (4) life‐threatening ventricular fibrillation (VF) and hemodynamically unstable ventricular tachycardia (VT) observed in patients with compromised cardiac function	Ready‐to‐use vial: 50 mg, 150 mg	(1)Intravenous landiolol infusion is started at 0.125 mg/kg/min and followed by continuous drip infusion at 0.01–0.04 mg/kg/min (2)Intravenous landiolol is started at 0.06 mg/kg/min and followed by drip infusion at 0.02 mg/kg/min for 5–10 min as a first regimen. The second regimen is started at 0.125 mg/kg/min and followed by continuos infusion at 0.04 mg/kg/min, when appropriate heart rate reduction is not obtained by the first regimen.(3), (4)Intravenous landiolol infusion is started at 1 *μ*g/kg/min, followed by continuous infusion at 1–10 *μ*g/kg/min as appropriate. The maximum infusion rate (dosage of landiolol) is limited to 40 *μ*g/kg/min	ID / ID	ID
III	amiodarone	amiodarone tablet is used for the life‐threatening recurrent VT, VF and AFIB in patients with hypertrophic cardiomyopathy or heart failure (HF) and those refractory to other antiarrhythmics or intolerant of alternative agents. Intravenous amiodarone is used for the urgent treatment of life‐threatening VF and hemodynamically unstable VT. Also used for the treatment and prophylaxis of VF refractory to electrical defibrillation and pulseless VT followed by ventricular standstill	Tablet: 100 mg OD tablet: 50 mg, 100 mg Ampule for dilution: 150 mg (3 mL/ampule)	Oral administration: amiodarone is initiated at 400 mg/day and continued at 200 mg/day once or twice daily Intravenous administration: follow the instructions in the package inserts for amiodarone ampule (150 mg)	ID / C	L5
III	sotalol	sotalol is used for the treatment of life‐threatening recurrent VT/VF refractory to other antiarrhythmic durgs or patient is intolerant of alternative agents	Tablet: 40 mg, 80 mg	Oral administration: sotalol is started at 80 mg/day twice daily and titrated up to 320 mg/day twice daily	B / C	L4
III	nifekalant	nifekalant is used for the treatment of life‐threatening VT/VF refractory to other antiarrhythmic drugs or patient is intolerant of alternative agents	Vial for dilution: 50 mg	Intravenous administration: after dilution by saline or glucose solution, intravenous nifekalant is administered slowly (5 min) at 0.3 mg/kg as a single use. nifekalant vial is also available for intravenous drip infusion at a constant speed of 0.4 mg/kg/h. ECG monitoring is required in both regimens	ID / ID	ID
IV	diltiazem	Intravenous diltiazem is used for the treatment of supraventricular tachyarrhythmias	Tablet: 30 mg, 60 mg Extended‐release capsule: 100 mg, 200 mg Vial for dilution: 10 mg, 50 mg, 250 mg	Intravenous administration: diltiazem (10 mg) is administered slowly (3 min). 10 mg and 50 mg vials are available for the treatment of arrhythmias; 250 mg vial is available only for hypertensive emergency and unstable angina (Oral application of diltiazem for patient with arrhythmias is not formally approved)	ID / C	L3
IV	bepridil	bepridil is used for the treatment of persistent AFIB and (ventricular) tachyarrhythmias refractory to other antiarrhythmic drugs or patient is intolerant of alternative agents	Tablet: 50 mg, 100 mg	bepridil is started at 100 mg/day twice daily and titrated to the maximum dose of 200 mg/day twice daily for persistent AFIB bepridil is administered at 200 mg/day twice daily for ventricular tachyarrhythmias	ID / ID	ID
IV	verapamil	verapamil tablet is used for the treatment of AFIB/AFL and the prevention of paroxysmal supraventricular tachycardia (PSVT) verapamil ampule is used for the termination of ongoing PSVT and the treatment of AFIB/AFL	Tablet: 40 mg Ampule for dilution: 5 mg (2 mL/ampule)	Oral administration: verapamil is taken at 40–80 mg as a single dose, and three times daily.Intravenous administration: verapamil (5 mg) is administered slowly after dilution with saline or glucose solution, if necessary	ID / C	L2
Others	ATP	ATP has no indication for the treatment of arrhythmias in the National Health Insurance. However, ATP is effective for the termination of the ongoing PSVT	Ready‐to‐use ampule: 10 mg, 20 mg, 40 mg	Intravenous administration: ATP (10 mg) should be injected within 1–2 s under monitoring of BP and ECG, although this procedure is not valid in the National Health Insurance	ID / ID	ID
Others	atropine	atropine is indicated for vagotonic bradyarrhythmias, including atrioventricular conduction disturbance	Powder as atropine sulfate (refined as purity of 98% or more) Ready‐to‐use ampule: 0.5 mg (1 mL)	Oral administration: atropine is administered at 1.5 mg/day three times daily Intravenous, intramuscular or subcutaneous administration: injection of atropine (0.5 mg) is performed as a single shot	ID / A	L3
Others	digoxin	digoxin is indicated for the prevention and the treatment of various types of tachyarrhythmias, including rapid AFIB/AFL and PSVT digoxin is also indicated for patients with perioperative HF or those with HF associated with febrile status, labor shock or acute intoxication	Tablet: 0.125 mg, 0.25 mg Ready‐to‐use ampule: 0.25 mg (1 mL/ampule)	Oral administration: digoxin is taken at an initial dose of 0.5–1.0 mg and a cumulative dose of 1.0–4.0 mg in a rapid titration regimen. Thereafter, oral digoxin is continued at a dose of 0.5 mg every 6–8 h as a maintenance regimen Intravenous administration: follow the instructions in the package insert	C / ID	L2
Others	deslanoside	deslanoside is indicated for the prevention and treatment of various types of tachyarrhythmias, including rapid AFIB/AFL and PSVT deslanoside is also indicated for patients with perioperative HF or those with HF associated with febrile status, labor shock or acute intoxication	Ready‐to‐use ampule: 0.4 mg (2 mL/ampule)	Intravenous administration: follow the instructions in the package insert for both rapid titration and maintenance regimens	ID / ID	ID
Others	magnesium	magnesium has no indication for the treatment of arrhythmias in the National Health Insurance However, magnesium is reported to be effective for polymorphic VT (so‐called torsade de pointes)	Ready‐to‐use plastic ampule: 2.0 g (20 mL/vial)	Intravenous administration: magnesium is infused slowly at 1–2 g as a single use under monitoring of BP and ECG	ID / ID	ID
Others	methyldigoxin	methyldigoxin is indicated for the treatment of rapid AFIB/AFL and PSVT	Tablet: 0.1 mg, 0.25 mg	Oral administration: follow the instructions in the package insert for both the rapid titration and maintenance regimens	ID / ID	ID

Risk categories for pregnant women and fetus rely on the criteria of U.S. FDA and ADEC. These are also based on the Physicians’ Desk Reference® (PDR), the most recognized drug information reference available in the U.S. (http://www.pdr.net). ID, insufficient data.

**Table:** Table 75 FDA Pregnancy Risk Categories Prior to 2015

Category	U.S. FDA Criterion
A	Adequate and well‐controlled studies have failed to demonstrate a risk to the fetus in the first trimester of pregnancy (and there is no evidence of risk in later trimesters)
B	Animal reproduction studies have failed to demonstrate a risk to the fetus and there are no adequate and well‐controlled studies in pregnant women
C	Animal reproduction studies have shown an adverse effect on the fetus and there are no adequate and well‐controlled studies in humans, but potential benefits may warrant use of the drug in pregnant women despite potential risks
D	There is positive evidence of human fetal risk based on adverse reaction data from investigational or marketing experience or studies in humans, but potential benefits may warrant use of the drug in pregnant women despite potential risks
X	Studies in animals or humans have demonstrated fetal abnormalities and/or there is positive evidence of human fetal risk based on adverse reaction data from investigational or marketing experience, and the risks involved in use of the drug in pregnant women clearly outweigh potential benefits

URL: https://www.drugs.com/pregnancy‐categories.html. In 2015, the FDA replaced the former pregnancy risk categories on prescription, which still may be found in some package inserts. The new labeling system requires patient‐specific counseling and informed decision making.

**Table:** Table 76 Definitions of the Australian Categories for Prescribing Medicines in Pregnancy

Category	Australian Drug Evaluation Committee (ADEC) Criterion
A	Drugs that have been taken by a large number of pregnant women and women of childbearing age without any proven increase in the frequency of malformations or other direct or indirect harmful effects on the fetus having been observed
B1	Drugs that have been taken by only a limited number of pregnant women and women of childbearing age, without an increase in the frequency of malformation or other direct or indirect harmful effects on the human fetus having been observed. Studies in animals have not shown evidence of an increased occurrence of fetal damage
B2	Drugs that have been taken by only a limited number of pregnant women and women of childbearing age, without an increase in the frequency of malformation or other direct or indirect harmful effects on the human fetus having been observed. Studies in animals are inadequate or may be lacking, but available data show no evidence of an increased occurrence of fetal damage
B3	Drugs that have been taken by only a limited number of pregnant women and women of childbearing age, without an increase in the frequency of malformation or other direct or indirect harmful effects on the human fetus having been observed. Studies in animals have shown evidence of an increased occurrence of fetal damage, the significance of which is considered uncertain in humans
C	Drugs that, owing to their pharmacological effects, have caused or may be suspected of causing, harmful effects on the human fetus or neonate without causing malformations. These effects may be reversible. Accompanying texts should be consulted for further details
D	Drugs that have caused, are suspected to have caused or may be expected to cause an increased incidence of human fetal malformations or irreversible damage. These drugs may also have adverse pharmacological effects. Accompanying texts should be consulted for further details
X	Drugs that have such a high risk of causing permanent damage to the fetus they should not be used in pregnancy or when there is a possibility of pregnancy

URL: https://www.tga.gov.au/australian‐categorisation‐system‐prescribing‐medicines‐pregnancy.

**Table:** Table 77 Lactation Risk Categories

Category	Medications and Mothers’ Milk 2019
L1	**Compatible (safest)**Drug which has been taken by a large number of breastfeeding mothers without any observed increase in adverse effects in the infant. Controlled studies in breastfeeding women fail to demonstrate a risk to the infant and the possibility of harm to the breastfeeding infant is remote; or the product is not orally bioavailable in an infant
L2	**Probably compatible (safer)**Drug which has been studied in a limited number of breastfeeding women without an increase in adverse effects in the infant; And/or, the evidence of a demonstrated risk which is likely to follow use of this medication in a breastfeeding women is remote
L3	**Probably compatible (moderately safe)**Here are no controlled studies in breastfeeding women, however the risk of untoward effects to a breasfed infant is possible, or controlled studies show only minimal non‐threatening adverse effects. Drugs should be given only if the potential benefit justifies the potential risk to the infant (New medications that have absolutely no published data are automatically categorized in this category, regardless of how safe they may be)
L4	**Potentially hazardous (possibly hazardous)**There is positive evidence of risk to a breastfed infant or to breastmilk production, but the benefits of use in breastfeeding mothers may be acceptable despite the risk to the infant (e.g., if the drug is needed in a life‐threatening situation or for a serious disease for which safer drugs cannot be used or are ineffective)
L5	**Hazardous (contraindicated)**Studies in breastfeeding mothers have demonstrated that there is significant and documented risk to the infant based on human experience, or it is a medication that has a high risk of causing significant damage to an infant. The risk of using the drug in breastfeeding women clearly outweighs any possible benefit from breastfeeding. THe drug is contraindicated in women who are breastfeeding an infant

From: Hale TW. Hale’s Medication & Mothers’ Milk^TM^ 2019, 18th edn. Springer 2019.^998^ Republished with permission of Springer Publishing Company, permission conveyed through Copyright Clearance Center, Inc.

**Table:** Table 78 Indication, Dosage and Administration of Antiarrhythmic Drugs for Children

Vaughan Williams Classification	Antiarrhythmic drug	Indication with respect to National Health Insurance	Administration	Dosage
IA	disopyramide	disopyramide is indicated for supraventricular tachycardia (SVT) and VT	Intravenous	Intravenous administration: disopyramide is diluted and administered slowly over 5 min at a dosage of 1–2 mg/kg
			Oral	Oral administration: disopyramide is taken at 5–15 mg/kg/day three times daily (maximum dose of 300 mg/day)
IA	procainamide	procainamide is indicated for tachyarrhythmias	Intravenous	Intravenous administration: procainamide is diluted and administered slowly over 10 min at a dosage of 2–10 mg/kg
			Oral	Oral administration: procainamide is taken at 20–60 mg/kg/day 3–4 times daily until termination of the tachycardia
IB	mexilletine	mexilletine is used for the treatment of ventricular tachyarrhythmias	Intravenous	Intravenous administration: mexilletine is diluted and administered slowly (5–10 min) at a dosage of 2–3 mg/kg. Intravenous injection may be followed by continuous drip infusion at 0.4–0.6 mg/kg/h, if the first injection is effective
			Oral	Oral administration: mexilletine is taken at 5–15 mg/kg/day 3–4 times daiy (maximum dose of 450 mg/day)
IB	lidocaine	lidocaine is administered for VT	Intravenous	Intravenous administration: lidocaine is diluted and infused at a dosage of 1 mg/kg, and continuous drip infusion at 0.025–0.05 mg/kg/min may follow i.v. lidocaine, if it is effective
IC	flecainide	flecainide is indicated for tachyarrhythmias	Intravenous	Intravenous administration: flecainide amplule is diluted and infused slowly (10 min) at dosage of 1–2 mg/kg with a maximum dose of 150 mg
			Oral	Oral administration: flecainide is taken at 1–4 mg/kg/day twice daily (maximum dose of 200 mg)
IC	propafenone	propafenone is indicated for tachyarrhythmias	Oral	Oral administration: propafenone is taken at 5–10 mg/kg three times daily (maximum dose of 450 mg)
II	atenolol	atenolol is indicated for tachyarrhythmias (sinus tachycardia and extrasystoles)	Oral	Oral administration: atenolol is taken at 1–2 mg/kg once daily
II	bisoprolol	bisoprolol tablet is indicated for ventricular extrasystoles	Oral	Oral administration: bisoprolol is taken at 0.08–0.1 mg/kg once daily
II	propranolol	propranolol is indicated for the treatment of tachyarrhythmias and the management of long‐QT syndrome	Intravenous	Intravenous administration: propranolol is injected slowly over 10 min at dose range of 0.05–0.1 mg/kg
			Oral	Oral administration: propranolol is taken at 1–3 mg/kg/day 3–4 times daily
III	amiodarone	amiodarone is indicated for life‐threatening VT/VF	Intravenous	Intravenous administration: amiodarone ampule (150 mg) is diluted by 5% glucose solution and intravenous injection is started at 2.5 mg/kg for 10 min. Consolidating administration is 1 mg/kg/h for the next 6 h. Maintenance administration is 0.5 mg/kg/h for the following 42 h. Additive administration is 2.5 mg/kg for 10 min with recurrence of hemodynamically unstable VT or VF
			Oral	Oral administration: amiodarone is initiated at 5–10 mg/kg once or twice daily for the first 1–2 weeks and continued at 2.5–5 mg/kg once or twice daily as a maintenance dose
III	sotalol	sotalol is used for the treatment of VT	Oral	Oral administration: sotalol is started at 1–2 mg/kg and titrated up to 8 mg/kg twice daily
III	nifekalant	nifekalant is used for the treatment of VT/VF	Intravenous	Intravenous administration: nifekalant is infused at 0.3 mg/kg slowly (10 min). Single use may be followed by intravenous drip infusion at a constant speed of 0.2–0.4 mg/kg/h
IV	bepridil	bepridil is used for the treatment of ventricular tachyarrhythmias	Oral	Oral administration: bepridil is started at 2–4 mg/kg and titrated to a maximum dose of 200 mg
IV	verapamil	verapamil is indicated for tachyarrhythmias	Intravenous	Intravenous administration: verapamil ampule (5 mg) is diluted and infused slowly over 5 min at 0.1 mg/kg
			Oral	Oral administration: verapamil is taken at 3–6 mg/kg/day three times daily
Others	ATP	ATP has no indication for the treatment of arrhythmias in the National Health Insurance. However, ATP is effective for terminating ongoing PSVT	Intravenous	Intravenous administration: ATP (10 mg) is injected rapidly without dilution
Others	atropine	atropine is indicated for vagotonic bradyarrhythmias, including atrioventricular conduction disturbance	Intravenous	Intravenous administration: atropine is infused at 0.01–0.02 mg/kg as a single shot
Others	digoxin	digoxin is indicated for the prevention and the treatment of PSVT	Intravenous	Intravenous administration: digoxin is infused 3–4 times daily at a dose of 0.03–0.05 mg/kg for infants and 0.02–0.04 mg/kg for school children
			Oral	Oral administration: digoxin is maintained at a dose of 0.01–0.025 mg/kg/day for infants and 0.008–0.02 mg/kg/day for school children
Others	magnesium	Intravenous magnesium has no indication for the treatment of arrhythmias in the National Health Insurance. However, magnesium is effective for polymorphic VT (so‐called torsade de pointes)	Intravenous	Intravenous administration: magnesium is started at an initial dose of 20–40 mg/kg for 1–2 min. This regimen is followed by continuous drip infusion at a maintenance dose of 0.05–0.3 mg/kg/min

Antiarrhythmic agents, except for digoxin and flecainide, prescribed for children are off‐label use in the package inserts, because limited data on efficacy and safety are available. Tablet crushing and dose adjustment for body weight are also included as off‐label use.

**Table:** Table 79 Indication, Dosage and Administration of Anticoagulants

Type		Anticoagulant (Release year)	Indication with respect to National Health Insurance	Composition, formulation and dosage	Usage and administration
Vitamine K antagonist		warfarin1962	warfarin is indicated for the treatment and prevention of thromboembolic diseases, including ischemic stroke and systemic thromboembolism in all patients with AFIB. Pediatric use of warfarin includes the treatment and prevention of thromboembolic diseases such as venous thrombosis, myocardial infarction, pulmonary embolism, acute or insidious ischemic stroke etc	Granule: 0.2%Tablet: 0.5 mg, 1 mg, 5 mg	Dose of warfarin is adjusted according to the prothrombin time (PT). After initial loading, titration is performed within several days to determine the maintenance dose of warfarin based on the appropriate international normalized ratio of PT (PT‐INR)Initial dose is usually 1–5 mg once daily for adults. Maintenance dose for children is 0.16 mg/kg/day for infants (<12 months old) and 0.04–0.1 mg/kg/day for children aged 1–15 years old
DOAC	Direct thrombin inhibitor	dabigatran2011	dabigatran is indicated for the prevention of ischemic stroke and systemic thromboembolism in patients with non‐valvular AFIB	Capsule: 75 mg, 110 mg	Oral administration: dabigatran is administered at 300 mg/day twice daily for adults. However, dabigatran is adjusted to 220 mg/day twice daily in the case of (1) moderate renal dysfunction of CCr=30–50 mL/min, (2) age >70 years, (3) coadministration with P‐glycoprotein inhibitors, (4) episodes of gastrointestinal bleeding dabigatran is contraindicated in the case of severe renal dysfunction (CCr <30 mL/min)

	Xa inhibitor	rivaroxaban2012	rivaroxaban is indicated for the prevention of ischemic stroke and systemic thromboembolism in patients with non‐valvular AFIB rivaroxaban is also indicated for the treatment and secondary prevention of deep vein thrombosis and pulmonary thromboembolism	Tablet: 10 mg, 15 mgGranule: 10 mg, 15 mg	Oral administration: rivaroxaban is taken at 15 mg once daily for adults rivaroxaban is administered at 10 mg once daily in the case of moderate renal dysfunction (CCr=15–50 mL/min) rivaroxaban is contraindicated in the case of severe renal dysfunction (CCr <15 mL/min)
Xa inhibitor	apixaban2013	apixaban is indicated for the prevention of ischemic stroke and systemic thromboembolism in patients with non‐valvular AFIB apixaban is also indicated for the treatment and secondary prevention of deep vein thrombosis and pulmonary thromboembolism	Tablets: 2.5 mg, 5 mg	Oral administration: apixaban is taken at 10 mg/day twice daily for adults apixaban is administered at 5 mg/day twice daily if the patient meets at least 2 of the following conditions: (1) renal dysfunction of serum Cr >1.5 mg/dL, (2) body weight <60 kg or (3) age >80 years apixaban is contraindicated in the case of severe renal dysfunction (CCr <15 mL/min)

Xa Inhibitor	edoxaban2011	edoxaban is indicated for the prevention of ischemic stroke and systemic thromboembolism in patients with non‐valvular AFIB (2014) edoxaban is also indicated for the treatment and secondary prevention of deep vein thrombosis and pulmonary thromboembolism (2011)	Tablets: 15 mg, 30 mg, 60 mgOD tablets: 15 mg, 30 mg, 60 mg	Oral administration: edoxaban is taken at 60 mg once a day for adults. edoxaban is administered at 30 mg once a day if the patients meet at least one of the following conditions; 1) body weight <60 kg, 2) renal dysfunction of CCr=15–50 mL/min, or 3) coadministration with P‐glycoprotein inhibitors. edoxaban is contraindicated in the case of severe renal dysfunction of CCr <15 mL/min.

DOAC (direct oral anticoagulants) include a direct thrombin inhibitor and Xa inhibitors. There is no evidence for the efficacy and safety of under‐dosing of DOACs to adults who do not meet the criteria of low‐dose adjustment. The dosage and administration of Xa inhibitors are indicated for the prevention of ischemic stroke and systemic thromboembolism in patients with non‐valvular AFIB. DOACs are not available but warfarin is conventionally approved for pediatric use in the cases of hereditary thrombosis, AFIB after Fontan’s operation, giant coronary aneurysms observed as a sequela of Kawasaki disease and so on. The calculation of CCr is based on the Cockcroft‐Gault’s equation.

## Appendix 2. JCS / JHRS Joint Working Group 2

### 2.1

**Chair:**

- Katsushige Ono, Oita University School of Medicine, Pathophysiology

**Co‐Chair:**

- Yu‐ki Iwasaki, Department of Cardiovascular Medicine, Nippon Medical School

**Advisor:**

- Wataru Shimizu, Department of Cardiovascular Medicine, Nippon Medical School

**Members:**

- Masaharu Akao, Department of Cardiovascular Medicine, National Hospital Organization Kyoto Medical Center
- Tetsushi Furukawa, Department of Bio‐information Pharmacology, Medical Research Institute, Tokyo Medical and Dental University
- Nobuhisa Hagiwara, Department of Cardiology, Tokyo Women’s Medical University
- Ichiro Hisatome, Division of Cardiology, Yonago Medical Center
- Haruo Honjo, Research Institute of Environmental Medicine, Nagoya University
- Takanori Ikeda, Department of Cardiovascular Medicine, Toho University Graduate School of Medicine
- Yasuya Inden, Department of Cardiology, Nagoya University Graduate School of Medicine
- Kuniaki Ishii, Department of Pharmacology, Yamagata University Faculty of Medicine
- Yoshinori Kobayashi, Division of Cardiology, Department of Medicine, Tokai University Hachioji Hospital
- Yukihiro Koretsune, National Hospital Organization Osaka National Hospital
- Kengo Kusano, Department of Cardiovascular Medicine, National Cerebral and Cardiovascular Center
- Toru Maruyama, Department of Hematology, Oncology and Cardiovascular Medicine, Kyushu University Hospital
- Yuji Murakawa, The 4th Department of Internal Medicine, Teikyo University School of Medicine, Mizonokuchi Hospital
- Shinichi Niwano, Department of Cardiovascular Medicine, Kitasato University School of Medicine
- Tetsuo Sasano, Department of Cardiovascular Medicine, Tokyo Medical and Dental University
- Naokata Sumitomo, Department of Pediatric Cardiology, Saitama Medical University International Medical Center
- Naohiko Takahashi, Department of Cardiology and Clinical Examination, Faculty of Medicine, Oita University
- Eiichi Watanabe, Department of Cardiology, Fujita Health University School of Medicine
- Masahiro Yasaka, Department of Cerebrovascular Medicine and Neurology, Clinical Research Institute, National Hospital Organization Kyushu Medical Center

**Collaborators:**

- Takeshi Aiba, Department of Cardiovascular Medicine, National Cerebral and Cardiovascular Center
- Mari Amino, Department of Cardiovascular Medicine, Tokai University School of Medicine
- Chizuko Aoki‐Kamiya, Department of Obstetrics and Gynecology, National Cerebral and Cardiovascular Center
- Tadashi Fujino, Department of Cardiovascular Medicine, Toho University, Faculty of Medicine
- Masahide Harada, Department of Cardiology, Fujita Health University
- Noriyuki Hayami, Department of Fourth Internal Medicine, Teikyo University Mizonokuchi Hospital
- Hideki Itoh, Division of Patient Safety, Hiroshima University Hospital
- Jun Kishihara, Department of Cardiovascular Medicine, Kitasato University School of Medicine
- Eitaro Kodani, Department of Cardiovascular Medicine, Nippon Medical School Tama Nagayama Hospital
- Takashi Komatsu, Division of Cardiology, Department of Internal Medicine, Iwate Medical University School of Medicine
- Takeru Makiyama, Department of Cardiovascular Medicine, Graduate School of Medicine, Kyoto University
- Mitsunori Maruyama, Department of Cardiovascular Medicine, Nippon Medical School Musashi Kosugi Hospital
- Junichiro Miake, Department of Pharmacology, Tottori University Faculty of Medicine
- Norishige Morita, Division of Cardiology, Department of Medicine, Tokai University Hachioji Hospital
- Shota Muraji, Department of Pediatric Cardiology, Saitama Medical University International Medical Center
- Hiroshige Murata, Department of Cardiovascular Medicine, Nippon Medical School
- Satoshi Nagase, Department of Cardiovascular Medicine, National Cerebral and Cardiovascular Center
- Hisashi Ogawa, Department of Cardiology, National Hospital Organisation Kyoto Medical Center
- Yasuo Okumura, Division of Cardiology, Department of Medicine, Nihon University School of Medicine
- Yusuke Sakamoto, Department of Cardiology, Tosei General Hospital
- Kazuhiro Satomi, Department of Cardiology, Tokyo Medical University Hospital
- Yukio Sekiguchi, Department of Cardiology, National Hospital Organization Kasumigaura Medical Center
- Tsuyoshi Shiga, Department of Clinical Pharmacology and Therapeutics, The Jikei University School of Medicine
- Tetsuji Shinohara, Department of Cardiology and Clinical Examination, Faculty of Medicine, Oita University
- Atsushi Suzuki, Department of Cardiology, Tokyo Women’s Medical University
- Shinya Suzuki, Department of Cardiovascular Medicine, The Cardiovascular Institute
- Kenji Yodogawa, Department of Cardiovascular Medicine, Nippon Medical School
- Hisashi Yokoshiki, Department of Cardiovascular Medicine, Sapporo City General Hospital
- Koichiro Yoshioka, Division of Cardiology, Department of Internal Medicine, Tokai University School of Medicine

**Independent Assessment Committee:**

- Hiroshi Inoue, Saiseikai Toyama Hospital
- Takeshi Kimura, Department of Cardiovascular Medicine, Graduate School of Medicine, Kyoto University
- Ken Okumura, Division of Cardiology, Saiseikai Kumamoto Hospital Cardiovascular Center
- Hiroyuki Tsutsui, Department of Cardiovascular Medicine, Faculty of Medical Sciences, Kyushu University

(Listed in alphabetical order; affiliations as of December 2019)

## Appendix 3. Disclosure of Potential Conflicts of Interest (COI): JCS/JHRS 2020 Guideline on Pharmacotherapy of Cardiac Arrhythmias (2017/01/01–2019/12/31) 3

### 3.1

**Table:**

Author	Member’s own declaration items									COI of the marital partner, first‐degree family members, or those who share income and property			COI of the head of the organization/department to which the member belongs (if the member is in a position to collaborate with the head of the organization/department)	
	Employer/leadership position (private company)	Stakeholder	Patent royalty	Honorarium	Payment for manuscripts	Research grant	Scholarship (educational) grant	Endowed chair	Other rewards	Employer/leadership position (private company)	Stakeholder	Patent royalty	Research grant	Scholarship (educational) grant
Co‐Chair: Yu‐ki Iwasaki						Daiichi Sankyo Company, Limited								Daiichi Sankyo Company, Limited
Advisor: Wataru Shimizu				Bayer Yakuhin, Ltd. Pfizer Japan Inc. Bristol‐Myers Squibb Ono Pharmaceutical Co., Ltd. Daiichi Sankyo Company, Limited Nippon Boehringer Ingelheim Co., Ltd.		Daiichi Sankyo Company, Limited Nippon Boehringer Ingelheim Co., Ltd.	Astellas Pharma Inc. Abbott Medical Japan L.L.C Eisai Co., Ltd. St. Jude Medical Japan Co., Ltd. Bayer Yakuhin, Ltd. Bristol‐Myers Squibb Ono Pharmaceutical Co., Ltd. Otsuka Pharmaceutical Co., Ltd. Daiichi Sankyo Company, Limited Mitsubishi Tanabe Pharma Corporation Nippon Boehringer Ingelheim Co., Ltd.							
Members: Masaharu Akao				Daiichi Sankyo Company, Limited Bayer Yakuhin, Ltd. Bristol‐Myers Squibb		Bayer Yakuhin, Ltd.	Bayer Yakuhin, Ltd. Daiichi Sankyo Company, Limited							
Members: Nobuhisa Hagiwara				Bayer Yakuhin, Ltd. Bristol‐Myers Squibb Nippon Boehringer Ingelheim Co., Ltd.			Aegerion Pharmaceuticals, Inc. Astellas Pharma Inc. Bayer Yakuhin, Ltd. Pfizer Japan Inc. Daiichi Sankyo Company, Limited Mochida Pharmaceutical Co.,Ltd. Otsuka Pharmaceutical Co., Ltd. Nippon Boehringer Ingelheim Co., Ltd. Takeda Pharmaceutical Company Limited							
Members: Ichiro Hisatome				Pfizer Japan Inc. Sanwa Kagaku Kenkyusho Co., Ltd. Mochida Pharmaceutical Co.,Ltd. Teijin Pharma Limited FUJI YAKUHIN CO., LTD.		Sanwa Kagaku Kenkyusho Co., Ltd. FUJI YAKUHIN CO., LTD.	Astellas Pharma Inc. Novartis Pharma K.K. Bayer Yakuhin, Ltd. Pfizer Japan Inc. Sanwa Kagaku Kenkyusho Co., Ltd. Mochida Pharmaceutical Co.,Ltd. Teijin Pharma Limited FUJI YAKUHIN CO., LTD.							
Members: Takanori Ikeda				TOA EIYO LTD. Bayer Yakuhin, Ltd. Pfizer Japan Inc. Bristol‐Myers Squibb Nippon Boehringer Ingelheim Co., Ltd. Ono Pharmaceutical Co., Ltd. Daiichi Sankyo Company, Limited			Bayer Yakuhin, Ltd. Daiichi Sankyo Company, Limited Medtronic Japan Co., Ltd. Japan Lifeline Co.,Ltd.							
Members: Yasuya Inden				Daiichi Sankyo Company, Limited Medtronic Japan Co., Ltd. Bristol‐Myers Squibb										Teijin Pharma Limited Mitsubishi Tanabe Pharma Corporation MSD K.K. Astellas Pharma Inc. Takeda Pharmaceutical Company Limited Daiichi Sankyo Company, Limited
Members: Yoshinori Kobayashi	Fukuda Denshi Co., Ltd Kowa Company, Ltd.,			Nippon Boehringer Ingelheim Co., Ltd. Daiichi Sankyo Company, Limited			Abbott Medical Japan L.L.C St. Jude Medical Japan Co., Ltd. Bayer Yakuhin, Ltd. Daiichi Sankyo Company, Limited Nippon Boehringer Ingelheim Co., Ltd.							
Members: Yukihiro Koretsune				Bristol‐Myers Squibb Daiichi Sankyo Company, Limited										
Members: Kengo Kusano				Abbott Vascular Japan Co., Ltd. Bayer Yakuhin, Ltd. Bristol‐Myers Squibb Medtronic Japan Co., Ltd. Daiichi Sankyo Company, Limited		EP‐CRSU Co., Ltd. EPS Corporation Boston Scientific Japan K.K. Medtronic Japan Co., Ltd.								
Members: Yuji Murakawa				Daiichi Sankyo Company, Limited Bayer Yakuhin, Ltd. Bristol‐Myers Squibb										
Members: Shinichi Niwano				Daiichi Sankyo Company, Limited	Daiichi Sankyo Company, zLimited		Bristol‐Myers Squibb Nippon Boehringer Ingelheim Co., Ltd. Daiichi Sankyo Company, Limited	Nippon Boehringer Ingelheim Co., Ltd. Daiichi Sankyo Company, Limited						Bayer Yakuhin, Ltd. Daiichi Sankyo Company, Limited Nippon Boehringer Ingelheim Co., Ltd.
Members: Tetsuo Sasano						Daiichi Sankyo Company, Limited	Nippon Boehringer Ingelheim Co., Ltd.							
Members: Naohiko Takahashi				Bayer Yakuhin, Ltd. Pfizer Japan Inc. Bristol‐Myers Squibb Nippon Boehringer Ingelheim Co., Ltd. Daiichi Sankyo Company, Limited Mitsubishi Tanabe Pharma Corporation		Ono Pharmaceutical Co., Ltd.								
Members: Eiichi Watanabe				Nippon Boehringer Ingelheim Co., Ltd. Daiichi Sankyo Company, Limited			Medtronic Japan Co., Ltd. Boston Scientific Japan K.K. BIOTRONIK Japan, Inc. St. Jude Medical Japan Co., Ltd. Daiichi Sankyo Company, Limited						Bayer Yakuhin, Ltd. Research Institute for Production Development	
Members: Masahiro Yasaka				CSL Behring K.K. Bayer Yakuhin, Ltd. Pfizer Japan Inc. Bristol‐Myers Squibbn Daiichi Sankyo Company, Limited Nippon Boehringer Ingelheim Co., Ltd. Takeda Pharmaceutical Company Limited		Nippon Boehringer Ingelheim Co., Ltd.	Nippon Boehringer Ingelheim Co., Ltd.							
Collaborators: Takeshi Aiba				Ono Pharmaceutical Co., Ltd. Bristol‐Myers Squibb			Daiichi Sankyo Company, Limited Nippon Boehringer Ingelheim Co., Ltd.	Medtronic Japan Co., Ltd.						
Collaborators: Masahide Harada				Johnson & Johnson K.K. Bristol‐Myers Squibb Daiichi Sankyo Company, Limited Nippon Boehringer Ingelheim Co., Ltd.										
Collaborators: Hideki Itoh						Bristol‐Myers Squibb								
Collaborators: Eitaro Kodani				Ono Pharmaceutical Co., Ltd. Bristol‐Myers Squibb Daiichi Sankyo Company, Limited			Ono Pharmaceutical Co., Ltd.							
Collaborators: Takashi Komatsu				Daiichi Sankyo Company, Limited Nippon Boehringer Ingelheim Co., Ltd.		Daiichi Sankyo Company, Limited	Daiichi Sankyo Company, Limited							
Collaborators: Takeru Makiyama				Daiichi Sankyo Company, Limited		Nissan Chemical Corporation							EP‐CRSU Co., Ltd. Daiichi Sankyo Company, Limited	Otsuka Pharmaceutical Co., Ltd. Daiichi Sankyo Company, Limited
Collaborators: Norishige Morita	Fukuda Denshi Co., Ltd			Daiichi Sankyo Company, Limited										
Collaborators: Satoshi Nagase								Medtronic Japan Co., Ltd.					EP‐CRSU Co., Ltd.	
Collaborators: Hisashi Ogawa				Bayer Yakuhin, Ltd. BIOTRONIK Japan, Inc. Pfizer Japan Inc. Bristol‐Myers Squibb Nippon Boehringer Ingelheim Co., Ltd. Daiichi Sankyo Company, Limited Medtronic Japan Co., Ltd.	Daiichi Sankyo Company, LimitedNippon Boehringer Ingelheim Co., Ltd.									
Collaborators: Yasuo Okumura				Daiichi Sankyo Co., Ltd. Bayer Yakuhin, Ltd. Bristol‐Myers Squibb Nippon Boehringer Ingelheim Co., Ltd.		Bristol‐Myers Squibb Amgen Astellas BioPharma K.K. A2 Healthcare Corporation Bayer Yakuhin, Ltd.	Astellas Pharma Inc. Sanofi K.K. Johnson & Johnson K.K. Otsuka Pharmaceutical Co., Ltd. Daiichi Sankyo Company, Limited Nihon Medi‐Physics Co.,Ltd.	Boston Scientific Japan K.K. Medtronic Japan Co., Ltd. Japan Lifeline Co.,Ltd. Nihon Kohden Corp. Abbott Medical Japan L.L.C						
Collaborators: Kazuhiro Satomi				Abbott Medical Japan L.L.C Johnson & Johnson K.K. St. Jude Medical Japan Co., Ltd. Bayer Yakuhin, Ltd. Bristol‐Myers Squibb Daiichi Sankyo Company, Limited Nippon Boehringer Ingelheim Co., Ltd. Medtronic Japan Co., Ltd. Japan Lifeline Co., Ltd.				BIOTRONIK Japan, Inc.						
Collaborators: Yukio Sekiguchi	Fukuda Denshi Co., Ltd							St. Jude Medical Japan Co., Ltd. Abbott Japan LLC						
Collaborators: Tsuyoshi Shiga				Eisai Co., Ltd. TOA EIYO LTD. Bayer Yakuhin, Ltd. Bristol‐Myers Squibb Ono Pharmaceutical Co., Ltd. Daiichi Sankyo Company, Limited		Daiichi Sankyo Company, Limited	Eisai Co., Ltd. Ono Pharmaceutical Co., Ltd.							
Collaborators: Shinya Suzuki				Daiichi Sankyo Company, Limited Bristol‐Myers Squibb		Daiichi Sankyo Company, Limited	Nippon Boehringer Ingelheim Co., Ltd.						Bristol‐Myers Squibb	
Collaborators: Koichiro Yoshioka							Accuray Japan K.K. DVx Inc. Bayer Yakuhin, Ltd. Mebix, Inc. Daiichi Sankyo Company, Limited							
Independent Assessment Committee: Hiroshi Inoue				Daiichi Sankyo Company, Limited Nippon Boehringer Ingelheim Co., Ltd. Bayer Yakuhin, Ltd. Bristol‐Myers Squibb										
Independent Assessment Committee: Takeshi Kimura				Amgen Astellas BioPharma K.K. Abbott Vascular Japan Co., Ltd. Sanofi K.K. Bristol‐Myers Squibb Boston Scientific Japan K.K. Kowa Pharmaceutical Co., Ltd. Daiichi Sankyo Company, Limited Nippon Boehringer Ingelheim Co., Ltd.		Nipro Corporation EP‐CRSU Co., Ltd. Edwards Lifesciences Corporation Daiichi Sankyo Company, Limited Pfizer Japan Inc.	Astellas Pharma Inc. Otsuka Pharmaceutical Co., Ltd. Daiichi Sankyo Company, Limited Mitsubishi Tanabe Pharma Corporation Nippon Boehringer Ingelheim Co., Ltd. Takeda Pharmaceutical Company Limited							
Independent Assessment Committee: Ken Okumura				Daiichi Sankyo Company, Limited Nippon Boehringer Ingelheim Co., Ltd. Johnson & Johnson K.K. Medtronic Japan Co., Ltd. Bayer Yakuhin, Ltd. Bristol‐Myers Squibb										
Independent Assessment Committee: Hiroyuki Tsutsui				MSD K.K. Novartis Pharma K.K. Bayer Yakuhin, Ltd. Pfizer Japan Inc. Bristol‐Myers Squibb Otsuka Pharmaceutical Co., Ltd. Daiichi Sankyo Company, Limited Teijin Pharma Limited Mitsubishi Tanabe Pharma Corporation Nippon Boehringer Ingelheim Co., Ltd. Takeda Pharmaceutical Company Limited	nippon rinsho Co.,Ltd.	IQVIA Services Japan K.K. Actelion Pharmaceuticals Japan Ltd. OMRON HEALTHCARE Co., Ltd. Daiichi Sankyo Company, Limited Mitsubishi Tanabe Pharma Corporation Japan Tobacco Inc. Nippon Boehringer Ingelheim Co., Ltd.	MSD K.K. Daiichi Sankyo Company, Limited Teijin Pharma Limited Mitsubishi Tanabe Pharma Corporation							

^*^ The following persons have no conflict of interest to declare:

Chair: Katsushige Ono, none

Members: Tetsushi Furukawa, none

Members: Haruo Honjo, none

Members: Kuniaki Ishii, none

Members: Toru Maruyama, none

Members: Naokata Sumitomo, none

Collaborators: Mari Amino, none

Collaborators: Chizuko Aoki‐Kamiya, none

Collaborators: Tadashi Fujino, none

Collaborators: Noriyuki Hayami, none

Collaborators: Jun Kishihara, none

Collaborators: Mitsunori Maruyama, none

Collaborators: Junichiro Miake, none

Collaborators: Shota Muraji, none

Collaborators: Hiroshige Murata, none

Collaborators: Yusuke Sakamoto, none

Collaborators: Tetsuji Shinohara, none

Collaborators: Atsushi Suzuki, none

Collaborators: Kenji Yodogawa, none

Collaborators: Hisashi Yokoshiki, none
